# Supplementary material for: Evaluation of Common Musculoskeletal Injuries in the Urgent Setting
Source: MedEdPORTAL. 2016 Dec 7;12:10514. doi: 10.15766/mep_2374-8265.10514 (PMC6440529; doi:10.15766/mep_2374-8265.10514)
Supplement: Supplementary file 1 — A. Evaluation of Common Musculoskeletal Injuries in the Urgent Setting.pptx B. Evaluation of Ankle Injuries in the Urgent Setting.pptx C. Evaluation of Hip Injuries in the Urgent Setting.pptx D. Evaluation of Shoulder Injuries in the Urgent Setting.pptx E. Evaluation of Wrist Injuries in the Urgent Setting.pptx [file mep-12-10514-s001.zip › E. Evaluation of Wrist Injuries in the Urgent Setting.pptx]

## Slide 1
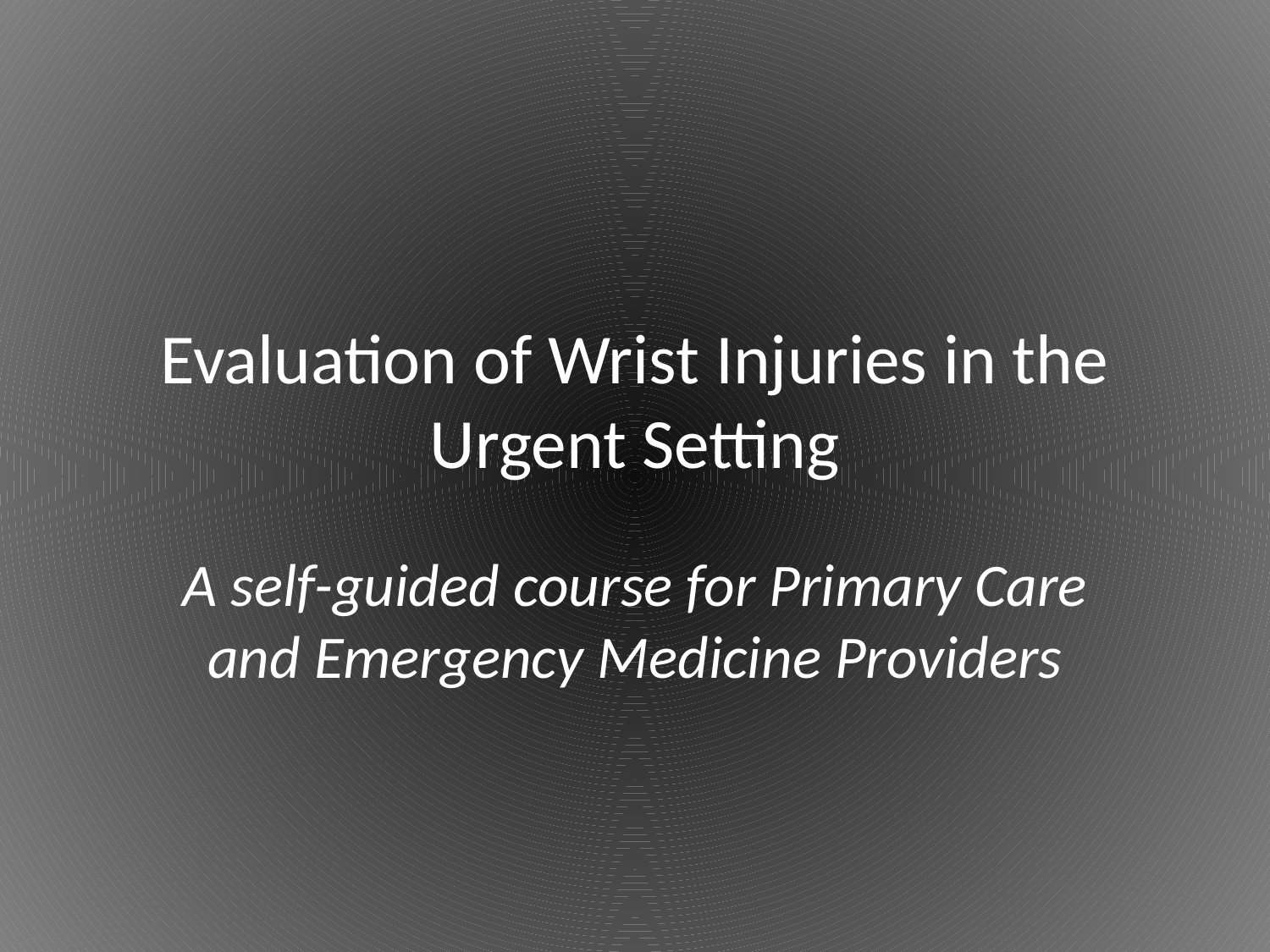

# Evaluation of Wrist Injuries in the Urgent Setting
A self-guided course for Primary Care and Emergency Medicine Providers

## Slide 2
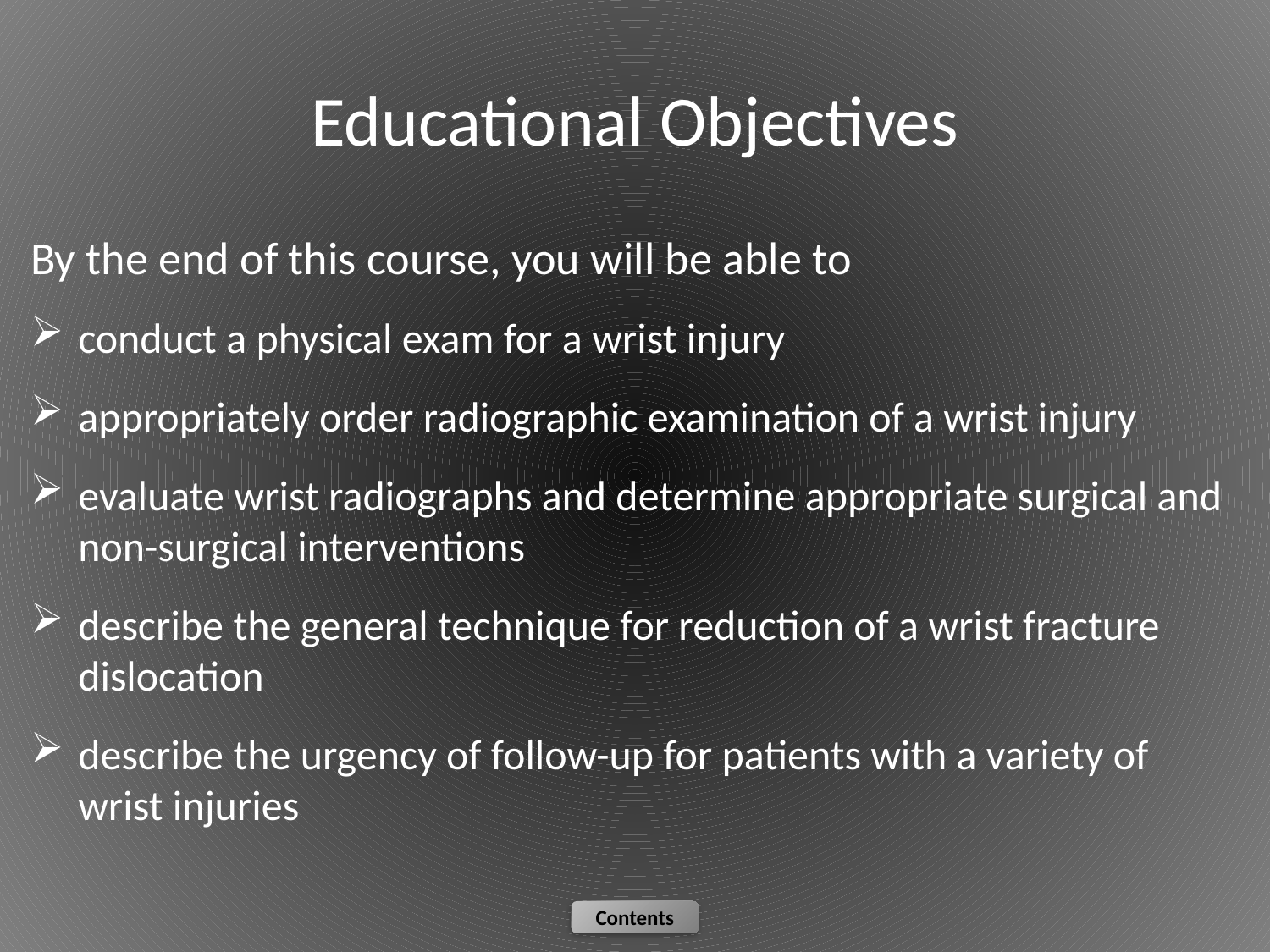

# Educational Objectives
By the end of this course, you will be able to
conduct a physical exam for a wrist injury
appropriately order radiographic examination of a wrist injury
evaluate wrist radiographs and determine appropriate surgical and non-surgical interventions
describe the general technique for reduction of a wrist fracture dislocation
describe the urgency of follow-up for patients with a variety of wrist injuries
Contents

## Slide 3
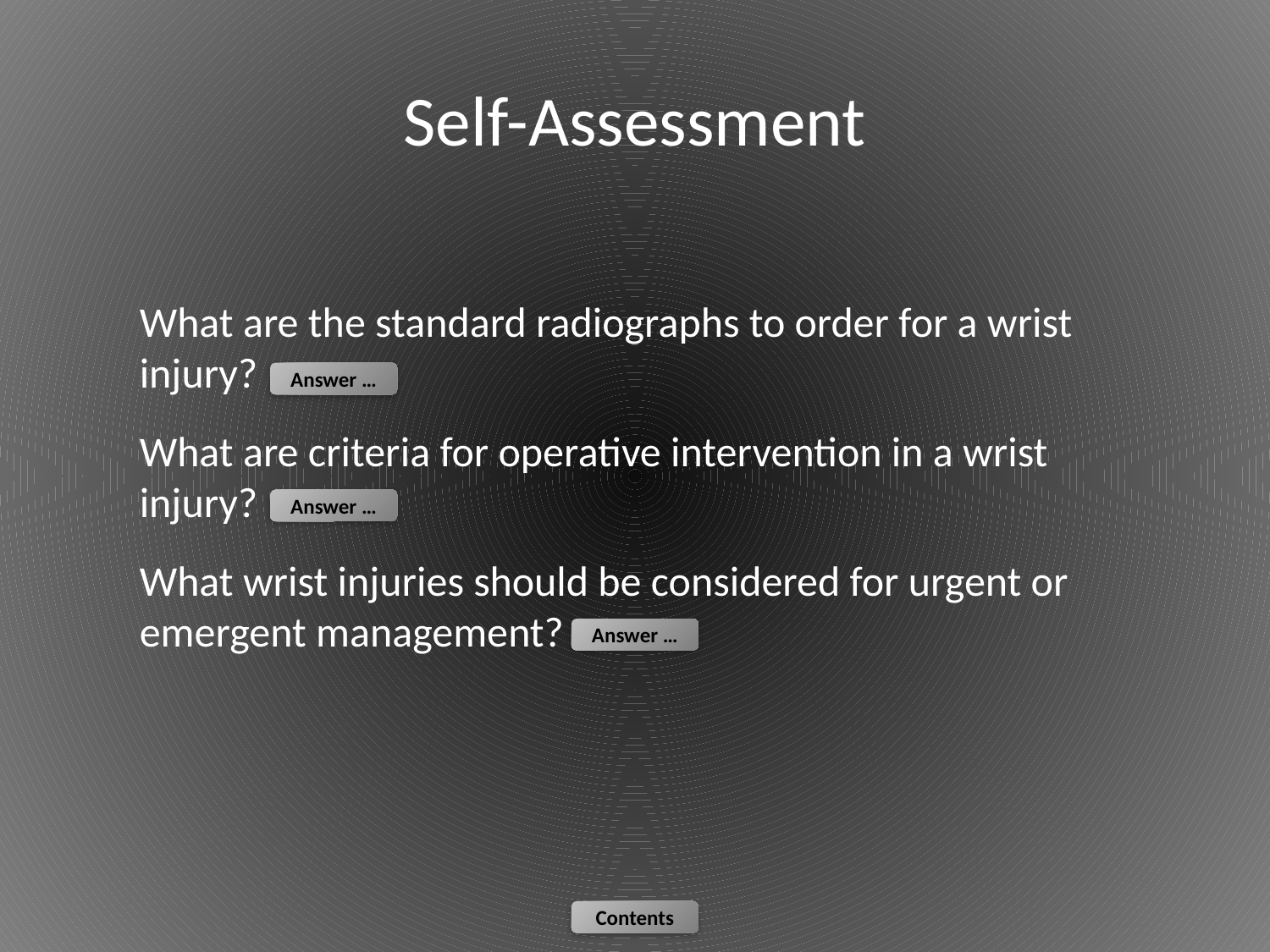

# Self-Assessment
What are the standard radiographs to order for a wrist injury?
What are criteria for operative intervention in a wrist injury?
What wrist injuries should be considered for urgent or emergent management?
Answer …
Answer …
Answer …
Contents

## Slide 4
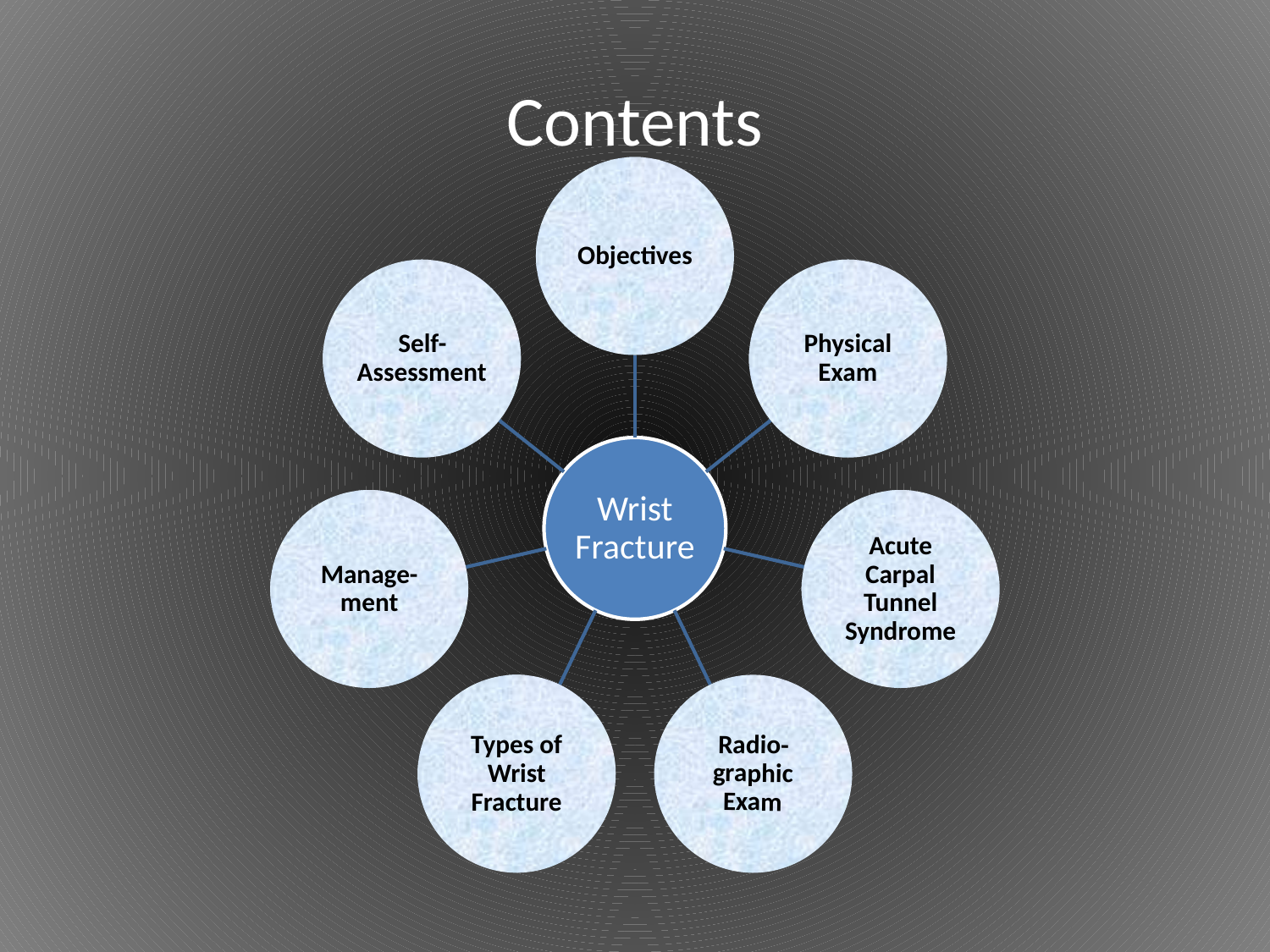

# Contents

## Slide 5
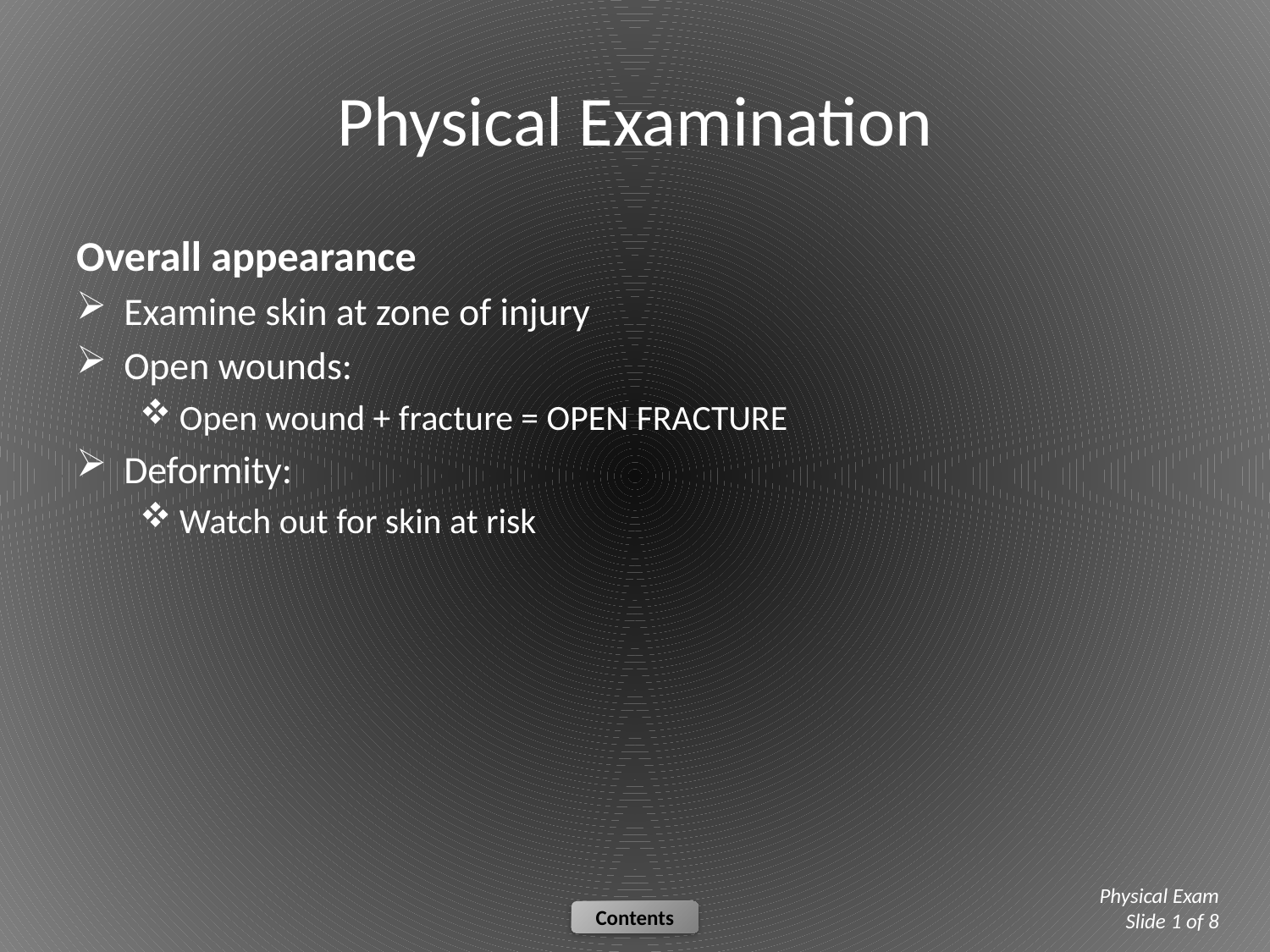

# Physical Examination
Overall appearance
Examine skin at zone of injury
Open wounds:
Open wound + fracture = OPEN FRACTURE
Deformity:
Watch out for skin at risk
Physical Exam
Slide 1 of 8
Contents

## Slide 6
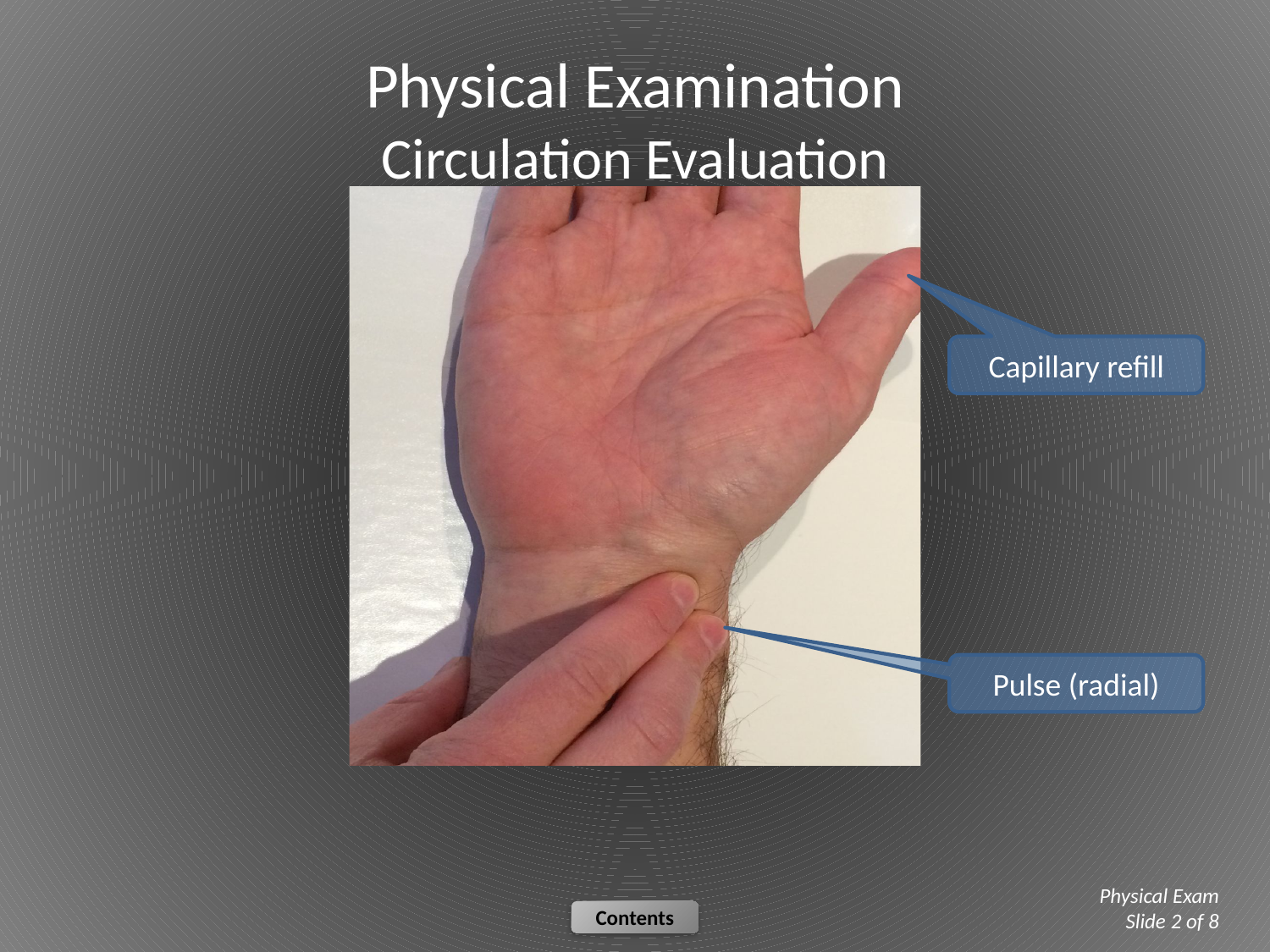

# Physical Examination Circulation Evaluation
Capillary refill
Pulse (radial)
Physical Exam
Slide 2 of 8
Contents

## Slide 7
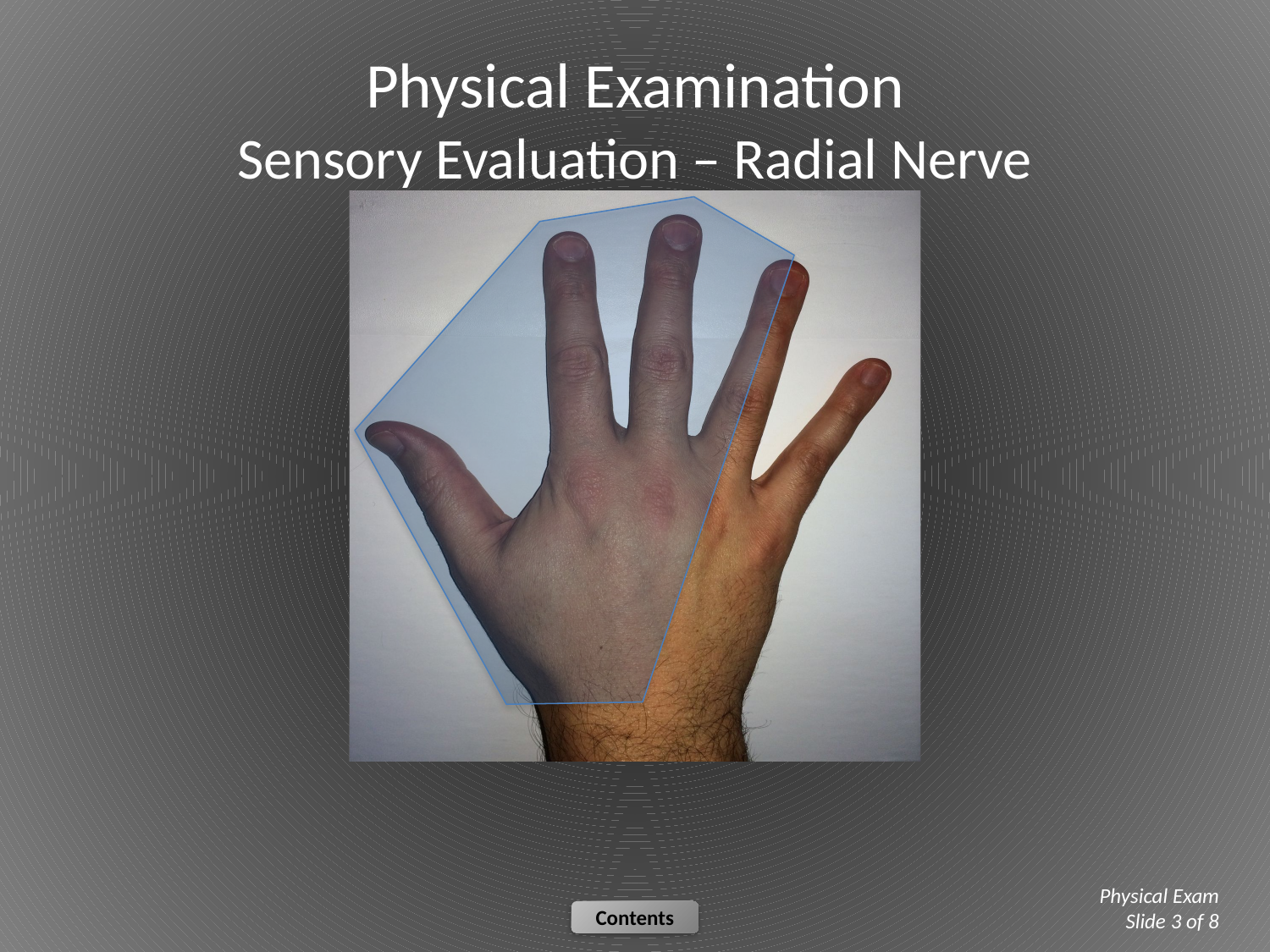

# Physical Examination Sensory Evaluation – Radial Nerve
Physical Exam
Slide 3 of 8
Contents

## Slide 8
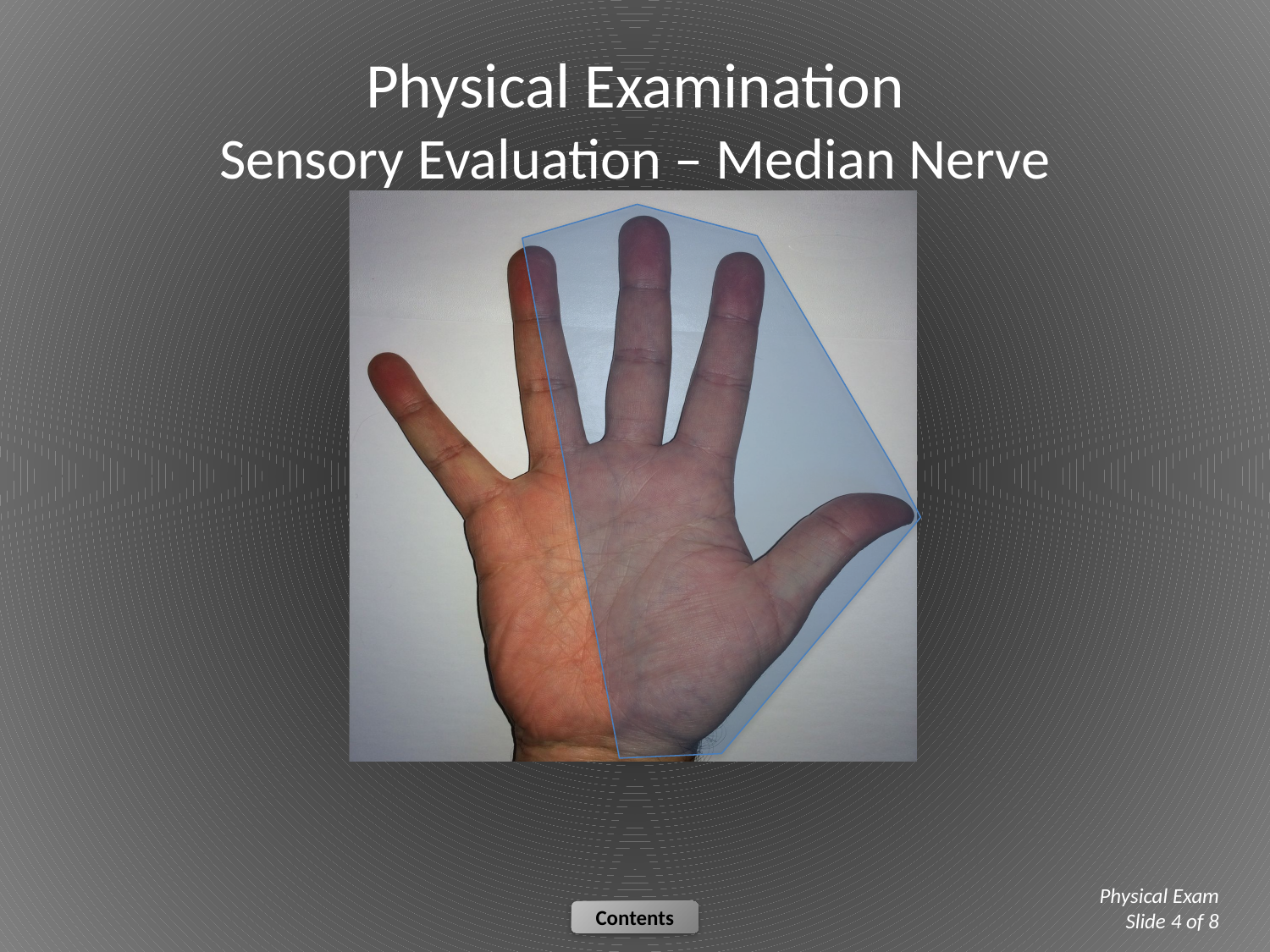

# Physical Examination Sensory Evaluation – Median Nerve
Physical Exam
Slide 4 of 8
Contents

## Slide 9
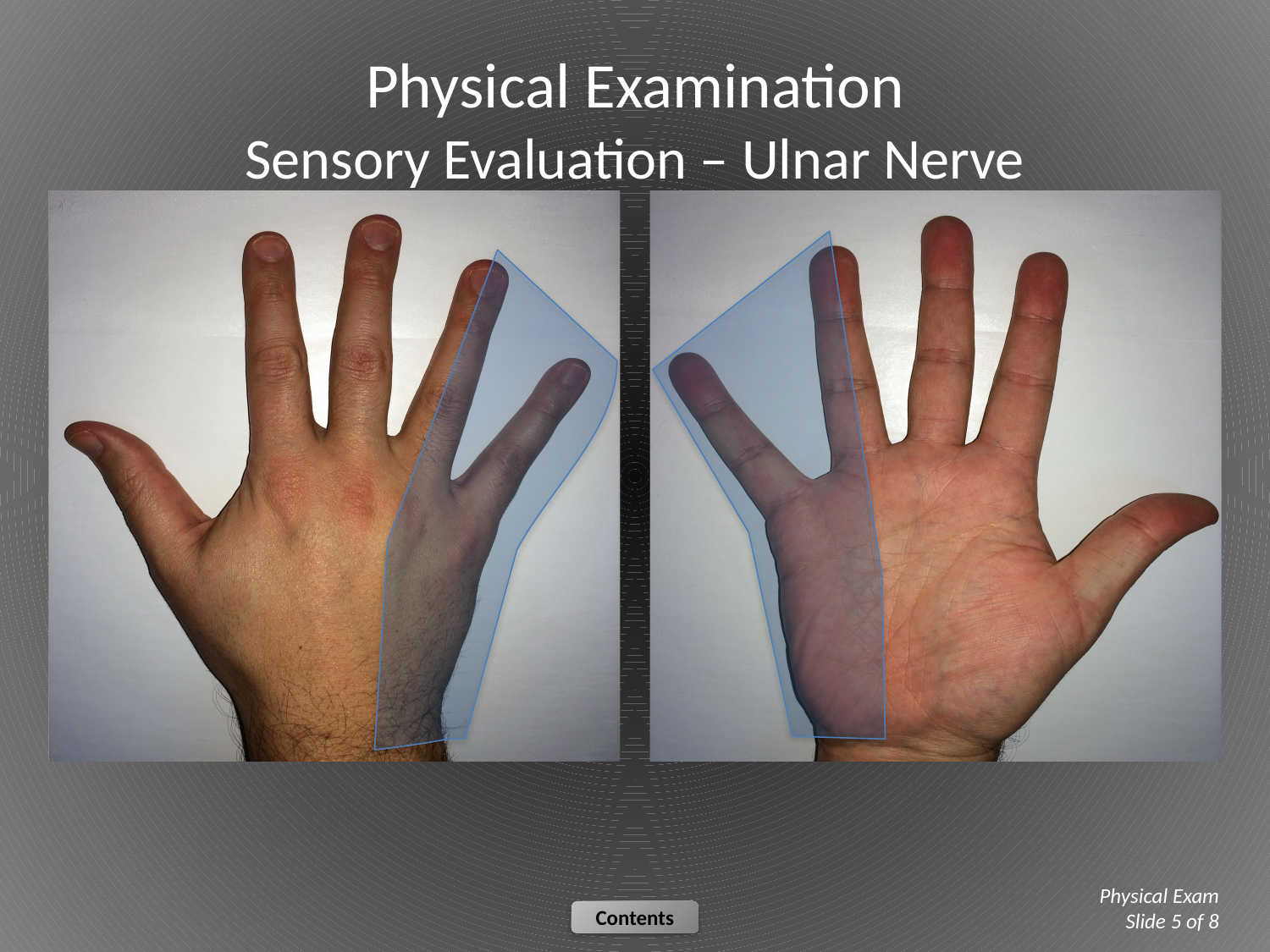

# Physical Examination Sensory Evaluation – Ulnar Nerve
Physical Exam
Slide 5 of 8
Contents

## Slide 10
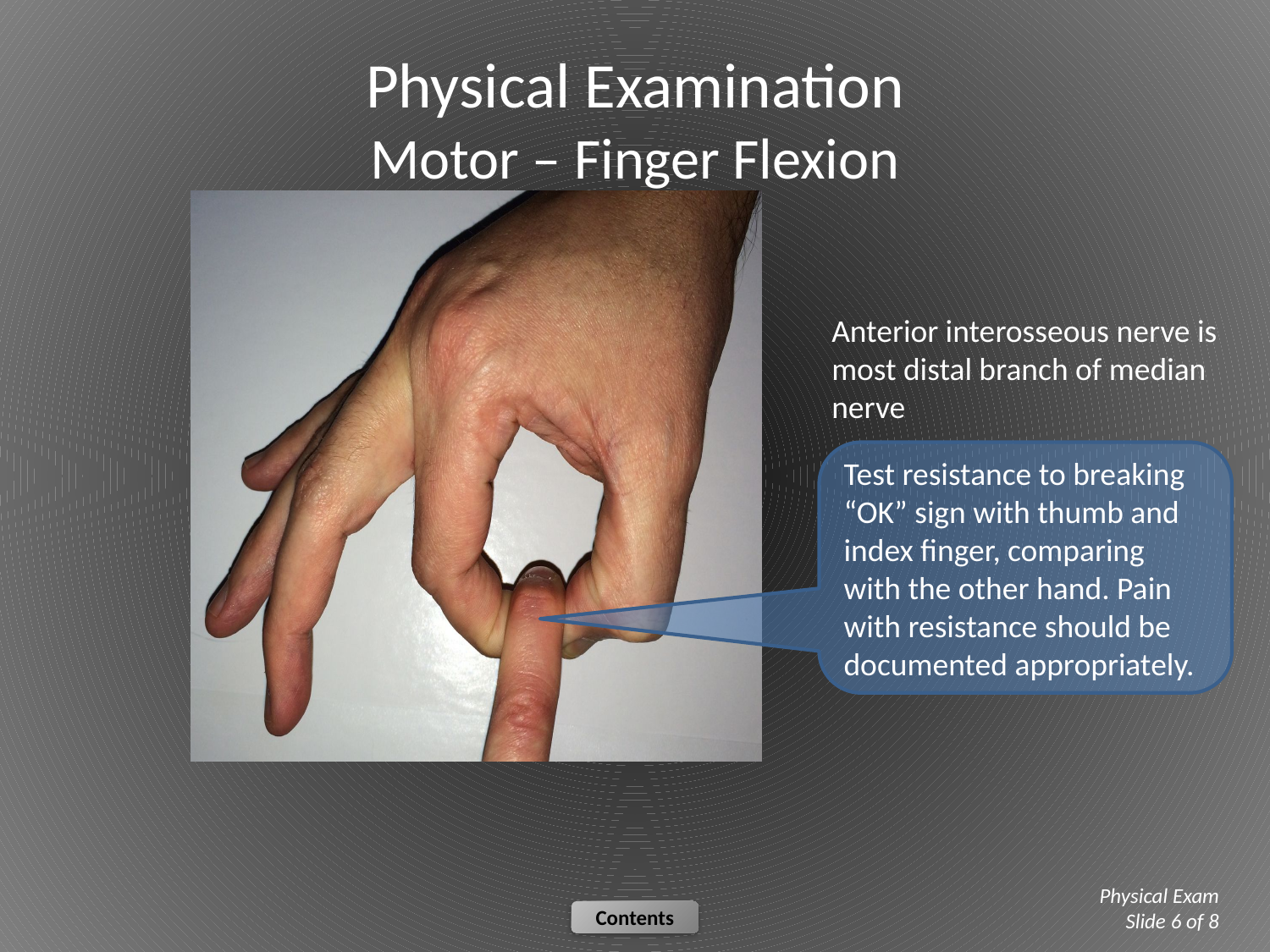

# Physical Examination Motor – Finger Flexion
Anterior interosseous nerve is most distal branch of median nerve
Test resistance to breaking “OK” sign with thumb and index finger, comparing with the other hand. Pain with resistance should be documented appropriately.
Physical Exam
Slide 6 of 8
Contents

## Slide 11
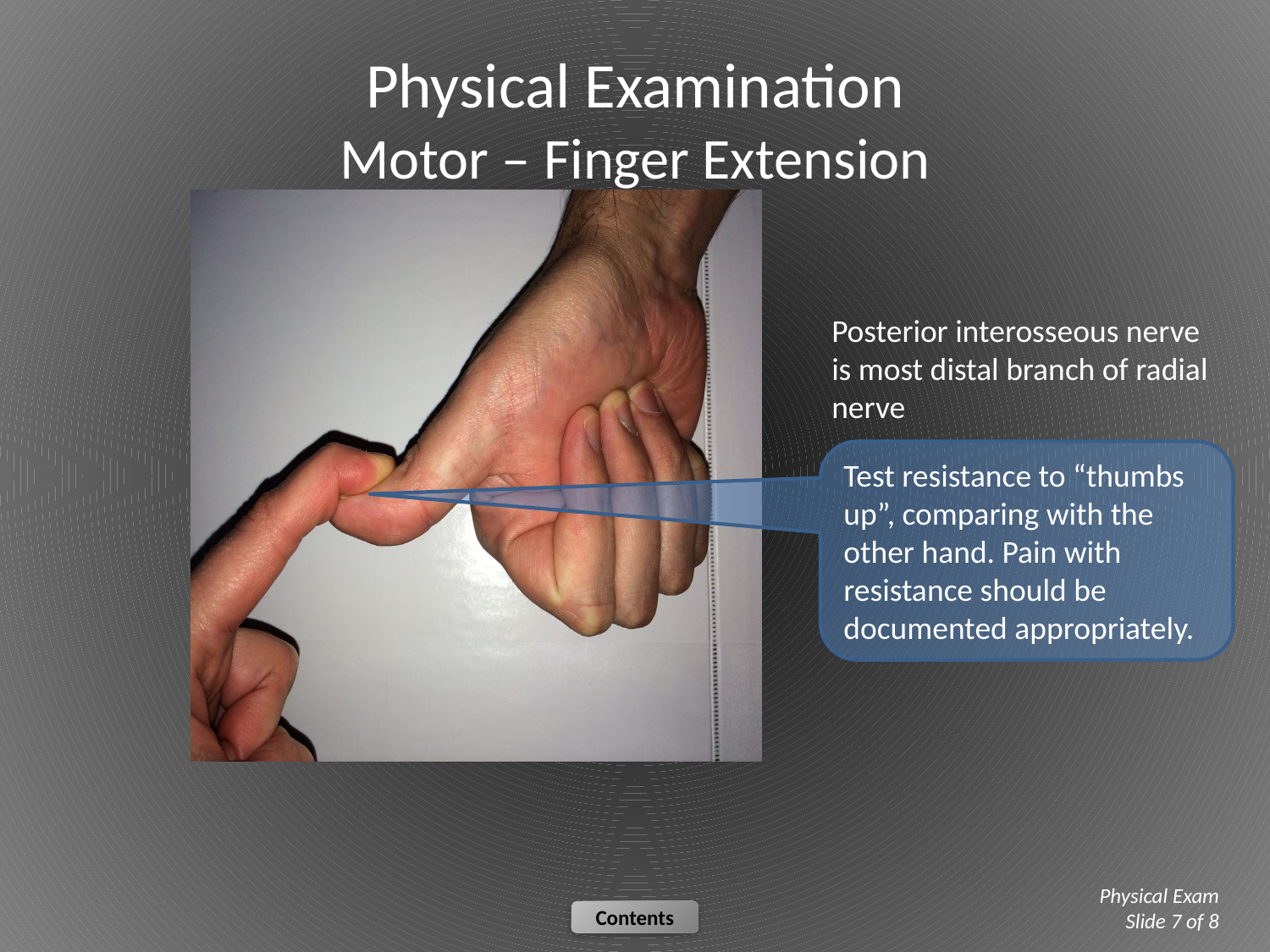

# Physical Examination Motor – Finger Extension
Posterior interosseous nerve is most distal branch of radial nerve
Test resistance to “thumbs up”, comparing with the other hand. Pain with resistance should be documented appropriately.
Physical Exam
Slide 7 of 8
Contents

## Slide 12
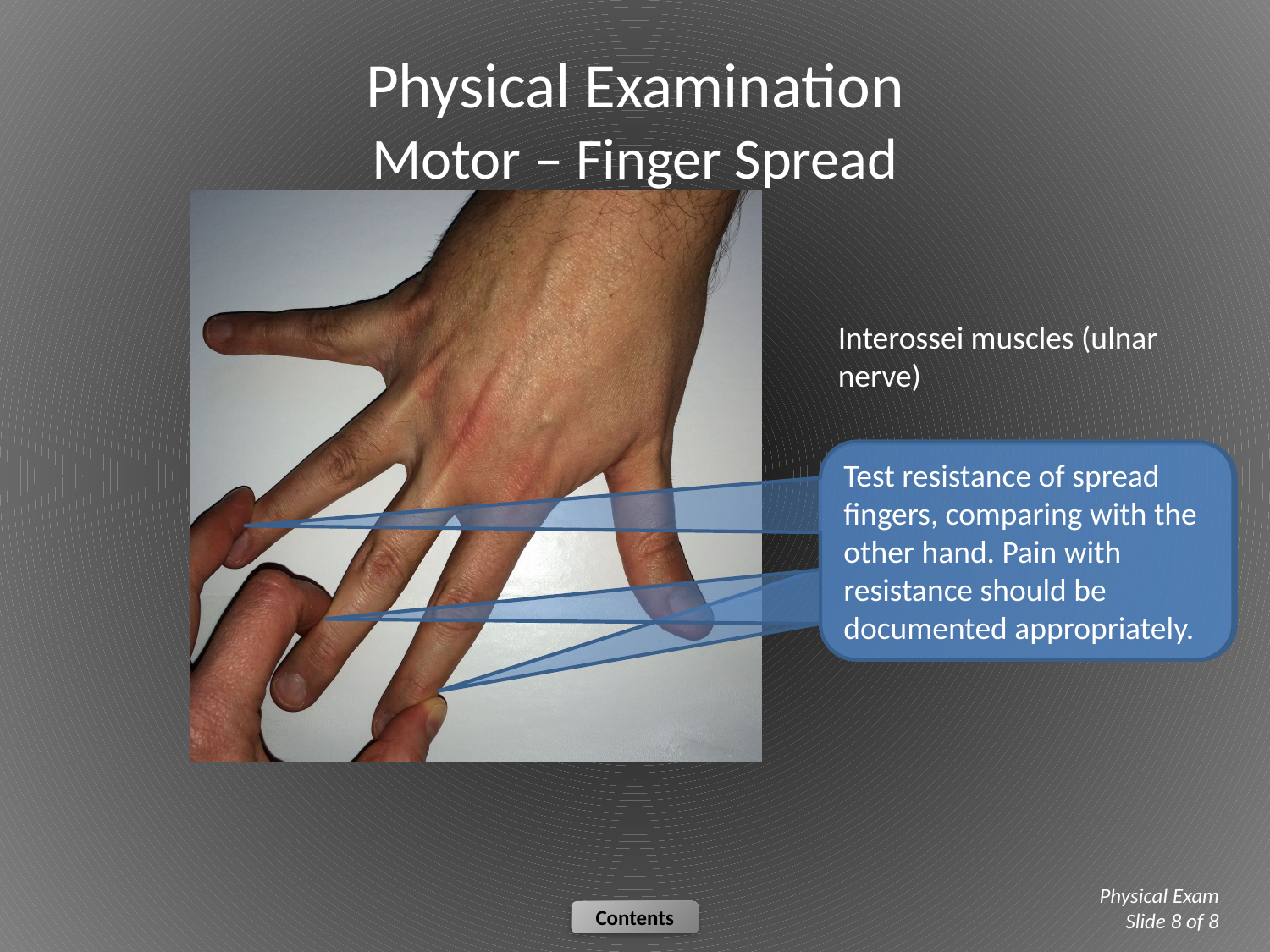

# Physical Examination Motor – Finger Spread
Interossei muscles (ulnar nerve)
Test resistance of spread fingers, comparing with the other hand. Pain with resistance should be documented appropriately.
Physical Exam
Slide 8 of 8
Contents

## Slide 13
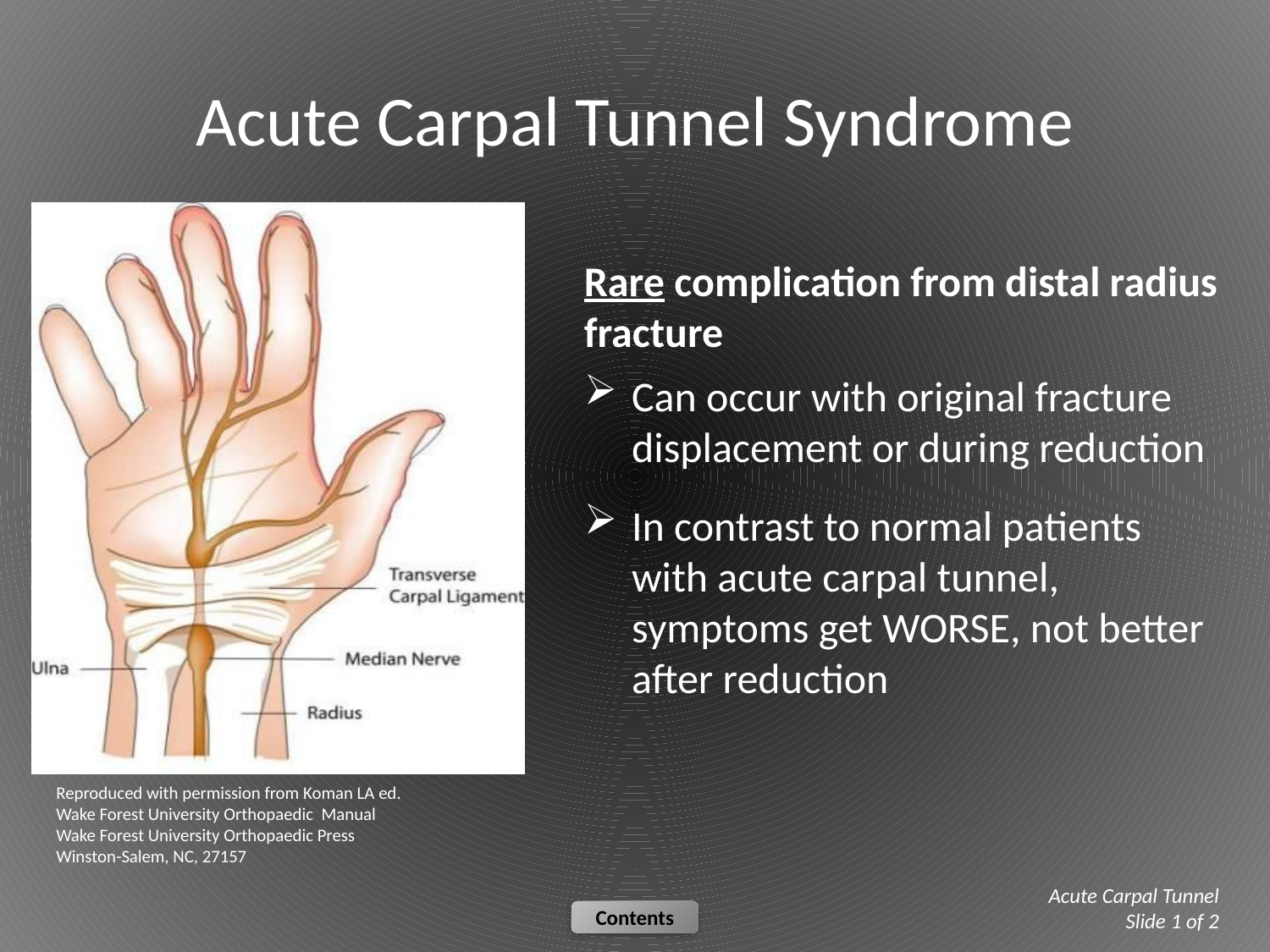

# Acute Carpal Tunnel Syndrome
Rare complication from distal radius fracture
Can occur with original fracture displacement or during reduction
In contrast to normal patients with acute carpal tunnel, symptoms get WORSE, not better after reduction
Reproduced with permission from Koman LA ed.
Wake Forest University Orthopaedic Manual
Wake Forest University Orthopaedic Press
Winston-Salem, NC, 27157
Acute Carpal Tunnel
Slide 1 of 2
Contents

## Slide 14
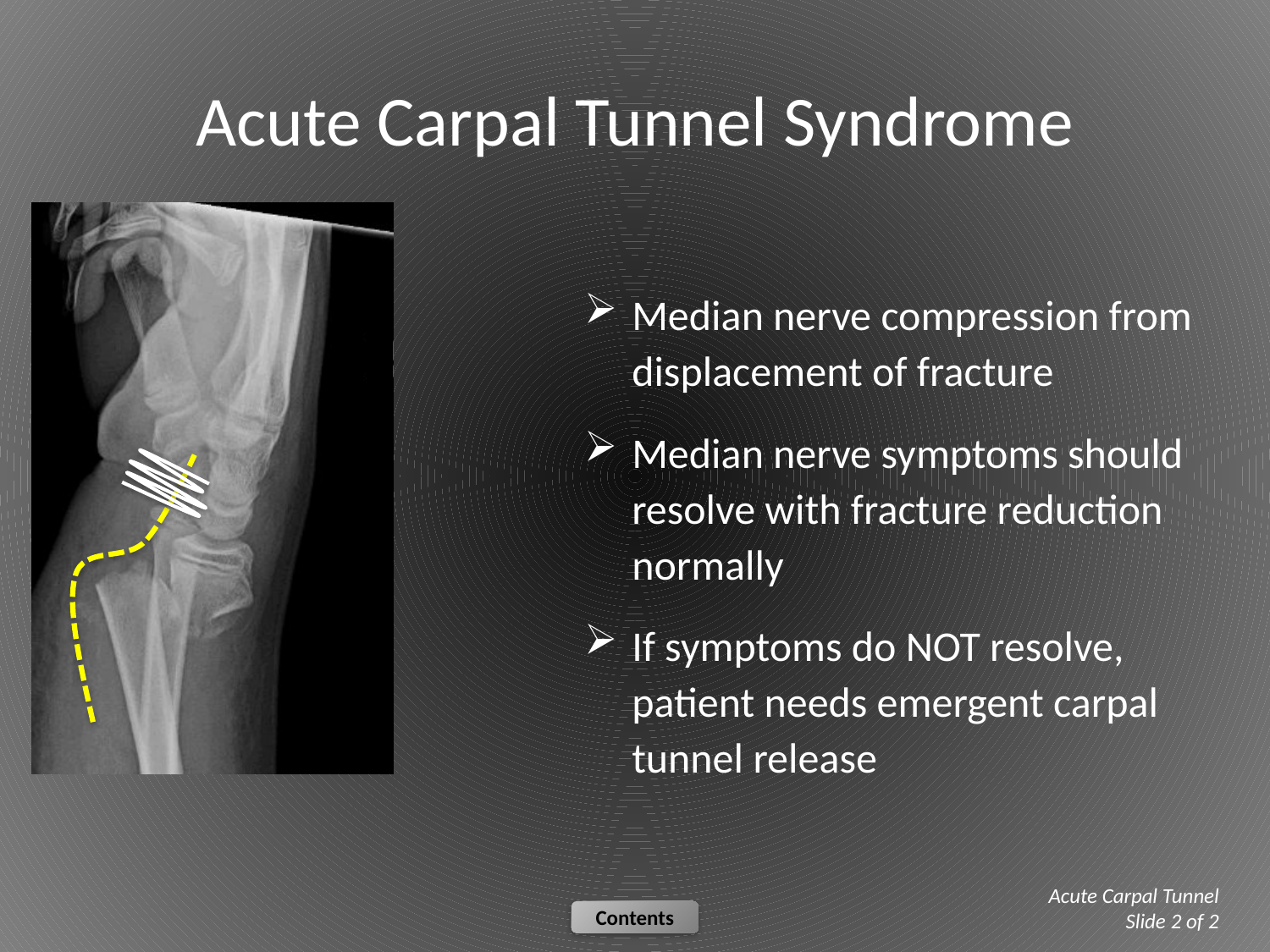

# Acute Carpal Tunnel Syndrome
Median nerve compression from displacement of fracture
Median nerve symptoms should resolve with fracture reduction normally
If symptoms do NOT resolve, patient needs emergent carpal tunnel release
Acute Carpal Tunnel
Slide 2 of 2
Contents

## Slide 15
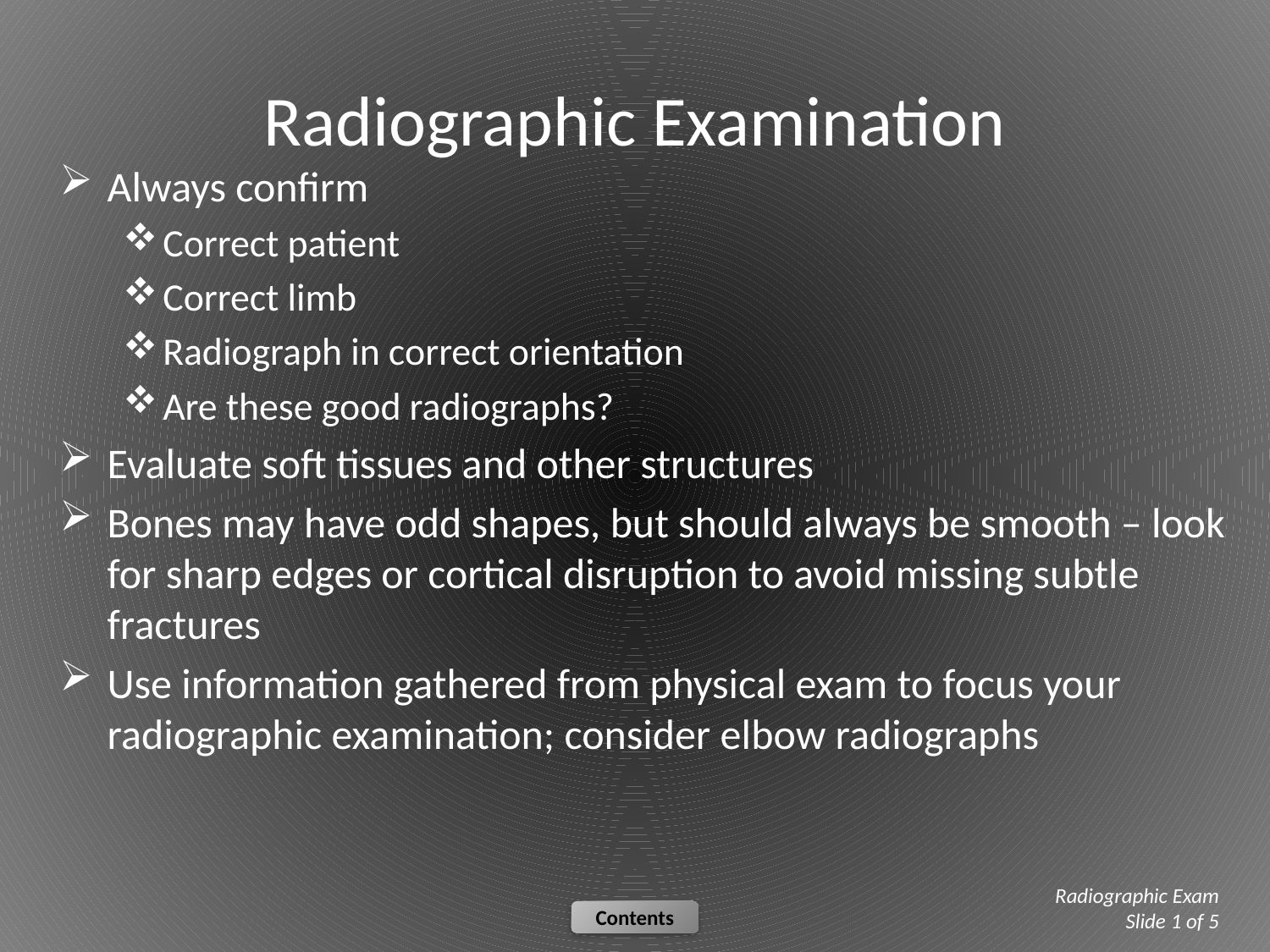

# Radiographic Examination
Always confirm
Correct patient
Correct limb
Radiograph in correct orientation
Are these good radiographs?
Evaluate soft tissues and other structures
Bones may have odd shapes, but should always be smooth – look for sharp edges or cortical disruption to avoid missing subtle fractures
Use information gathered from physical exam to focus your radiographic examination; consider elbow radiographs
Radiographic Exam
Slide 1 of 5
Contents

## Slide 16
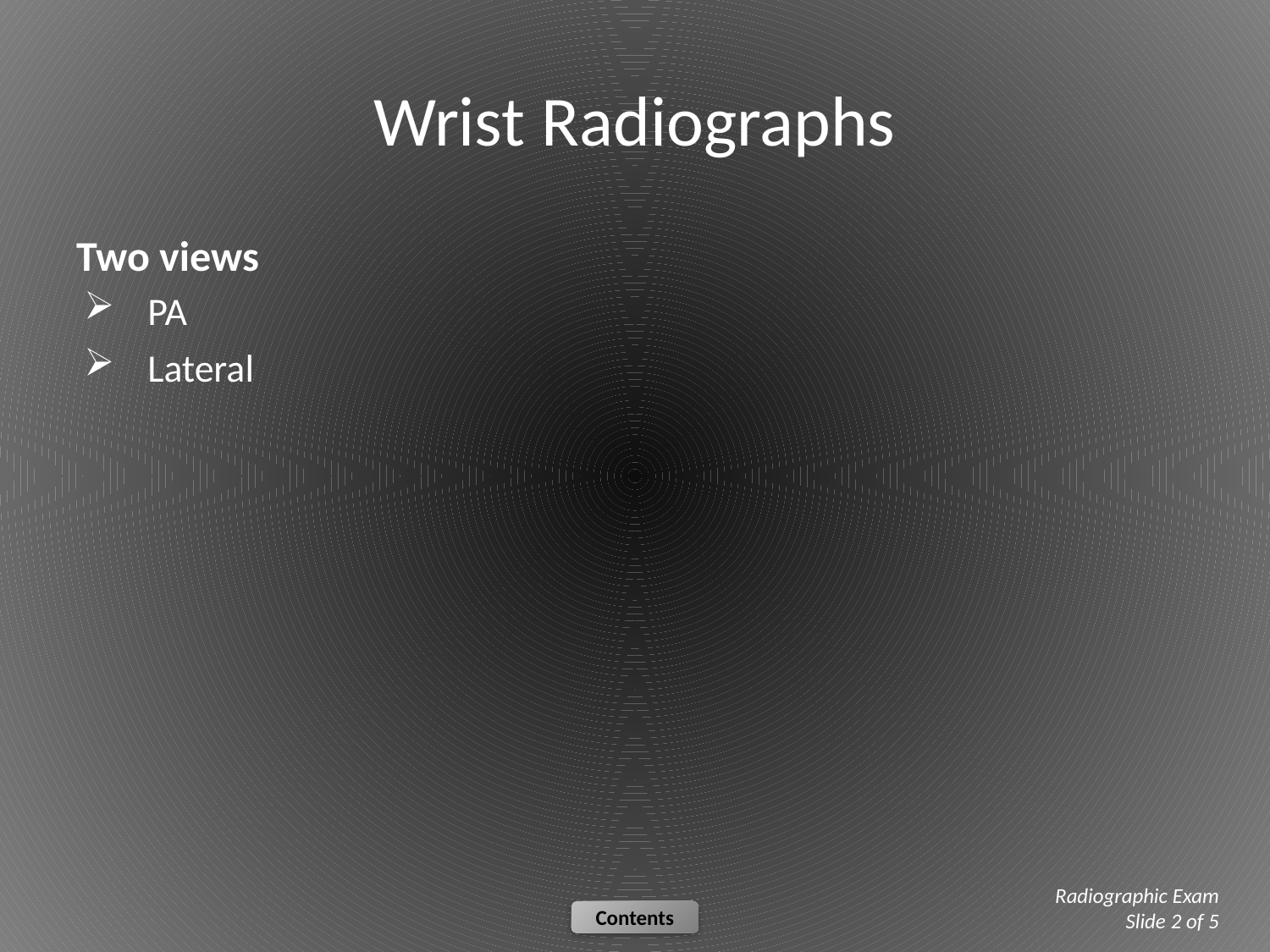

# Wrist Radiographs
Two views
PA
Lateral
Radiographic Exam
Slide 2 of 5
Contents

## Slide 17
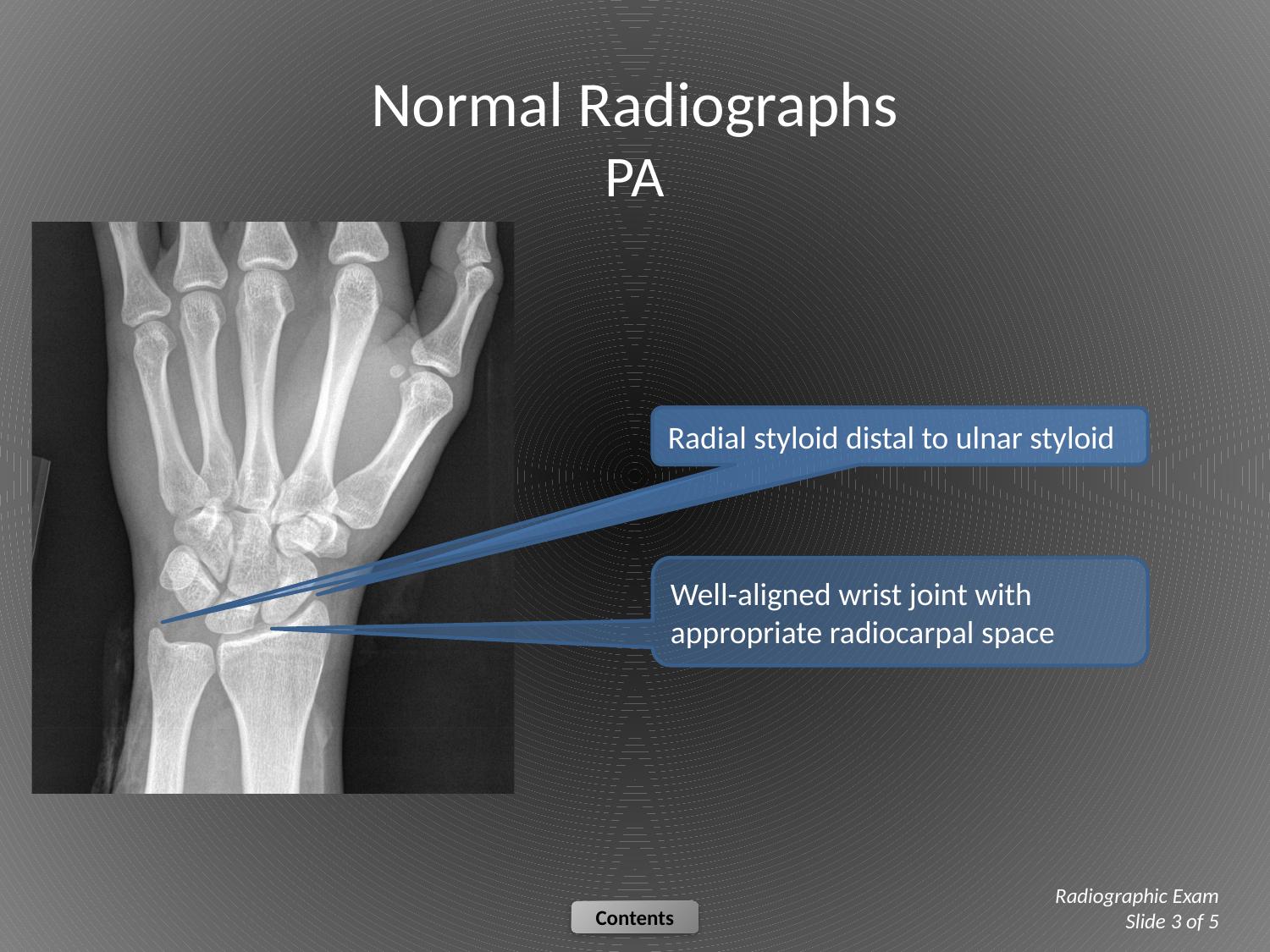

# Normal Radiographs PA
Radial styloid distal to ulnar styloid
Well-aligned wrist joint with appropriate radiocarpal space
Radiographic Exam
Slide 3 of 5
Contents

## Slide 18
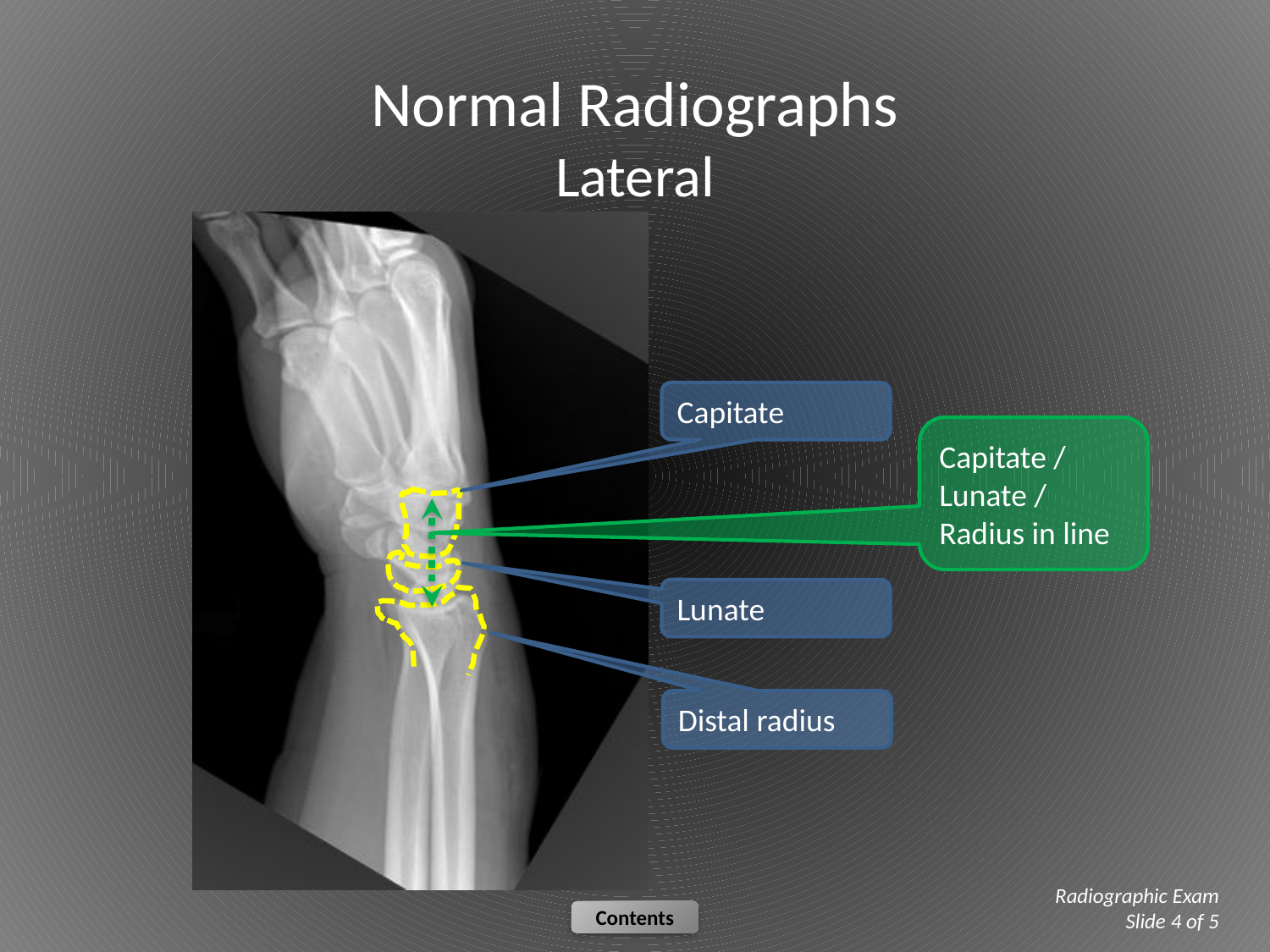

# Normal Radiographs Lateral
Capitate
Capitate / Lunate / Radius in line
Lunate
Distal radius
Radiographic Exam
Slide 4 of 5
Contents

## Slide 19
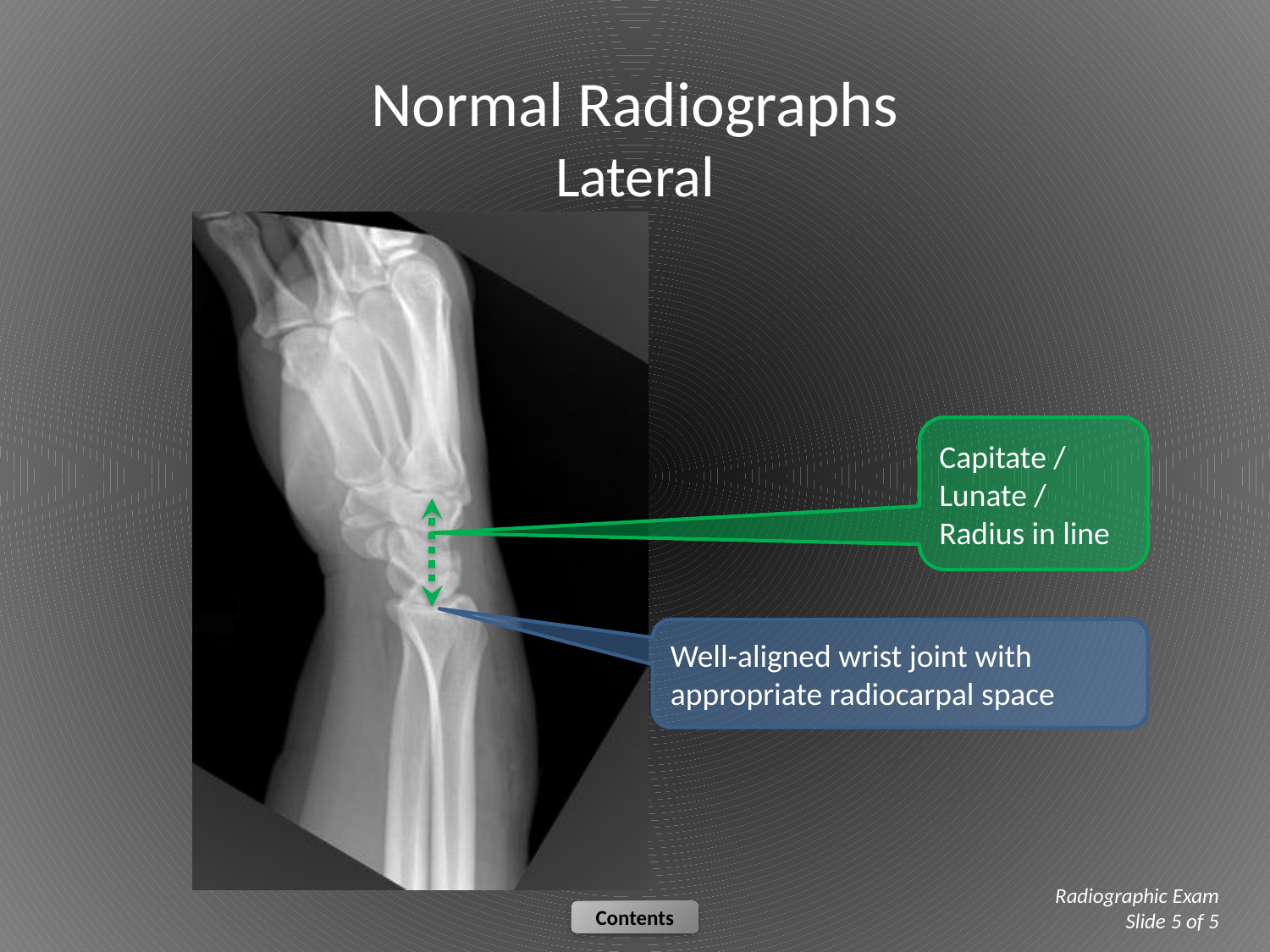

# Normal Radiographs Lateral
Capitate / Lunate / Radius in line
Well-aligned wrist joint with appropriate radiocarpal space
Radiographic Exam
Slide 5 of 5
Contents

## Slide 20
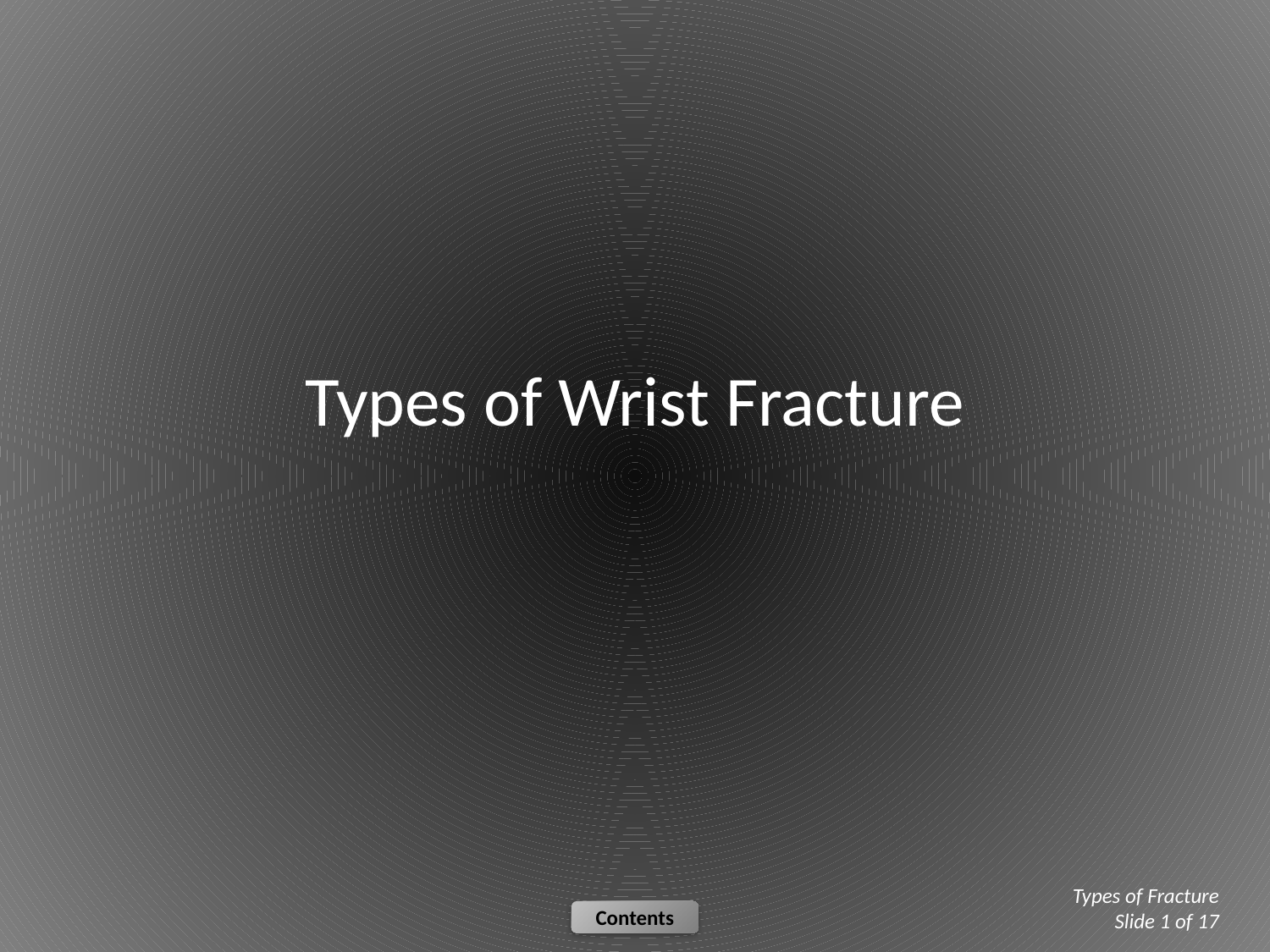

# Types of Wrist Fracture
Types of Fracture
Slide 1 of 17
Contents

## Slide 21
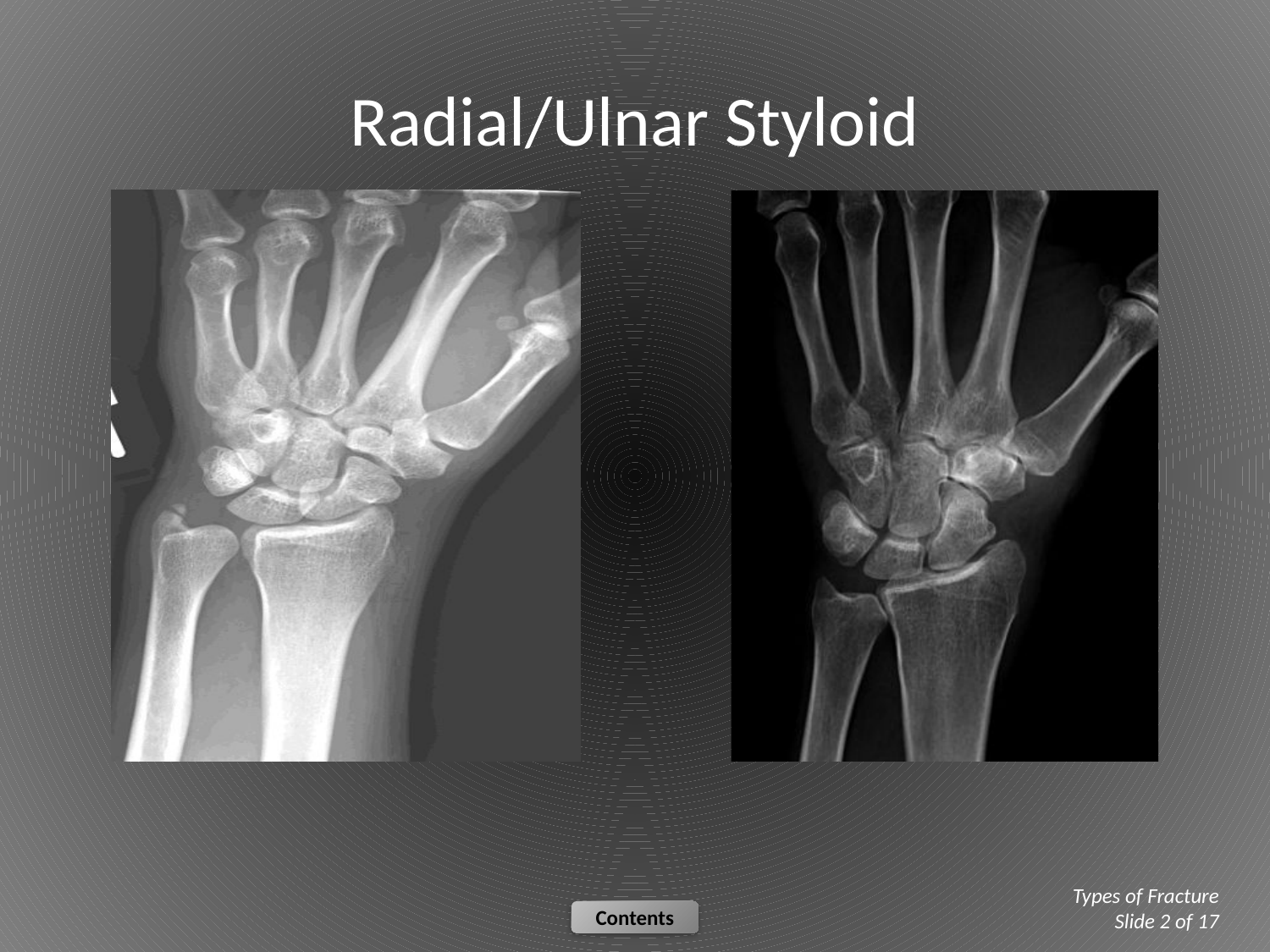

# Radial/Ulnar Styloid
Types of Fracture
Slide 2 of 17
Contents

## Slide 22
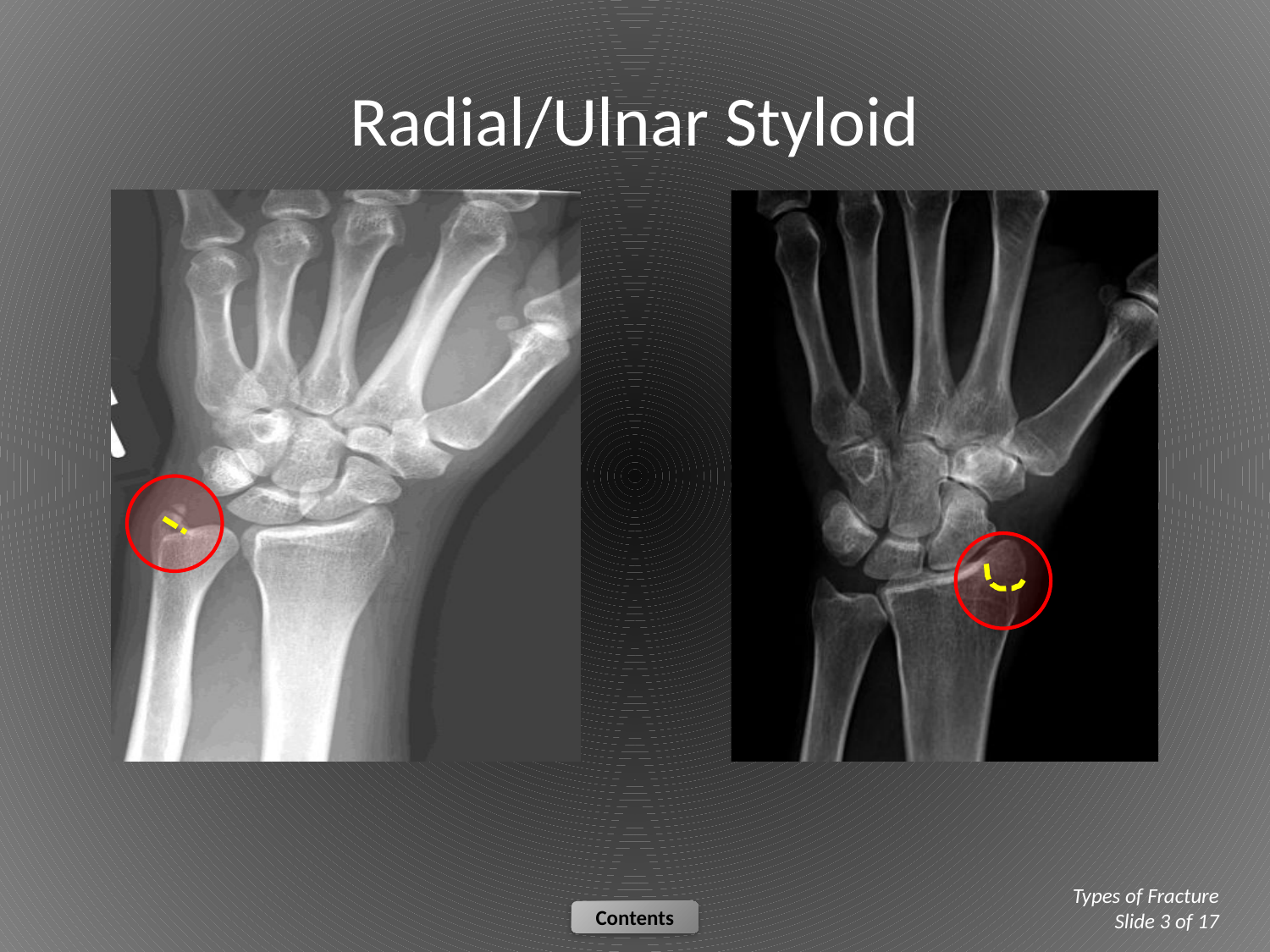

# Radial/Ulnar Styloid
Types of Fracture
Slide 3 of 17
Contents

## Slide 23
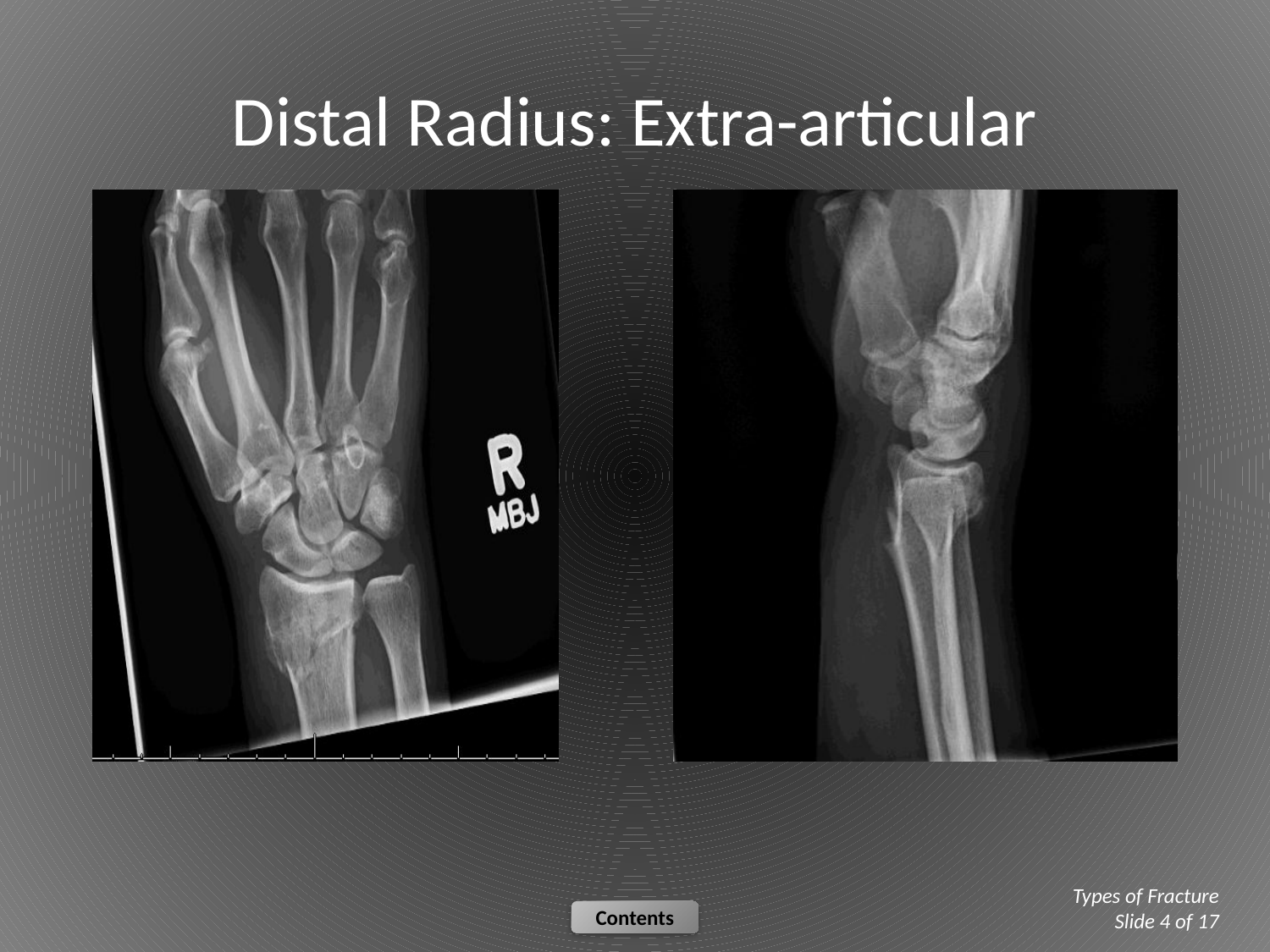

# Distal Radius: Extra-articular
Types of Fracture
Slide 4 of 17
Contents

## Slide 24
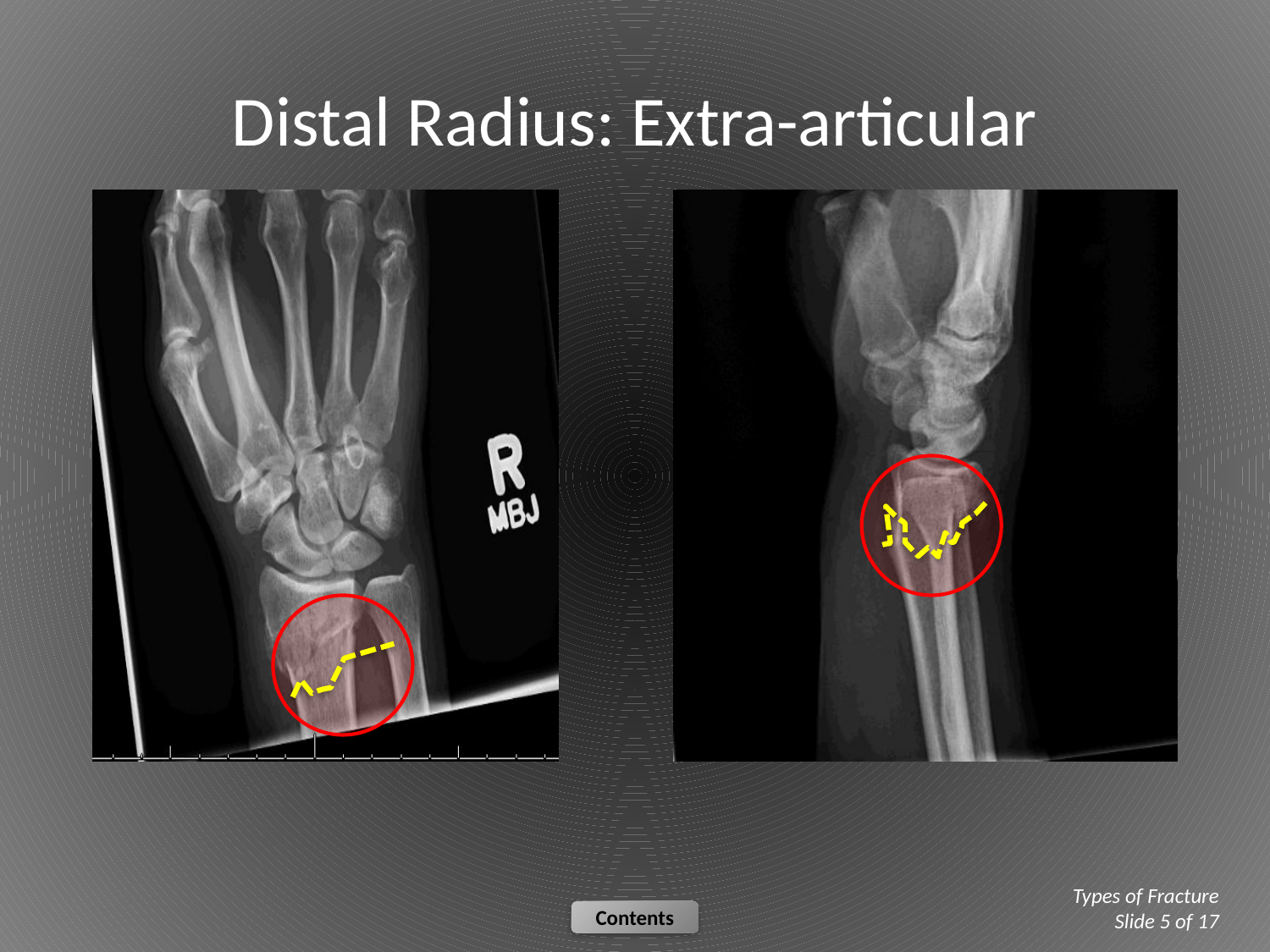

# Distal Radius: Extra-articular
Types of Fracture
Slide 5 of 17
Contents

## Slide 25
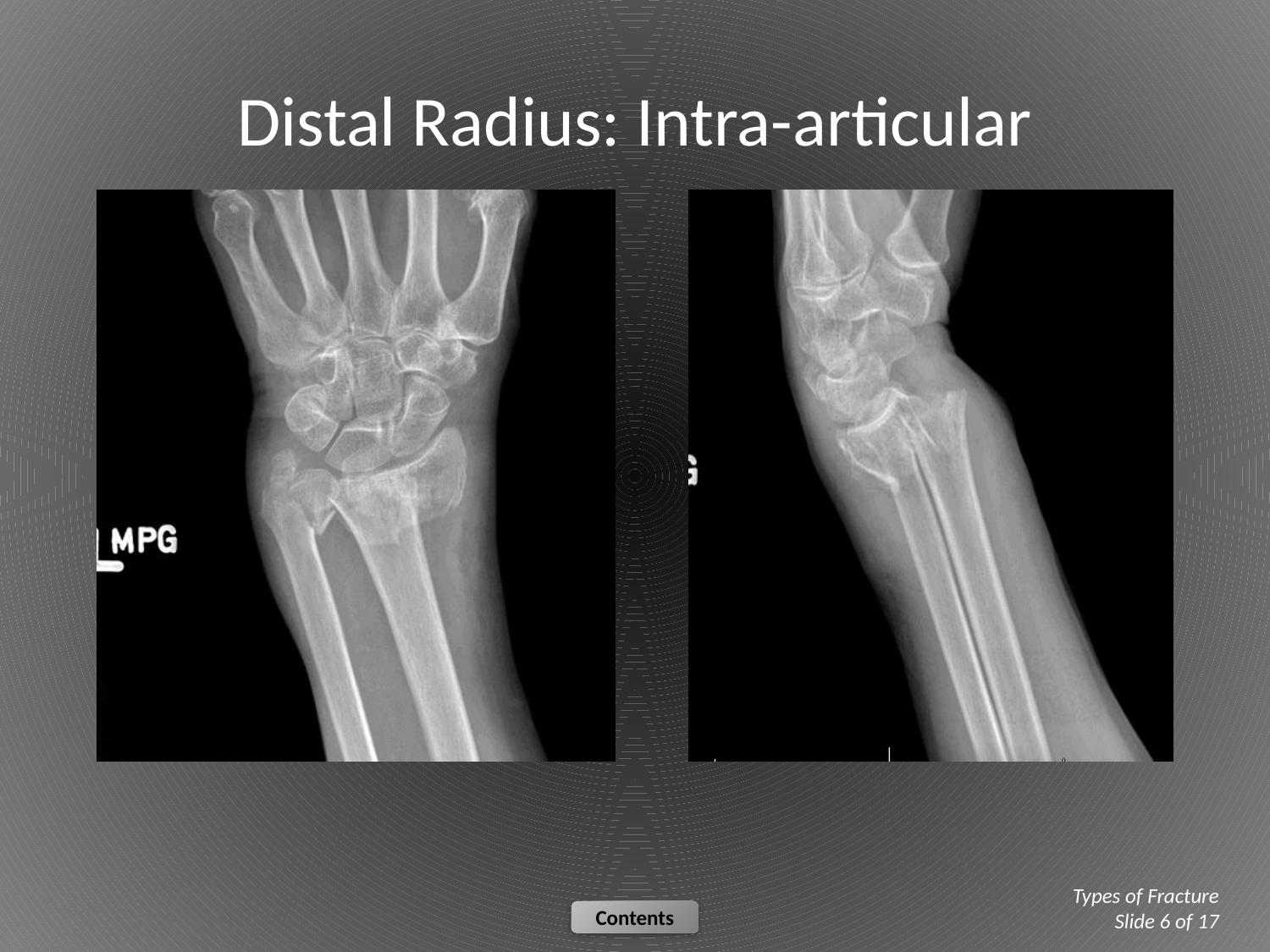

# Distal Radius: Intra-articular
Types of Fracture
Slide 6 of 17
Contents

## Slide 26
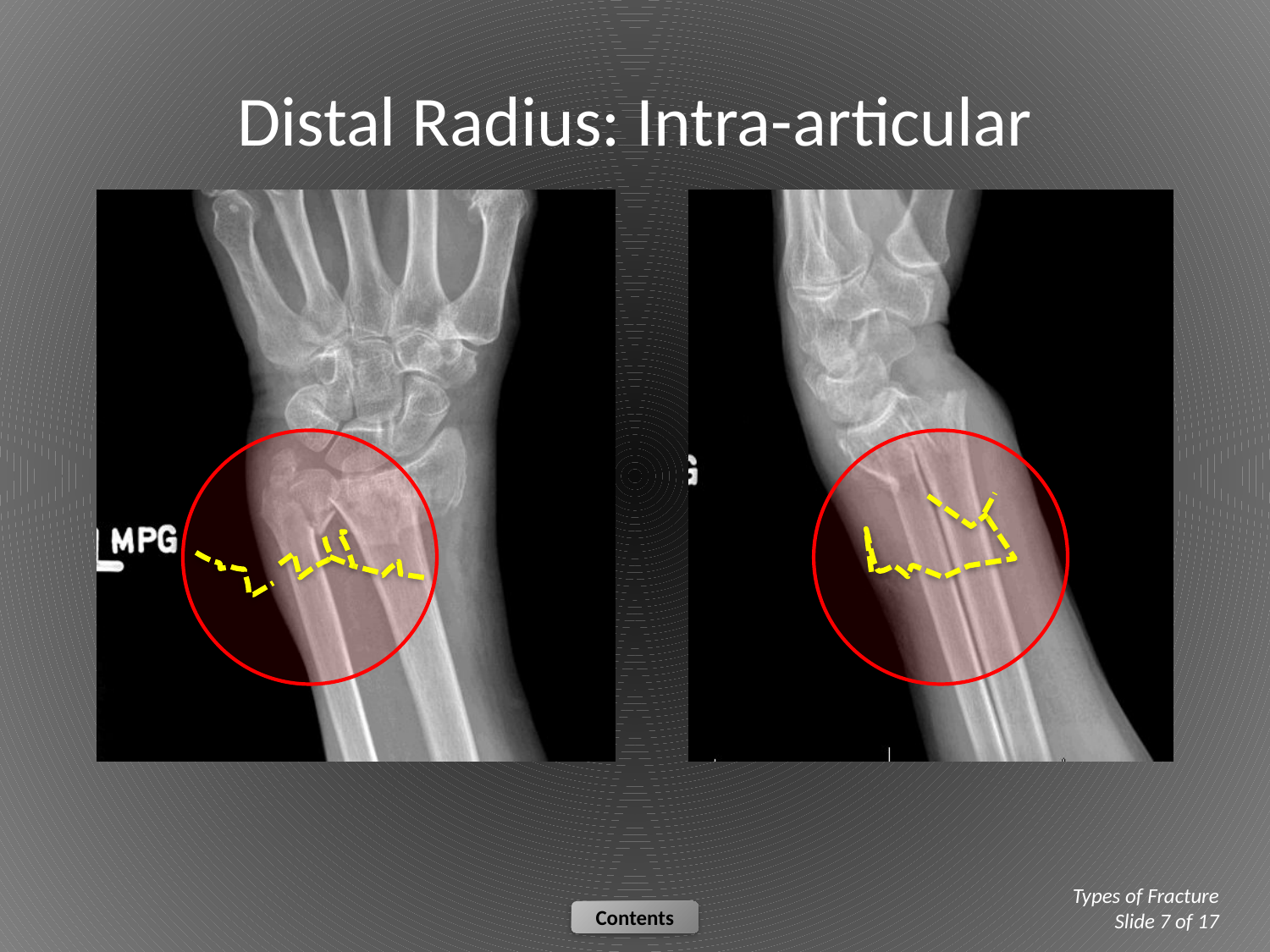

# Distal Radius: Intra-articular
Types of Fracture
Slide 7 of 17
Contents

## Slide 27
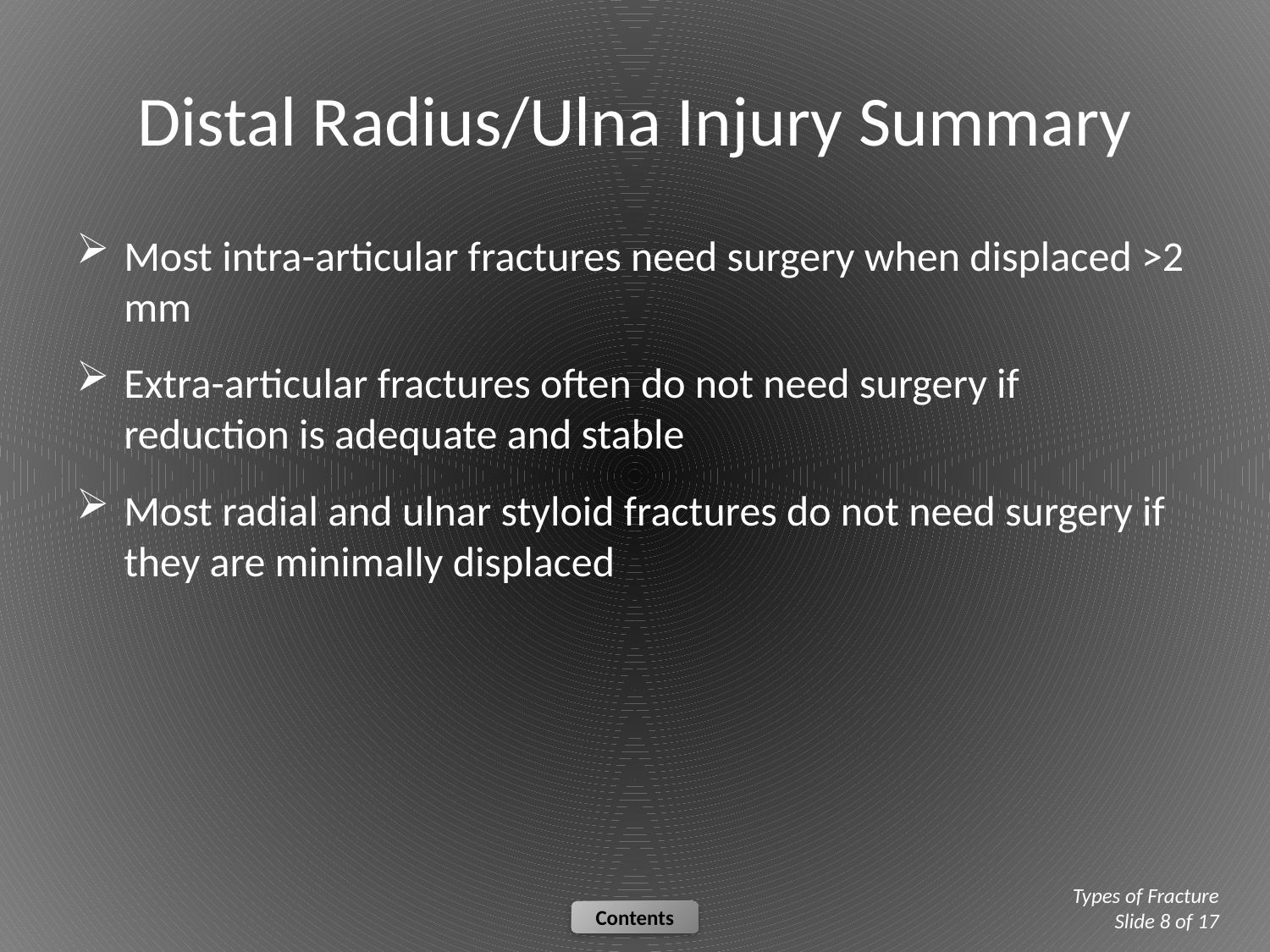

# Distal Radius/Ulna Injury Summary
Most intra-articular fractures need surgery when displaced >2 mm
Extra-articular fractures often do not need surgery if reduction is adequate and stable
Most radial and ulnar styloid fractures do not need surgery if they are minimally displaced
Types of Fracture
Slide 8 of 17
Contents

## Slide 28
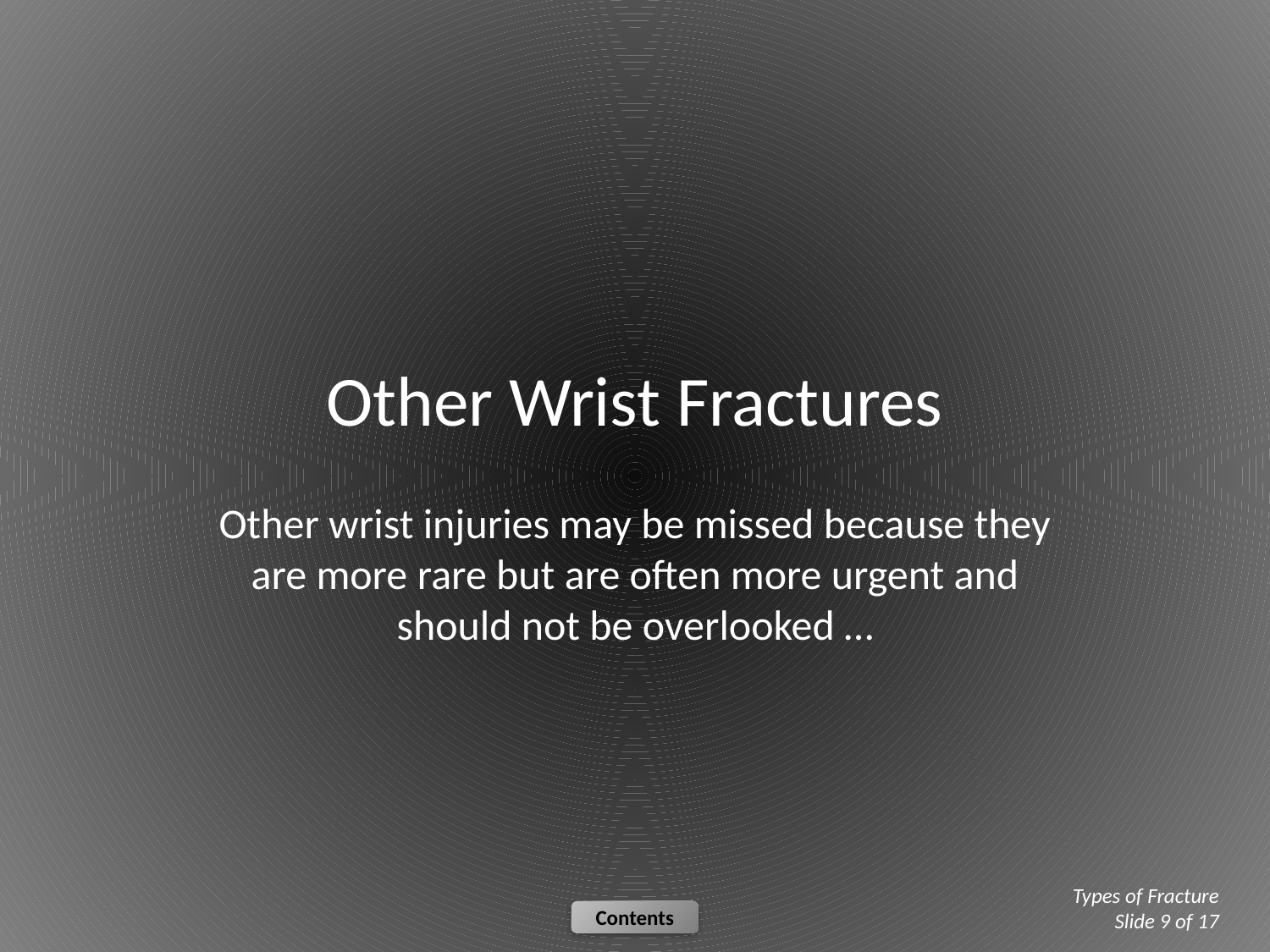

# Other Wrist Fractures
Other wrist injuries may be missed because they are more rare but are often more urgent and should not be overlooked …
Types of Fracture
Slide 9 of 17
Contents

## Slide 29
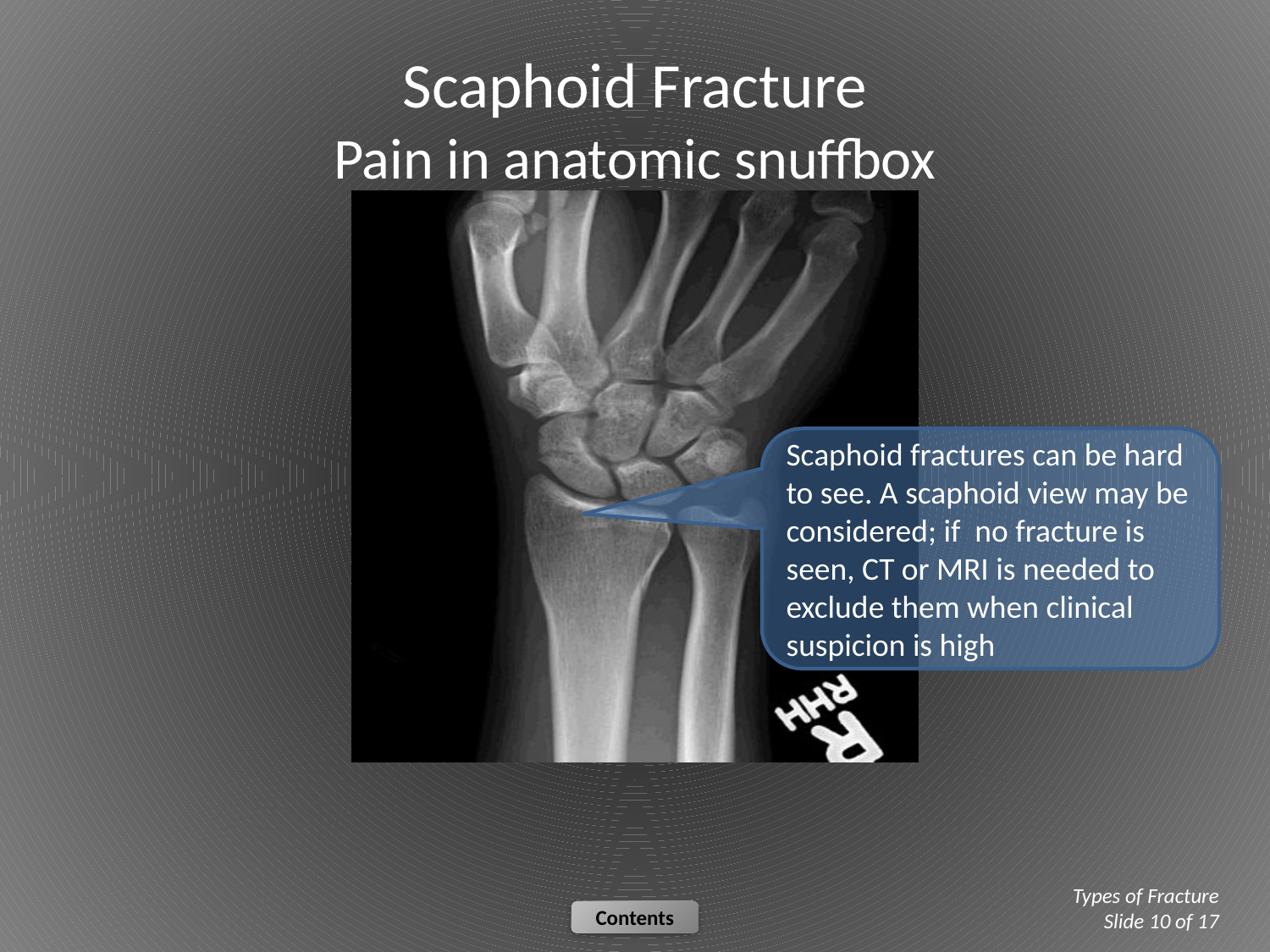

# Scaphoid Fracture Pain in anatomic snuffbox
Scaphoid fractures can be hard to see. A scaphoid view may be considered; if no fracture is seen, CT or MRI is needed to exclude them when clinical suspicion is high
Types of Fracture
Slide 10 of 17
Contents

## Slide 30
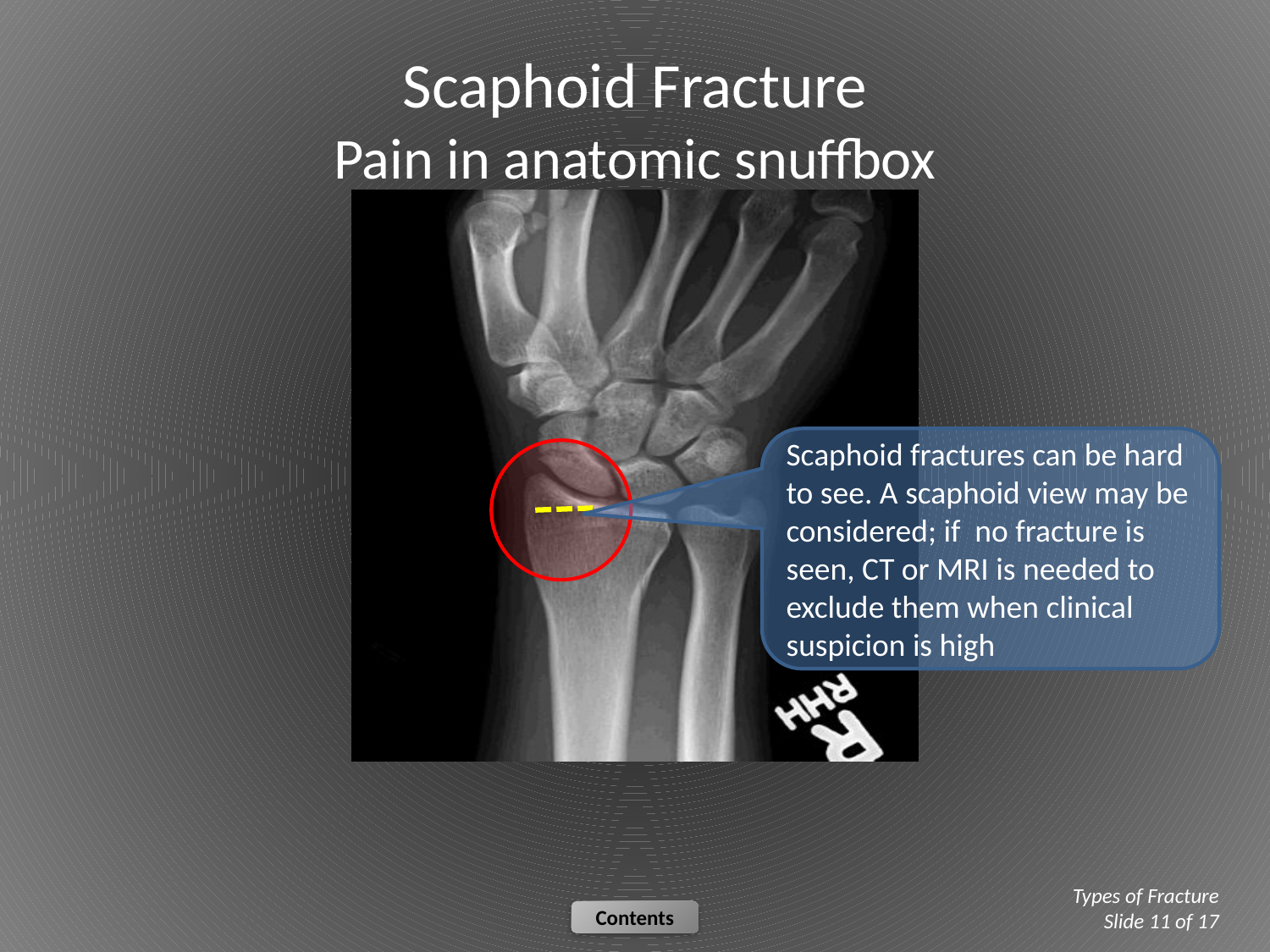

# Scaphoid Fracture Pain in anatomic snuffbox
Scaphoid fractures can be hard to see. A scaphoid view may be considered; if no fracture is seen, CT or MRI is needed to exclude them when clinical suspicion is high
Types of Fracture
Slide 11 of 17
Contents

## Slide 31
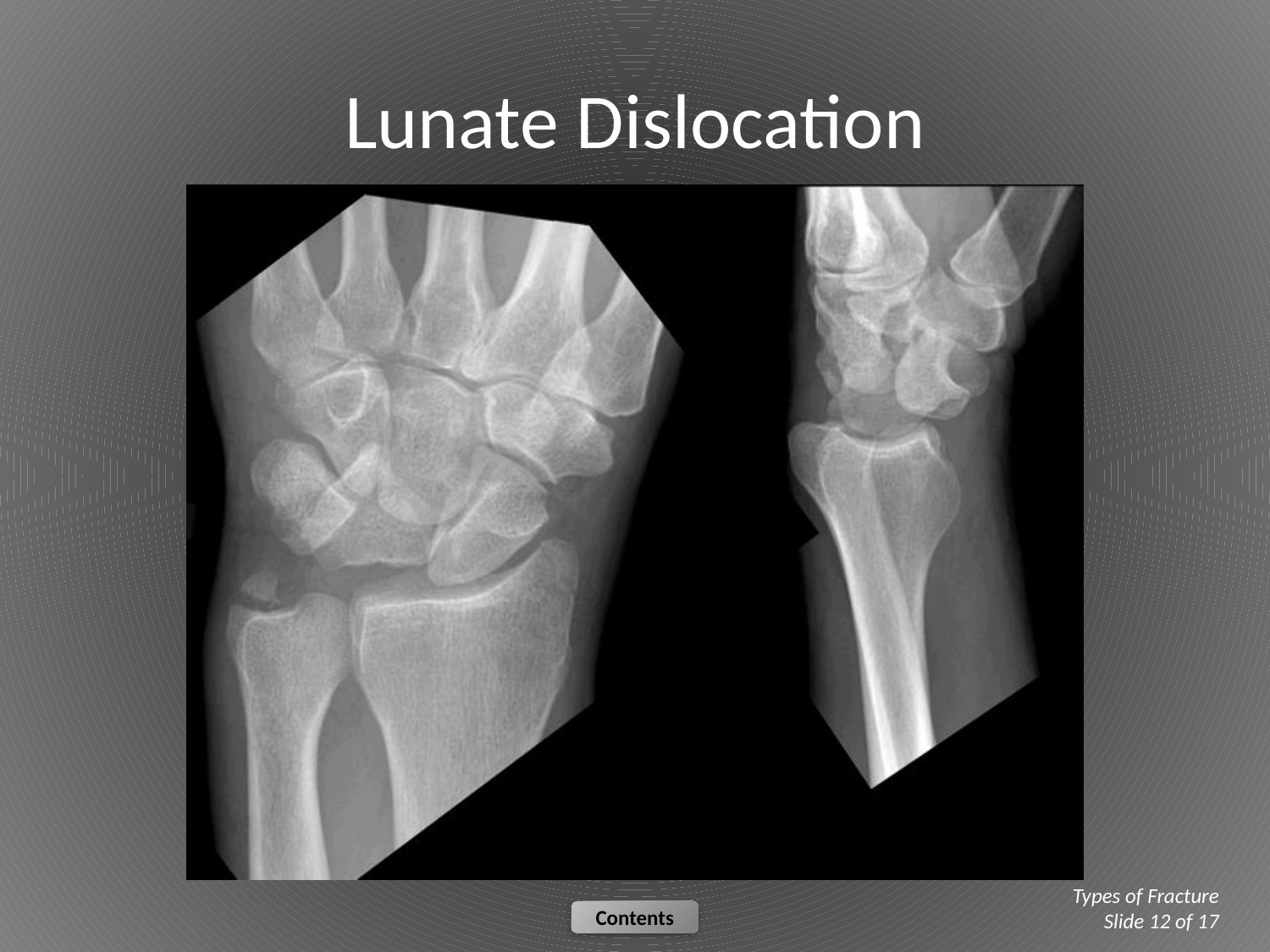

# Lunate Dislocation
Types of Fracture
Slide 12 of 17
Contents

## Slide 32
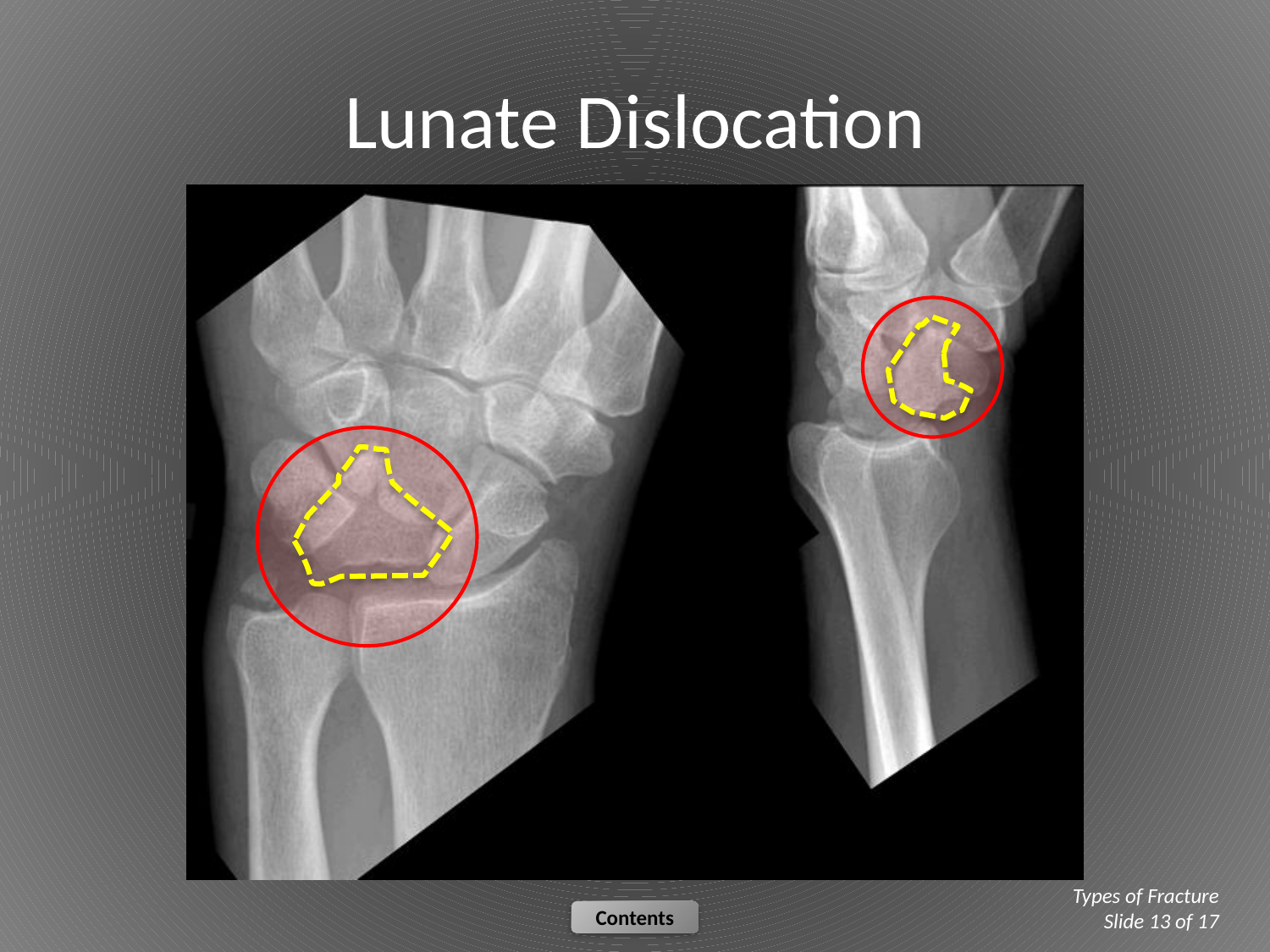

# Lunate Dislocation
Types of Fracture
Slide 13 of 17
Contents

## Slide 33
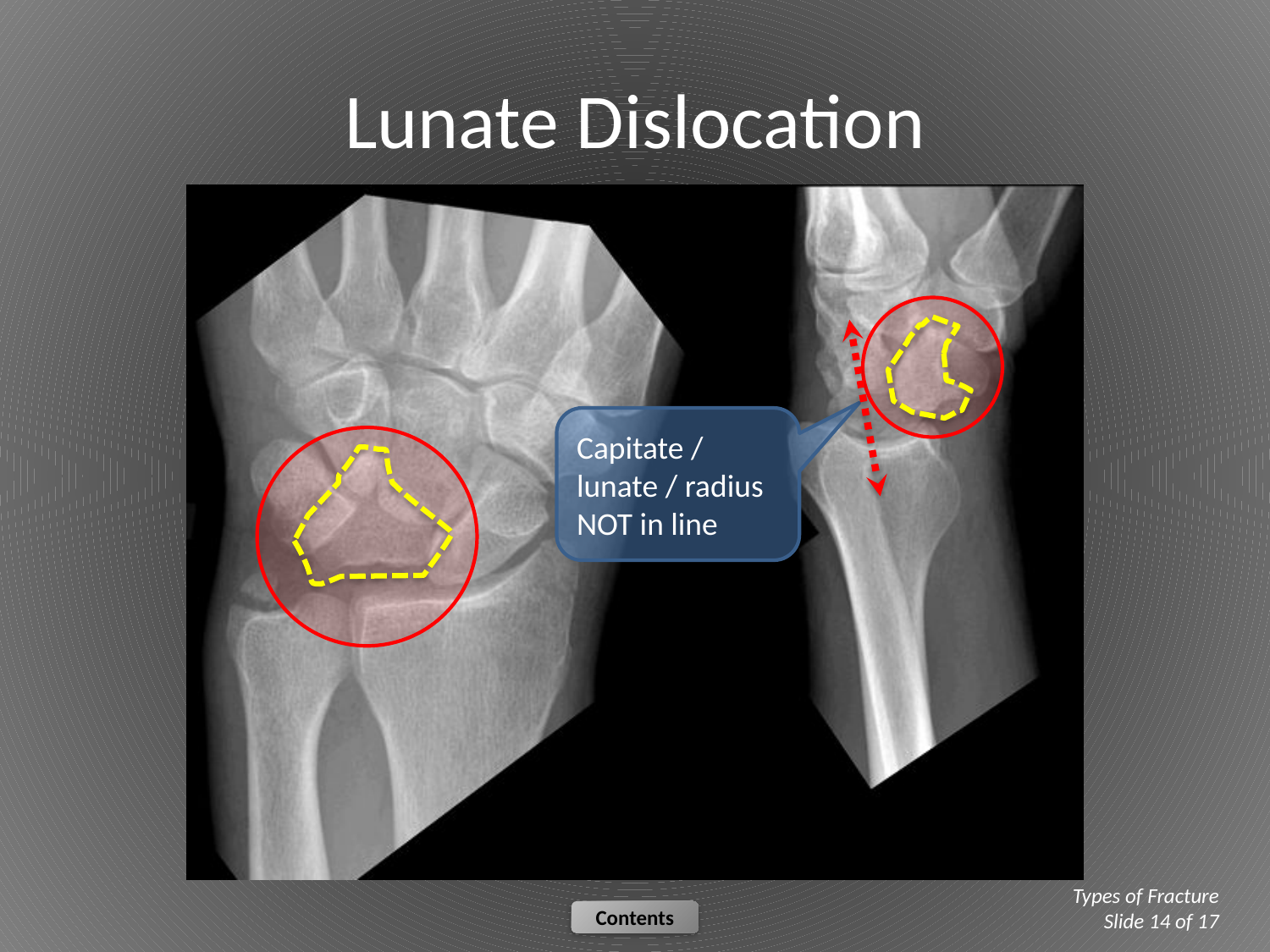

# Lunate Dislocation
Capitate / lunate / radius NOT in line
Types of Fracture
Slide 14 of 17
Contents

## Slide 34
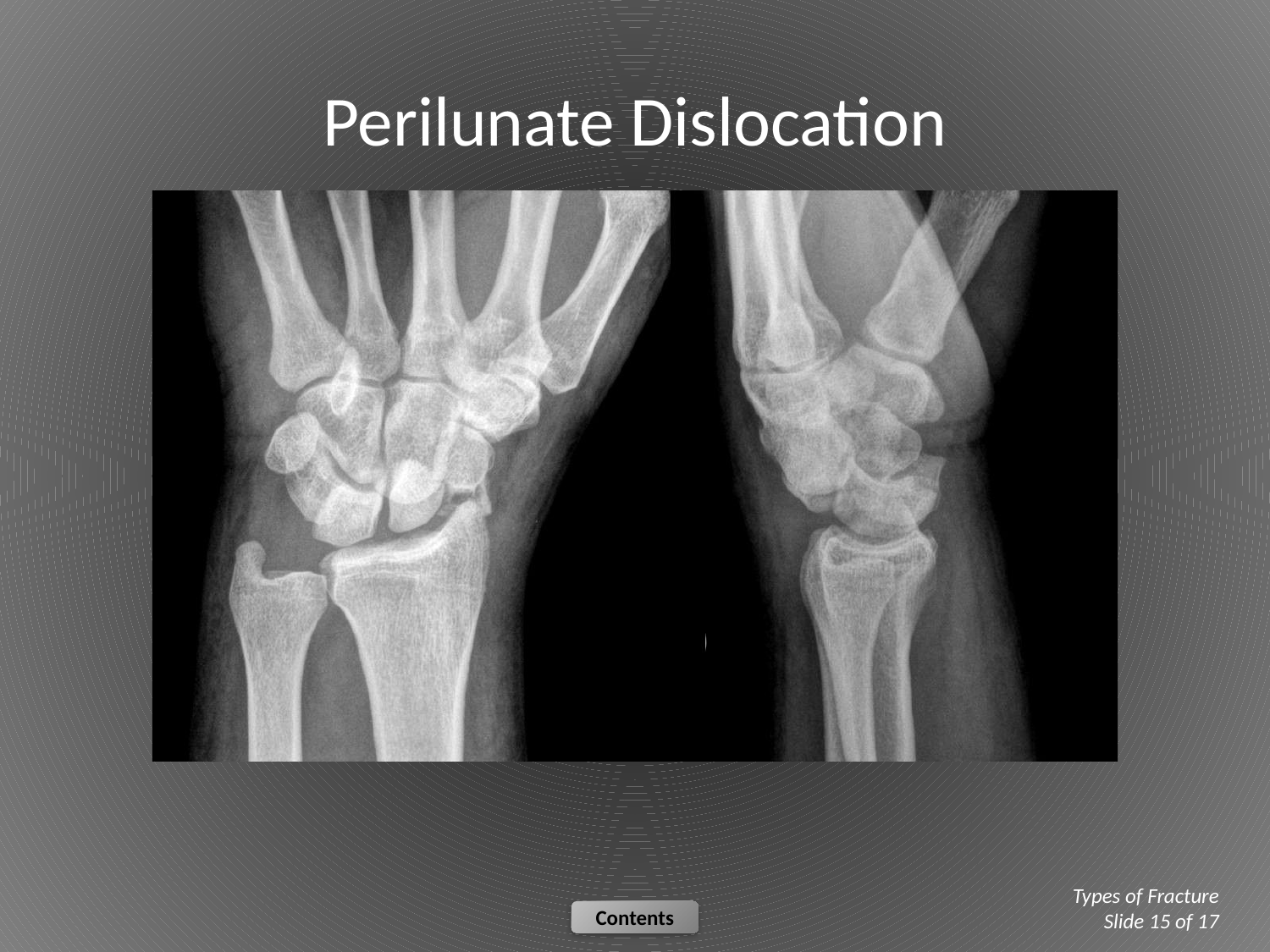

# Perilunate Dislocation
Types of Fracture
Slide 15 of 17
Contents

## Slide 35
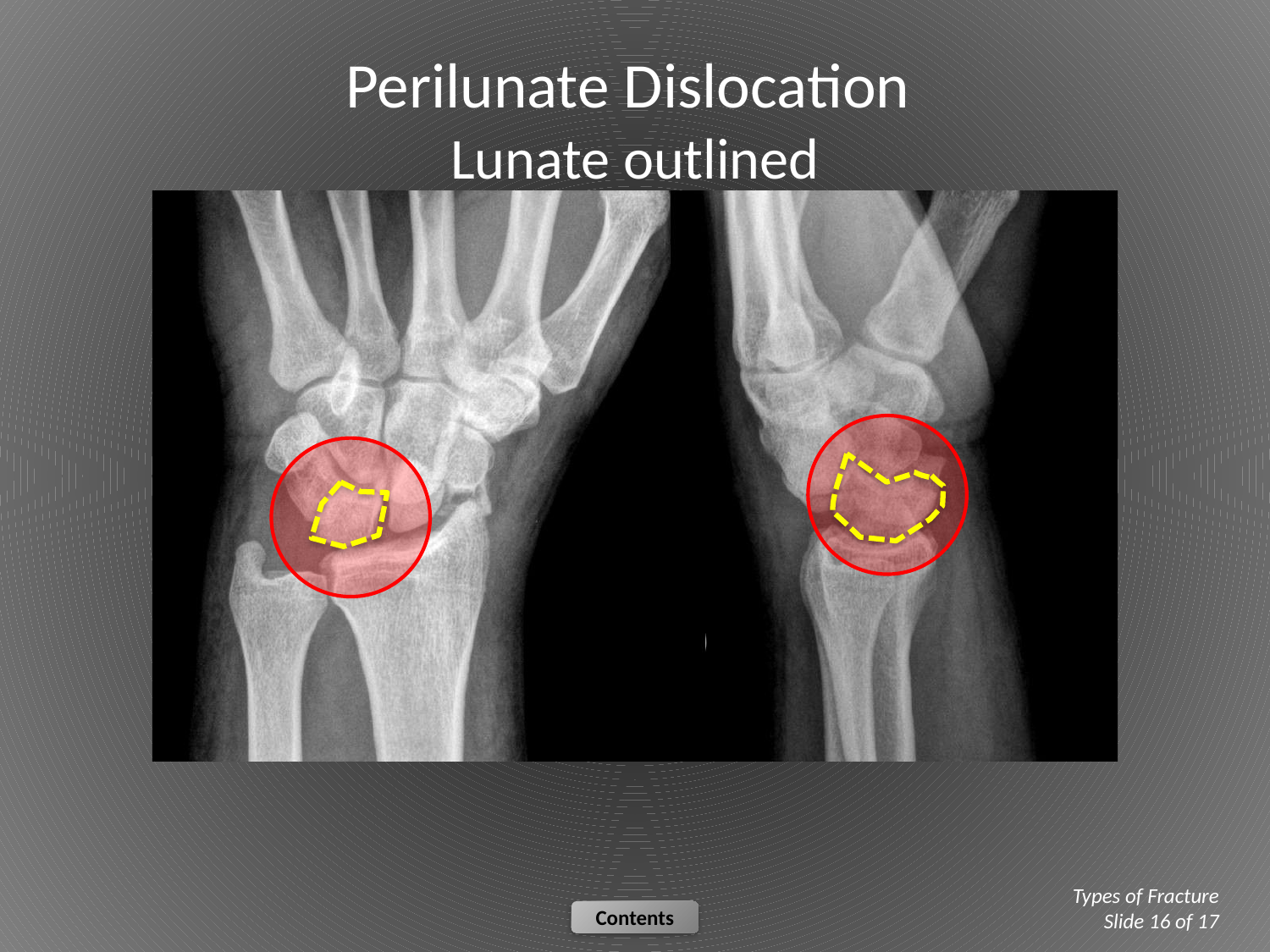

# Perilunate Dislocation Lunate outlined
Types of Fracture
Slide 16 of 17
Contents

## Slide 36
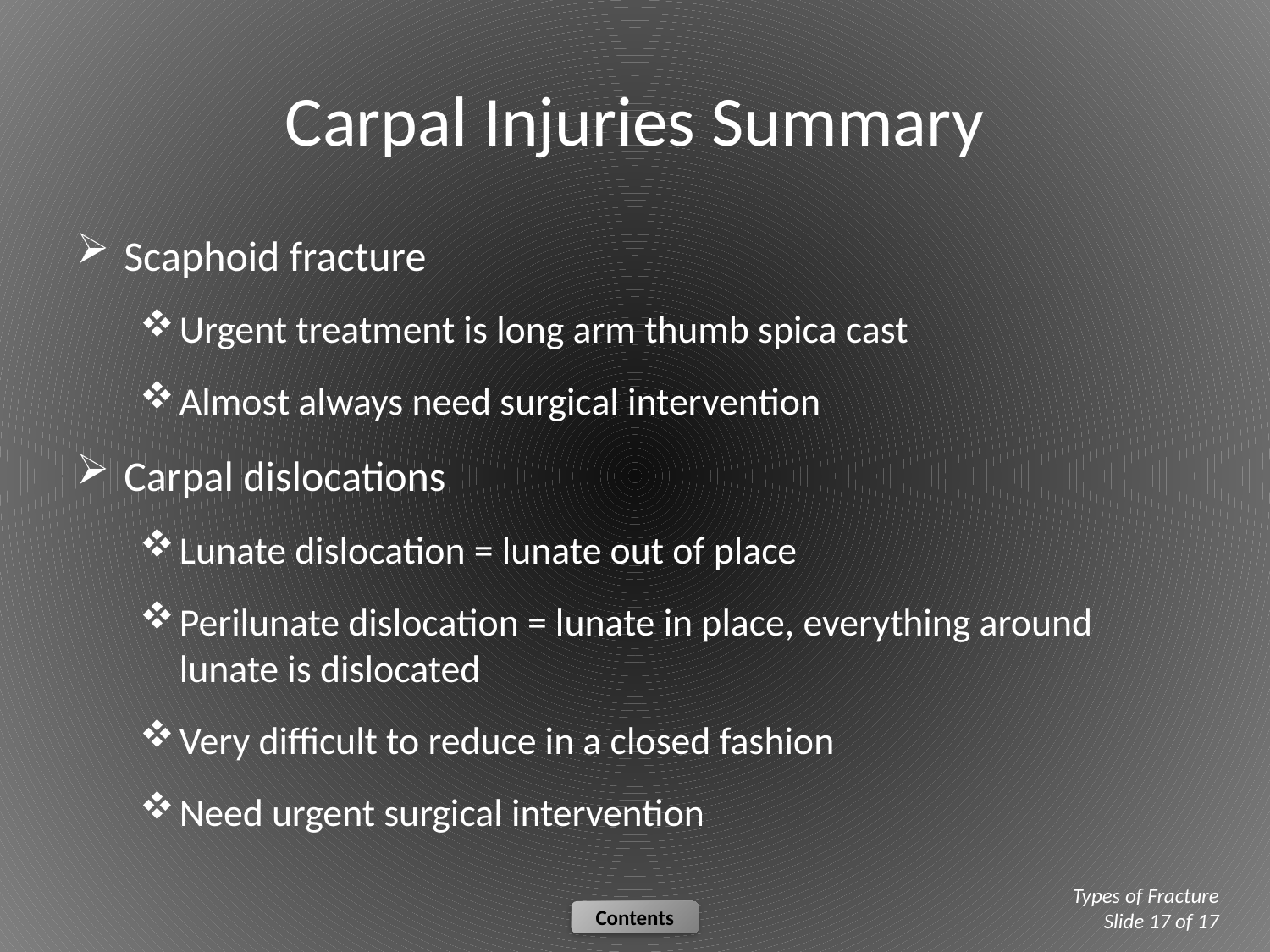

# Carpal Injuries Summary
Scaphoid fracture
Urgent treatment is long arm thumb spica cast
Almost always need surgical intervention
Carpal dislocations
Lunate dislocation = lunate out of place
Perilunate dislocation = lunate in place, everything around lunate is dislocated
Very difficult to reduce in a closed fashion
Need urgent surgical intervention
Types of Fracture
Slide 17 of 17
Contents

## Slide 37
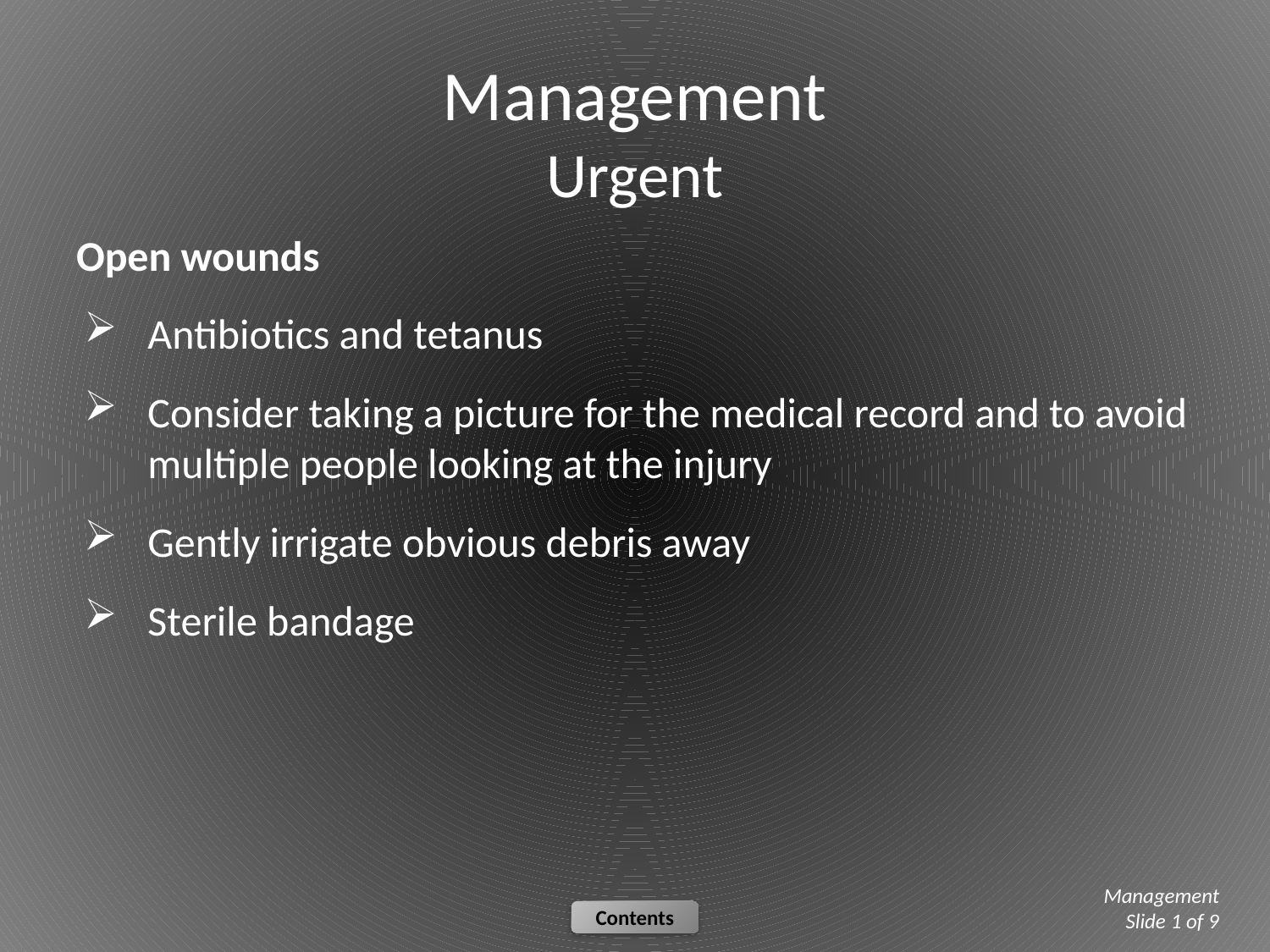

# Management Urgent
Open wounds
Antibiotics and tetanus
Consider taking a picture for the medical record and to avoid multiple people looking at the injury
Gently irrigate obvious debris away
Sterile bandage
Management
Slide 1 of 9
Contents

## Slide 38
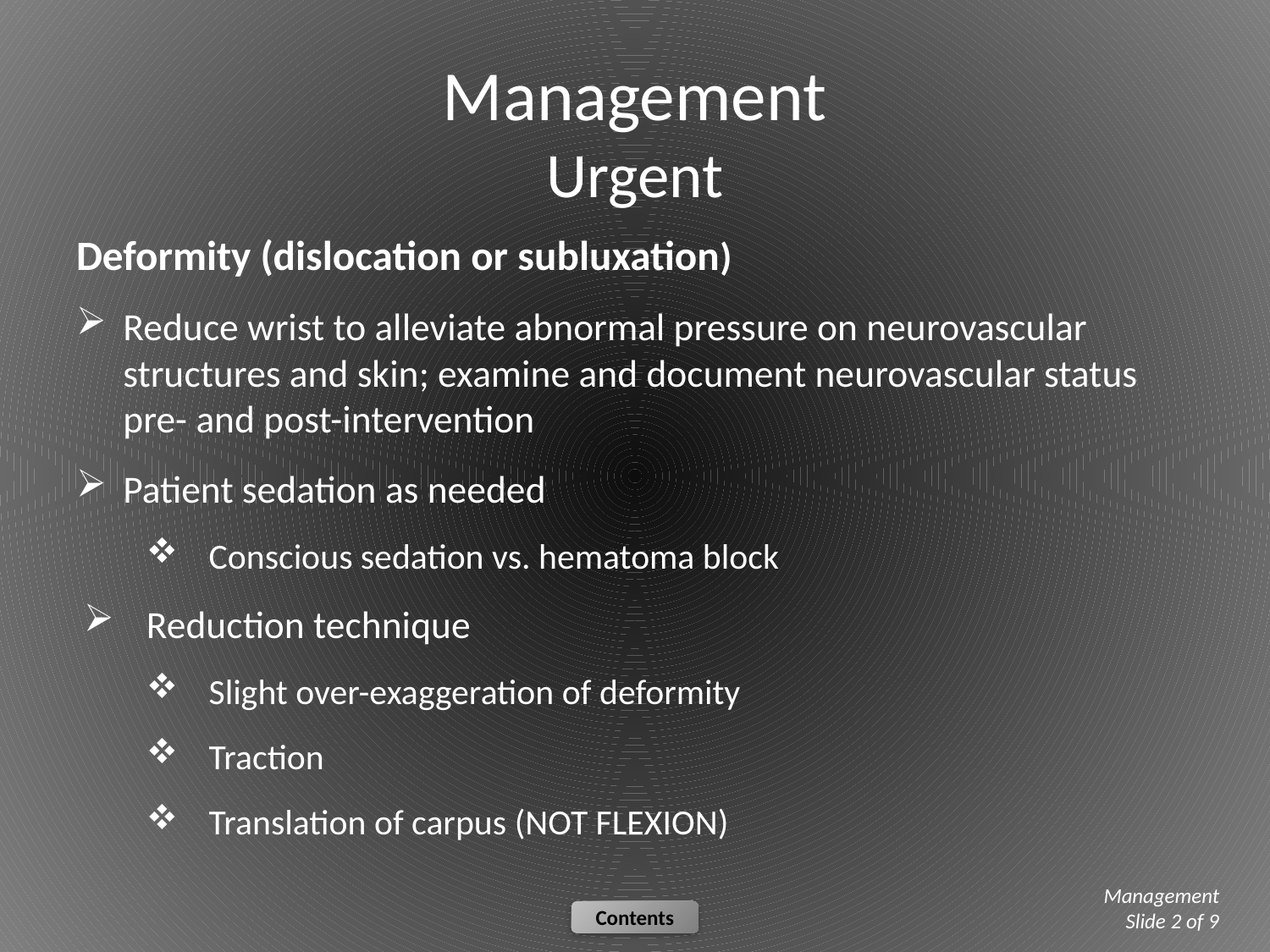

# Management Urgent
Deformity (dislocation or subluxation)
Reduce wrist to alleviate abnormal pressure on neurovascular structures and skin; examine and document neurovascular status pre- and post-intervention
Patient sedation as needed
Conscious sedation vs. hematoma block
Reduction technique
Slight over-exaggeration of deformity
Traction
Translation of carpus (NOT FLEXION)
Management
Slide 2 of 9
Contents

## Slide 39
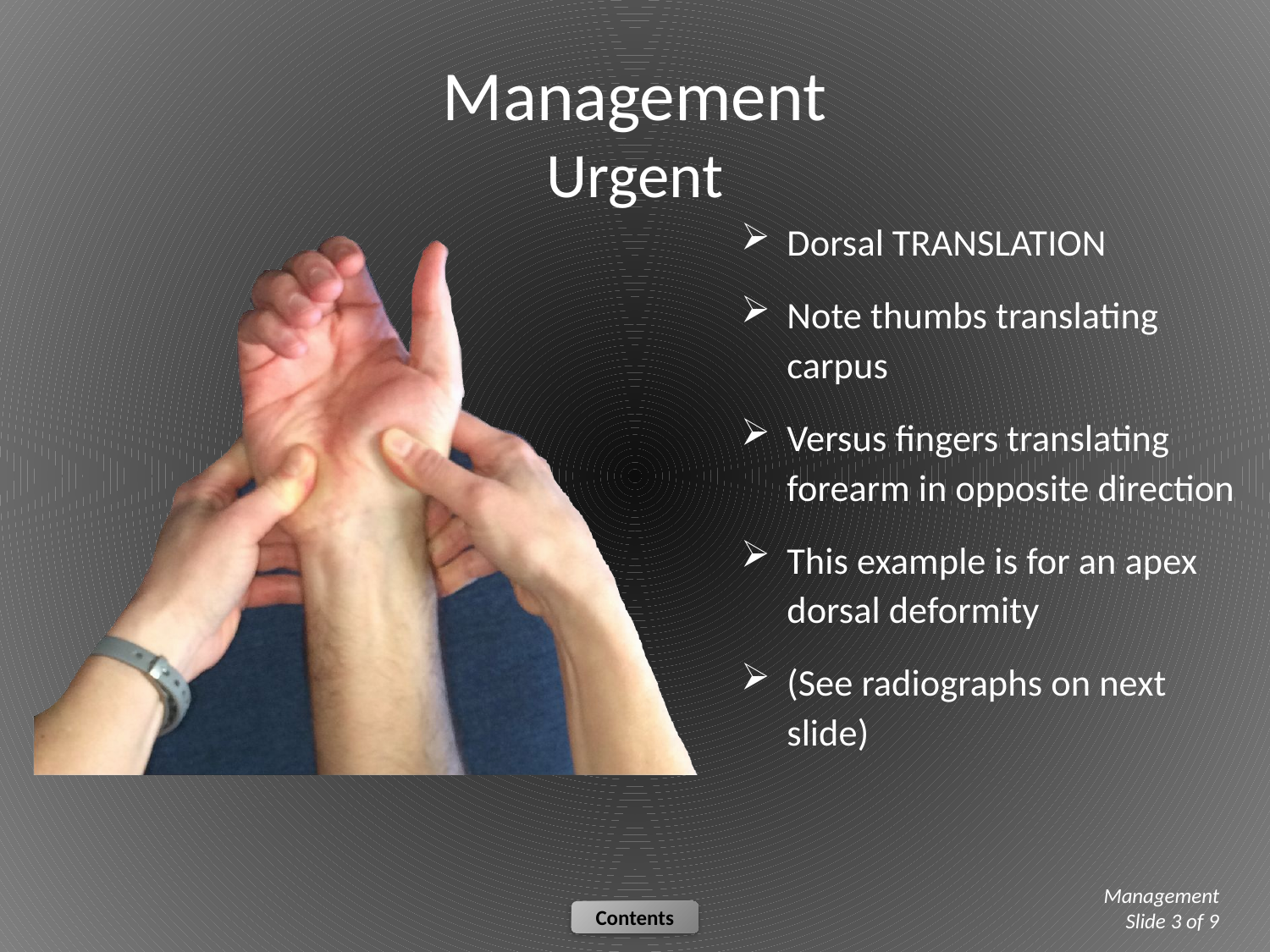

# Management Urgent
Dorsal TRANSLATION
Note thumbs translating carpus
Versus fingers translating forearm in opposite direction
This example is for an apex dorsal deformity
(See radiographs on next slide)
Management
Slide 3 of 9
Contents

## Slide 40
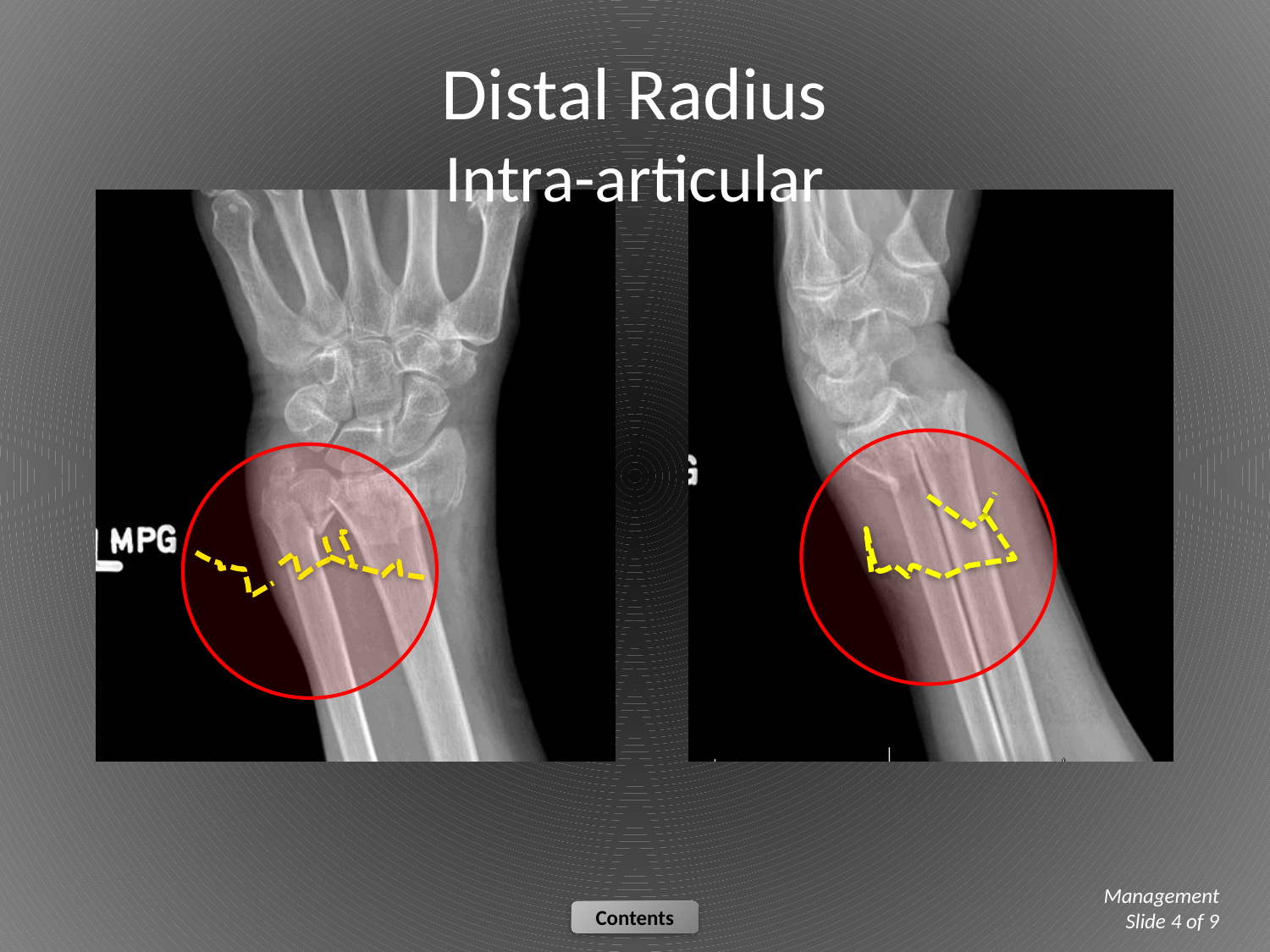

# Distal Radius Intra-articular
Management
Slide 4 of 9
Contents

## Slide 41
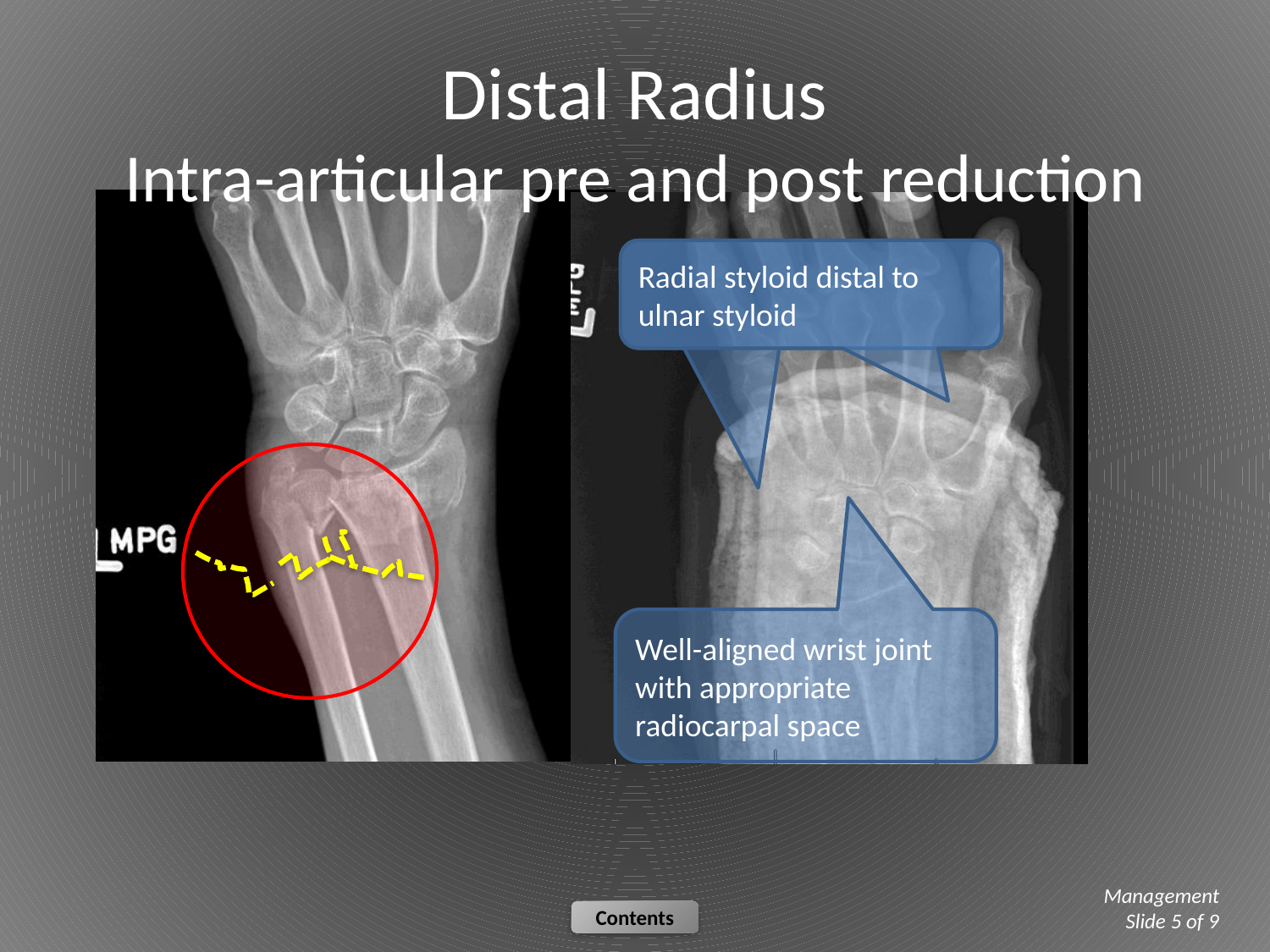

# Distal Radius Intra-articular pre and post reduction
Radial styloid distal to ulnar styloid
Well-aligned wrist joint with appropriate radiocarpal space
Management
Slide 5 of 9
Contents

## Slide 42
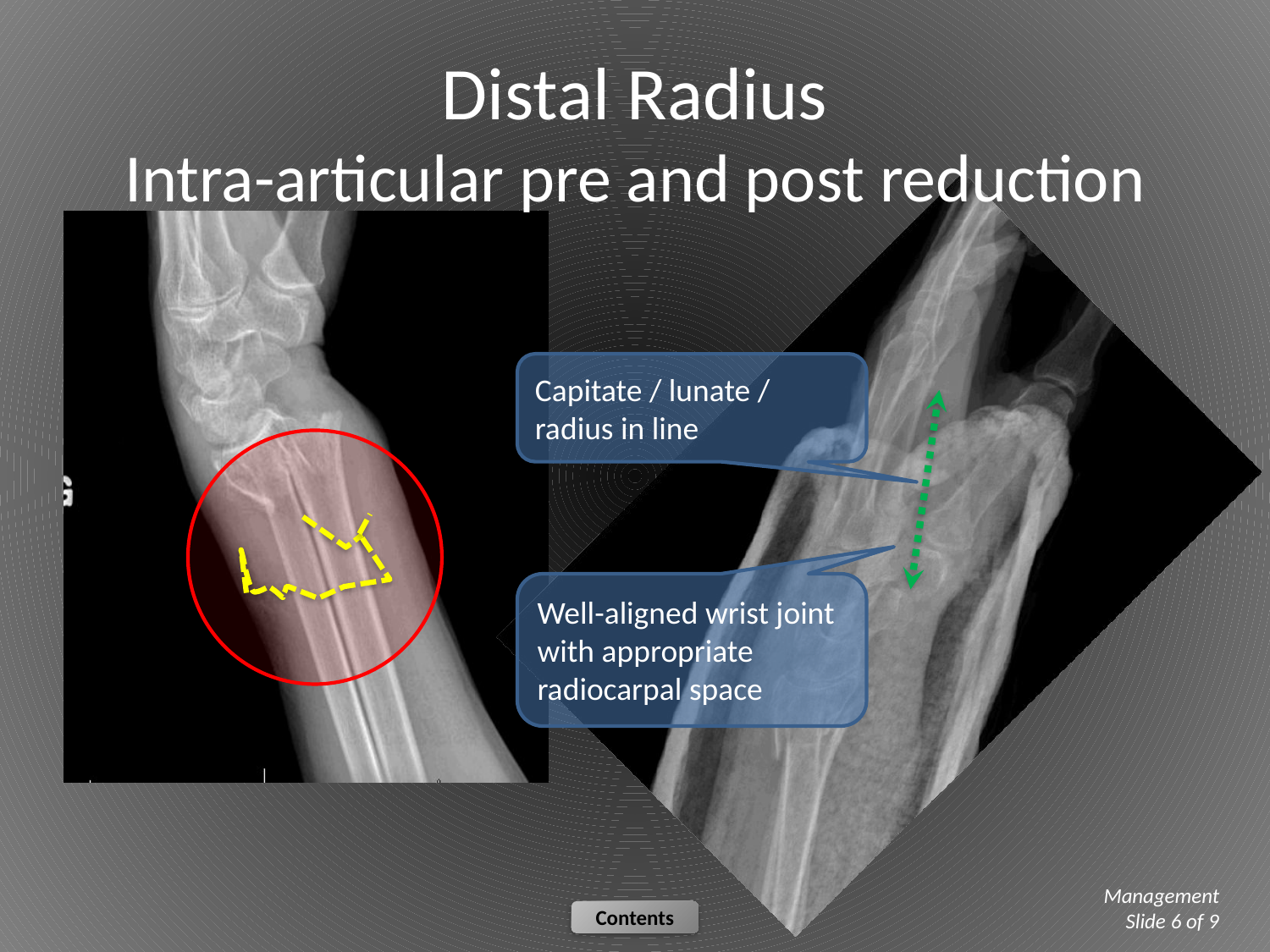

# Distal Radius Intra-articular pre and post reduction
Capitate / lunate / radius in line
Well-aligned wrist joint with appropriate radiocarpal space
Management
Slide 6 of 9
Contents

## Slide 43
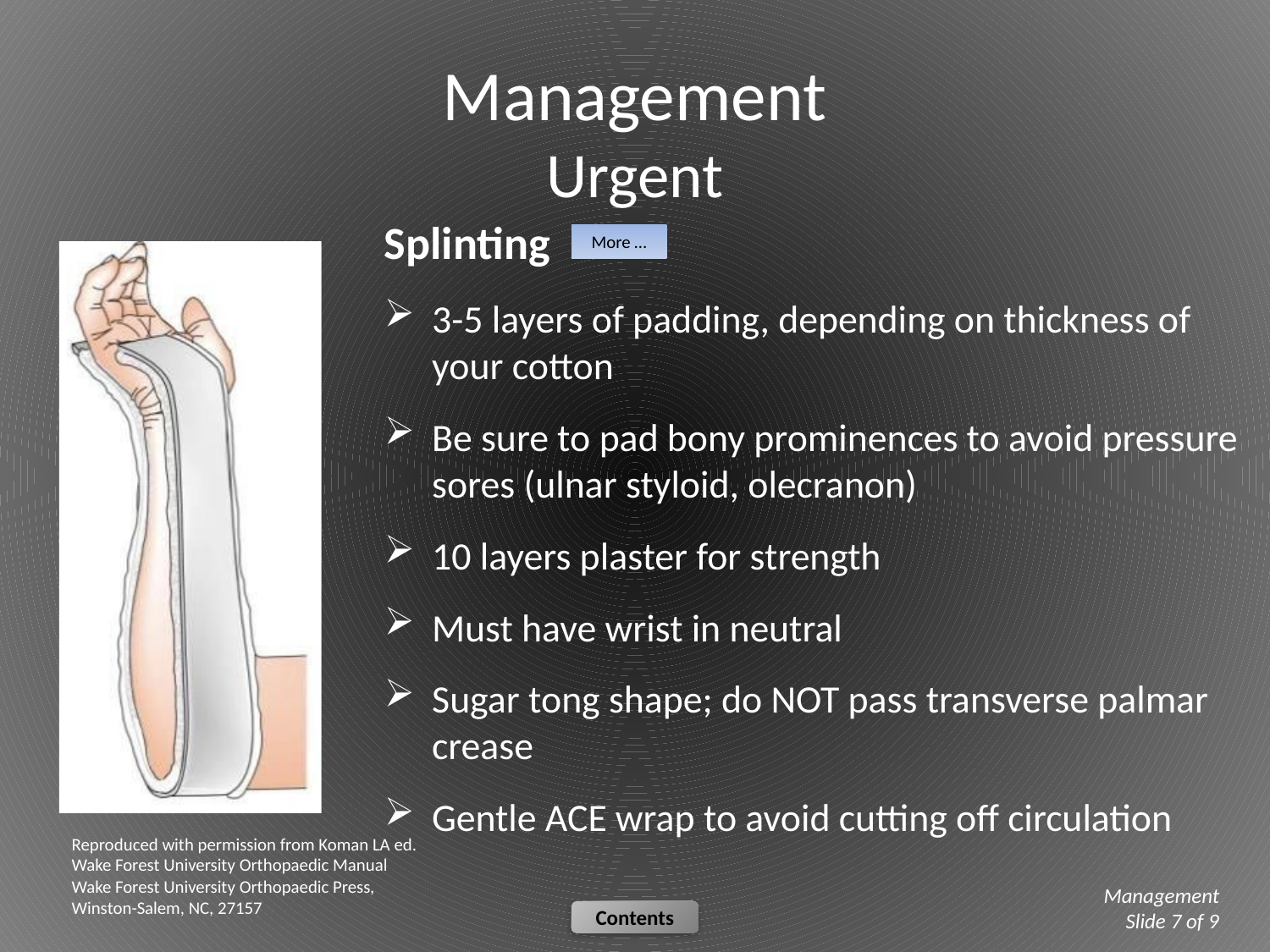

# Management Urgent
Splinting
3-5 layers of padding, depending on thickness of your cotton
Be sure to pad bony prominences to avoid pressure sores (ulnar styloid, olecranon)
10 layers plaster for strength
Must have wrist in neutral
Sugar tong shape; do NOT pass transverse palmar crease
Gentle ACE wrap to avoid cutting off circulation
More …
Reproduced with permission from Koman LA ed.
Wake Forest University Orthopaedic Manual
Wake Forest University Orthopaedic Press,
Winston-Salem, NC, 27157
Management
Slide 7 of 9
Contents

## Slide 44
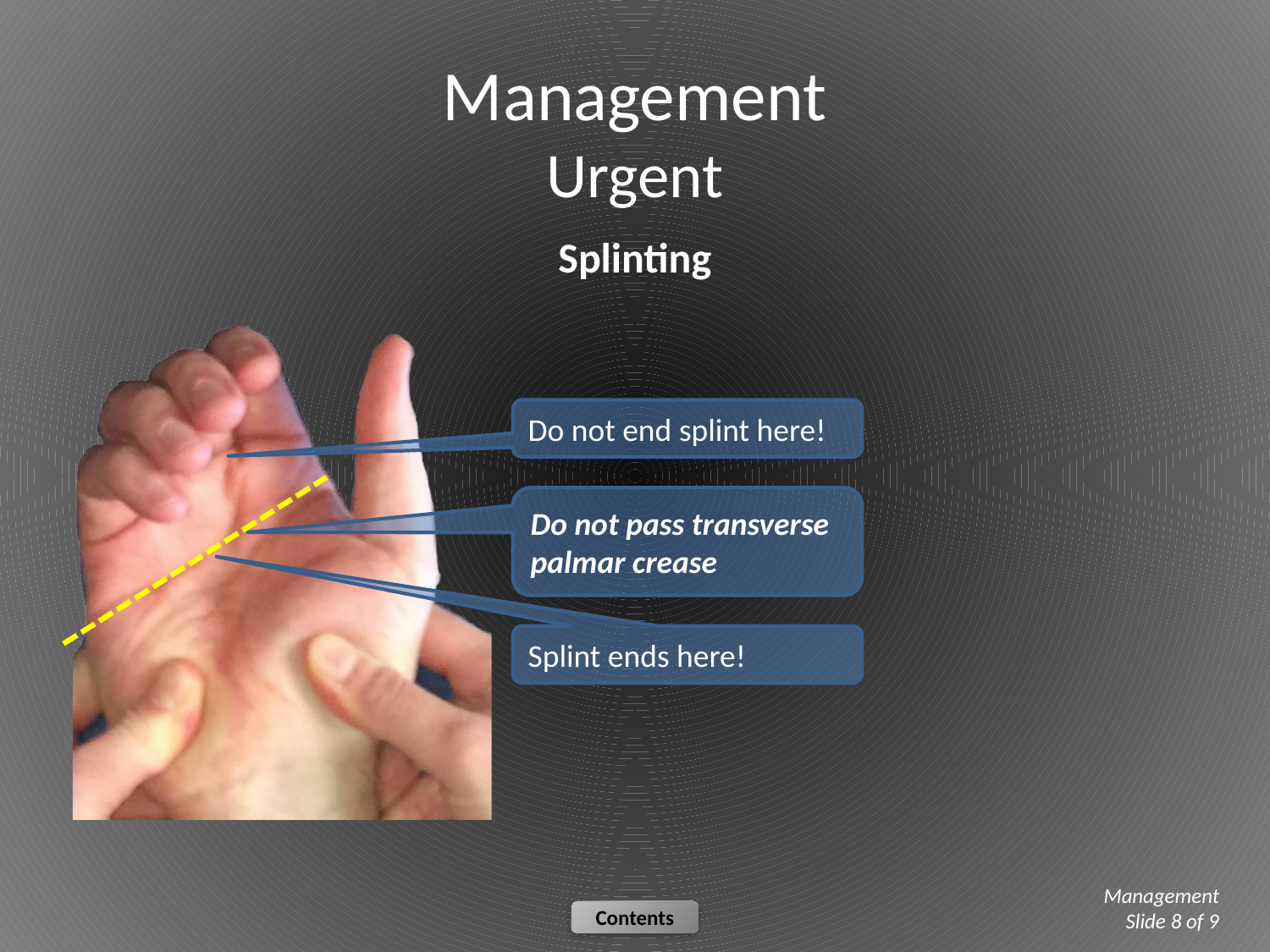

# Management Urgent
Splinting
Do not end splint here!
Do not pass transverse palmar crease
Splint ends here!
Management
Slide 8 of 9
Contents

## Slide 45
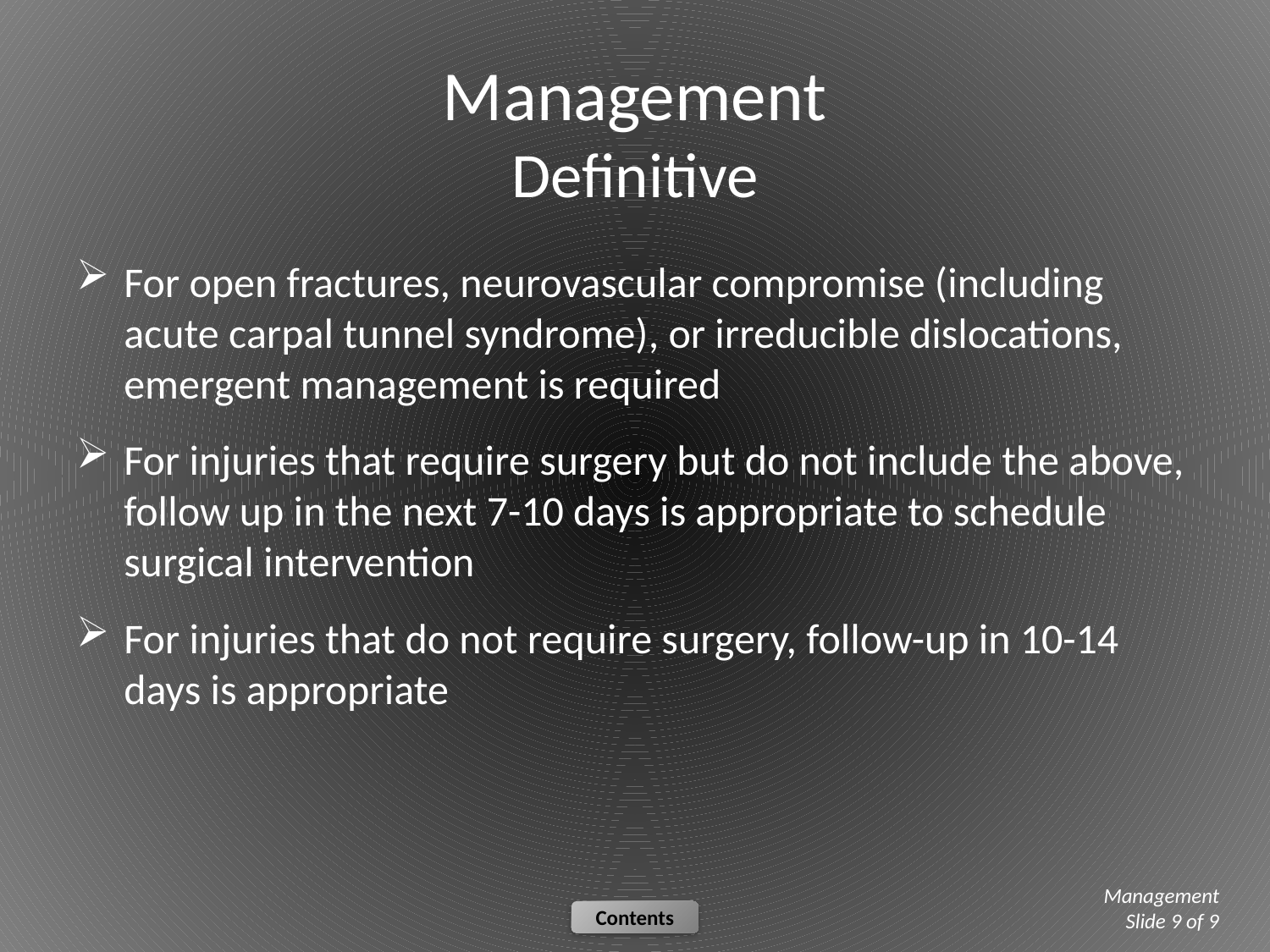

# Management Definitive
For open fractures, neurovascular compromise (including acute carpal tunnel syndrome), or irreducible dislocations, emergent management is required
For injuries that require surgery but do not include the above, follow up in the next 7-10 days is appropriate to schedule surgical intervention
For injuries that do not require surgery, follow-up in 10-14 days is appropriate
Management
Slide 9 of 9
Contents

## Slide 46
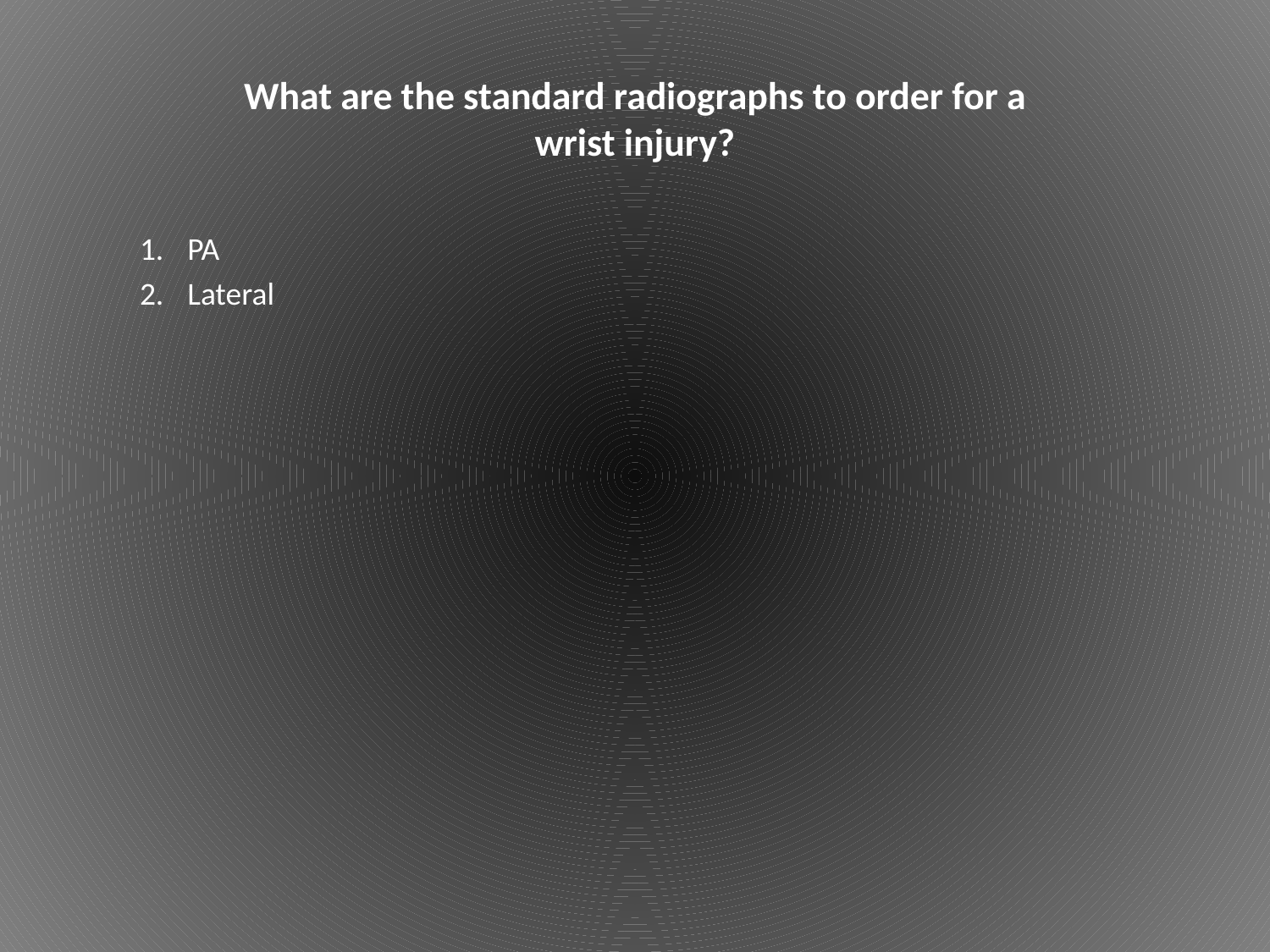

# What are the standard radiographs to order for a wrist injury?
PA
Lateral

## Slide 47
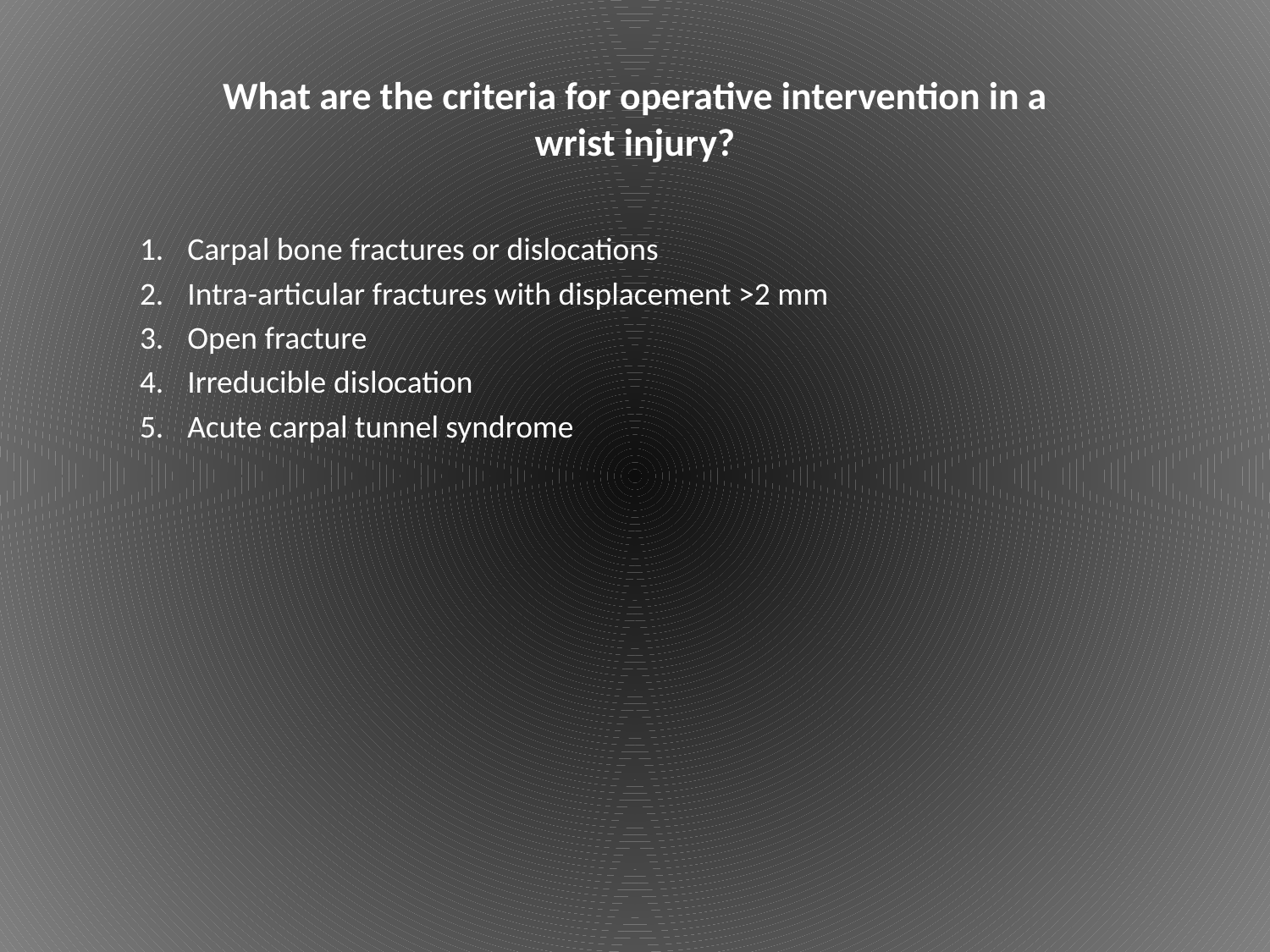

# What are the criteria for operative intervention in a wrist injury?
Carpal bone fractures or dislocations
Intra-articular fractures with displacement >2 mm
Open fracture
Irreducible dislocation
Acute carpal tunnel syndrome

## Slide 48
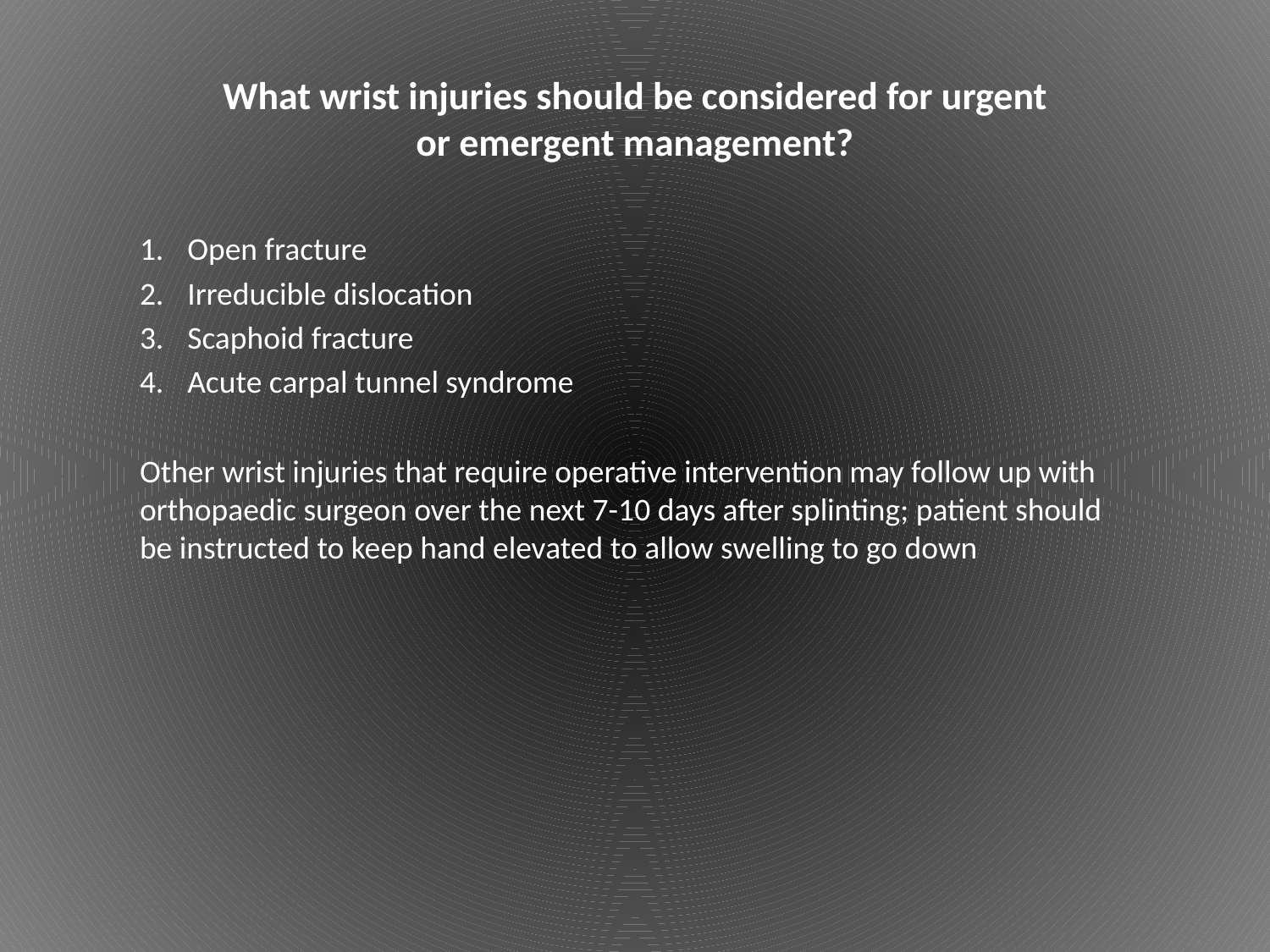

# What wrist injuries should be considered for urgent or emergent management?
Open fracture
Irreducible dislocation
Scaphoid fracture
Acute carpal tunnel syndrome
Other wrist injuries that require operative intervention may follow up with orthopaedic surgeon over the next 7-10 days after splinting; patient should be instructed to keep hand elevated to allow swelling to go down

## Slide 49
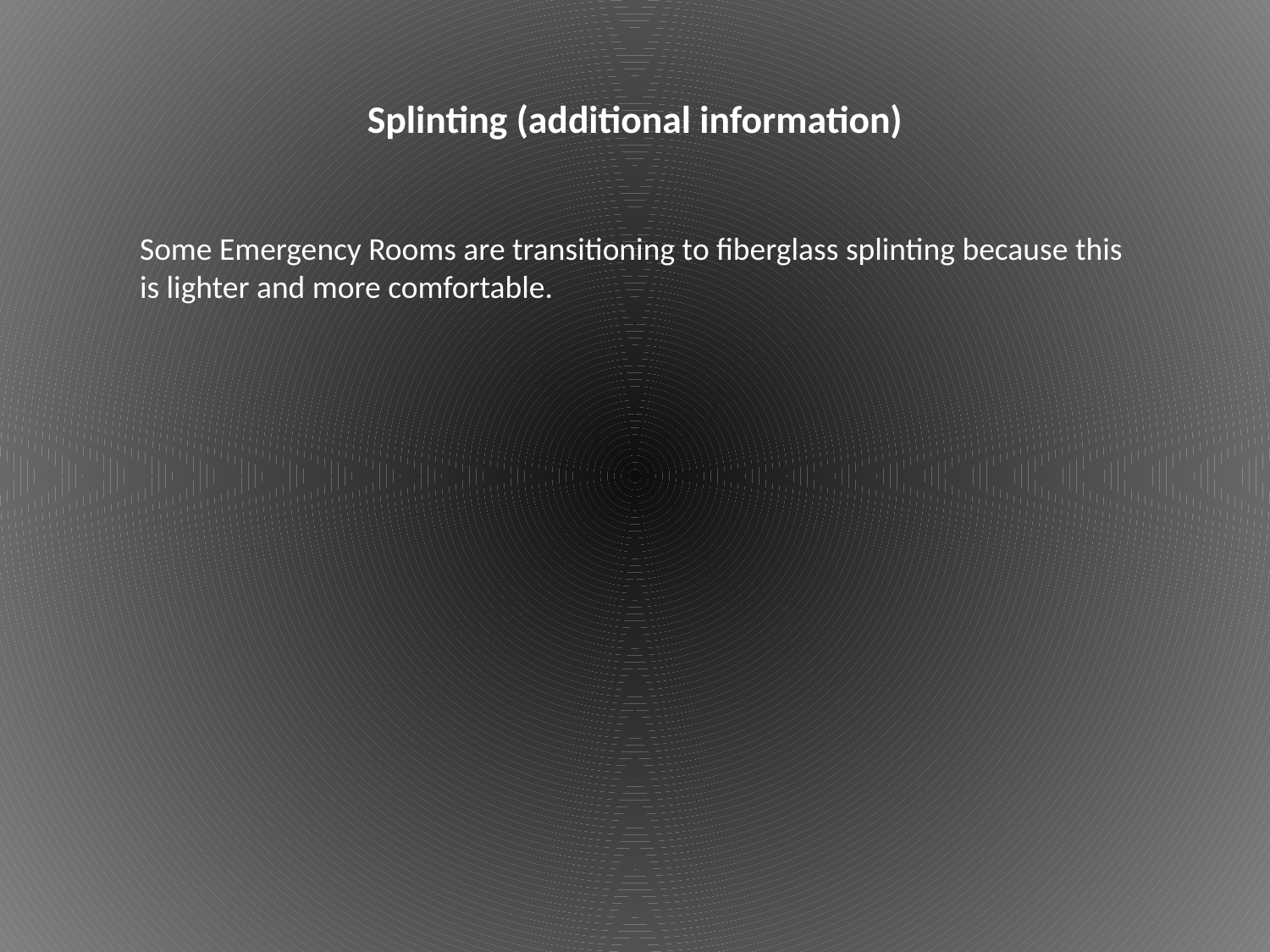

# Splinting (additional information)
Some Emergency Rooms are transitioning to fiberglass splinting because this is lighter and more comfortable.
