# Supplementary material for: Evaluation of Common Musculoskeletal Injuries in the Urgent Setting
Source: MedEdPORTAL. 2016 Dec 7;12:10514. doi: 10.15766/mep_2374-8265.10514 (PMC6440529; doi:10.15766/mep_2374-8265.10514)
Supplement: Supplementary file 1 — A. Evaluation of Common Musculoskeletal Injuries in the Urgent Setting.pptx B. Evaluation of Ankle Injuries in the Urgent Setting.pptx C. Evaluation of Hip Injuries in the Urgent Setting.pptx D. Evaluation of Shoulder Injuries in the Urgent Setting.pptx E. Evaluation of Wrist Injuries in the Urgent Setting.pptx [file mep-12-10514-s001.zip › _Educational Summary Report_10514.pdf]

MedEdPORTAL®
The Journal of Teaching and Learning Resources

Crossref

Original Publication

OPEN ACCESS

Evaluation of Common Musculoskeletal Injuries in the Urgent Setting

Anna N. Miller, MD*
*Corresponding author: anmiller@gmail.com

Citation: Miller AN. Evaluation of common musculoskeletal injuries in the urgent setting. *MedEdPORTAL*. 2016;12:10514. https://doi.org/10.15766/mep_2374-8265.10514

Copyright: © 2016 Miller. This is an open-access article distributed under the terms of the Creative Commons Attribution license.

Abstract

Introduction: Musculoskeletal (MSK) complaints comprise approximately 20% of primary care and emergency department visits annually in the United States. At the same time, there is a shortage of MSK specialists specifically trained to evaluate and treat these conditions. Improper management of patients with MSK diagnoses increases morbidity and undesirable outcomes for patients and leads to higher health care costs resulting from extraneous tests and imaging. **Methods:** This resource consists of a set of four modules reviewing ankle, hip, shoulder, and wrist injuries and their initial treatment. Each module is a PowerPoint presentation formulated as a self-study guide for the evaluation of common MSK injuries by primary care and emergency medicine providers (initial providers). Each module includes material on physical examination, imaging, and management, including reduction and splinting, as well as indications for referral to an orthopedic specialist. **Results:** The efficacy of the course for 43 first-year residents in internal medicine, orthopedic surgery, and the emergency department was measured using an external instrument. Pre- and posttest scores improved by 28% for knowledge of the material and by 30% for confidence level with the disposition of these injuries. All participants stated they were moderately to extremely satisfied with the course. **Discussion:** Completion of this course will result in improved evaluation and management of injuries by initial providers, including—where appropriate—improved communication between these providers and treating surgeons.

Keywords

Evaluation, Shoulder, Wrist, Injury, Musculoskeletal, Ankle, Fracture, Hip, Dislocation

Appendices

A. Evaluation of Common Musculoskeletal Injuries in the Urgent Setting.pptx
B. Evaluation of Ankle Injuries in the Urgent Setting.pptx
C. Evaluation of Hip Injuries in the Urgent Setting.pptx
D. Evaluation of Shoulder Injuries in the Urgent Setting.pptx
E. Evaluation of Wrist Injuries in the Urgent Setting.pptx

All appendices are peer reviewed as integral parts of the Original Publication.

Educational Objectives

On completion of each module, the learner will be able to:

1. Conduct a physical exam for an injury.
2. Appropriately order radiographic examination evaluation of an injury.
3. Evaluate radiographs and determine appropriate surgical and nonsurgical interventions.
4. Describe the general technique for nonsurgical treatment of injuries.
5. Describe the urgency of follow-up for patients with a variety of injuries.

Introduction

Musculoskeletal (MSK) complaints comprise approximately 20% of primary care and emergency department visits annually in the United States.[1] The Centers for Disease Control reported over 40 million encounters for MSK disease in these two settings in 2006, not including another 49 million injurious presentations, a large percentage of which are MSK in nature.[2] MSK conditions also disproportionately affect individuals older than 64 years of age; thus, these numbers are expected to increase as the population ages. Concurrently, there is a shortage of MSK specialists specifically trained to evaluate and treat these conditions.[3] Improper management of patients with MSK diagnoses increases morbidity and undesirable outcomes for the patients and leads to higher health care costs resulting from extraneous tests and imaging.

Establishing competence in this field should be a priority for all medical graduates, especially those entering primary care and emergency medicine fields. MSK education at the medical school level in the United States has been notoriously lacking. In 2003, only 65 (45%) of 122 medical schools required MSK courses as part of their curricula. Over the last decade, this has improved to 93% of schools having some MSK requirement, but quantity may not translate to quality.[4] In a 1998 study, 82% of medical graduates failed a test of MSK competency, and a similar study in 2012 (after most medical schools expanded MSK education) reported negligible improvement, with an 81% failure rate.[5] Baseline knowledge of simple fracture care is lacking even among incoming orthopedic residents, as was demonstrated by a multisite survey presented at the Orthopaedic Trauma Association's 2013 annual meeting.[6]

Finally, standardized measures of competence are only one aspect of effective patient care and mean little in the absence of physician confidence and comfort in managing these patients. Perhaps the most telling data show that practicing physicians do not feel properly trained to appropriately treat orthopedic complaints. In one survey, only 51% of respondents felt their MSK training in medical school was adequate.[7] A more-recent study found that among primary care physicians, there was a statistically significant difference between their level of comfort with medical issues and MSK issues.[8]

This resource was created to provide a foundation for management of common MSK injuries that primary care and emergency medicine providers (initial providers) may be routinely exposed to in their practice. It was created by an attending orthopedic trauma surgeon with the goal of improving both evaluation and urgent management of this patient population. In addition, communication between these initial providers and orthopedic surgeons is facilitated by using the same terminology. Finally, the urgency of care of these patients is noted to avoid unnecessarily urgent office visits or hospital stays while continuing to maintain patient safety.

The resource was developed based on the experience of orthopedic trauma surgeons on the most important points of the urgent assessment and management of these patients necessary to instruct the learner and improve the knowledge of referring physicians in these areas. As the surgeons who are ultimately responsible for the long-term treatment of these patients, referring physicians should understand that these are the points to provide the best possible care initially. The material may be of particular utility to initial providers in settings where orthopedic surgeons may not be an immediately accessible resource. The self-assessment questions were derived from consensus from orthopedic surgeons, commonly tested areas on orthopedic board examinations, and MSK testing tools that have previously been published in the literature.[7]

Methods

This resource is designed as a set of independent self-study modules, recognizing that the principal target audience is adult, self-directed learners. It was designed as a tool to be studied in its entirety or quickly implemented as a refresher for providers who are managing these common injuries in an emergency, urgent, or primary care setting. It may be used as a way to check on management or as a reminder of anatomic and physical examination pointers that are necessary for providers to work through. It is also a resource for appropriate radiographic imaging, although initial providers taking this course should already be versed in radiographic imaging and reading radiographs is not a main learning point of the resource per se. The resource covers the most significant aspects of urgent handling of injury: physical exam, imaging, management, and referral. Each module—ankle (Appendix B), hip (Appendix C), shoulder (Appendix D), and wrist (Appendix E)—may be navigated independently from a table of contents slide (Appendix A). The resource includes a set of self-assessment questions, together with an extensive set of slides as shown in the Table.

Note that with respect to the physical exam, the ankle and hip modules share six slides, and the shoulder and wrist modules share eight slides. Completion of each module in the resource on any electronic device capable of running PowerPoint should normally take no more than 1 hour.

Table. Number of Slides

Module	Physical Exam	Radiographic Imaging	Types of Fracture	Management	Self-Assessment	Other	Total
Ankle	8[a]	6	26	5	5	7	57
Hip	7[a]	6	14	4	4	6	41
Shoulder	9[b]	4	21	5	4	4	47
Wrist	8[b]	5	17	9	4	6	49
Total	32	21	78	23	17	23	194

[a]With respect to Physical Exam, Ankle and Hip share six slides.
[b]With respect to Physical Exam, Shoulder and Wrist share eight slides.

Results

After creation of this resource, the course was given to our incoming residents to test its efficacy. We specifically wanted to confirm that the course material was understandable, that participants were getting the material they needed from it in order to improve on related examination questions, and that they felt they were getting useful information from it by evaluating their comfort level with the knowledge and treatment of these injuries before and after using the resources. The efficacy of the course was shown based on testing of 43 first-year residents in internal medicine, orthopedic surgery, and the emergency department, as previously discussed. Pre- and posttest scores improved by 28% for knowledge of the material and by 30% for confidence level with the disposition of these injuries. All participants stated they were moderately to extremely satisfied with the course.

Discussion

This resource was originally designed to resolve a practice gap in the management of common MSK injuries identified by practicing orthopedic surgeons when reviewing the performance of referring physicians. As indicated in the Results section, the use of the resource has resulted in improvements in performance for its target audience. Nevertheless, to address suggestions for improvement by learners and other reviewers, it has been significantly enhanced since its original implementation, primarily in the presentation of the sections on physical exam and radiographic imaging of injuries.

The main limitation of the resource at this time is the lack of hands-on experience in the reduction of any displaced fracture or dislocation. Thus, additional benefits for the intended audience might accrue from using the resource in a small-group, workshop setting (e.g., five to 10 participants) facilitated by a practicing orthopedic surgeon and/or MSK-trained sports medicine physician. Such a setting would also be conducive to a more-objective assessment of gains in both knowledge and skill.

Other populations (e.g., medical students, residents, physician assistants, and nurse practitioners) might also benefit from completing this course, though they may need assistance from an attending physician with experience in MSK medicine to achieve a full understanding of the material. Interpretation of the radiographic images may require assistance from a practicing radiologist.

Anna N. Miller, MD: Assistant Professor, Department of Orthopaedic Surgery, Wake Forest University School of Medicine of Medicine of Wake Forest Baptist Medical Center; the Assistant Director of Orthopaedic Trauma, Wake Forest University School of Medicine of Medicine of Wake Forest Baptist Medical Center

Disclosures
None to report.

Funding/Support
Brooks Scholar Fellowship from Wake Forest Baptist Medical Center funding to help design a musculoskeletal education program for primary care physicians.

Ethical Approval
Reported as not applicable.

References

1. Grunfeld R, Banks S, Fox E, Levy BA, Craig C, Black K. An assessment of musculoskeletal knowledge in graduating medical and physician assistant students and implications for musculoskeletal care providers. *J Bone Joint Surg Am.* 2012;94(4)343-348. http://dx.doi.org/10.2106/JBJS.J.00417
2. Schappert SM, Rechtsteiner EA. *Ambulatory Medical Care Utilization Estimates for 2007.* Hyattsville, MD: National Center for Health Statistics; 2011. DHHS publication (PHS) 2011-1740.
3. Burden of Musculoskeletal Diseases in the United States Web site. http://www.boneandjointburden.org. Accessed April 26, 2016.
4. Bernstein J, Garcia GH, Guevara JL, Mitchell GW. Progress report: the prevalence of required medical school instruction in musculoskeletal medicine at decade's end. *Clin Orthop Relat Res.* 2011;469(3):895-897. http://dx.doi.org/10.1007/s11999-010-1477-3
5. Medical School Objectives Project. Report VII: contemporary issues in medicine: musculoskeletal medicine education [member-only publication]. AAMC Web site. https://members.aamc.org/eweb/upload/Contemporary%20Issues%20in%20Med%20Musculoskeletal%20Med%20Report%20VII%20.pdf. Published September 2005. Accessed April 26, 2016.
6. Graves M, Paryavi E, Faulhaber A, Reilly MC, Baumgaertner M, O'Toole RV. Treating the trauma knowledge gap: a validated approach to understanding resident knowledge and addressing deficiencies. Poster presented at: Orthopaedic Trauma Association 2013 Annual Meeting; October 9-12, 2013; Phoenix, AZ.
7. Freedman KB, Bernstein J. The adequacy of medical school education in musculoskeletal medicine. *J Bone Joint Surg Am.* 1998;80(10):1421-1427.
8. Lynch JR, Schmale GA, Schaad DC, Leopold SS. Important demographic variables impact the musculoskeletal knowledge and confidence of academic primary care physicians. *J Bone Joint Surg Am.* 2006;88(7):1589-1595. http://dx.doi.org/10.2106/JBJS.E.01365

Received: July 21, 2016 | **Accepted:** November 7, 2016 | **Published:** December 7, 2016
