# Supplementary material for: Evaluation of Common Musculoskeletal Injuries in the Urgent Setting
Source: MedEdPORTAL. 2016 Dec 7;12:10514. doi: 10.15766/mep_2374-8265.10514 (PMC6440529; doi:10.15766/mep_2374-8265.10514)
Supplement: Supplementary file 1 — A. Evaluation of Common Musculoskeletal Injuries in the Urgent Setting.pptx B. Evaluation of Ankle Injuries in the Urgent Setting.pptx C. Evaluation of Hip Injuries in the Urgent Setting.pptx D. Evaluation of Shoulder Injuries in the Urgent Setting.pptx E. Evaluation of Wrist Injuries in the Urgent Setting.pptx [file mep-12-10514-s001.zip › A. Evaluation of Common Musculoskeletal Injuries in the Urgent Setting.pptx]

## Slide 1
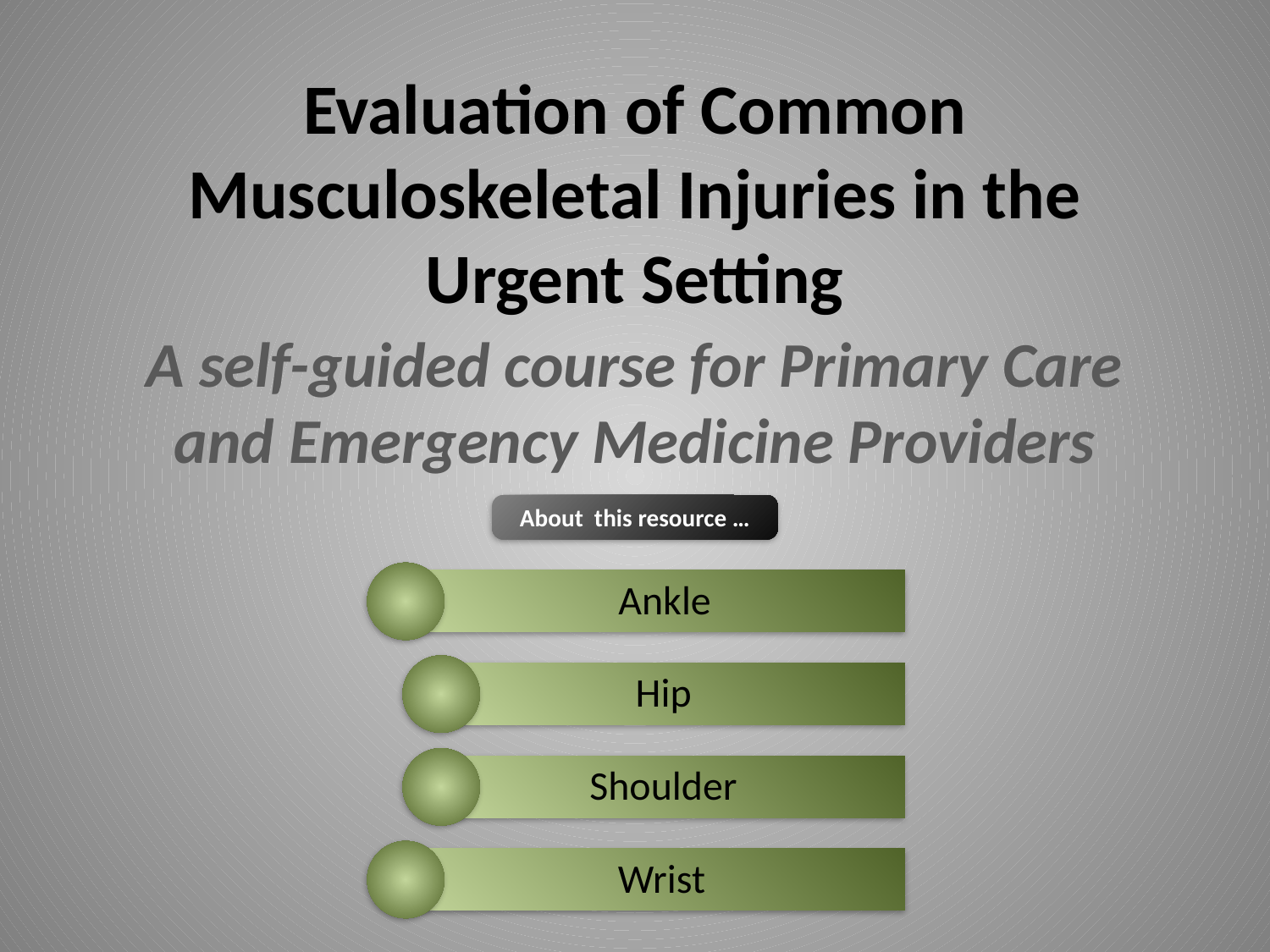

# Evaluation of Common Musculoskeletal Injuries in the Urgent Setting
A self-guided course for Primary Care and Emergency Medicine Providers
About this resource …

## Slide 2
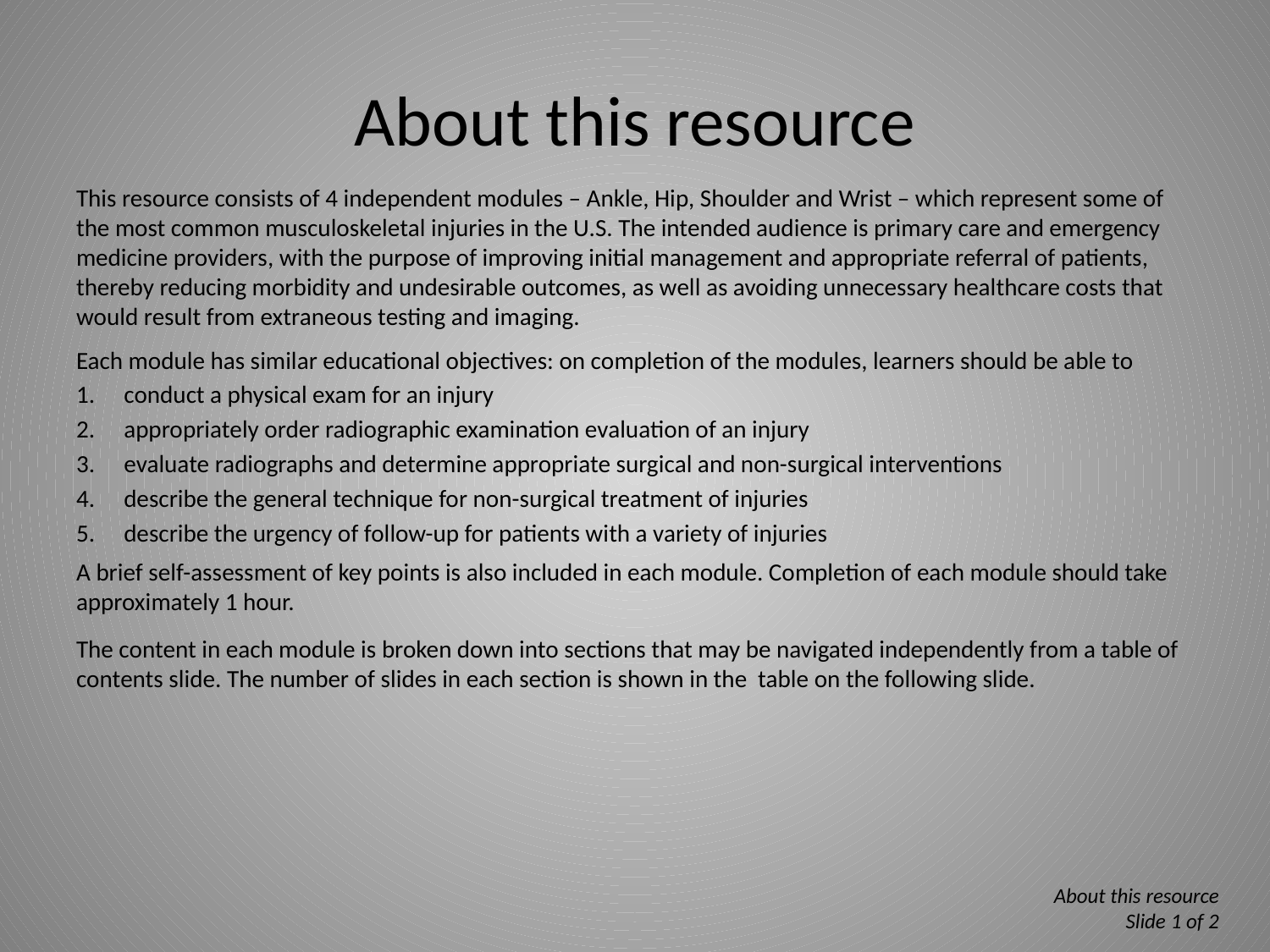

# About this resource
This resource consists of 4 independent modules – Ankle, Hip, Shoulder and Wrist – which represent some of the most common musculoskeletal injuries in the U.S. The intended audience is primary care and emergency medicine providers, with the purpose of improving initial management and appropriate referral of patients, thereby reducing morbidity and undesirable outcomes, as well as avoiding unnecessary healthcare costs that would result from extraneous testing and imaging.
Each module has similar educational objectives: on completion of the modules, learners should be able to
conduct a physical exam for an injury
appropriately order radiographic examination evaluation of an injury
evaluate radiographs and determine appropriate surgical and non-surgical interventions
describe the general technique for non-surgical treatment of injuries
describe the urgency of follow-up for patients with a variety of injuries
A brief self-assessment of key points is also included in each module. Completion of each module should take approximately 1 hour.
The content in each module is broken down into sections that may be navigated independently from a table of contents slide. The number of slides in each section is shown in the table on the following slide.
About this resource
Slide 1 of 2

## Slide 3
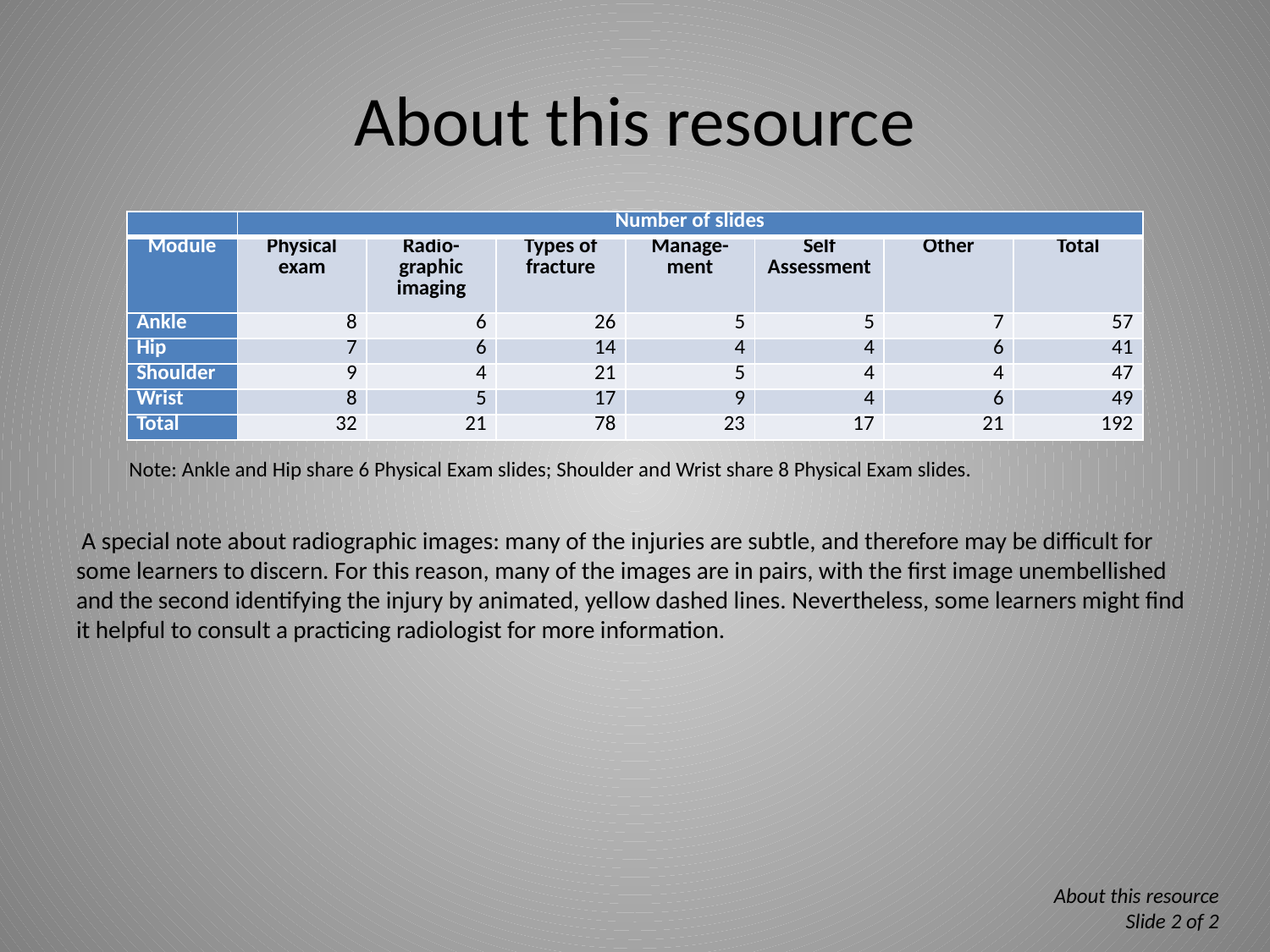

# About this resource
| | Number of slides | | | | | | |
| --- | --- | --- | --- | --- | --- | --- | --- |
| Module | Physical exam | Radio-graphic imaging | Types of fracture | Manage-ment | Self Assessment | Other | Total |
| Ankle | 8 | 6 | 26 | 5 | 5 | 7 | 57 |
| Hip | 7 | 6 | 14 | 4 | 4 | 6 | 41 |
| Shoulder | 9 | 4 | 21 | 5 | 4 | 4 | 47 |
| Wrist | 8 | 5 | 17 | 9 | 4 | 6 | 49 |
| Total | 32 | 21 | 78 | 23 | 17 | 21 | 192 |
Note: Ankle and Hip share 6 Physical Exam slides; Shoulder and Wrist share 8 Physical Exam slides.
 A special note about radiographic images: many of the injuries are subtle, and therefore may be difficult for some learners to discern. For this reason, many of the images are in pairs, with the first image unembellished and the second identifying the injury by animated, yellow dashed lines. Nevertheless, some learners might find it helpful to consult a practicing radiologist for more information.
About this resource
Slide 2 of 2
