# Supplementary material for: Evaluation of Common Musculoskeletal Injuries in the Urgent Setting
Source: MedEdPORTAL. 2016 Dec 7;12:10514. doi: 10.15766/mep_2374-8265.10514 (PMC6440529; doi:10.15766/mep_2374-8265.10514)
Supplement: Supplementary file 1 — A. Evaluation of Common Musculoskeletal Injuries in the Urgent Setting.pptx B. Evaluation of Ankle Injuries in the Urgent Setting.pptx C. Evaluation of Hip Injuries in the Urgent Setting.pptx D. Evaluation of Shoulder Injuries in the Urgent Setting.pptx E. Evaluation of Wrist Injuries in the Urgent Setting.pptx [file mep-12-10514-s001.zip › B. Evaluation of Ankle Injuries in the Urgent Setting.pptx]

## Slide 1
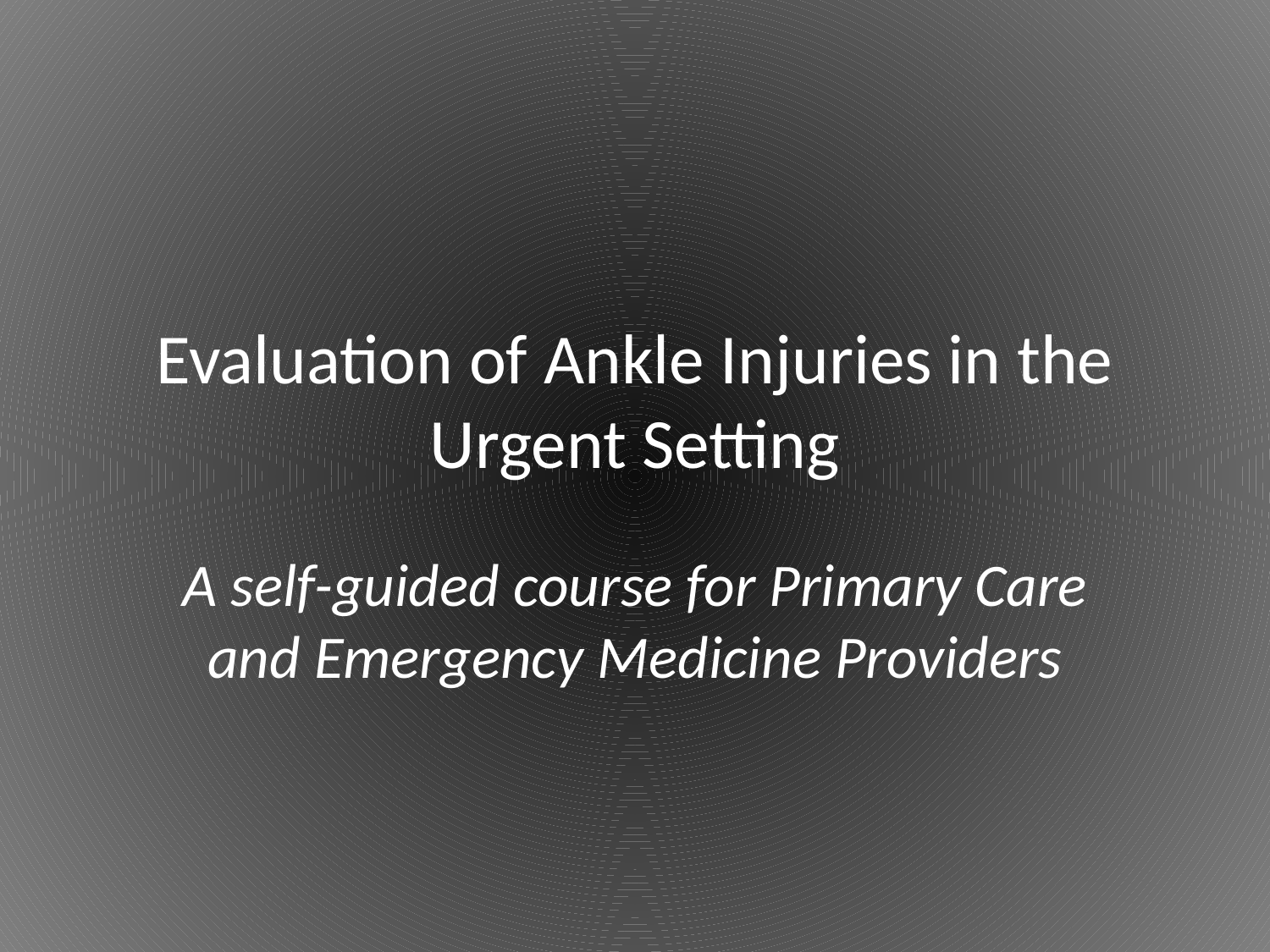

# Evaluation of Ankle Injuries in the Urgent Setting
A self-guided course for Primary Care and Emergency Medicine Providers

## Slide 2
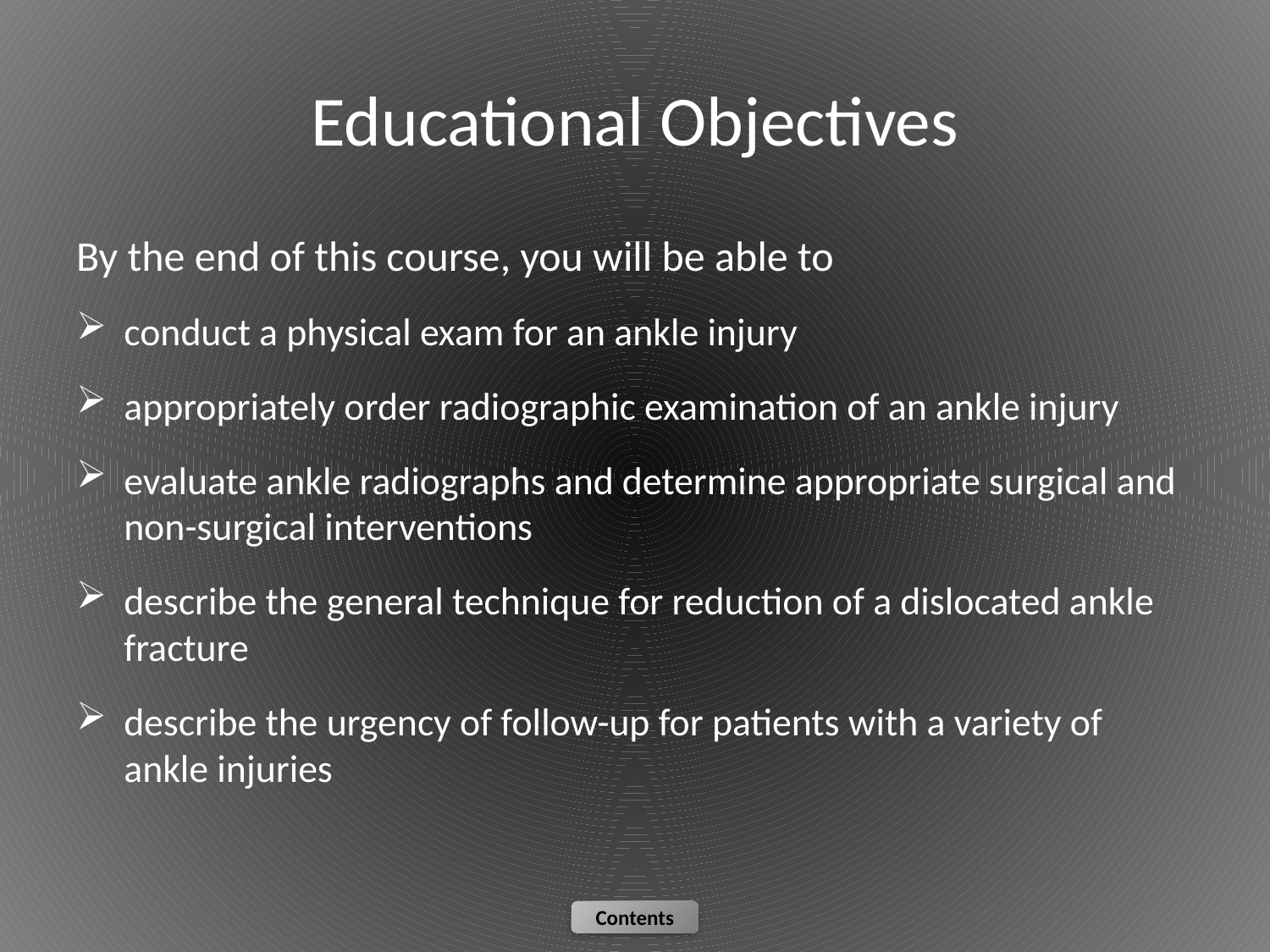

# Educational Objectives
By the end of this course, you will be able to
conduct a physical exam for an ankle injury
appropriately order radiographic examination of an ankle injury
evaluate ankle radiographs and determine appropriate surgical and non-surgical interventions
describe the general technique for reduction of a dislocated ankle fracture
describe the urgency of follow-up for patients with a variety of ankle injuries
Contents

## Slide 3
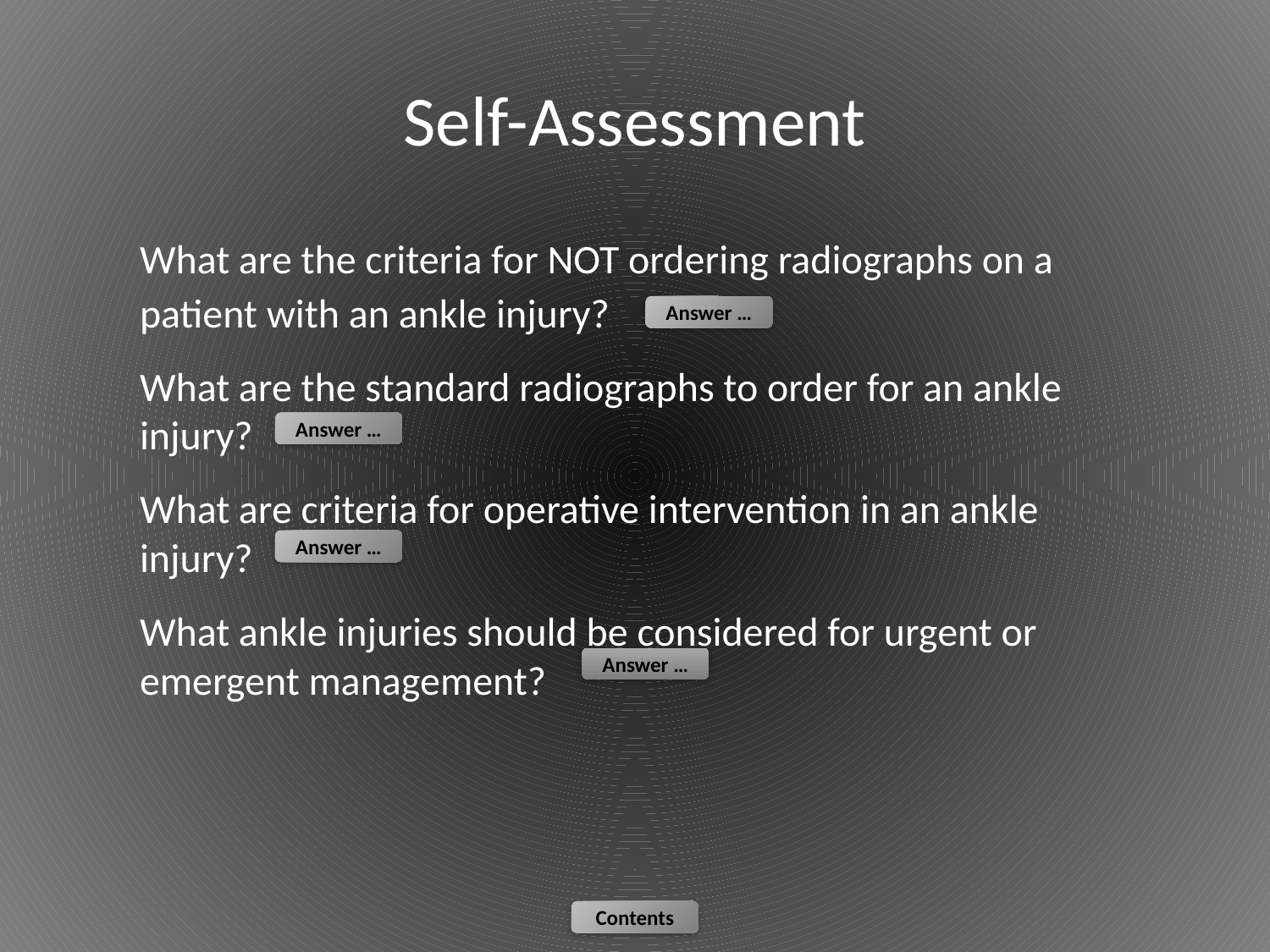

# Self-Assessment
What are the criteria for NOT ordering radiographs on a patient with an ankle injury?
What are the standard radiographs to order for an ankle injury?
What are criteria for operative intervention in an ankle injury?
What ankle injuries should be considered for urgent or emergent management?
Answer …
Answer …
Answer …
Answer …
Contents

## Slide 4
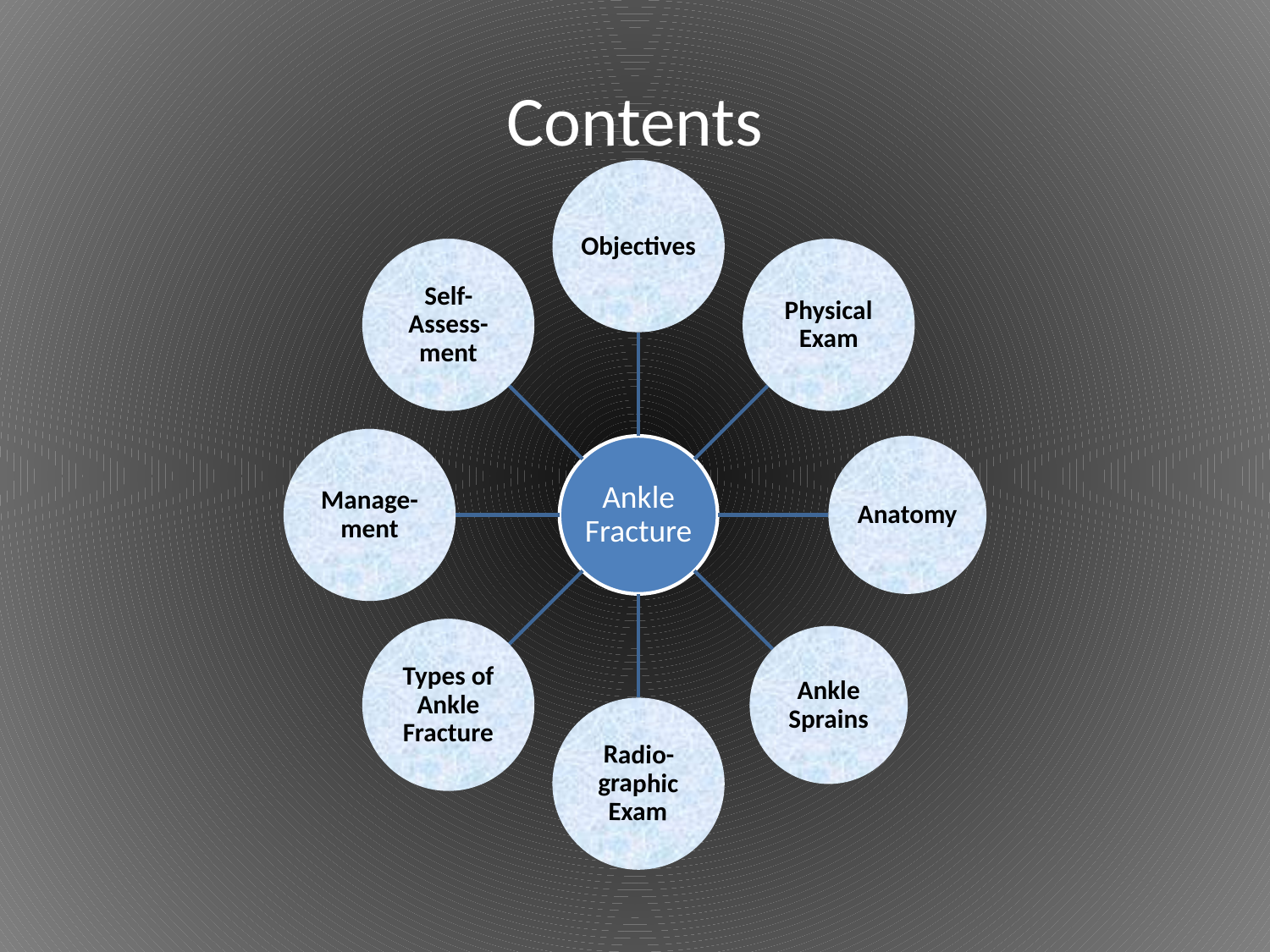

# Contents

## Slide 5
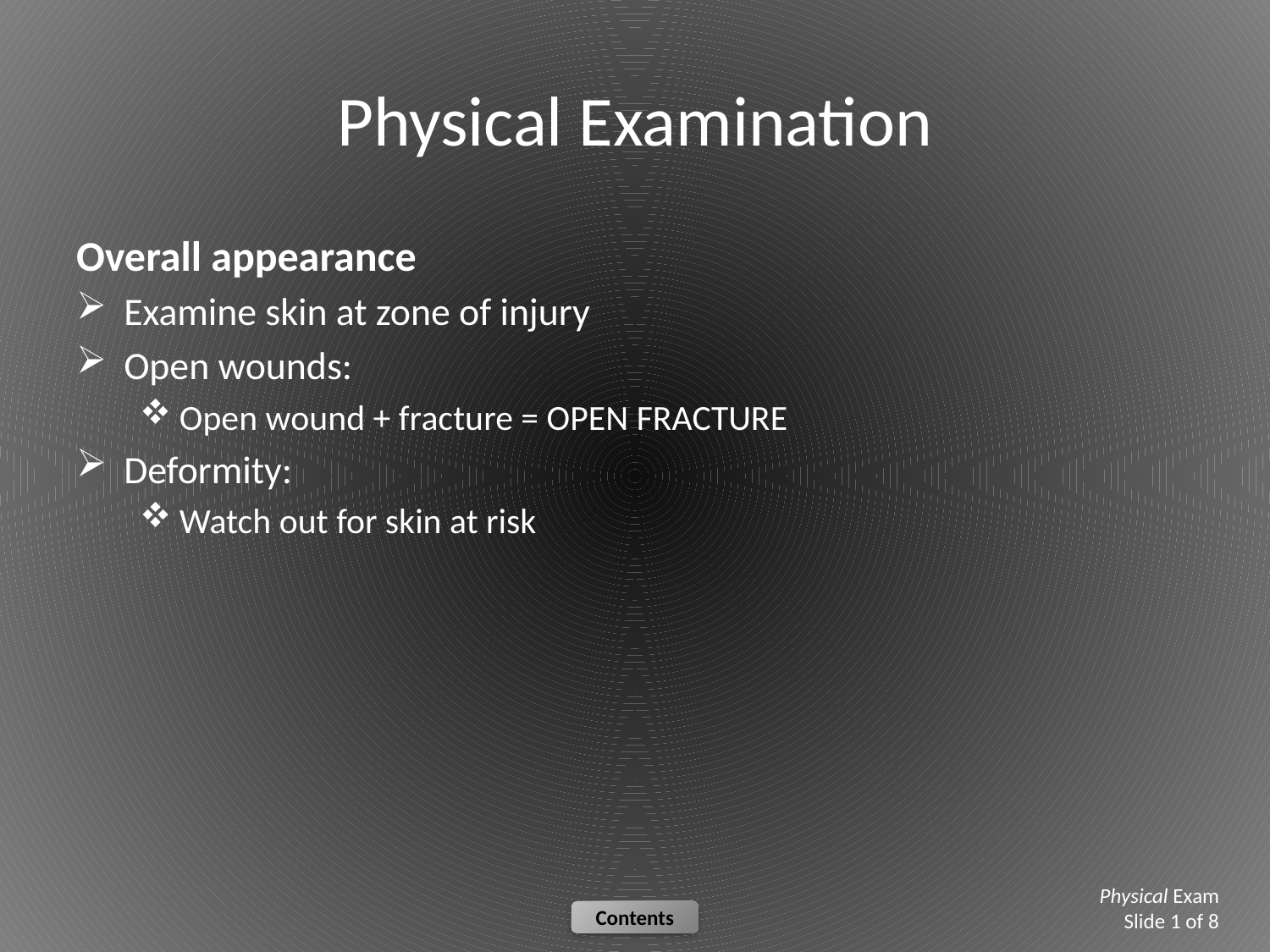

# Physical Examination
Overall appearance
Examine skin at zone of injury
Open wounds:
Open wound + fracture = OPEN FRACTURE
Deformity:
Watch out for skin at risk
Physical Exam
Slide 1 of 8
Contents

## Slide 6
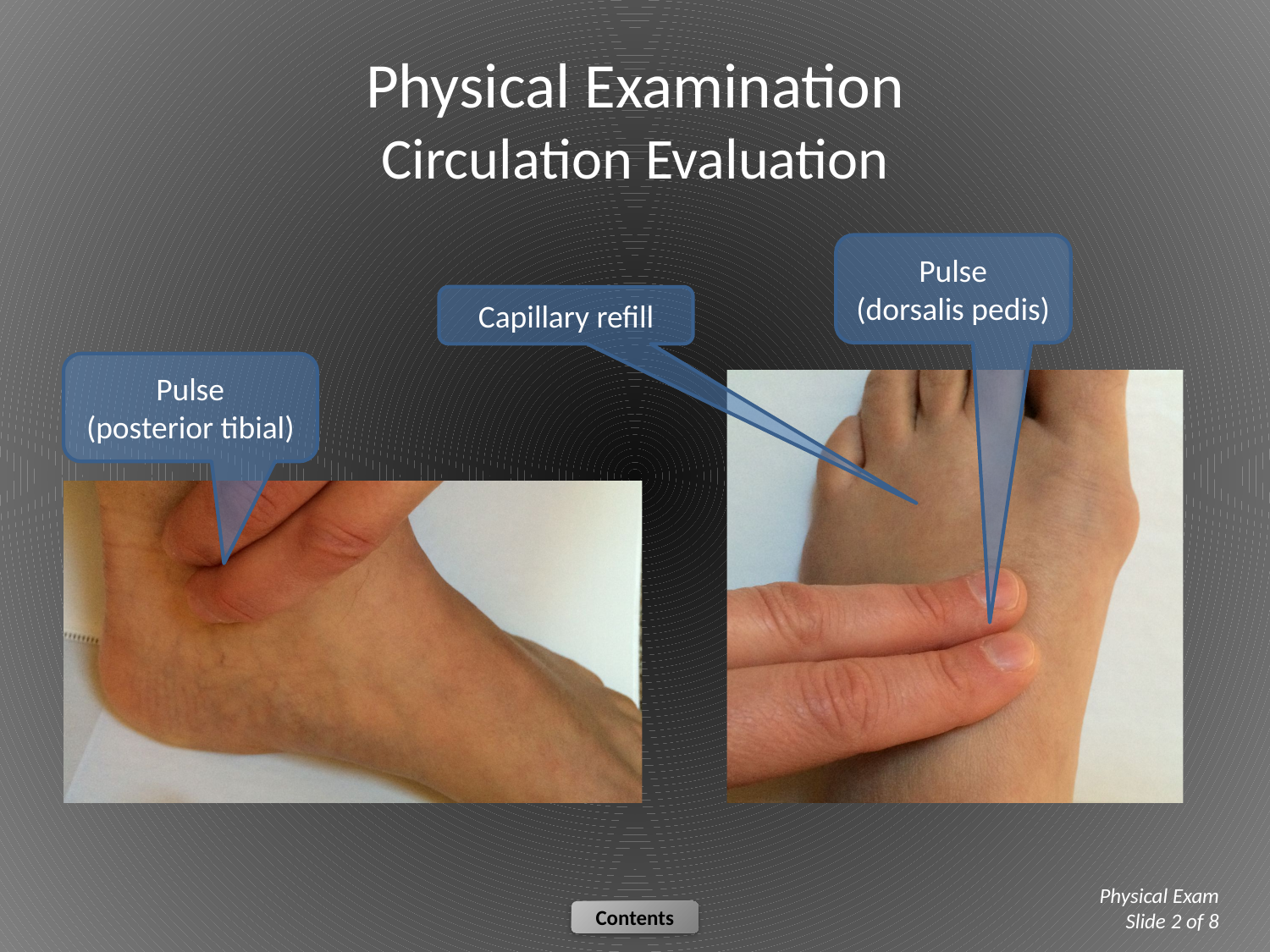

# Physical Examination Circulation Evaluation
Pulse
(dorsalis pedis)
Capillary refill
Pulse
(posterior tibial)
Physical Exam
Slide 2 of 8
Contents

## Slide 7
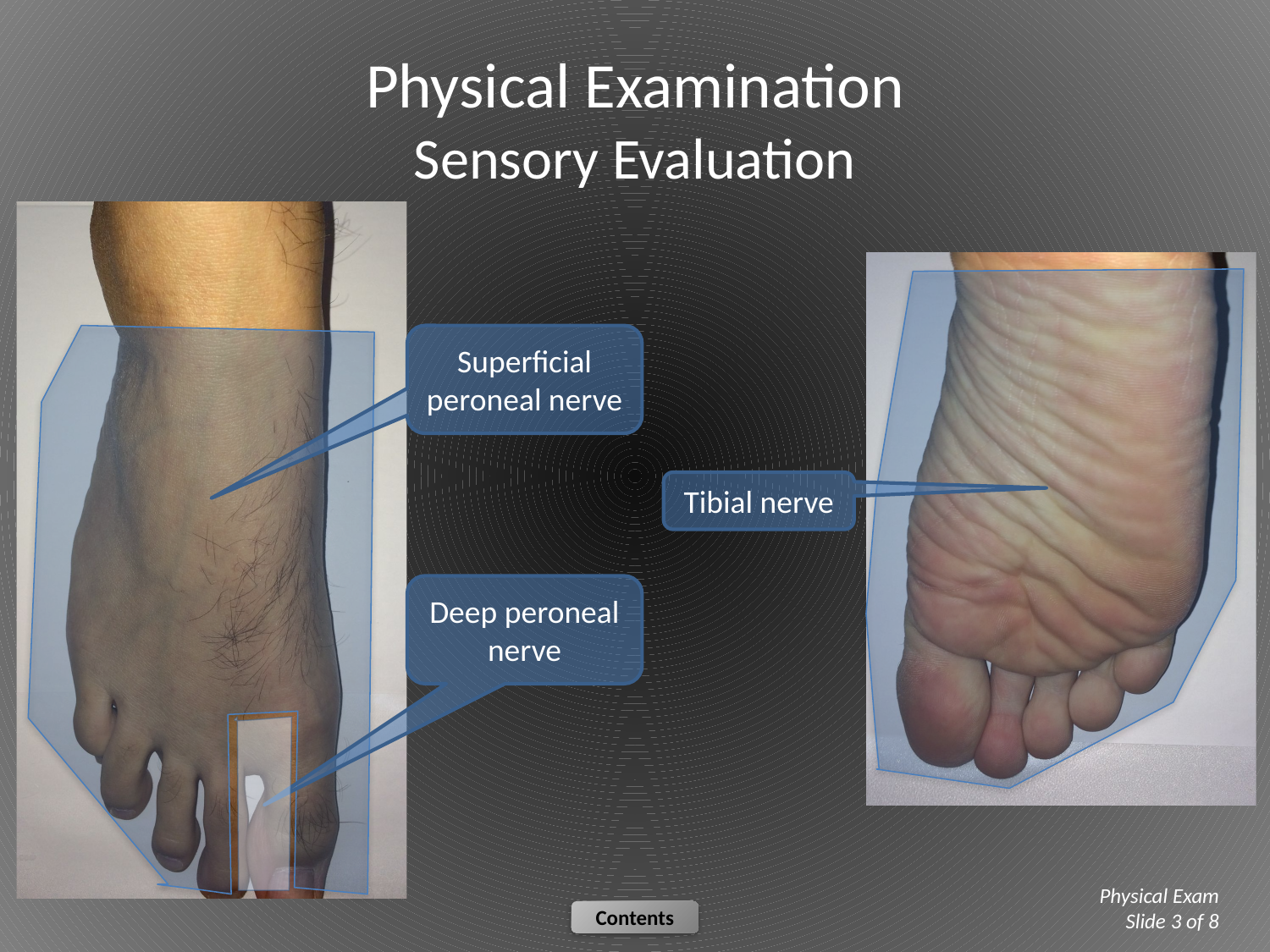

# Physical Examination Sensory Evaluation
Superficial peroneal nerve
Tibial nerve
Deep peroneal nerve
Physical Exam
Slide 3 of 8
Contents

## Slide 8
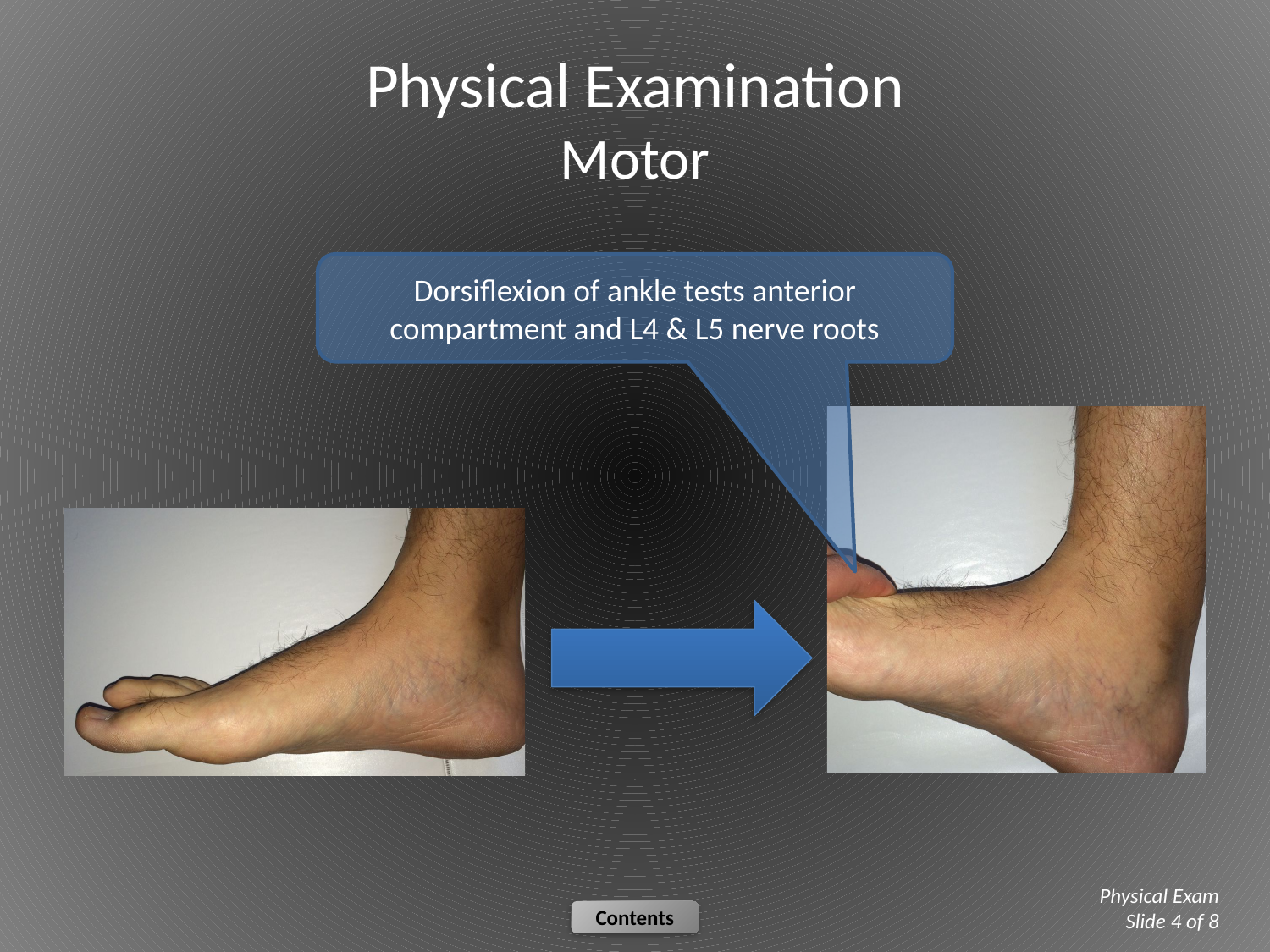

# Physical Examination Motor
Dorsiflexion of ankle tests anterior compartment and L4 & L5 nerve roots
Physical Exam
Slide 4 of 8
Contents

## Slide 9
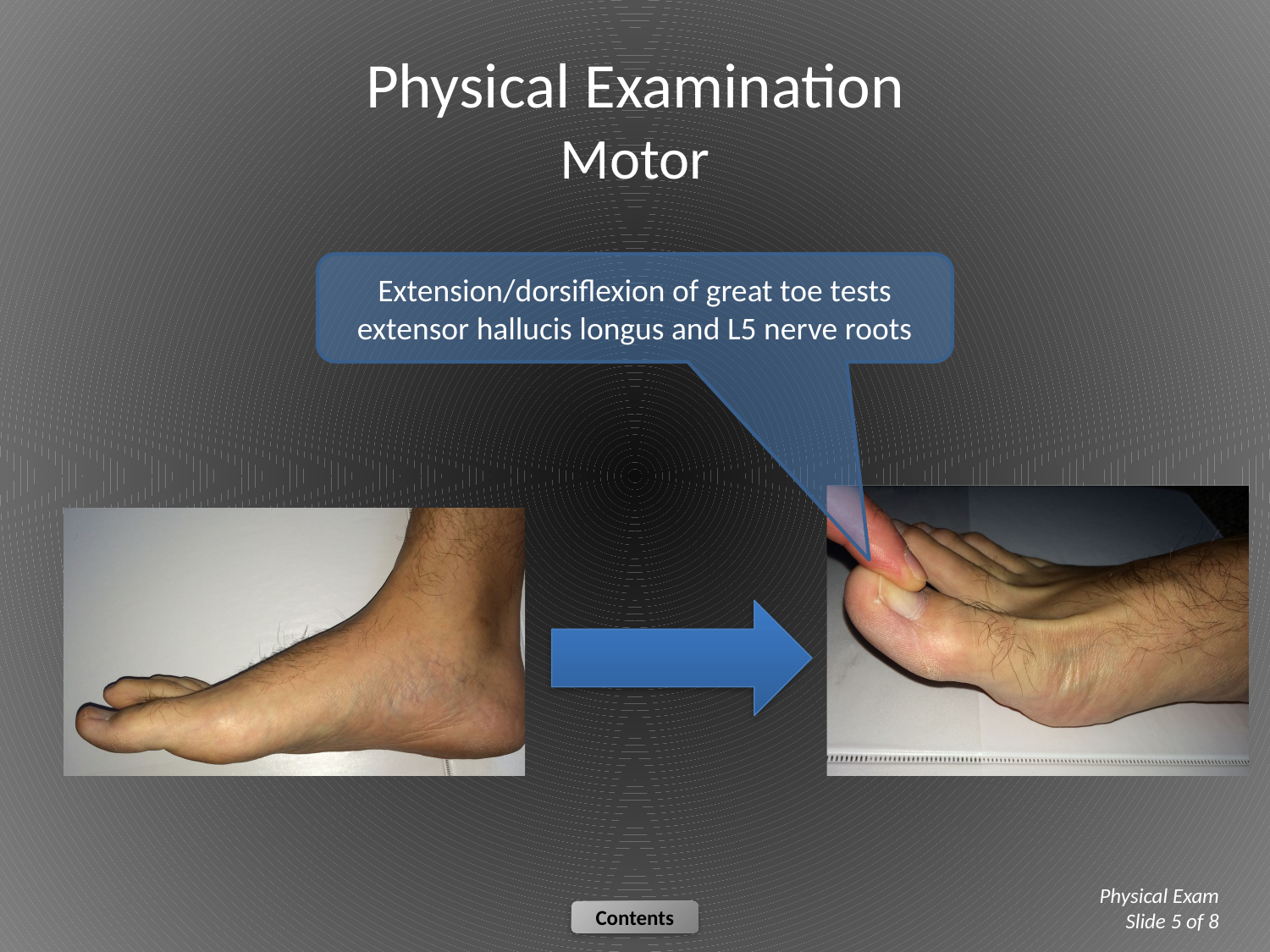

# Physical Examination Motor
Extension/dorsiflexion of great toe tests extensor hallucis longus and L5 nerve roots
Physical Exam
Slide 5 of 8
Contents

## Slide 10
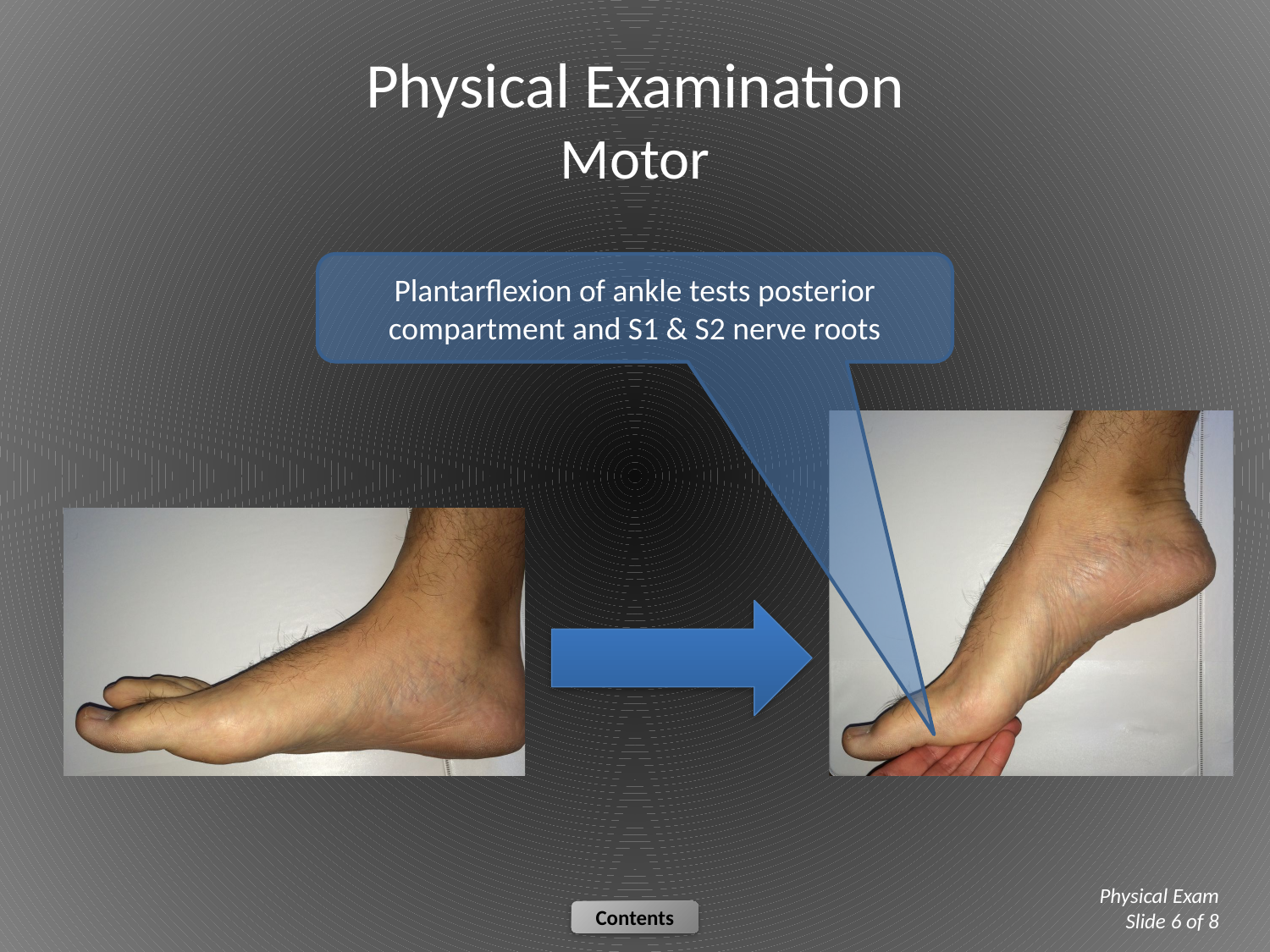

# Physical Examination Motor
Plantarflexion of ankle tests posterior compartment and S1 & S2 nerve roots
Physical Exam
Slide 6 of 8
Contents

## Slide 11
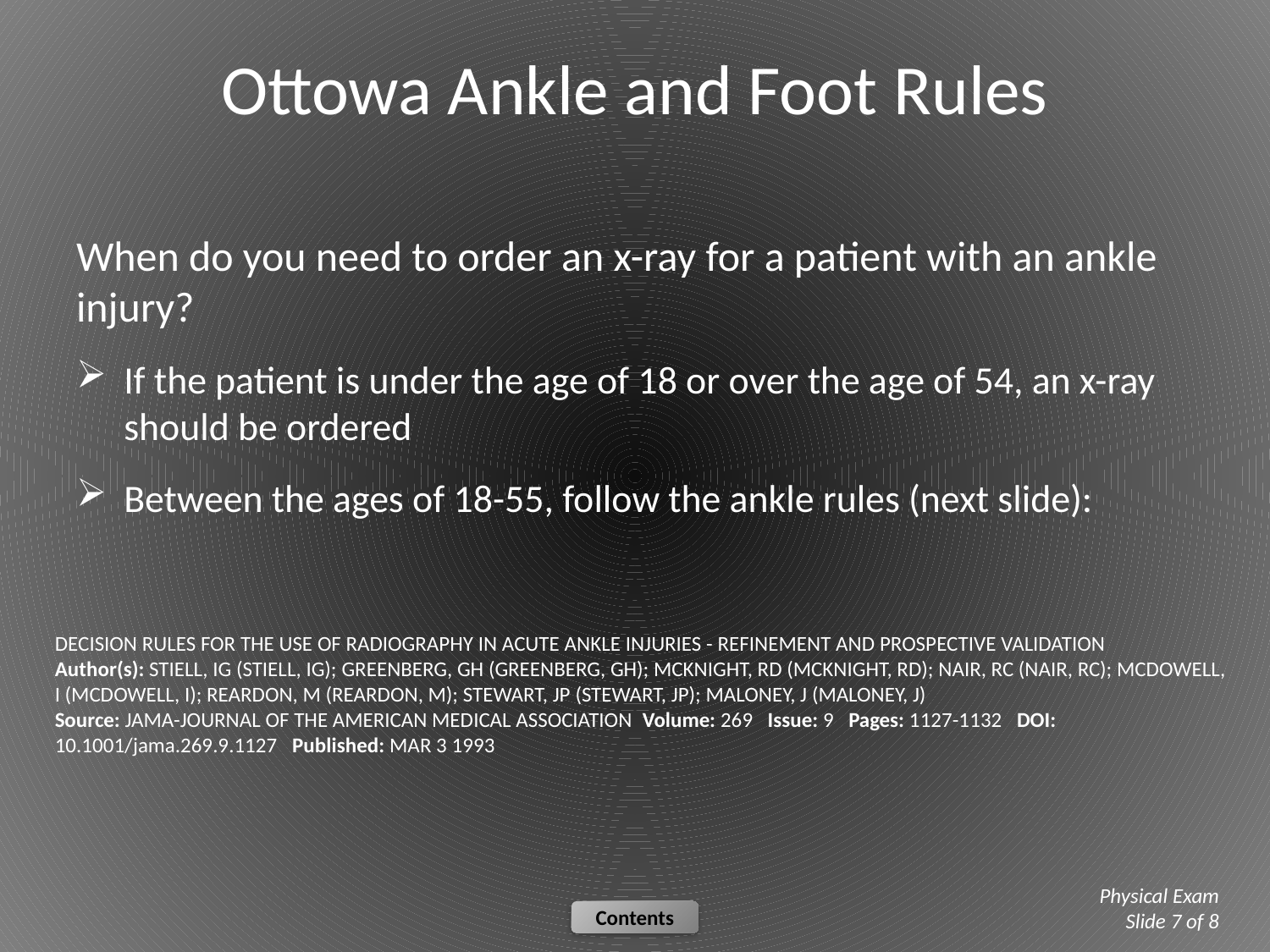

Ottowa Ankle and Foot Rules
When do you need to order an x-ray for a patient with an ankle injury?
If the patient is under the age of 18 or over the age of 54, an x-ray should be ordered
Between the ages of 18-55, follow the ankle rules (next slide):
DECISION RULES FOR THE USE OF RADIOGRAPHY IN ACUTE ANKLE INJURIES - REFINEMENT AND PROSPECTIVE VALIDATION
Author(s): STIELL, IG (STIELL, IG); GREENBERG, GH (GREENBERG, GH); MCKNIGHT, RD (MCKNIGHT, RD); NAIR, RC (NAIR, RC); MCDOWELL, I (MCDOWELL, I); REARDON, M (REARDON, M); STEWART, JP (STEWART, JP); MALONEY, J (MALONEY, J)
Source: JAMA-JOURNAL OF THE AMERICAN MEDICAL ASSOCIATION  Volume: 269   Issue: 9   Pages: 1127-1132   DOI: 10.1001/jama.269.9.1127   Published: MAR 3 1993
Physical Exam
Slide 7 of 8
Contents

## Slide 12
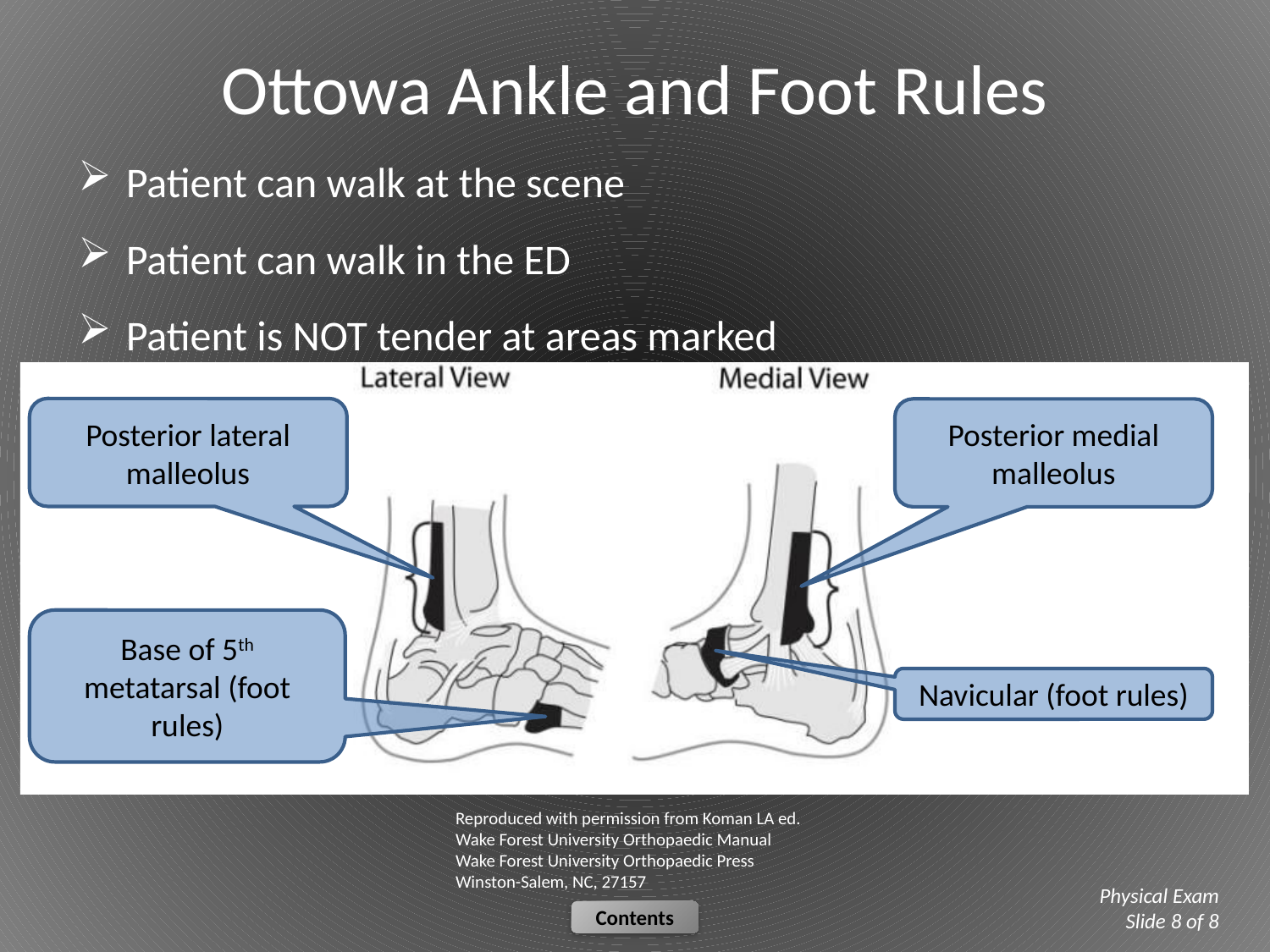

Ottowa Ankle and Foot Rules
Patient can walk at the scene
Patient can walk in the ED
Patient is NOT tender at areas marked
Posterior lateral malleolus
Posterior medial malleolus
Base of 5th metatarsal (foot rules)
Navicular (foot rules)
Reproduced with permission from Koman LA ed.
Wake Forest University Orthopaedic Manual
Wake Forest University Orthopaedic Press
Winston-Salem, NC, 27157
Physical Exam
Slide 8 of 8
Contents

## Slide 13
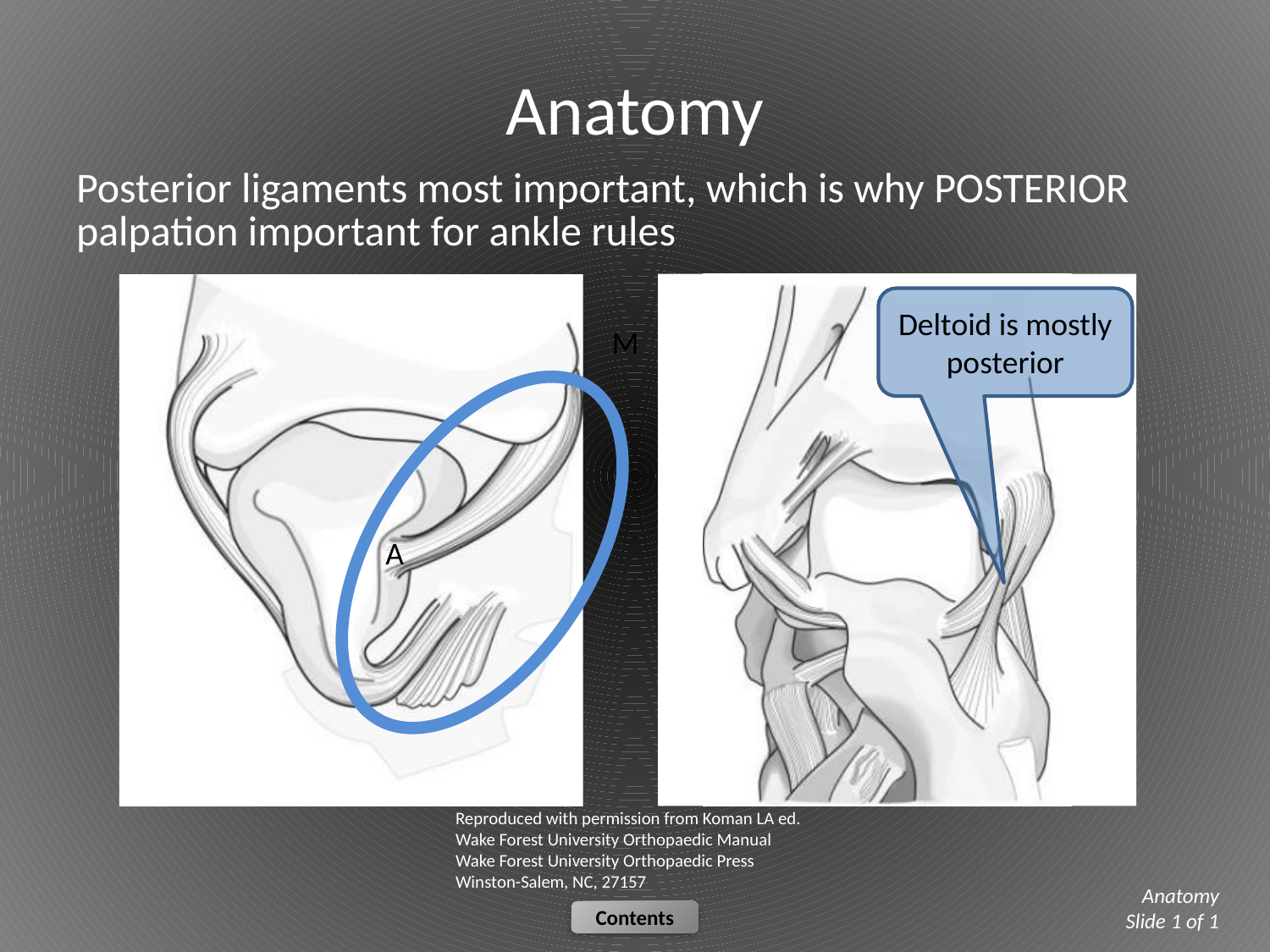

# Anatomy
Posterior ligaments most important, which is why POSTERIOR palpation important for ankle rules
Deltoid is mostly posterior
M
A
P
Reproduced with permission from Koman LA ed.
Wake Forest University Orthopaedic Manual
Wake Forest University Orthopaedic Press
Winston-Salem, NC, 27157
Anatomy
Slide 1 of 1
Contents

## Slide 14
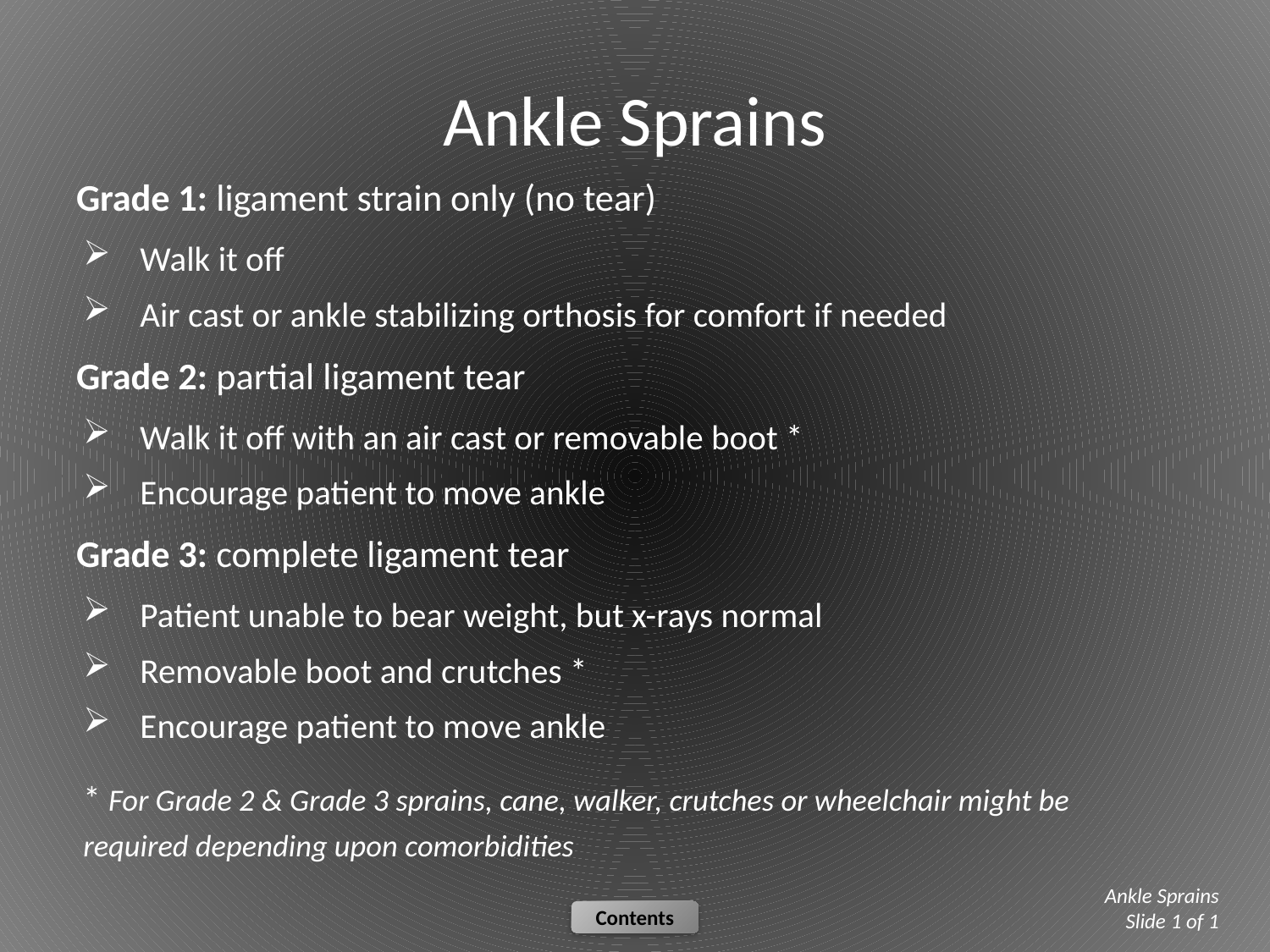

# Ankle Sprains
Grade 1: ligament strain only (no tear)
Walk it off
Air cast or ankle stabilizing orthosis for comfort if needed
Grade 2: partial ligament tear
Walk it off with an air cast or removable boot *
Encourage patient to move ankle
Grade 3: complete ligament tear
Patient unable to bear weight, but x-rays normal
Removable boot and crutches *
Encourage patient to move ankle
* For Grade 2 & Grade 3 sprains, cane, walker, crutches or wheelchair might be required depending upon comorbidities
Ankle Sprains
Slide 1 of 1
Contents

## Slide 15
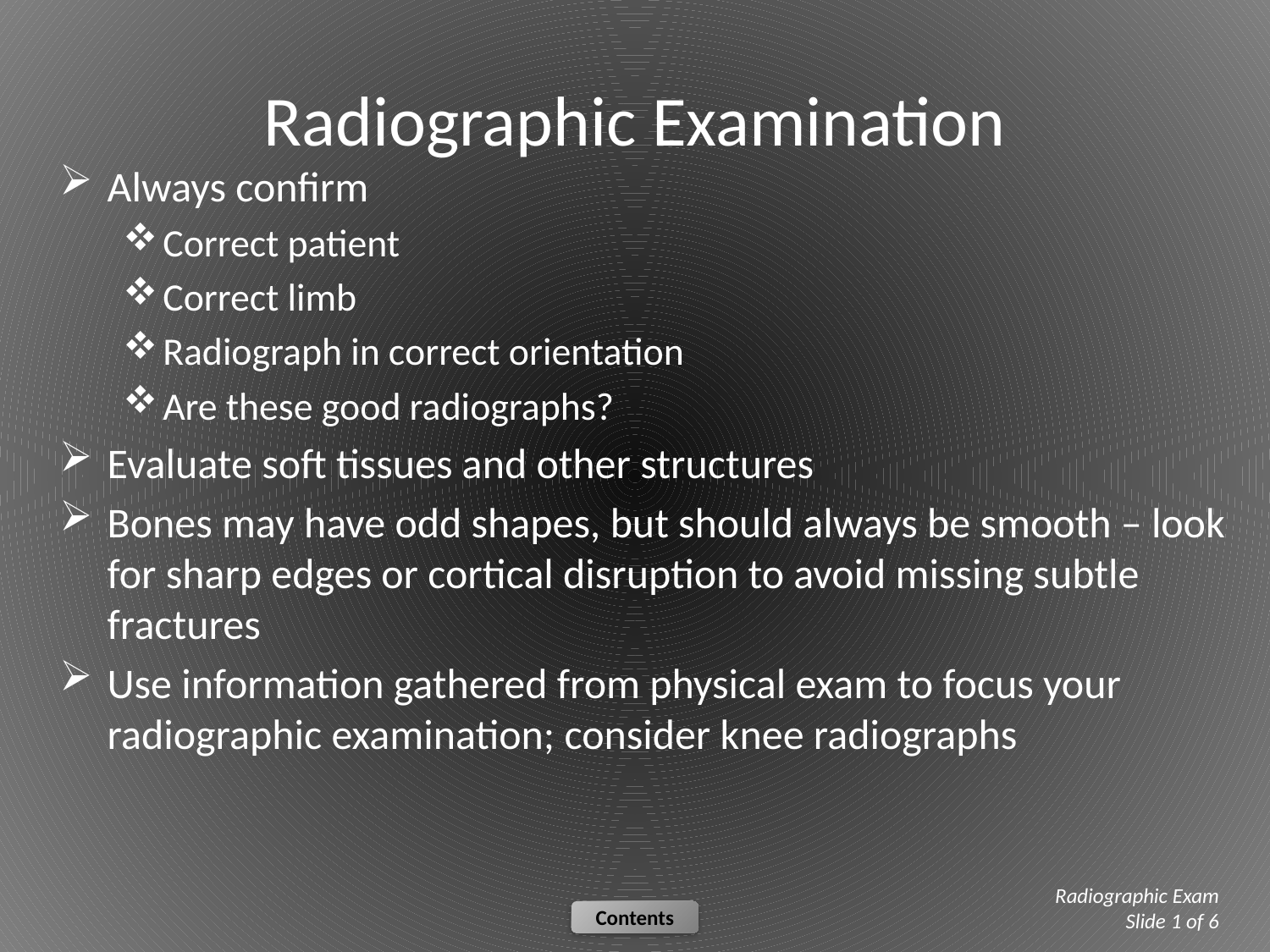

# Radiographic Examination
Always confirm
Correct patient
Correct limb
Radiograph in correct orientation
Are these good radiographs?
Evaluate soft tissues and other structures
Bones may have odd shapes, but should always be smooth – look for sharp edges or cortical disruption to avoid missing subtle fractures
Use information gathered from physical exam to focus your radiographic examination; consider knee radiographs
Radiographic Exam
Slide 1 of 6
Contents

## Slide 16
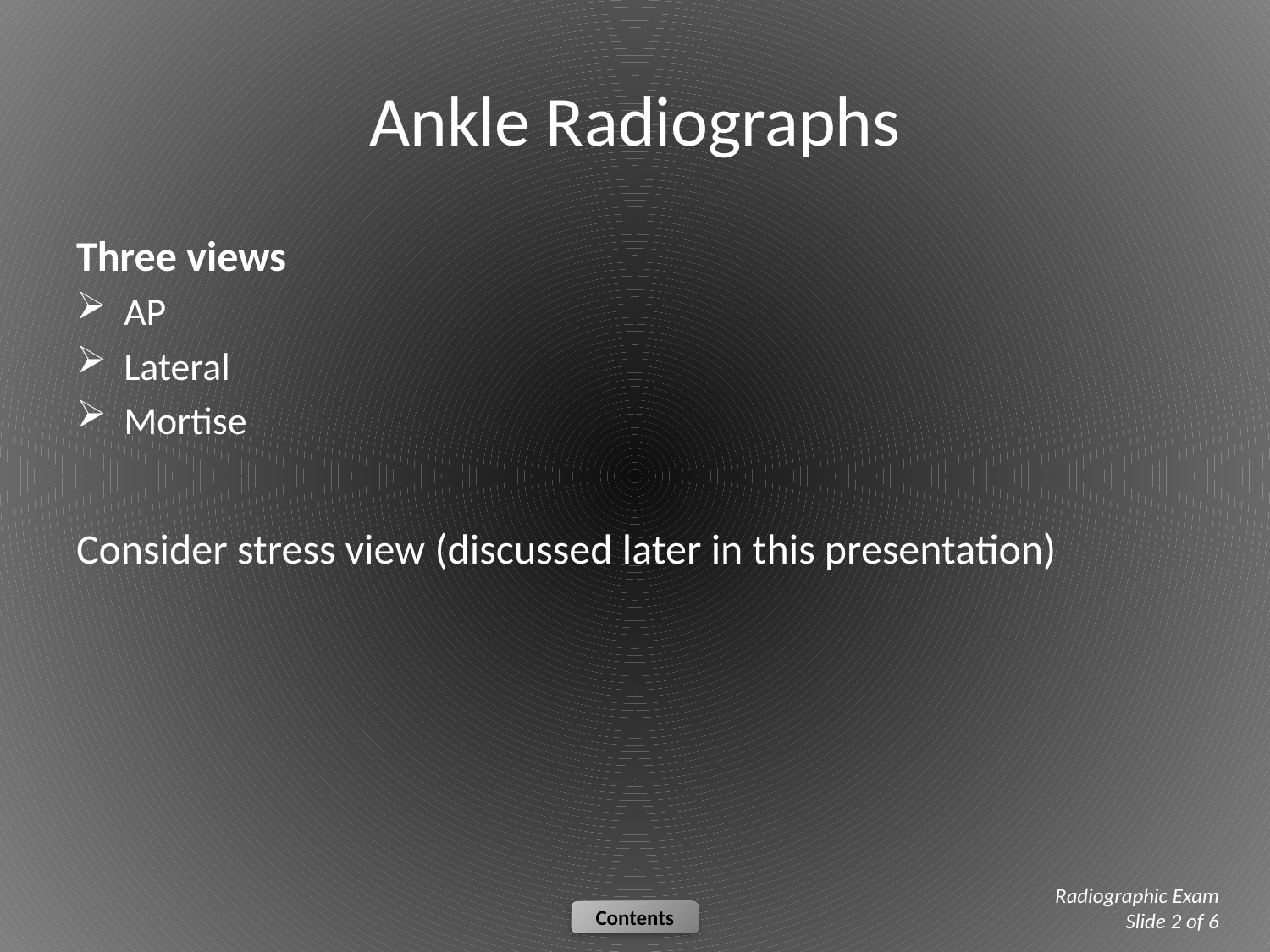

# Ankle Radiographs
Three views
AP
Lateral
Mortise
Consider stress view (discussed later in this presentation)
Radiographic Exam
Slide 2 of 6
Contents

## Slide 17
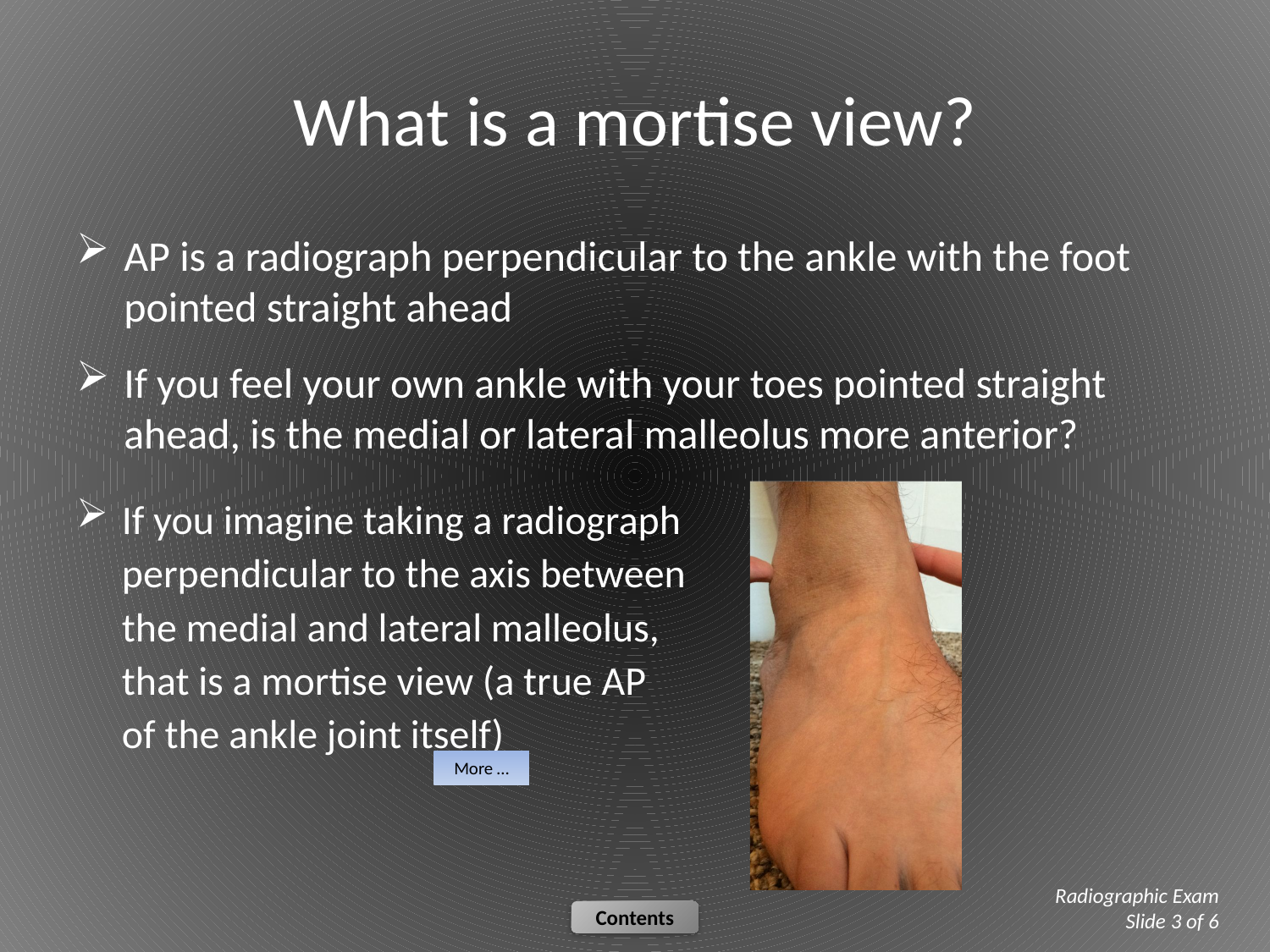

# What is a mortise view?
AP is a radiograph perpendicular to the ankle with the foot pointed straight ahead
If you feel your own ankle with your toes pointed straight ahead, is the medial or lateral malleolus more anterior?
If you imagine taking a radiograph perpendicular to the axis between the medial and lateral malleolus, that is a mortise view (a true AP of the ankle joint itself)
More …
Radiographic Exam
Slide 3 of 6
Contents

## Slide 18
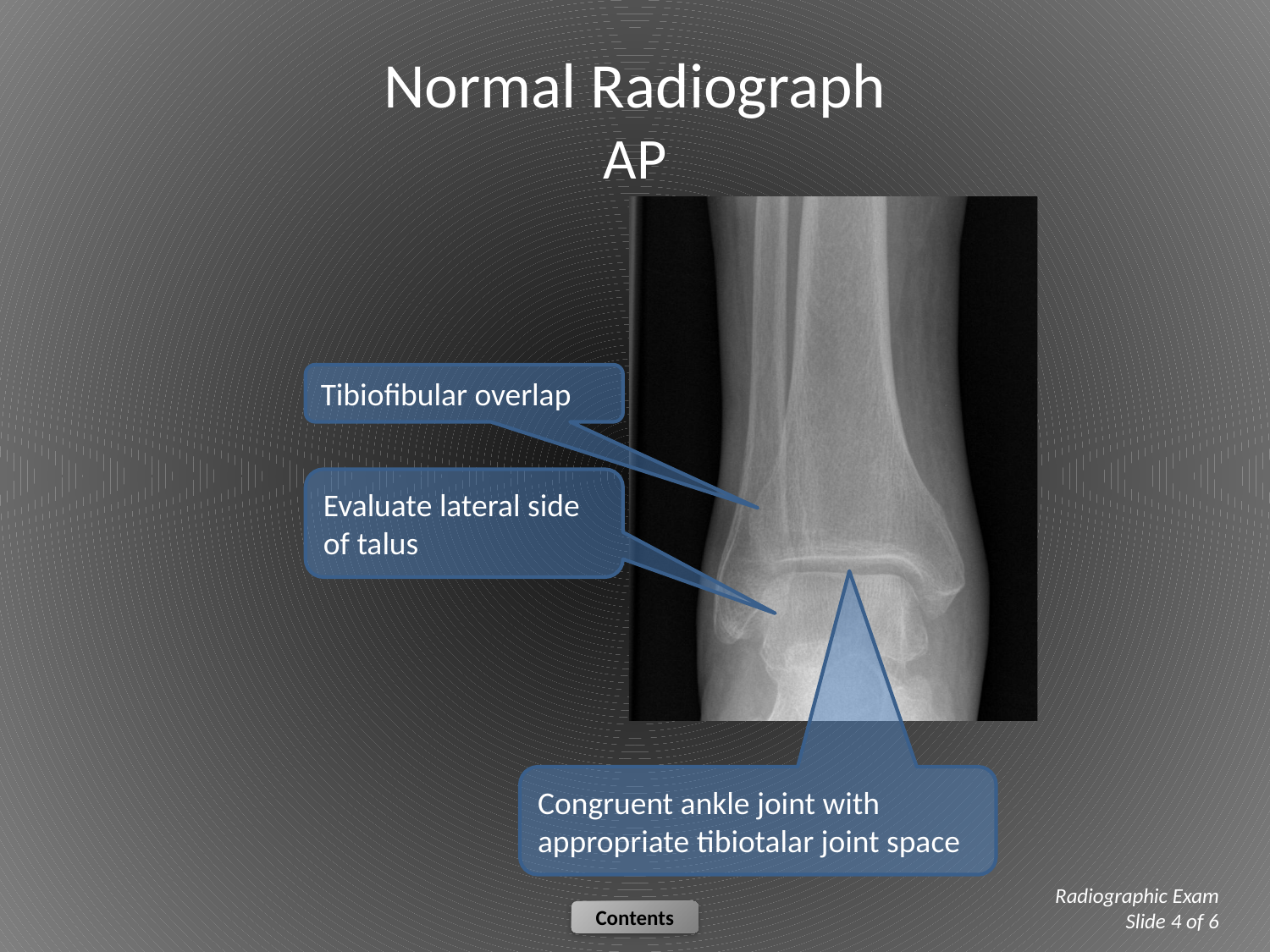

# Normal Radiograph AP
Tibiofibular overlap
Evaluate lateral side of talus
Congruent ankle joint with appropriate tibiotalar joint space
Radiographic Exam
Slide 4 of 6
Contents

## Slide 19
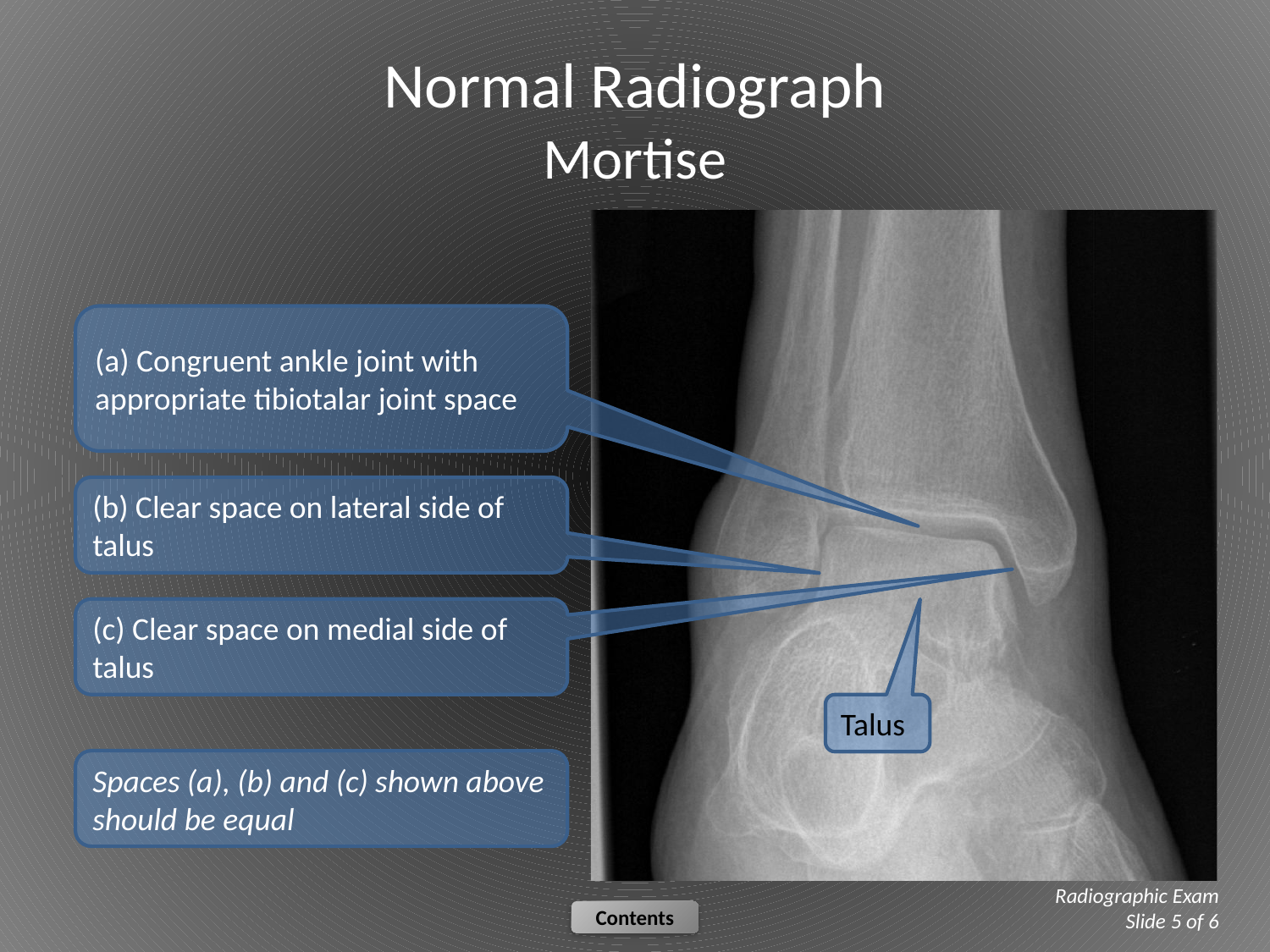

# Normal Radiograph Mortise
(a) Congruent ankle joint with appropriate tibiotalar joint space
(b) Clear space on lateral side of talus
(c) Clear space on medial side of talus
Talus
Spaces (a), (b) and (c) shown above should be equal
Radiographic Exam
Slide 5 of 6
Contents

## Slide 20
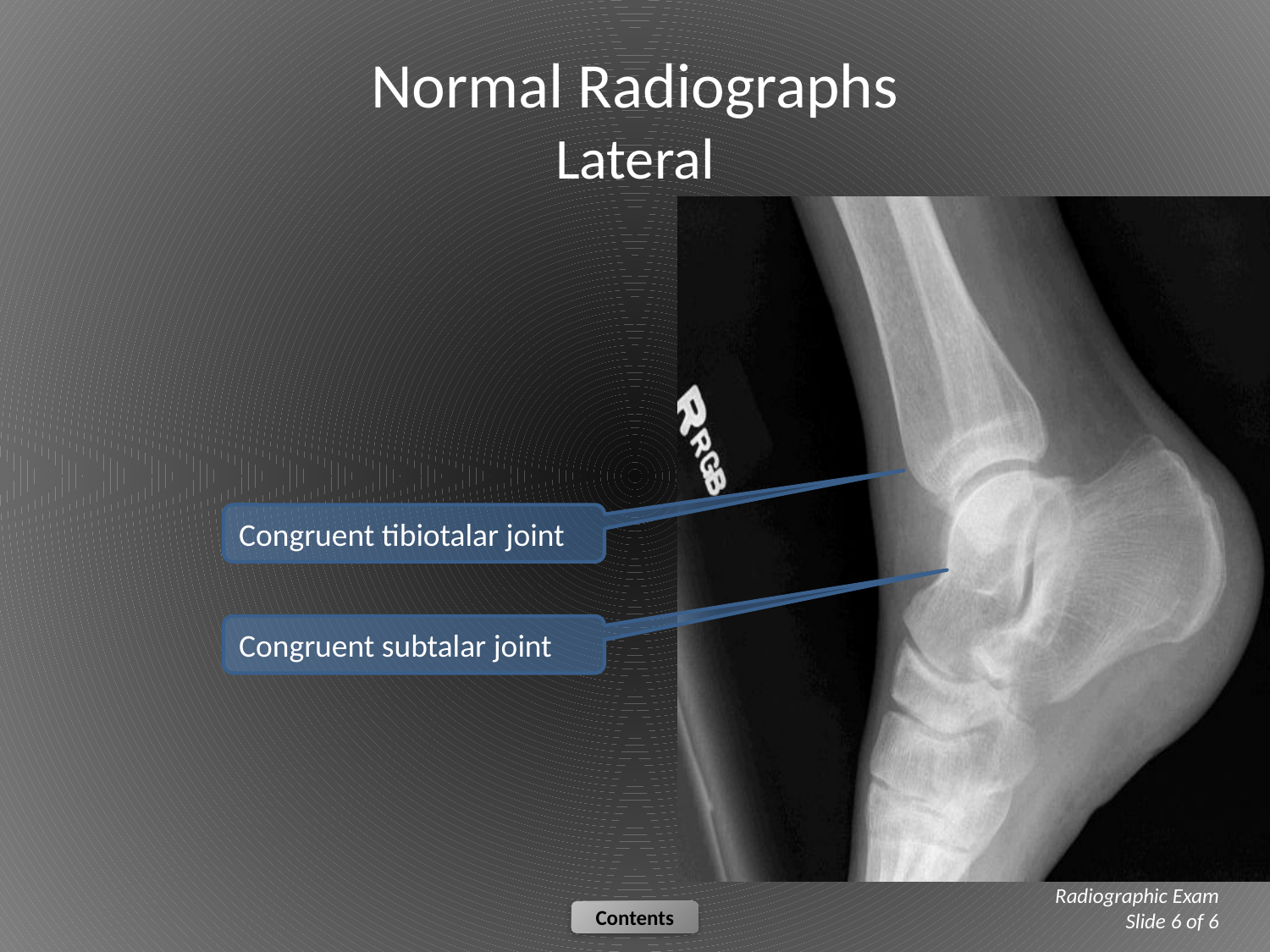

# Normal Radiographs Lateral
Congruent tibiotalar joint
Congruent subtalar joint
Radiographic Exam
Slide 6 of 6
Contents

## Slide 21
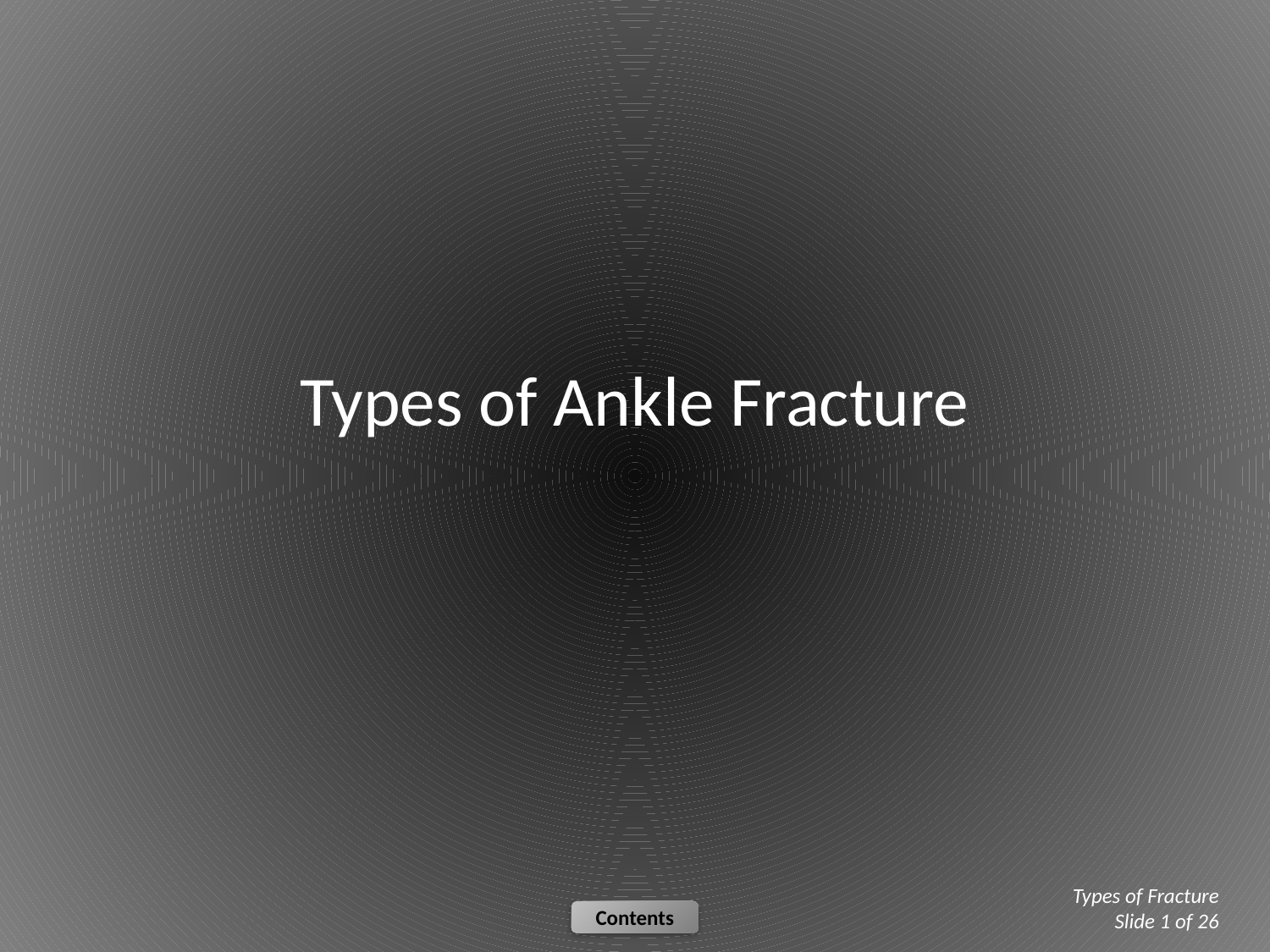

# Types of Ankle Fracture
Types of Fracture
Slide 1 of 26
Contents

## Slide 22
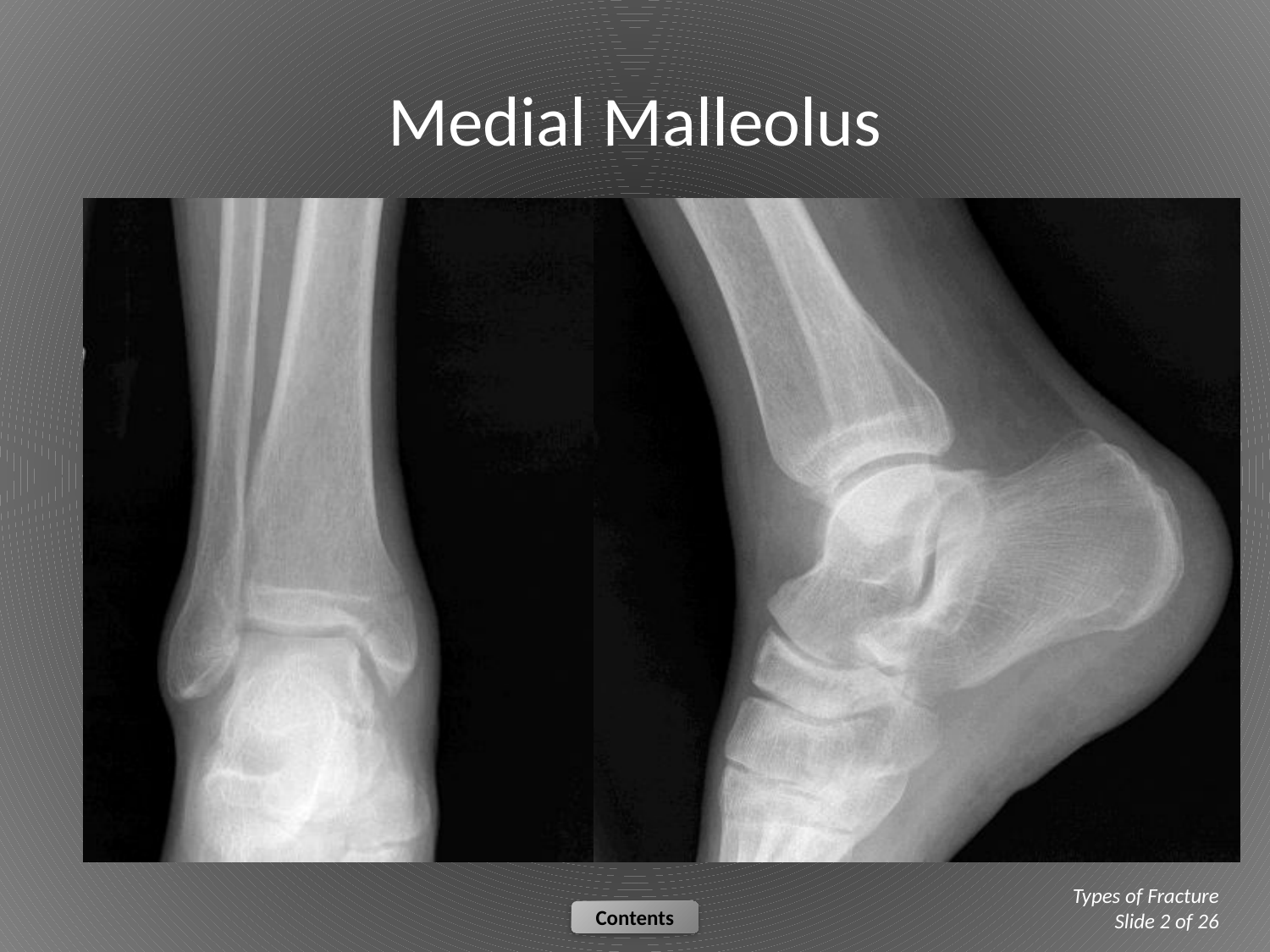

# Medial Malleolus
Types of Fracture
Slide 2 of 26
Contents

## Slide 23
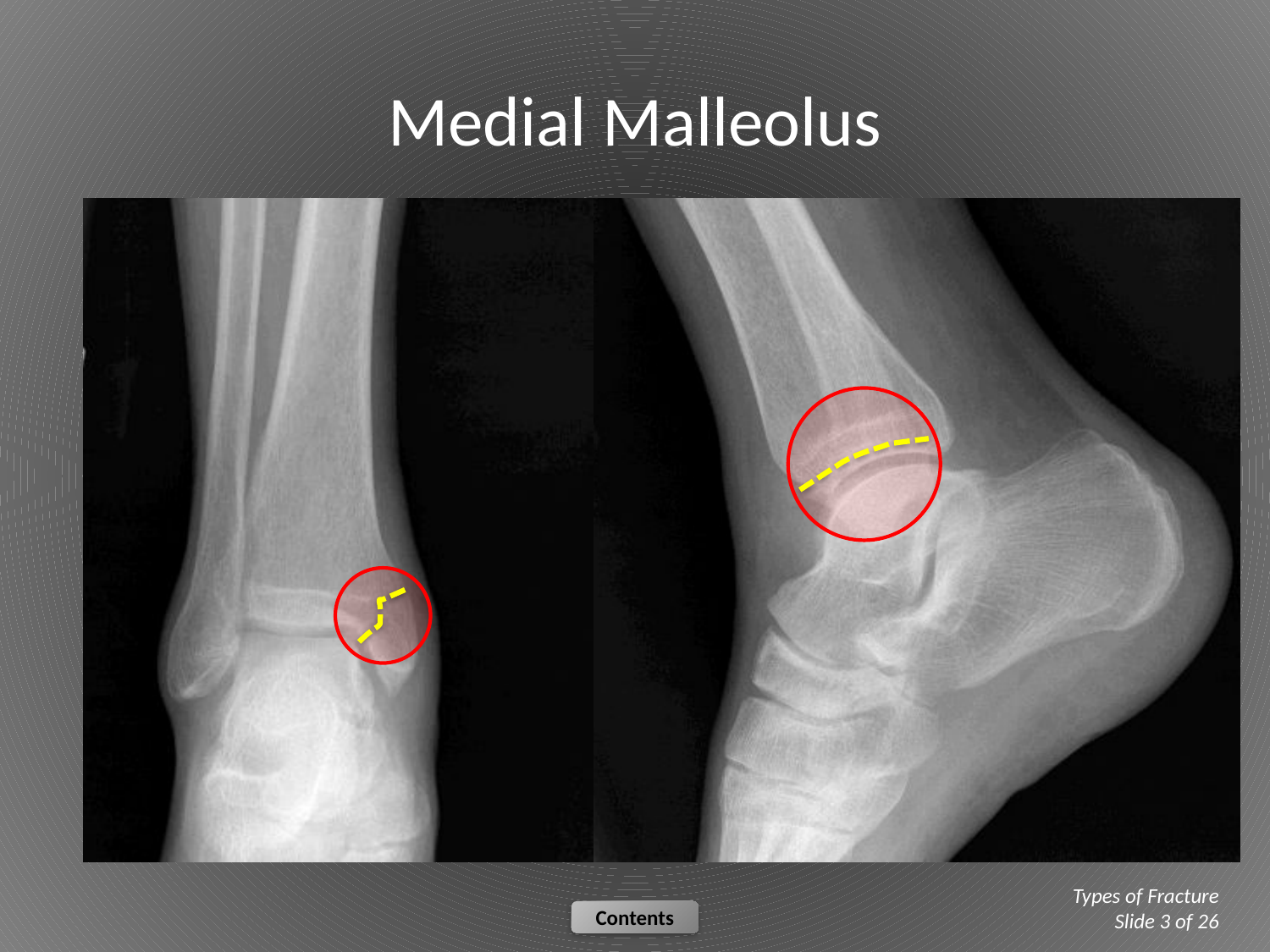

# Medial Malleolus
Types of Fracture
Slide 3 of 26
Contents

## Slide 24
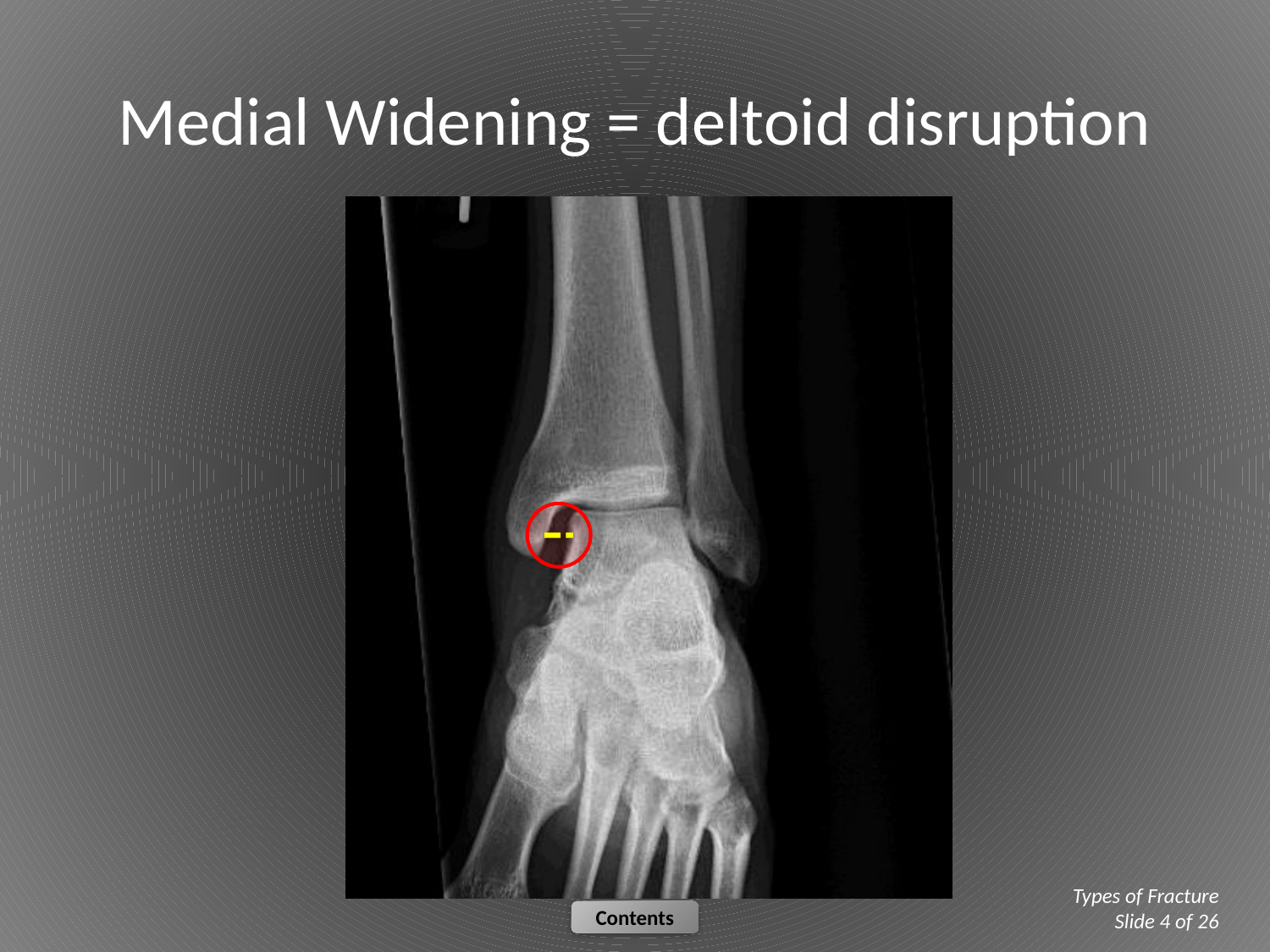

# Medial Widening = deltoid disruption
Types of Fracture
Slide 4 of 26
Contents

## Slide 25
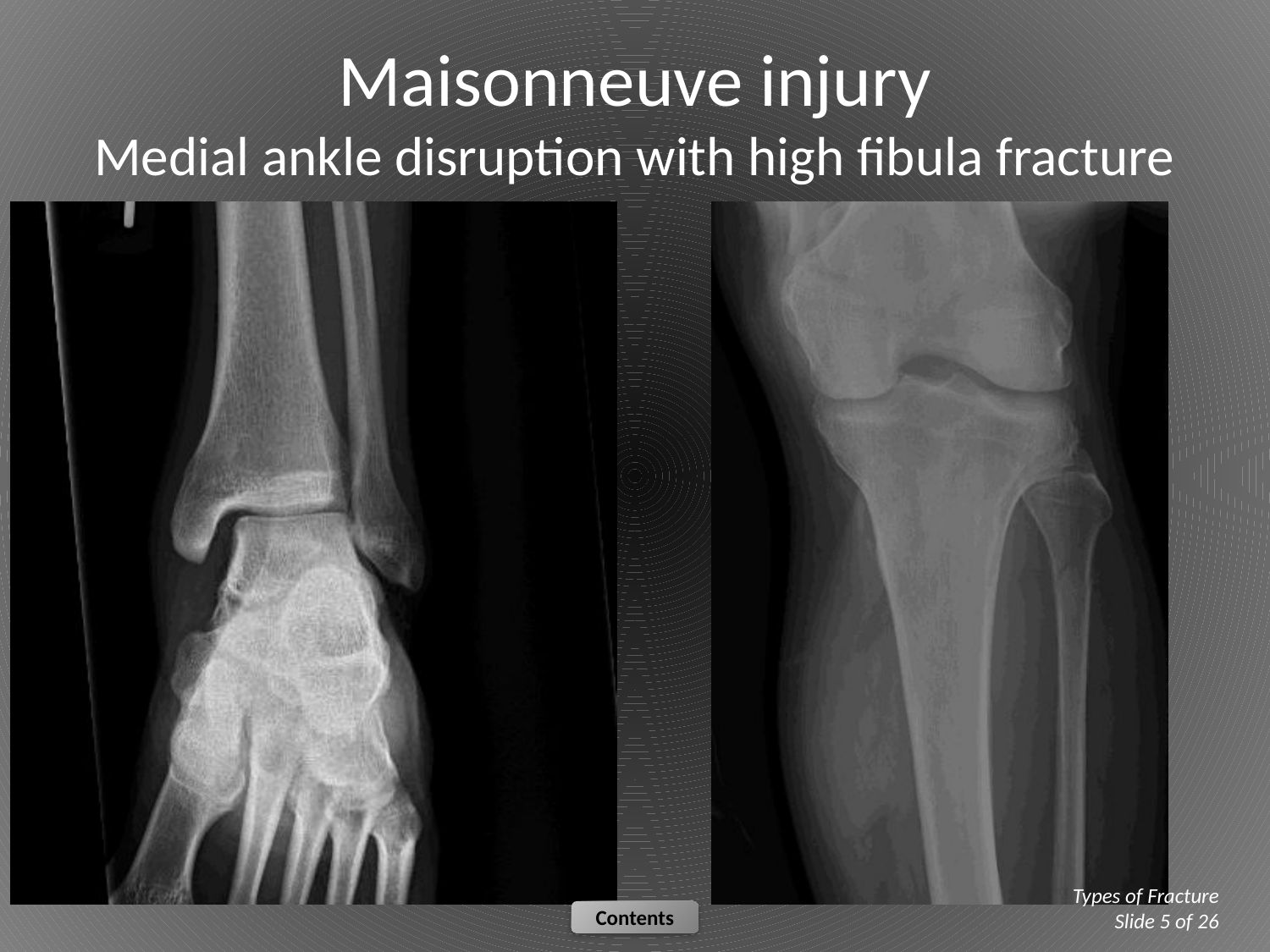

# Maisonneuve injury Medial ankle disruption with high fibula fracture
Types of Fracture
Slide 5 of 26
Contents

## Slide 26
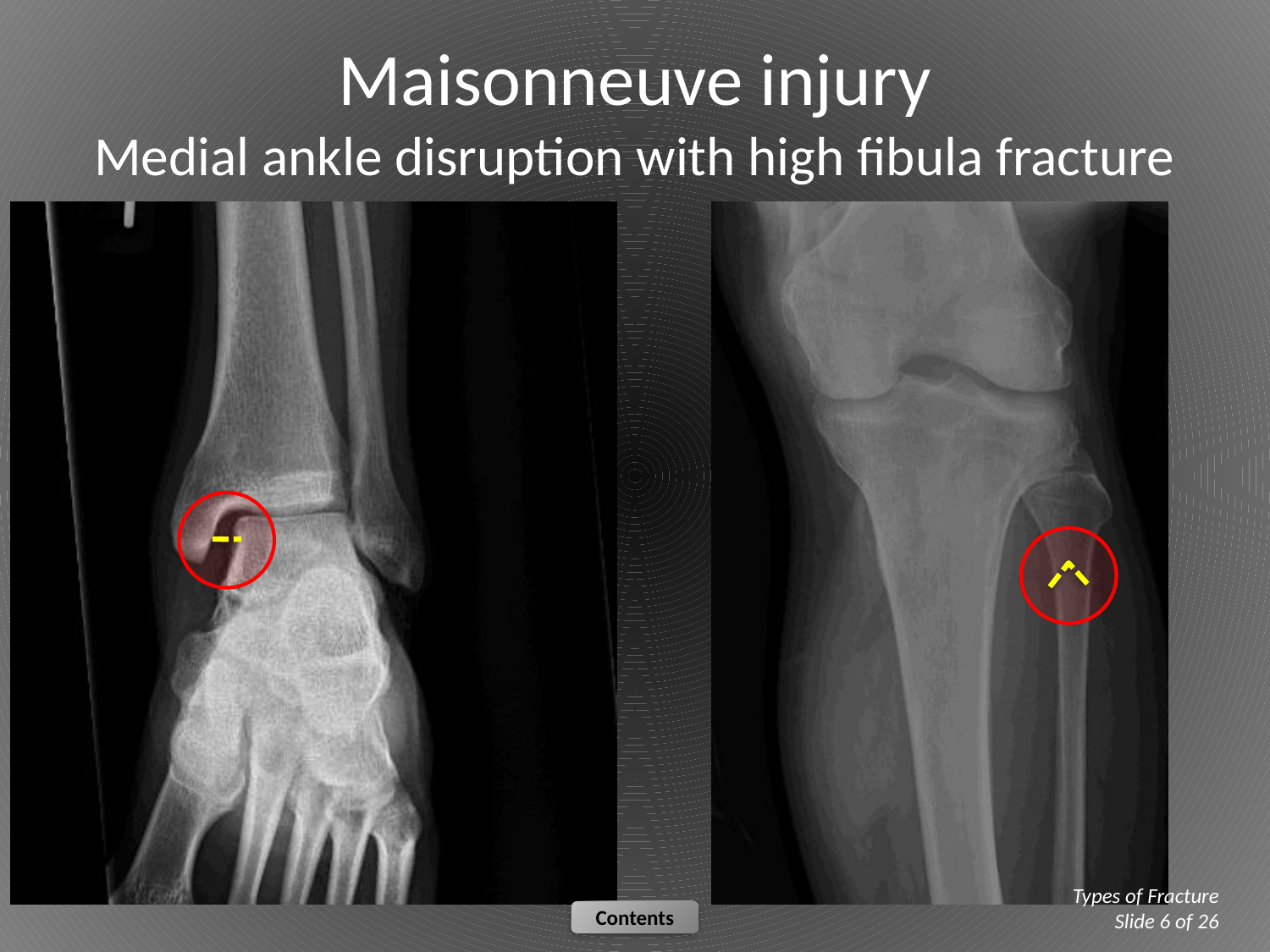

# Maisonneuve injury Medial ankle disruption with high fibula fracture
Types of Fracture
Slide 6 of 26
Contents

## Slide 27
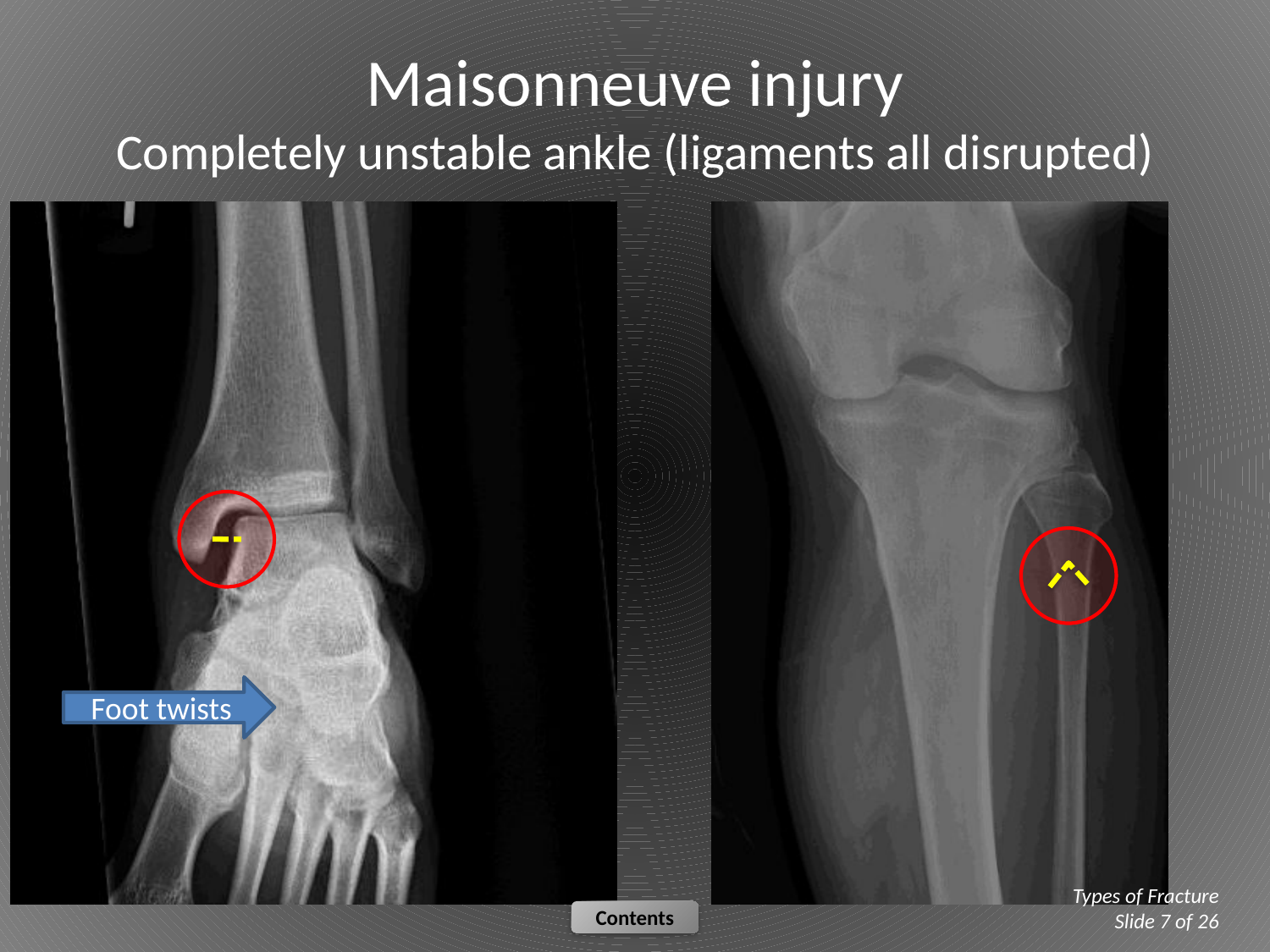

# Maisonneuve injury Completely unstable ankle (ligaments all disrupted)
Foot twists
Types of Fracture
Slide 7 of 26
Contents

## Slide 28
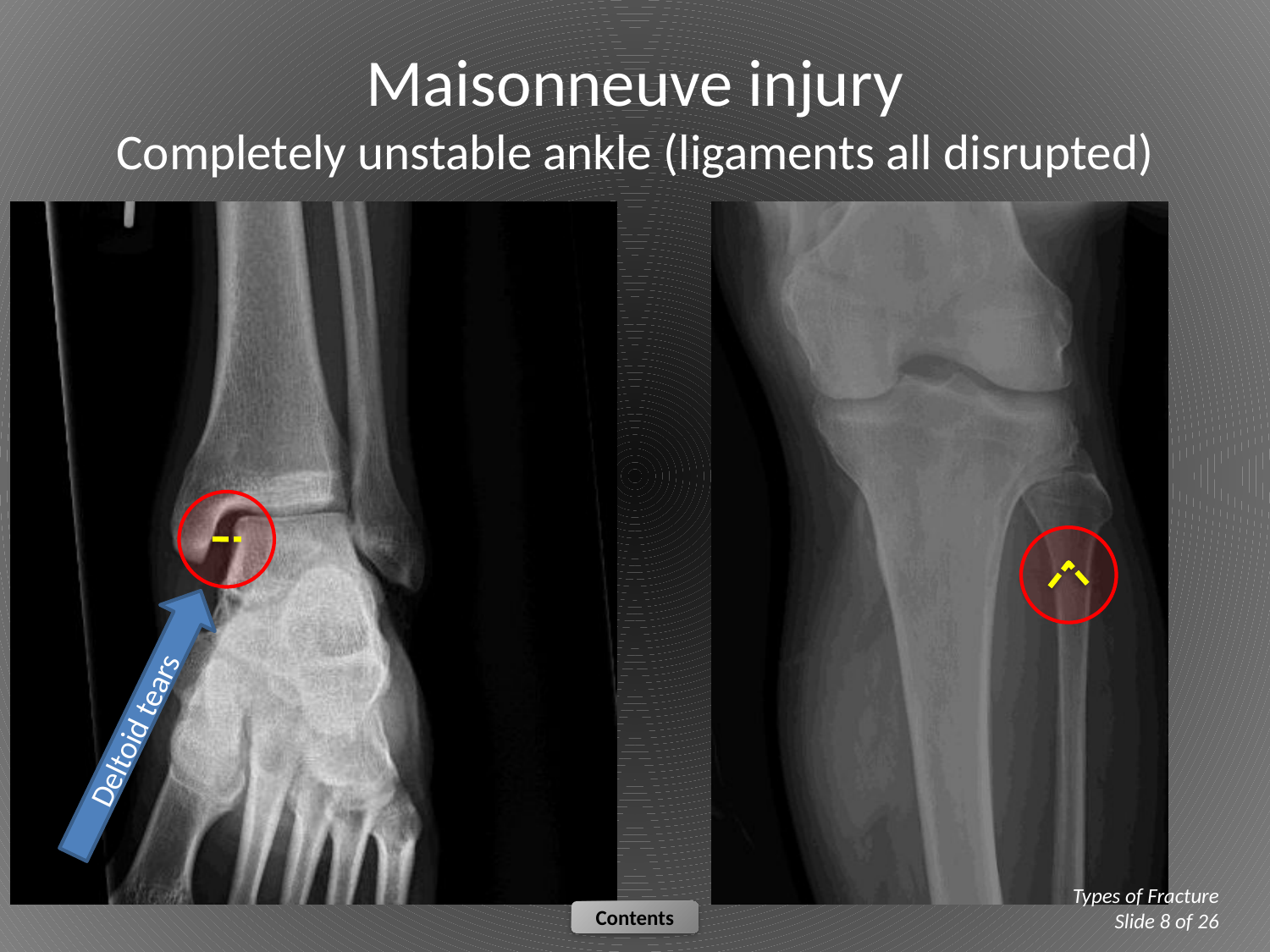

# Maisonneuve injury Completely unstable ankle (ligaments all disrupted)
Deltoid tears
Types of Fracture
Slide 8 of 26
Contents

## Slide 29
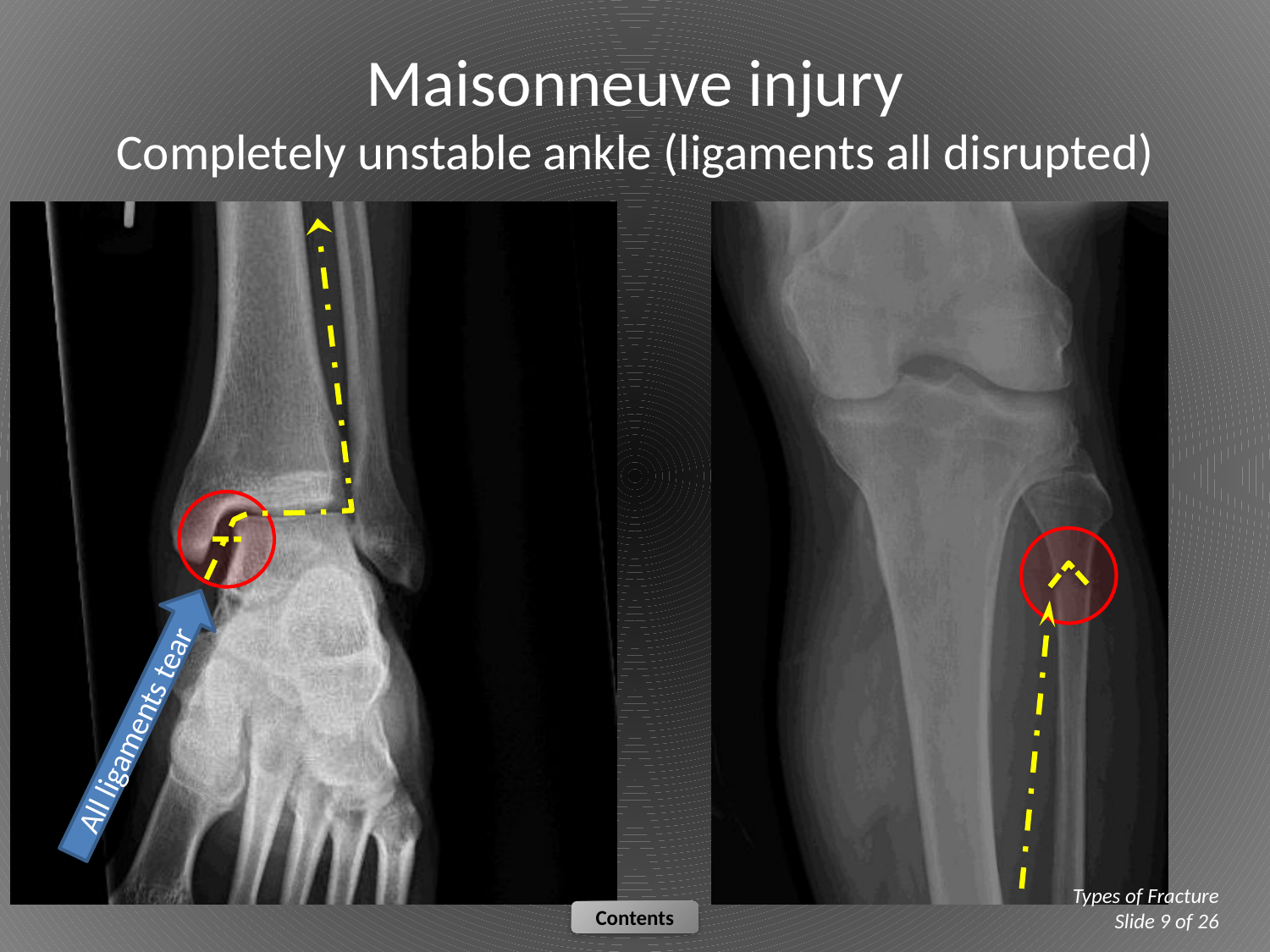

# Maisonneuve injury Completely unstable ankle (ligaments all disrupted)
All ligaments tear
Types of Fracture
Slide 9 of 26
Contents

## Slide 30
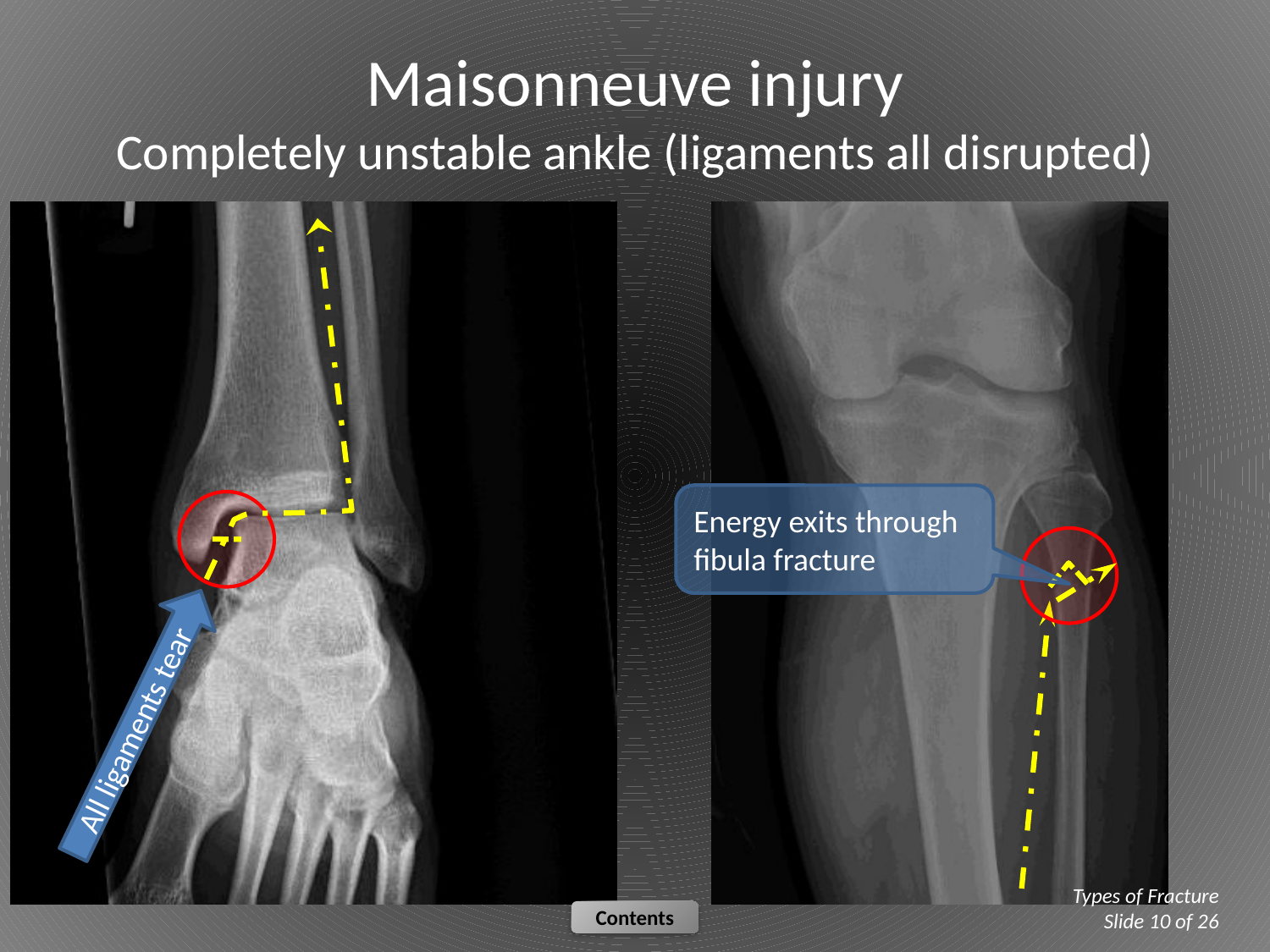

# Maisonneuve injury Completely unstable ankle (ligaments all disrupted)
Energy exits through fibula fracture
All ligaments tear
Types of Fracture
Slide 10 of 26
Contents

## Slide 31
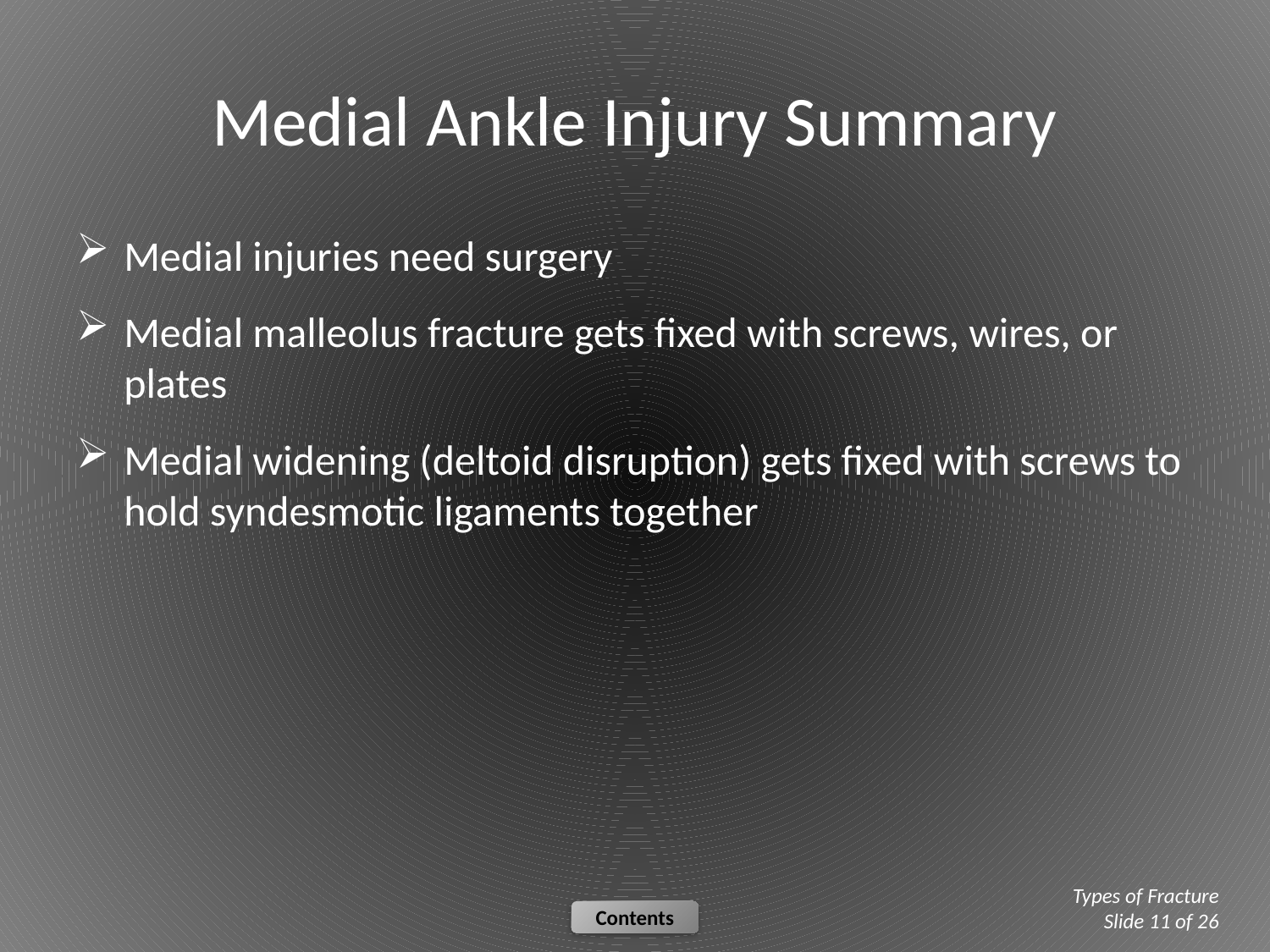

# Medial Ankle Injury Summary
Medial injuries need surgery
Medial malleolus fracture gets fixed with screws, wires, or plates
Medial widening (deltoid disruption) gets fixed with screws to hold syndesmotic ligaments together
Types of Fracture
Slide 11 of 26
Contents

## Slide 32
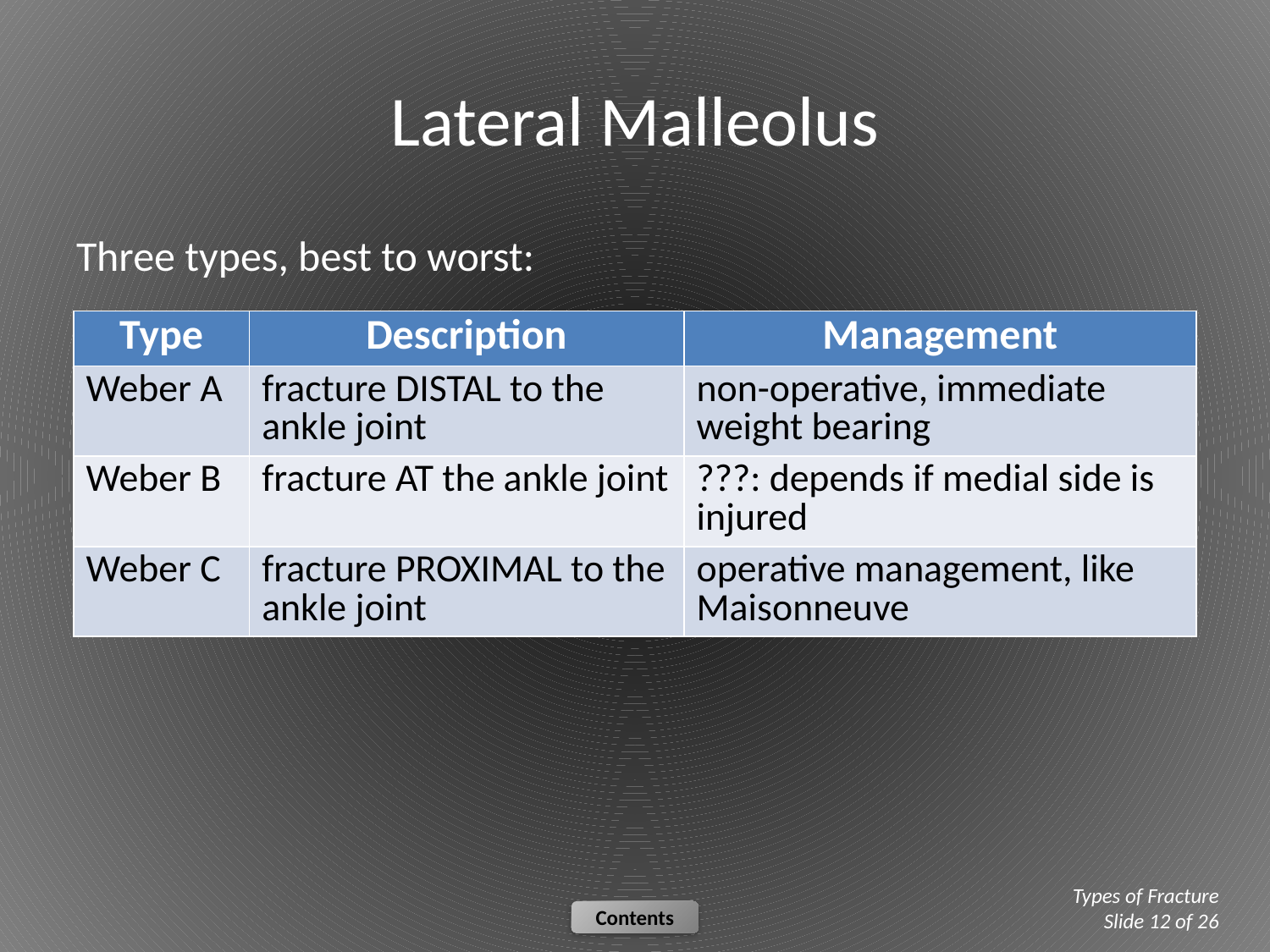

# Lateral Malleolus
Three types, best to worst:
| Type | Description | Management |
| --- | --- | --- |
| Weber A | fracture DISTAL to the ankle joint | non-operative, immediate weight bearing |
| Weber B | fracture AT the ankle joint | ???: depends if medial side is injured |
| Weber C | fracture PROXIMAL to the ankle joint | operative management, like Maisonneuve |
Types of Fracture
Slide 12 of 26
Contents

## Slide 33
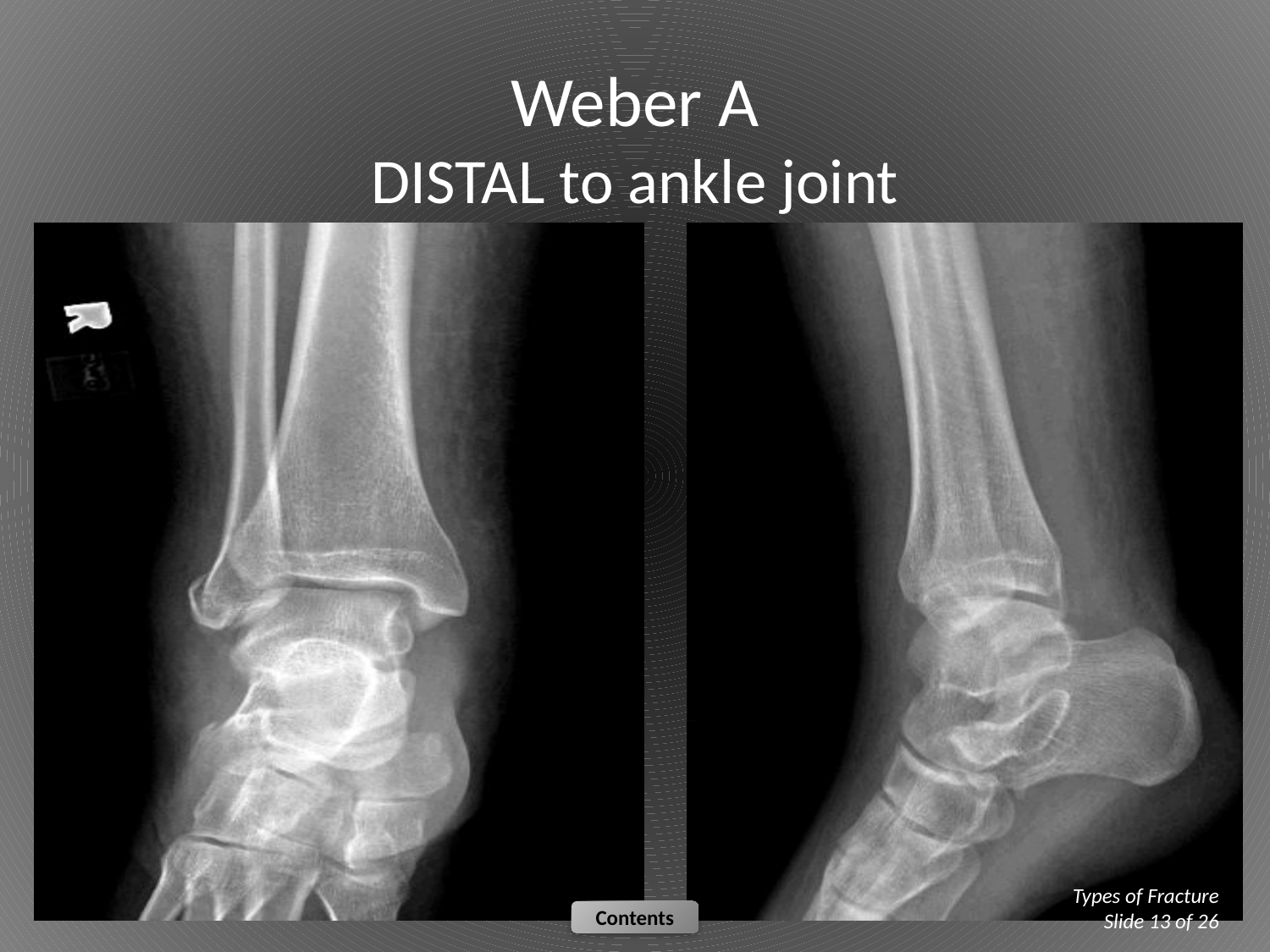

# Weber A DISTAL to ankle joint
Types of Fracture
Slide 13 of 26
Contents

## Slide 34
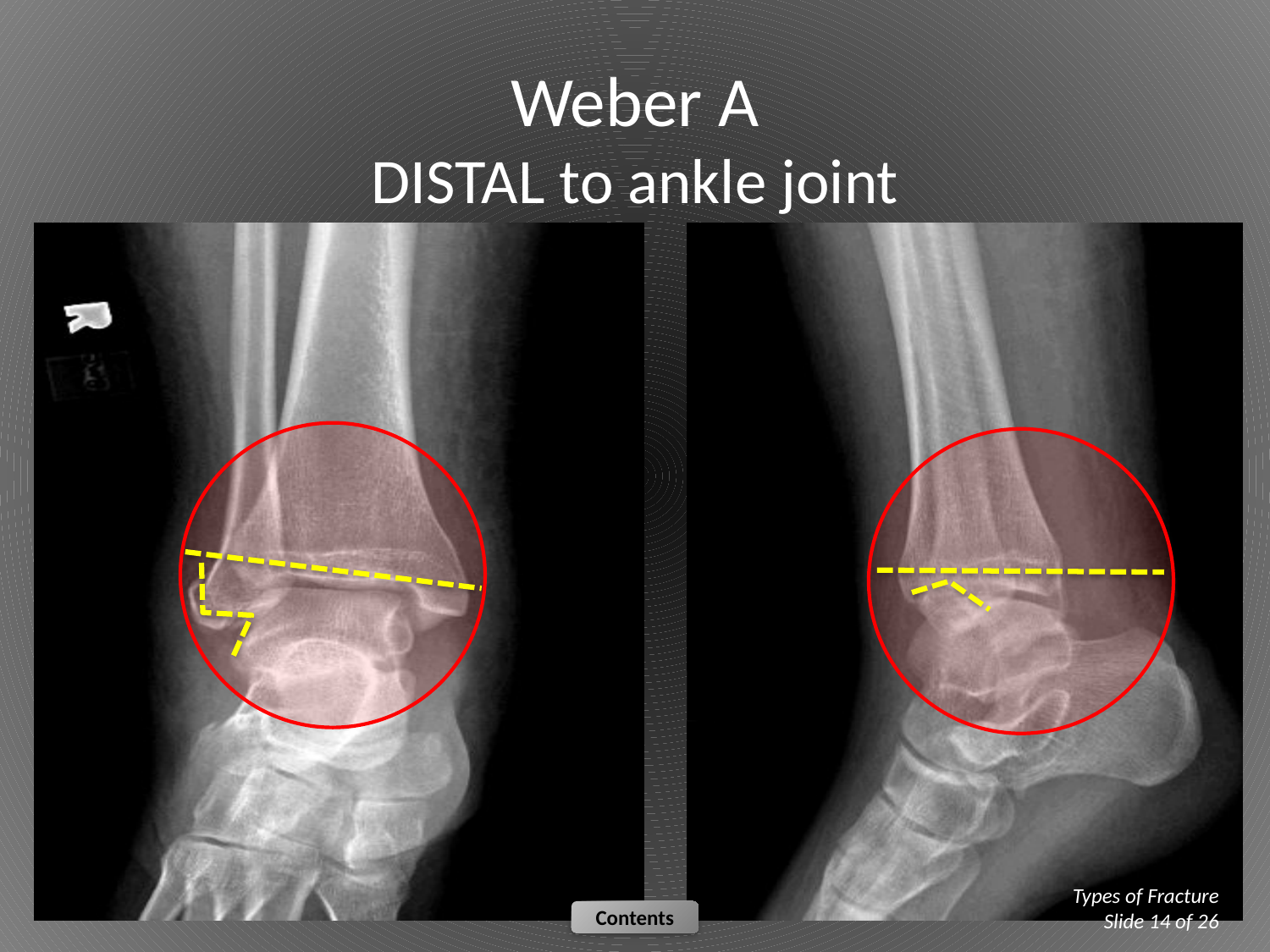

# Weber A DISTAL to ankle joint
Types of Fracture
Slide 14 of 26
Contents

## Slide 35
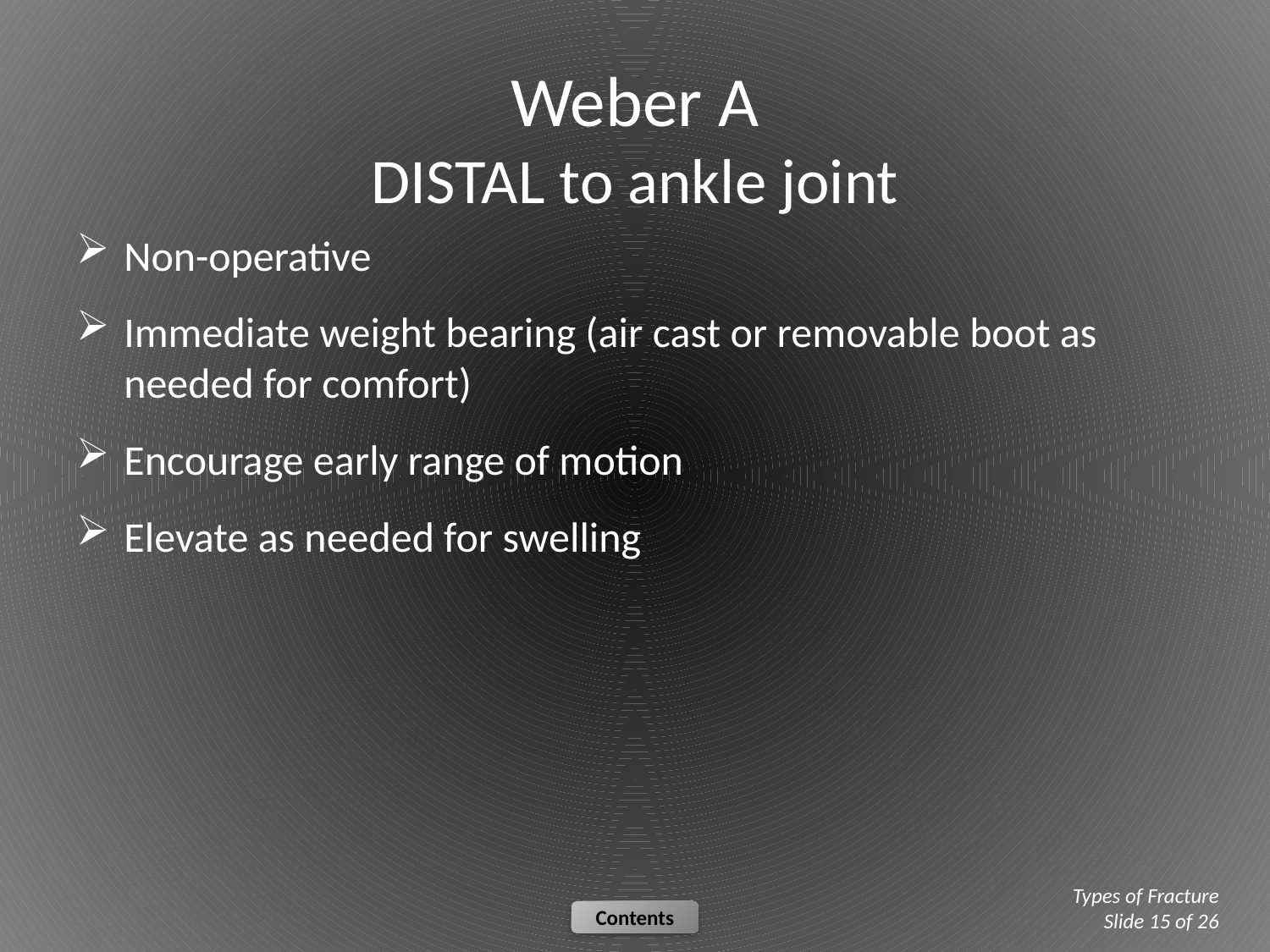

# Weber A DISTAL to ankle joint
Non-operative
Immediate weight bearing (air cast or removable boot as needed for comfort)
Encourage early range of motion
Elevate as needed for swelling
Types of Fracture
Slide 15 of 26
Contents

## Slide 36
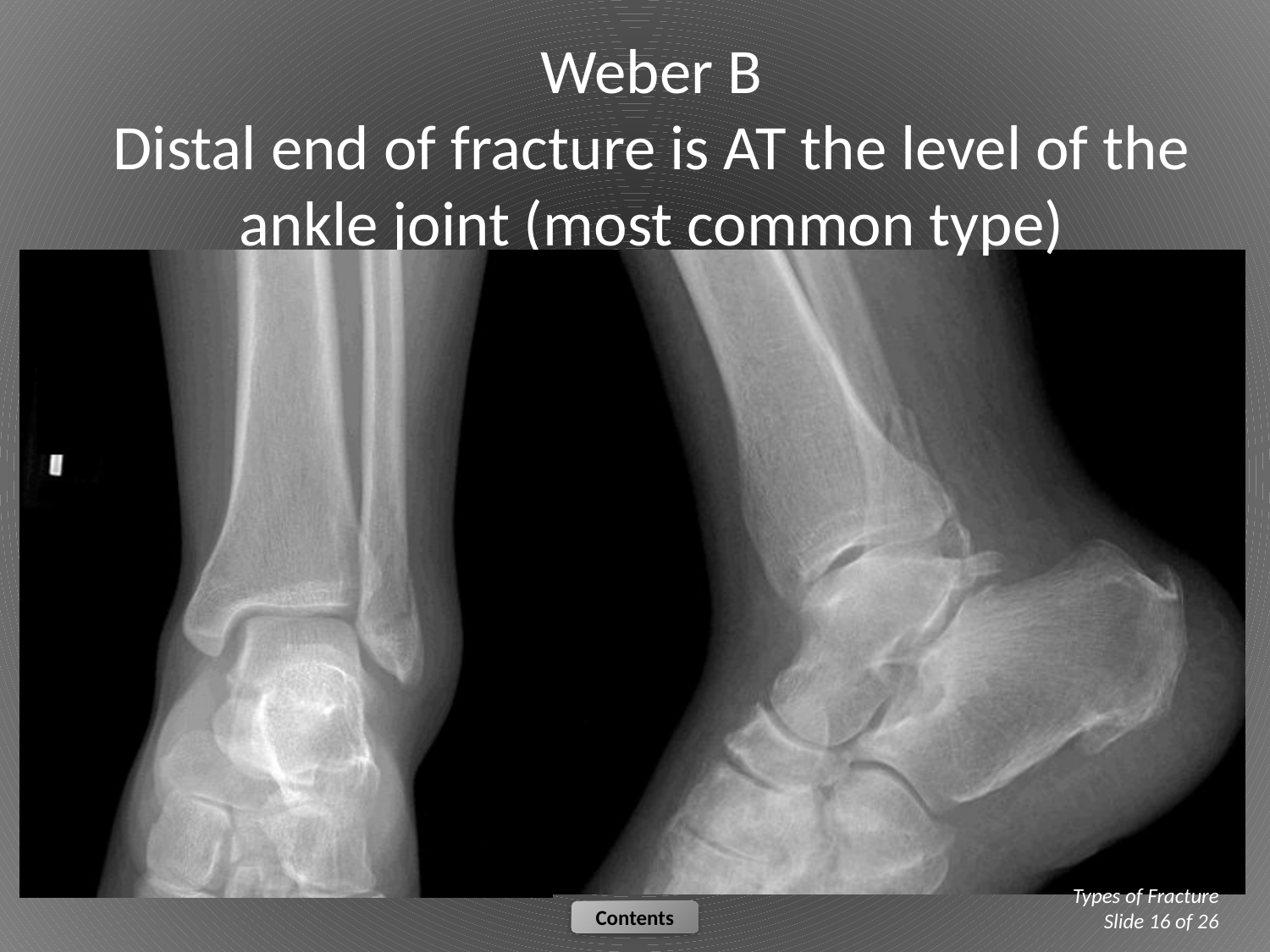

# Weber B Distal end of fracture is AT the level of the ankle joint (most common type)
Types of Fracture
Slide 16 of 26
Contents

## Slide 37
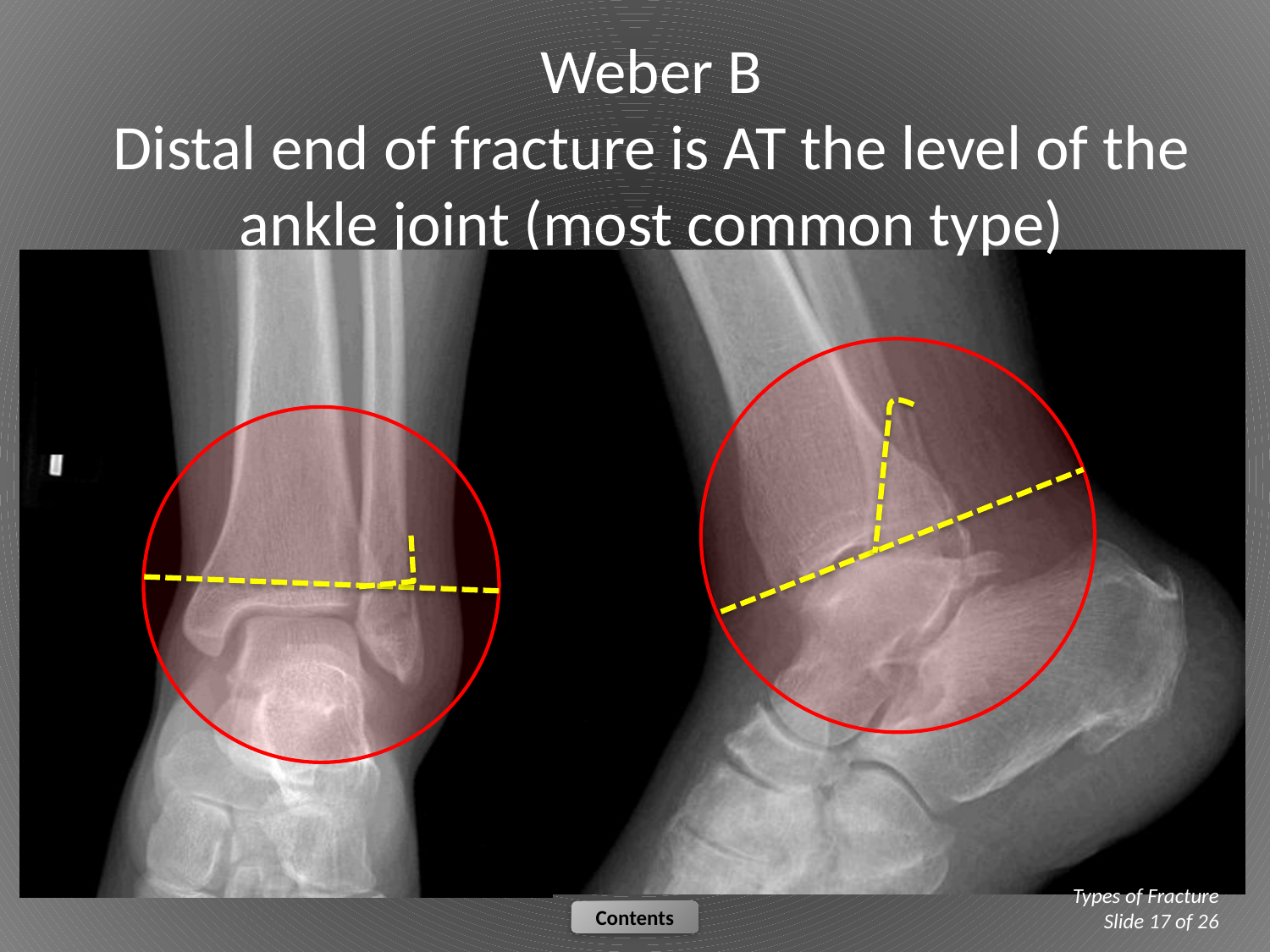

# Weber B Distal end of fracture is AT the level of the ankle joint (most common type)
Types of Fracture
Slide 17 of 26
Contents

## Slide 38
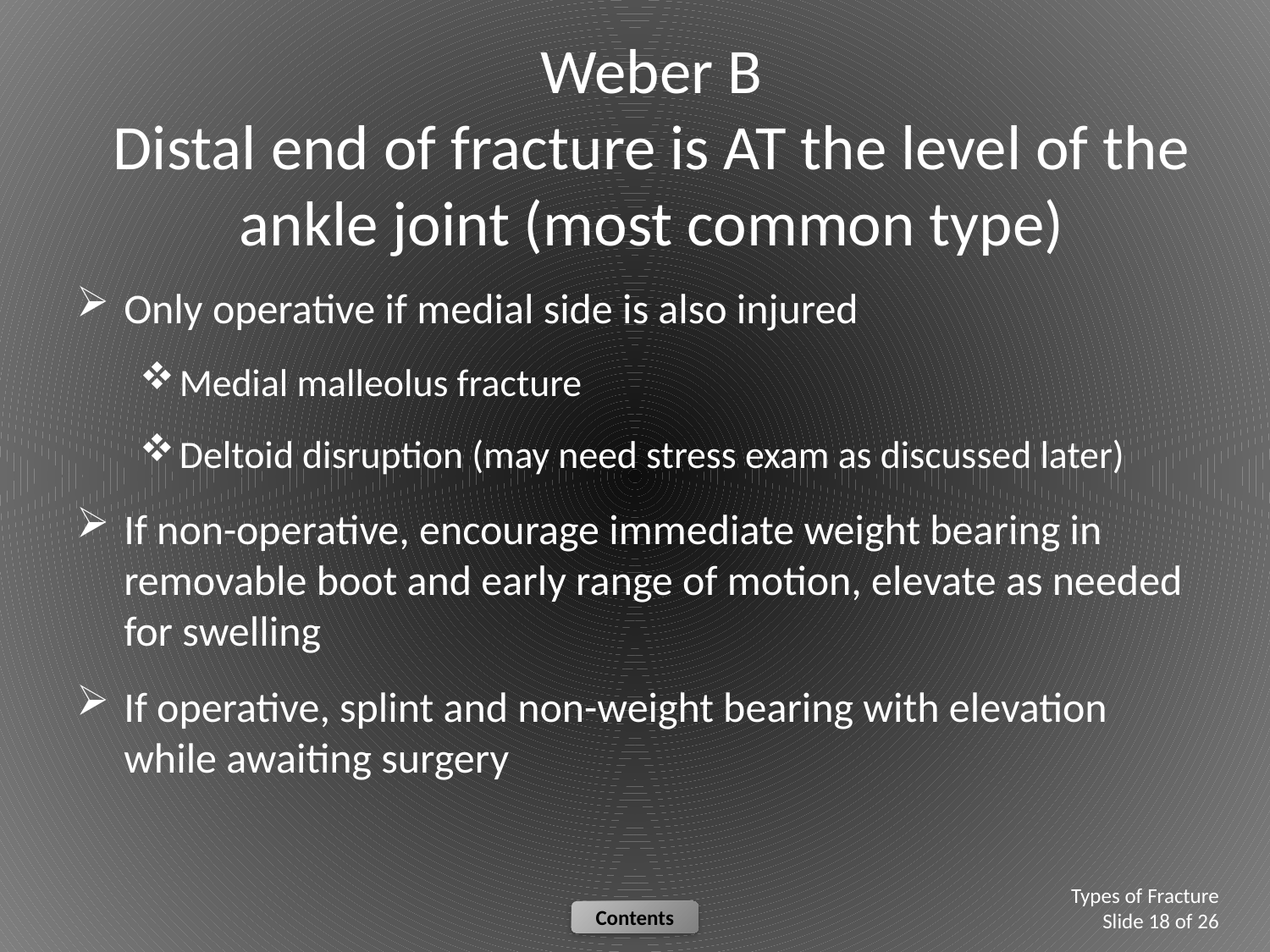

# Weber B Distal end of fracture is AT the level of the ankle joint (most common type)
Only operative if medial side is also injured
Medial malleolus fracture
Deltoid disruption (may need stress exam as discussed later)
If non-operative, encourage immediate weight bearing in removable boot and early range of motion, elevate as needed for swelling
If operative, splint and non-weight bearing with elevation while awaiting surgery
Types of Fracture
Slide 18 of 26
Contents

## Slide 39
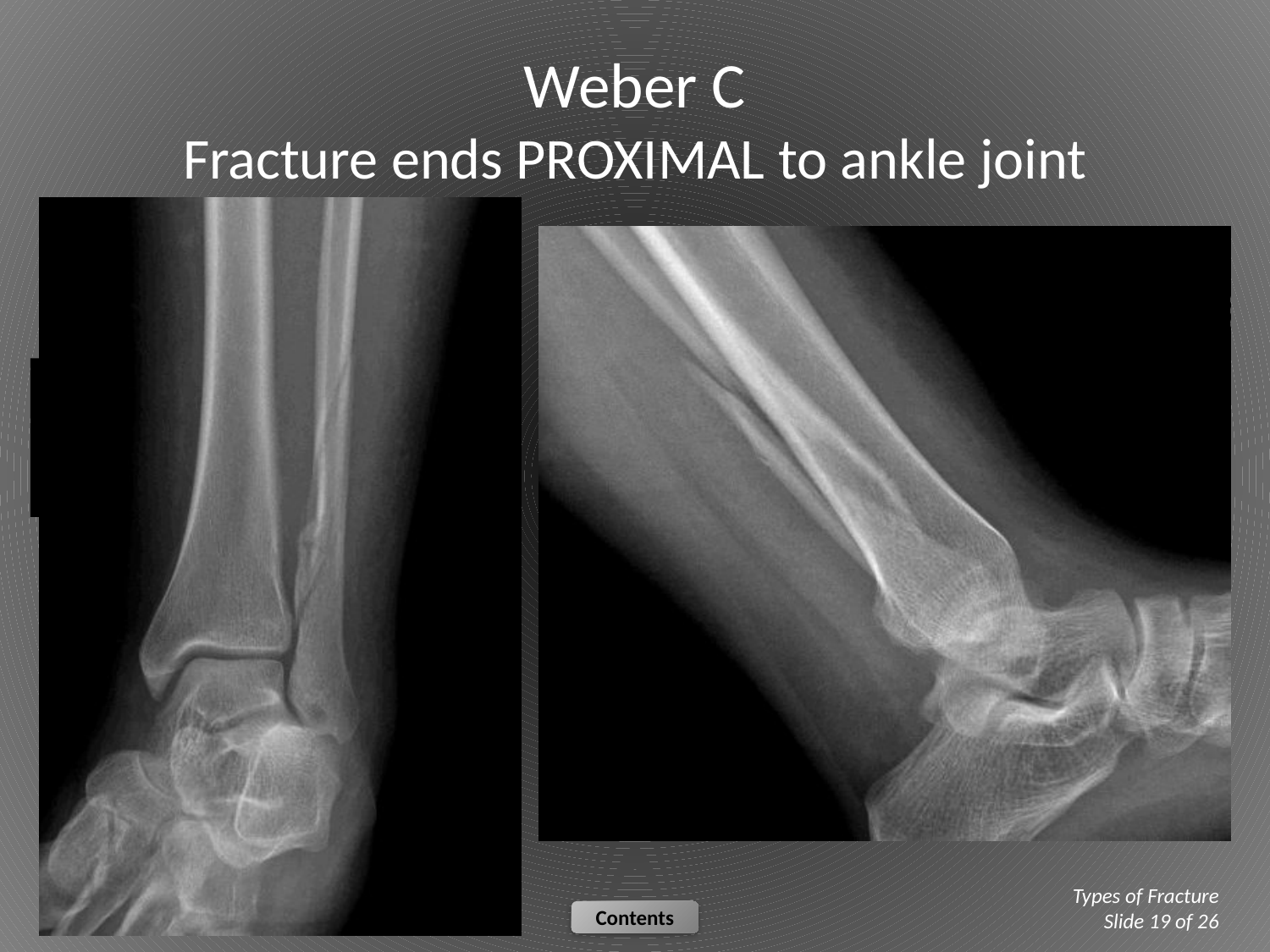

# Weber C Fracture ends PROXIMAL to ankle joint
Types of Fracture
Slide 19 of 26
Contents

## Slide 40
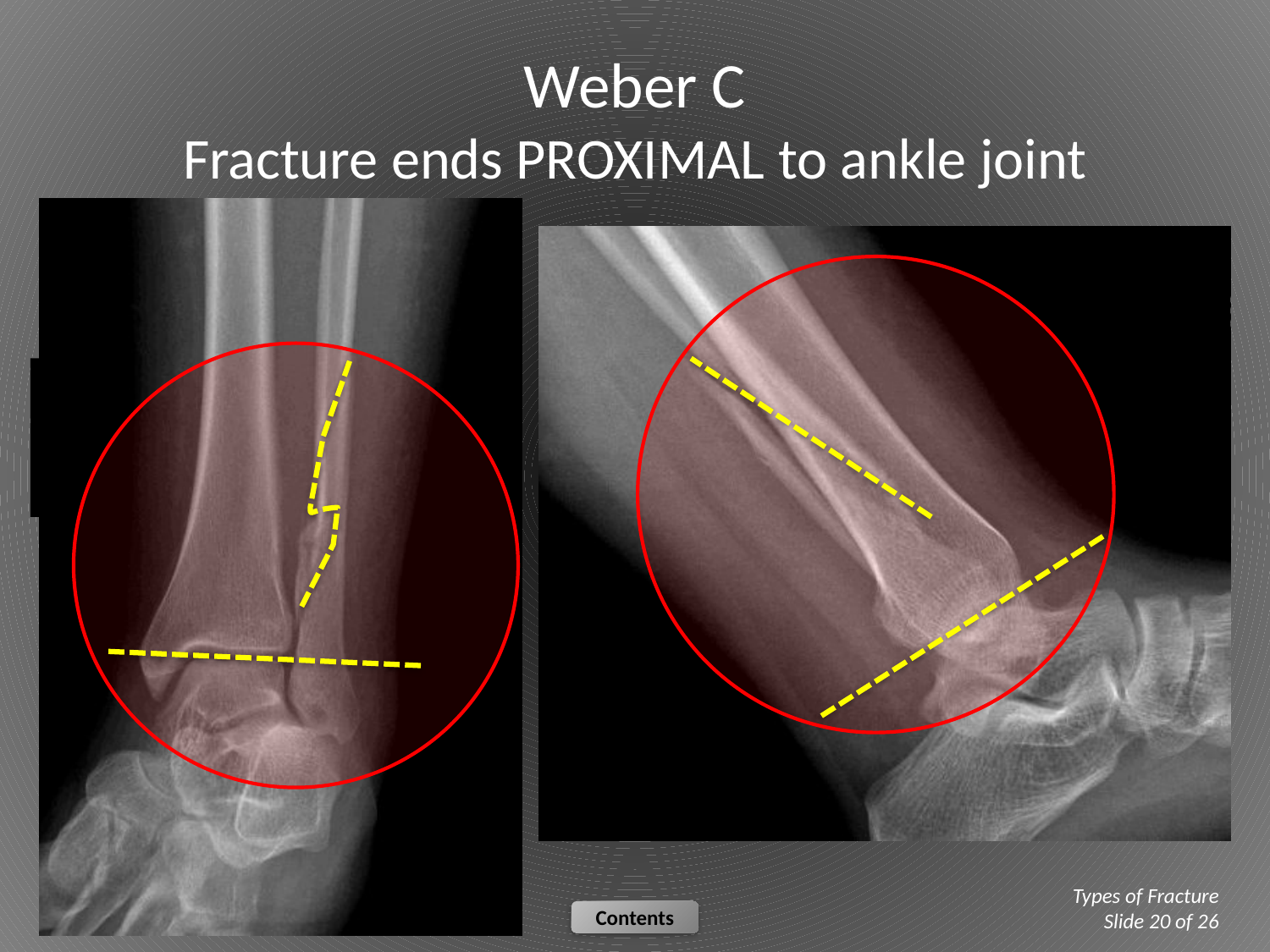

# Weber C Fracture ends PROXIMAL to ankle joint
Types of Fracture
Slide 20 of 26
Contents

## Slide 41
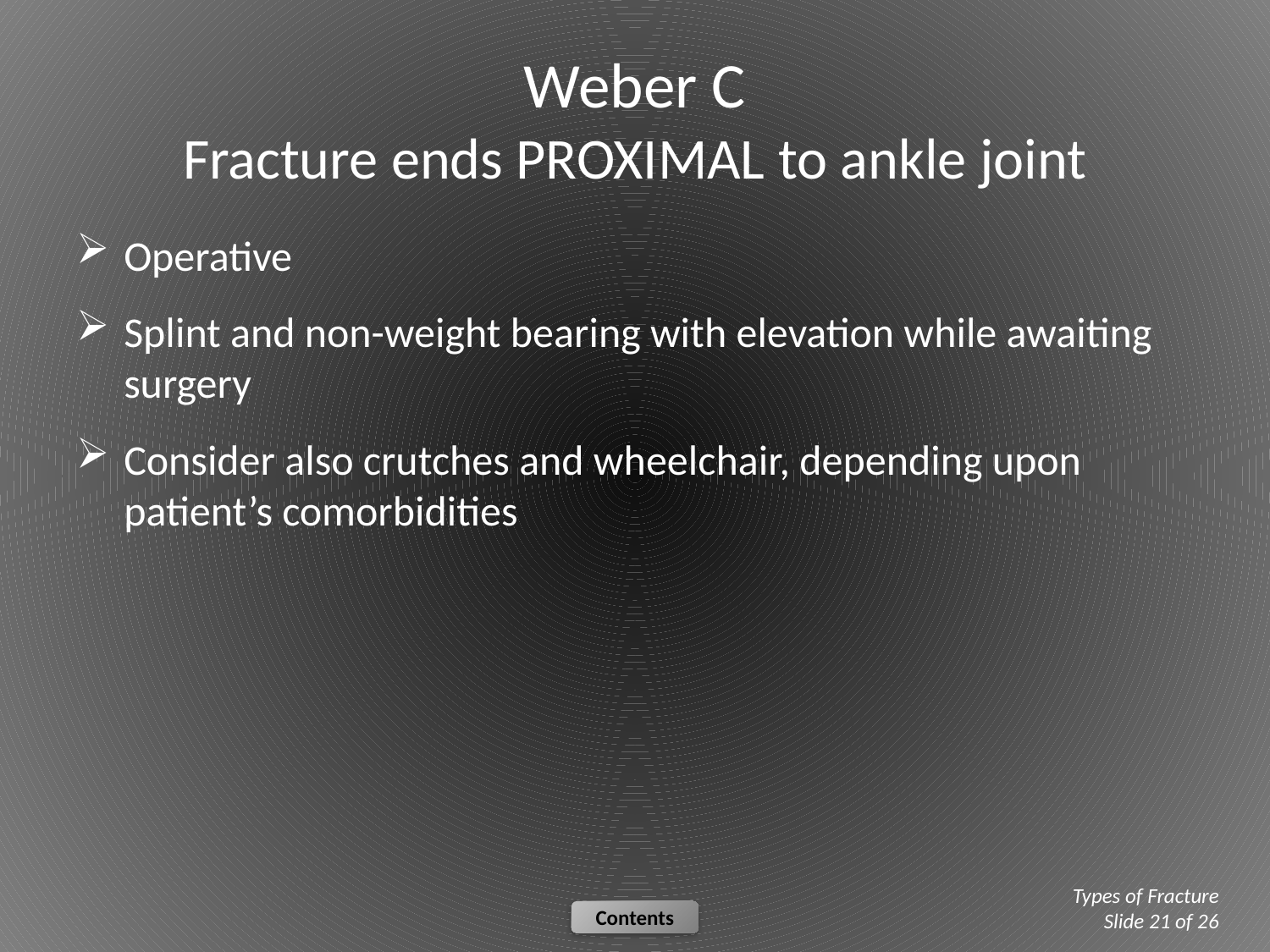

# Weber C Fracture ends PROXIMAL to ankle joint
Operative
Splint and non-weight bearing with elevation while awaiting surgery
Consider also crutches and wheelchair, depending upon patient’s comorbidities
Types of Fracture
Slide 21 of 26
Contents

## Slide 42
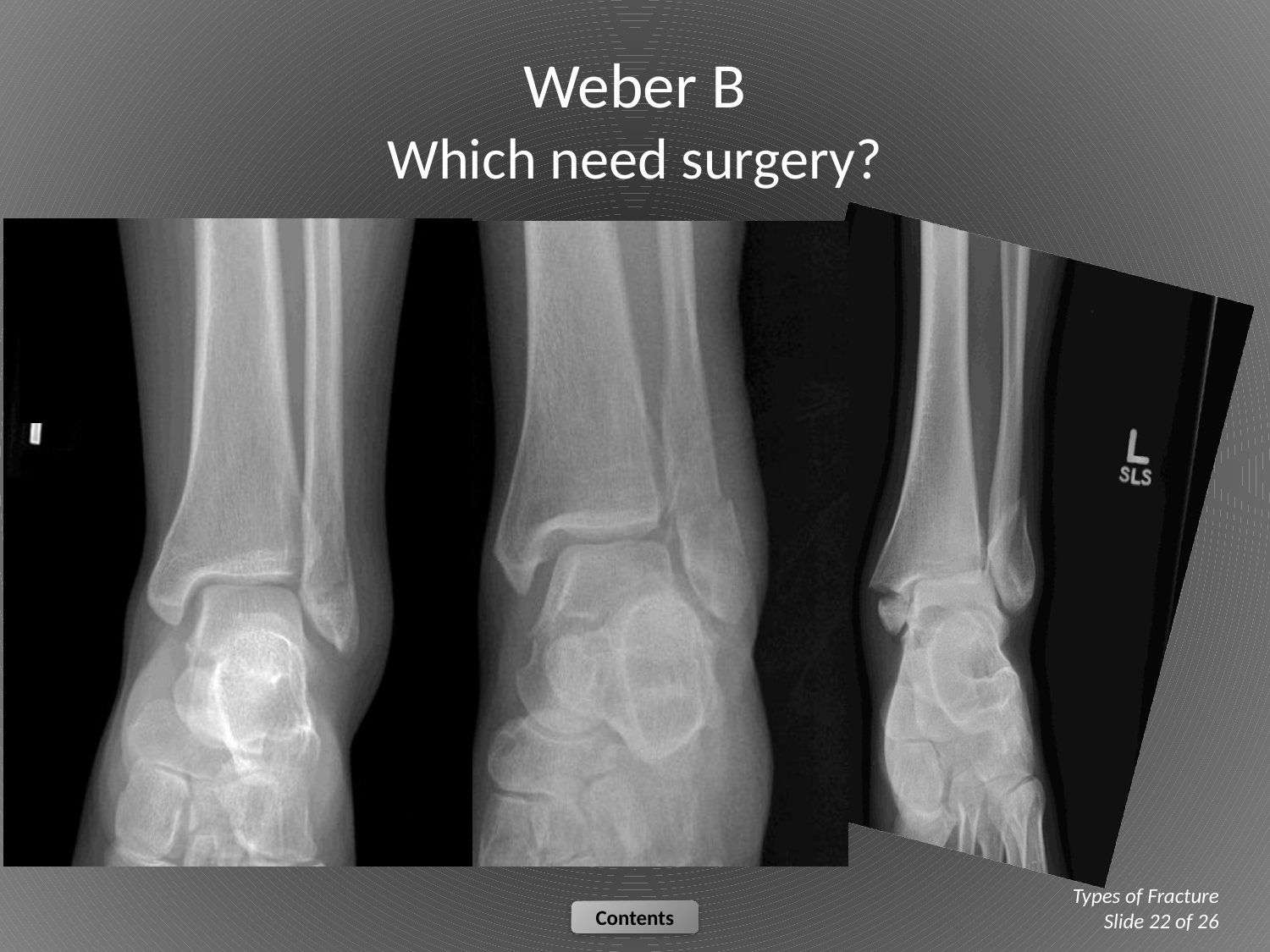

# Weber B Which need surgery?
Types of Fracture
Slide 22 of 26
Contents

## Slide 43
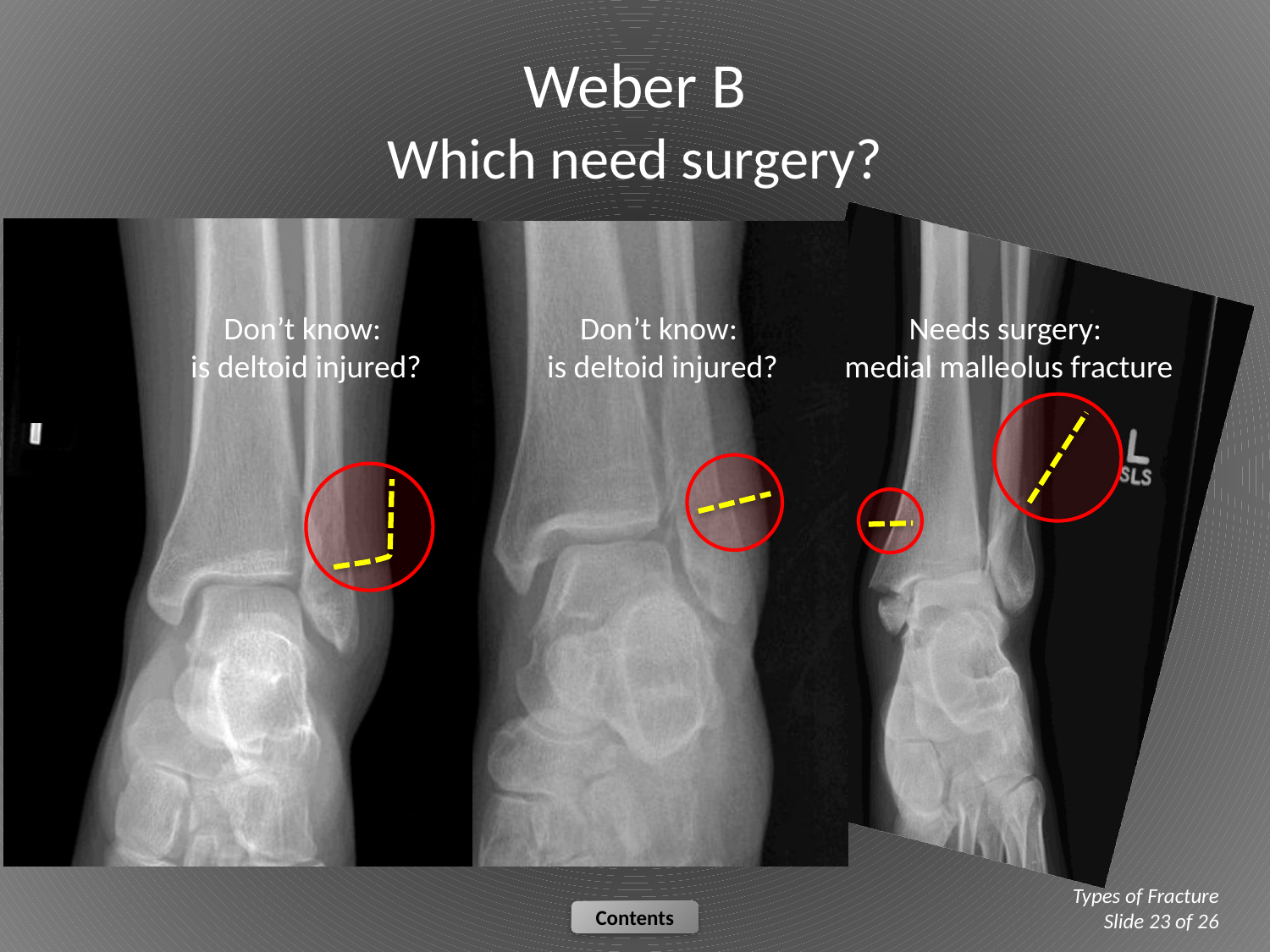

# Weber B Which need surgery?
Don’t know:
is deltoid injured?
Don’t know:
is deltoid injured?
Needs surgery:
medial malleolus fracture
Types of Fracture
Slide 23 of 26
Contents

## Slide 44
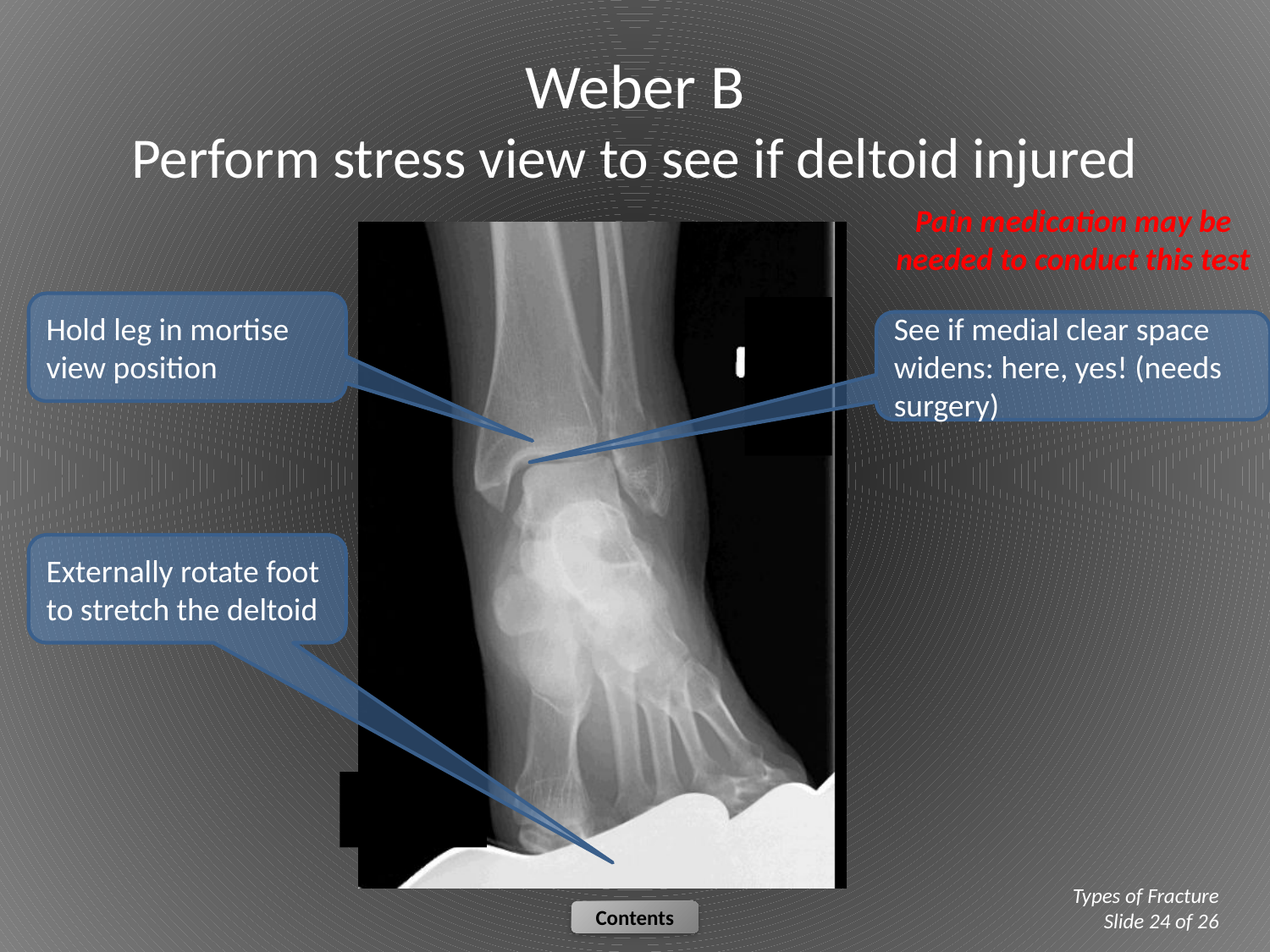

# Weber B Perform stress view to see if deltoid injured
Pain medication may be needed to conduct this test
Hold leg in mortise view position
See if medial clear space widens: here, yes! (needs surgery)
Externally rotate foot to stretch the deltoid
Types of Fracture
Slide 24 of 26
Contents

## Slide 45
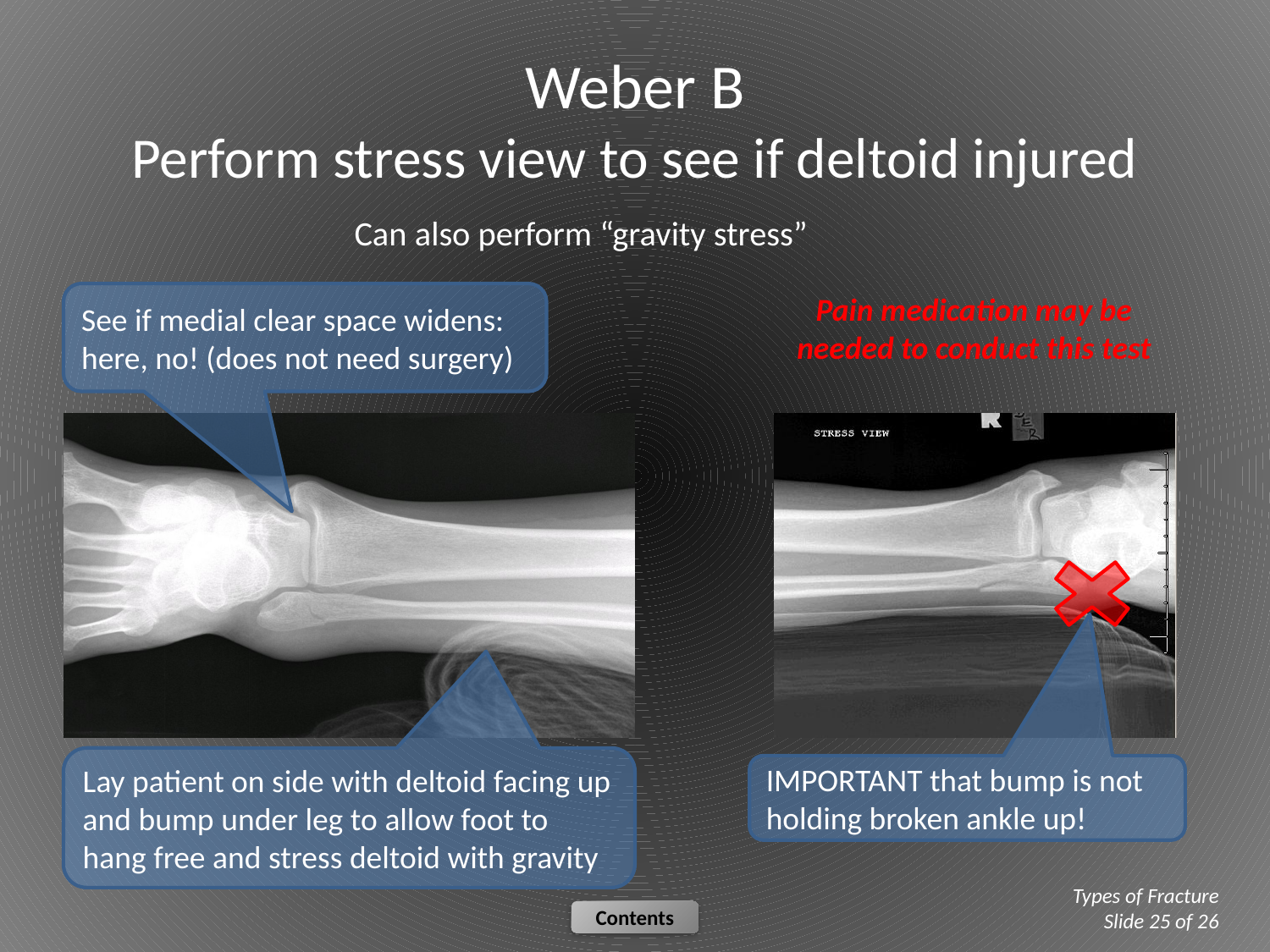

# Weber B Perform stress view to see if deltoid injured
Can also perform “gravity stress”
See if medial clear space widens: here, no! (does not need surgery)
Pain medication may be needed to conduct this test
Lay patient on side with deltoid facing up and bump under leg to allow foot to hang free and stress deltoid with gravity
IMPORTANT that bump is not holding broken ankle up!
Types of Fracture
Slide 25 of 26
Contents

## Slide 46
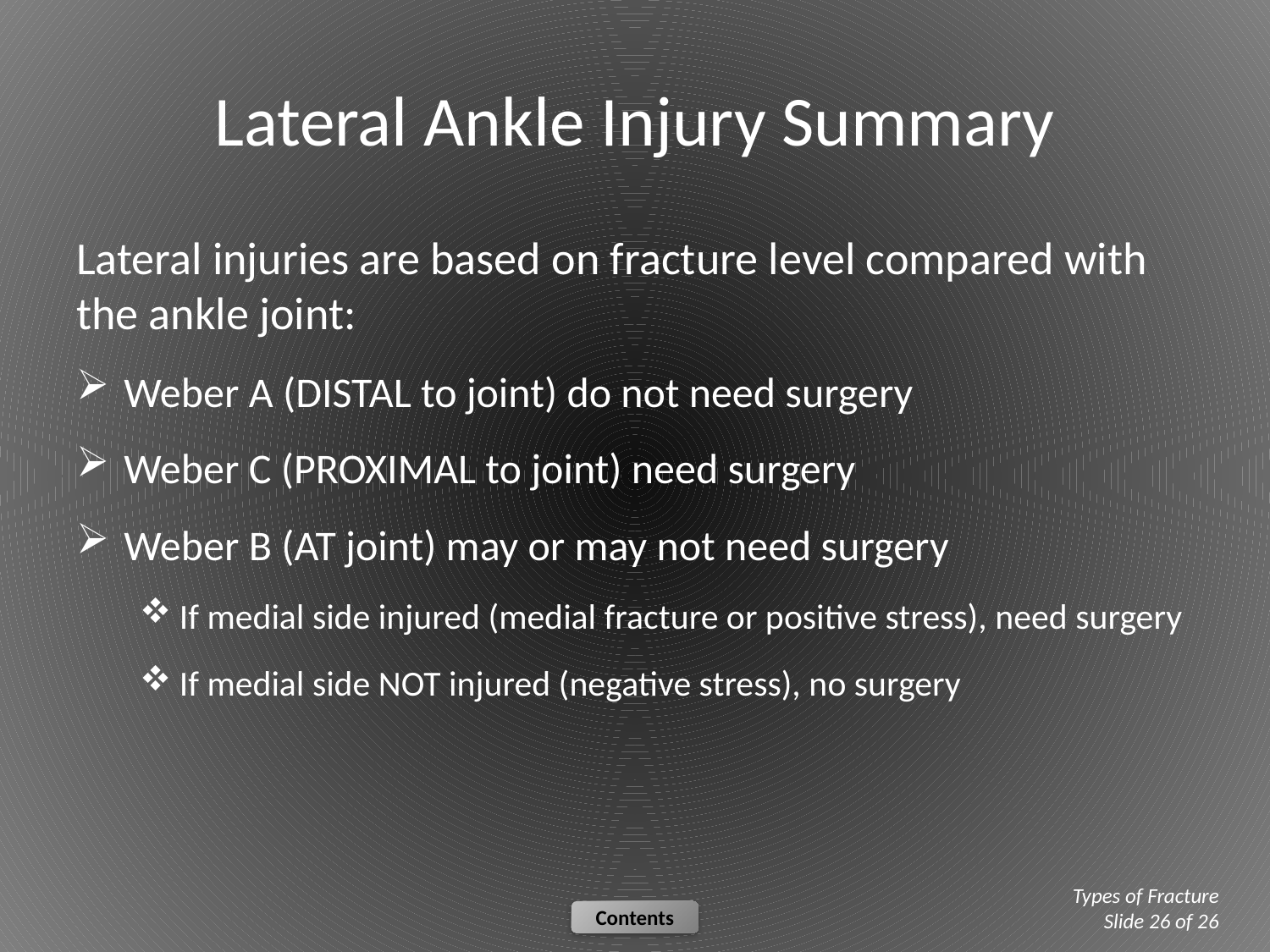

# Lateral Ankle Injury Summary
Lateral injuries are based on fracture level compared with the ankle joint:
Weber A (DISTAL to joint) do not need surgery
Weber C (PROXIMAL to joint) need surgery
Weber B (AT joint) may or may not need surgery
If medial side injured (medial fracture or positive stress), need surgery
If medial side NOT injured (negative stress), no surgery
Types of Fracture
Slide 26 of 26
Contents

## Slide 47
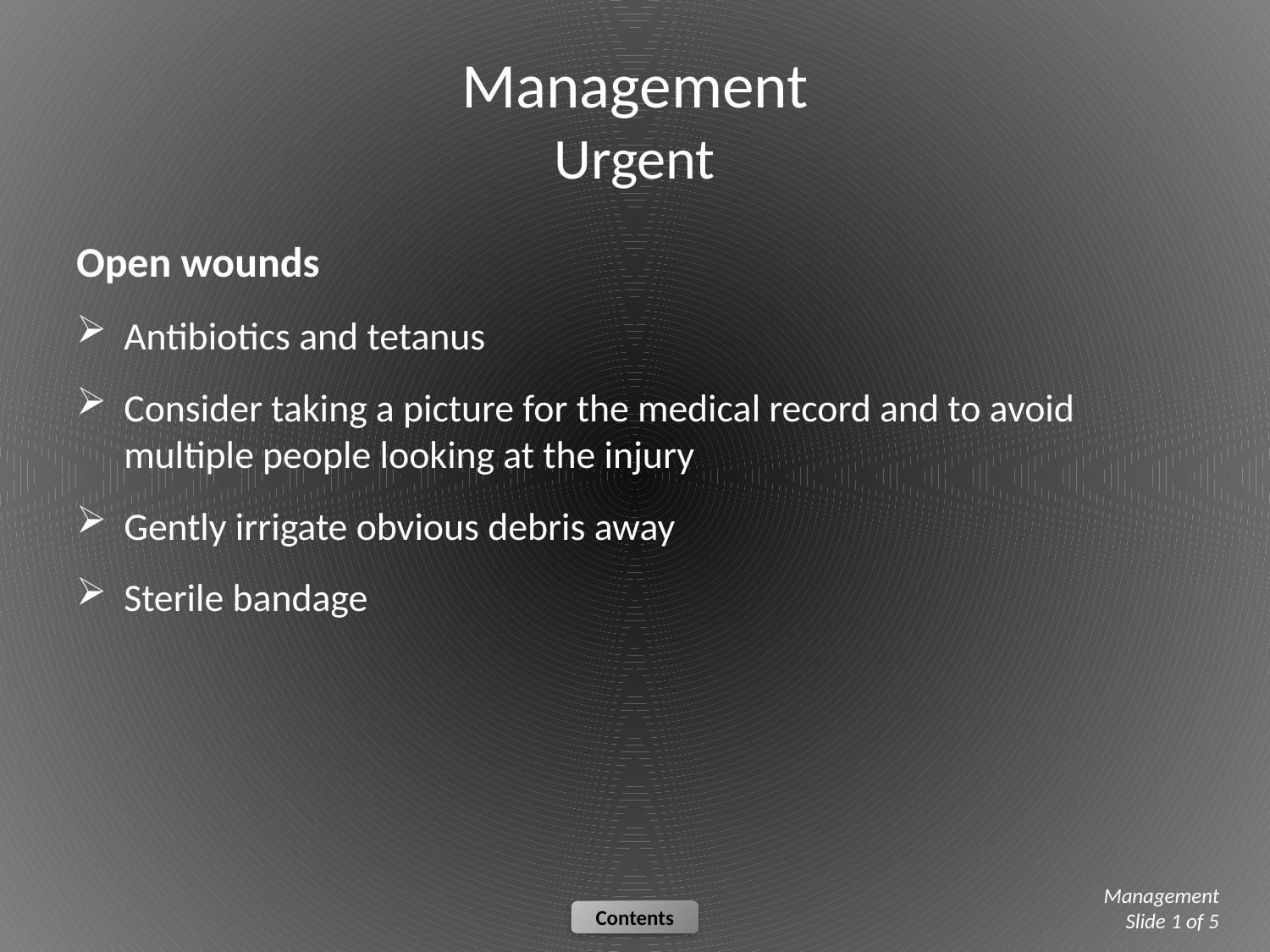

# Management Urgent
Open wounds
Antibiotics and tetanus
Consider taking a picture for the medical record and to avoid multiple people looking at the injury
Gently irrigate obvious debris away
Sterile bandage
Management
Slide 1 of 5
Contents

## Slide 48
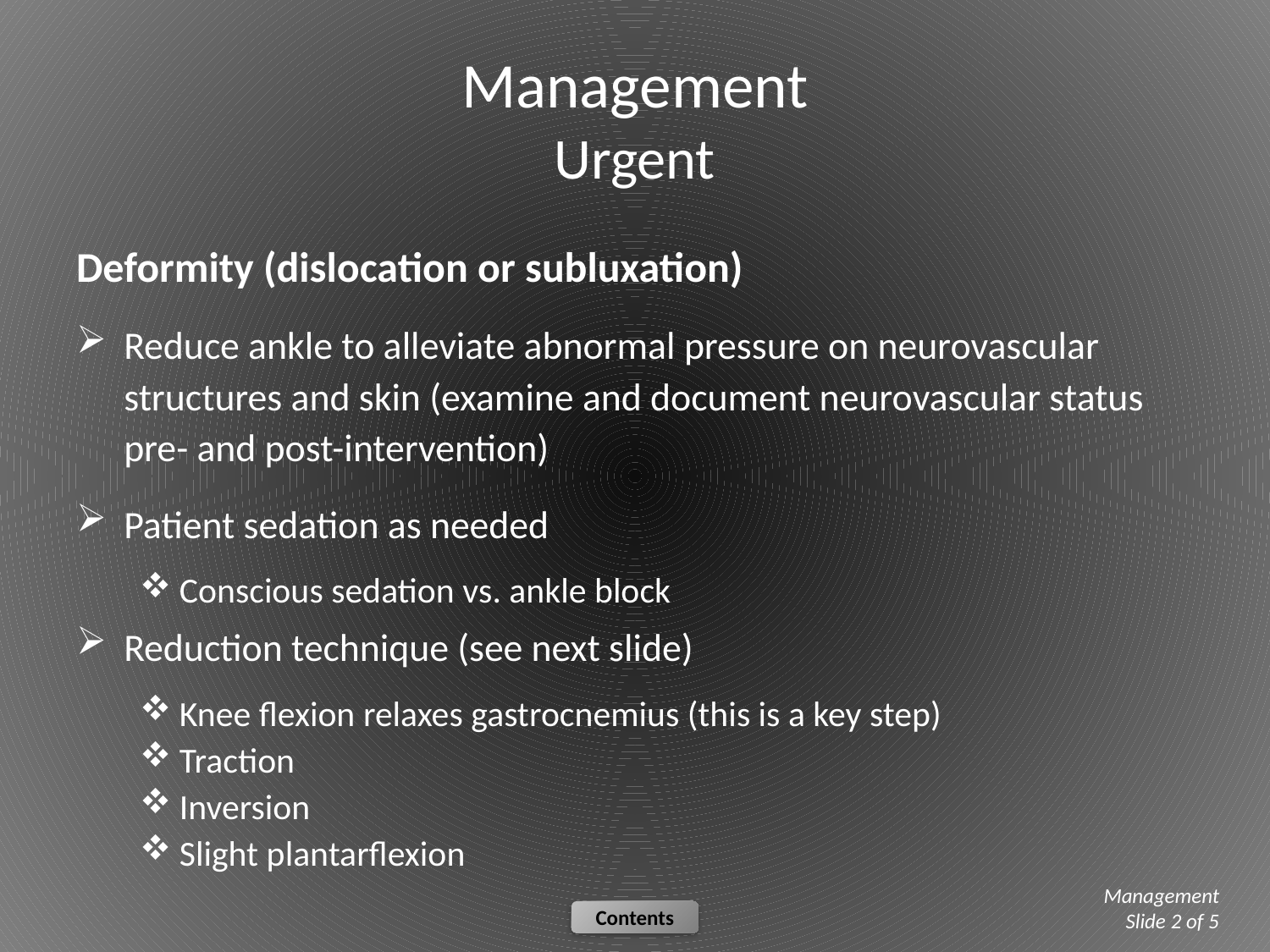

# Management Urgent
Deformity (dislocation or subluxation)
Reduce ankle to alleviate abnormal pressure on neurovascular structures and skin (examine and document neurovascular status pre- and post-intervention)
Patient sedation as needed
Conscious sedation vs. ankle block
Reduction technique (see next slide)
Knee flexion relaxes gastrocnemius (this is a key step)
Traction
Inversion
Slight plantarflexion
Management
Slide 2 of 5
Contents

## Slide 49
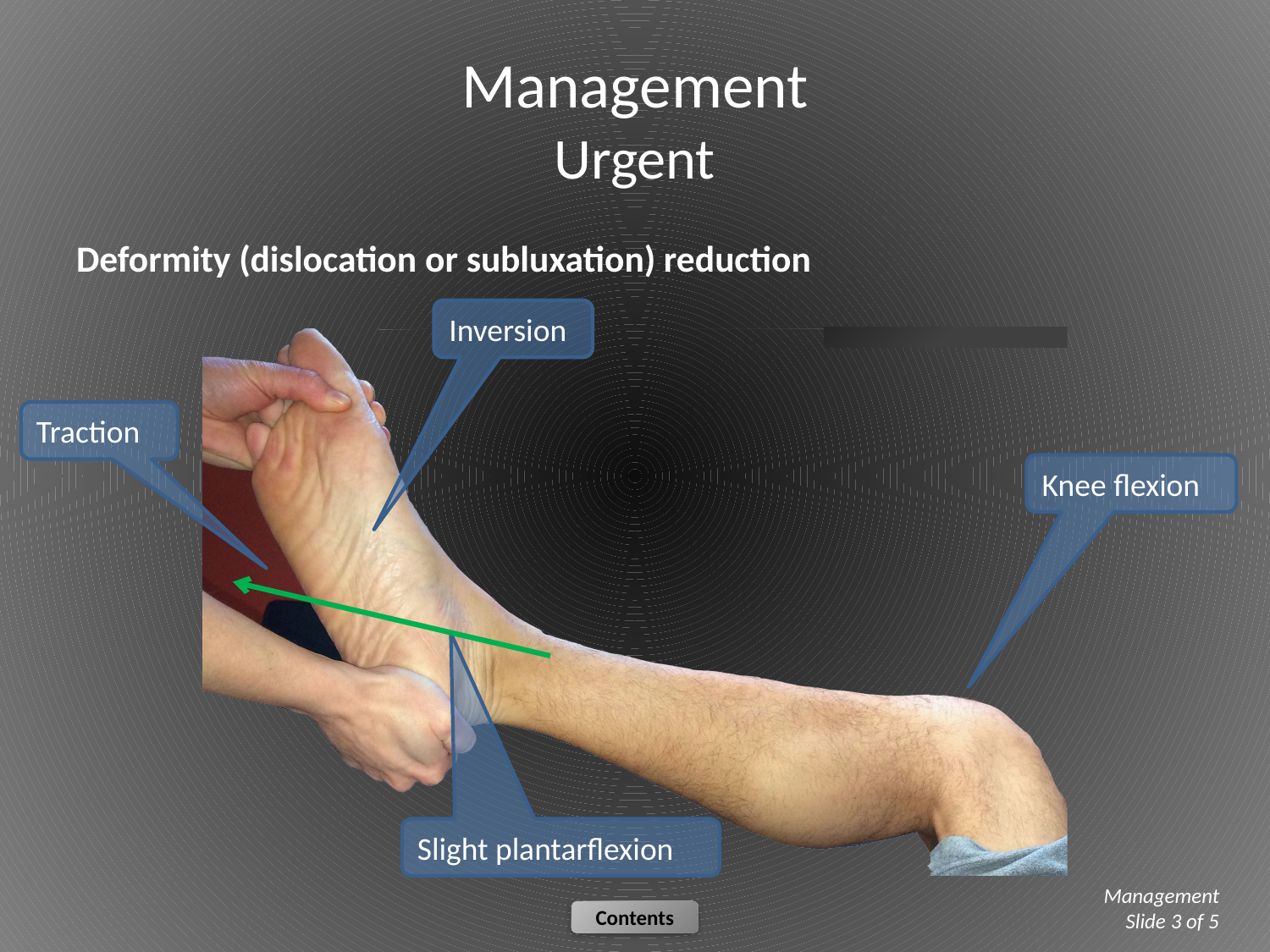

# Management Urgent
Deformity (dislocation or subluxation) reduction
Inversion
Traction
Knee flexion
Slight plantarflexion
Management
Slide 3 of 5
Contents

## Slide 50
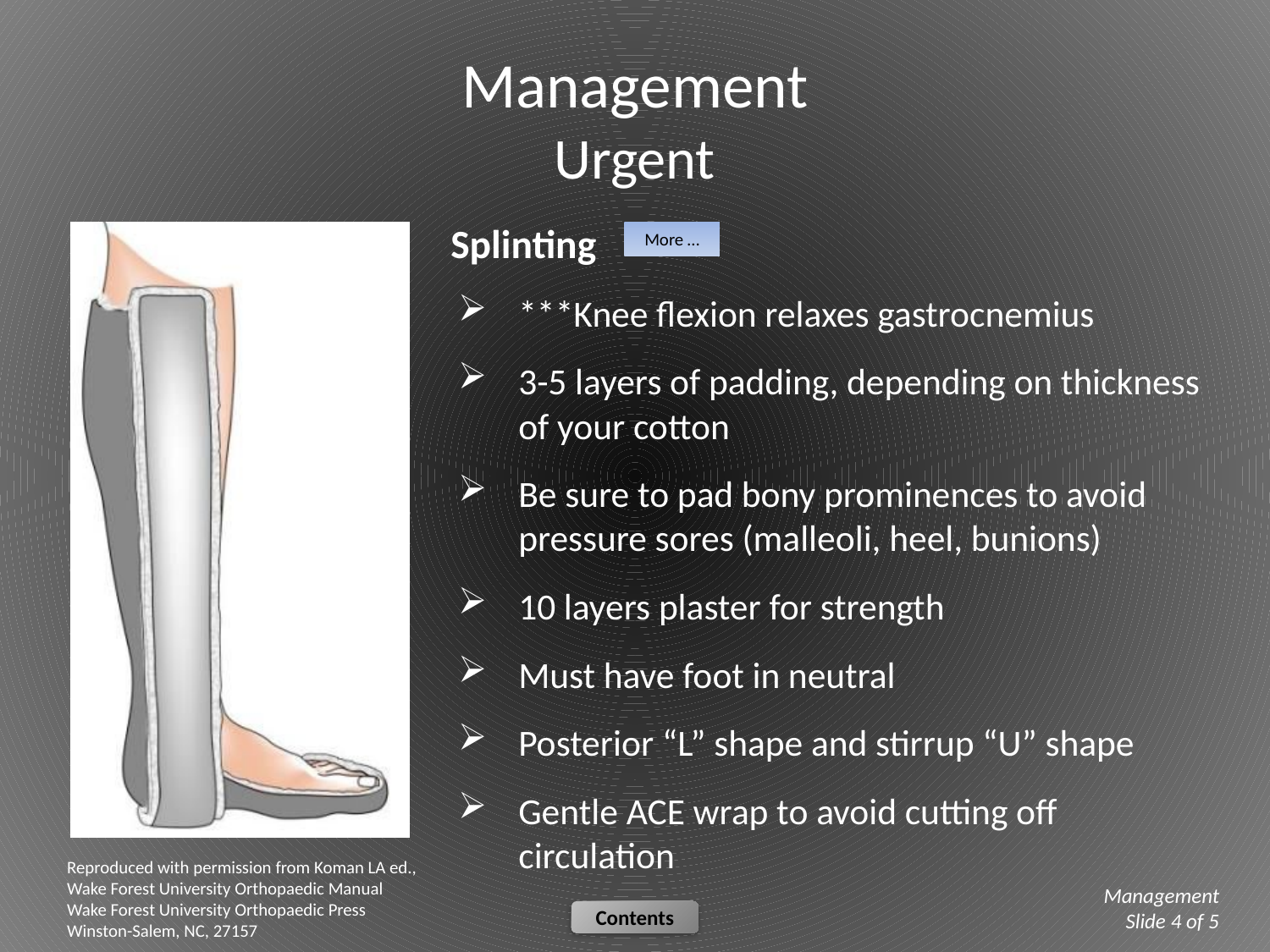

# Management Urgent
Splinting
***Knee flexion relaxes gastrocnemius
3-5 layers of padding, depending on thickness of your cotton
Be sure to pad bony prominences to avoid pressure sores (malleoli, heel, bunions)
10 layers plaster for strength
Must have foot in neutral
Posterior “L” shape and stirrup “U” shape
Gentle ACE wrap to avoid cutting off circulation
More …
Reproduced with permission from Koman LA ed.,
Wake Forest University Orthopaedic Manual
Wake Forest University Orthopaedic Press
Winston-Salem, NC, 27157
Management
Slide 4 of 5
Contents

## Slide 51
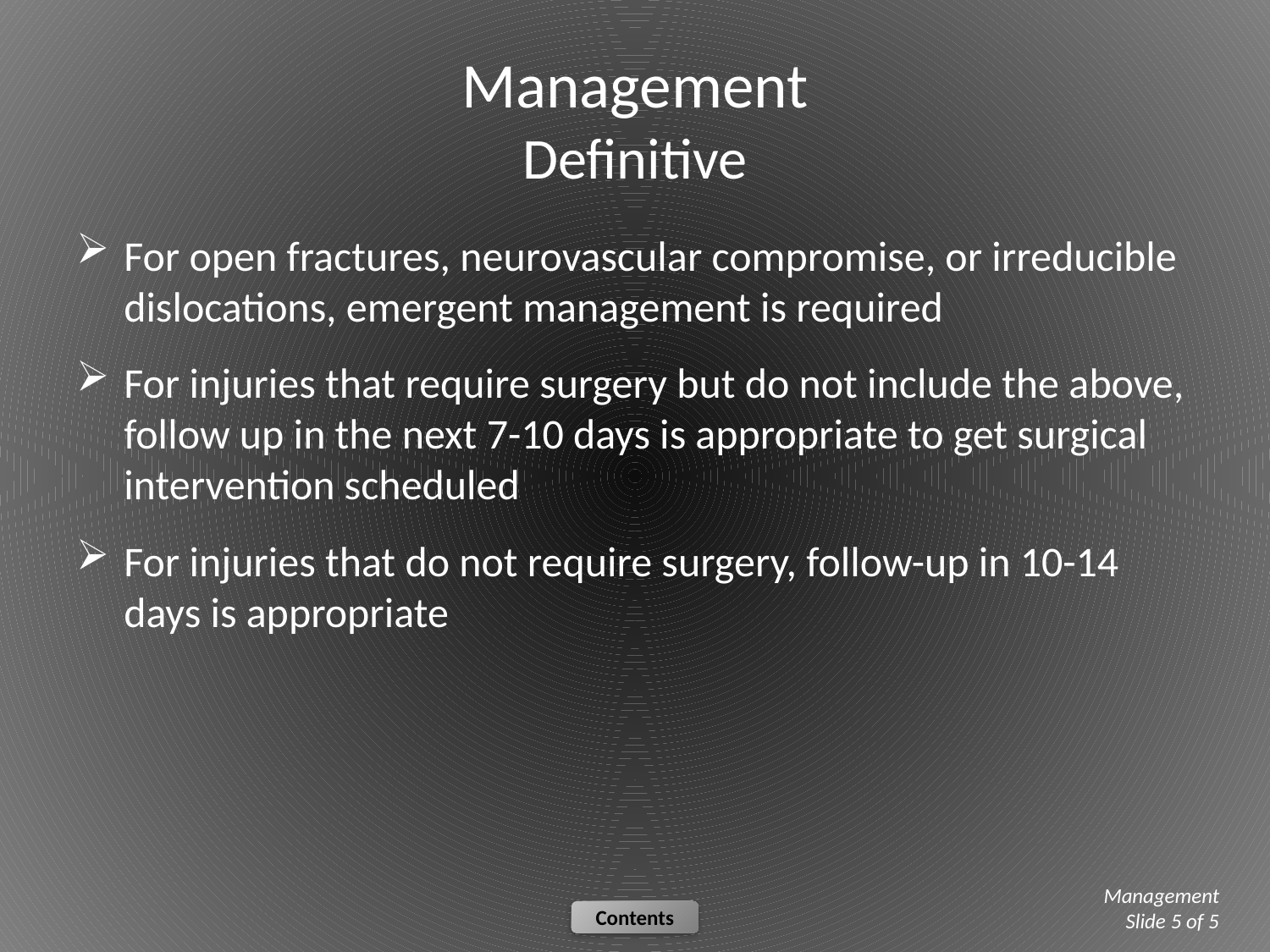

# Management Definitive
For open fractures, neurovascular compromise, or irreducible dislocations, emergent management is required
For injuries that require surgery but do not include the above, follow up in the next 7-10 days is appropriate to get surgical intervention scheduled
For injuries that do not require surgery, follow-up in 10-14 days is appropriate
Management
Slide 5 of 5
Contents

## Slide 52
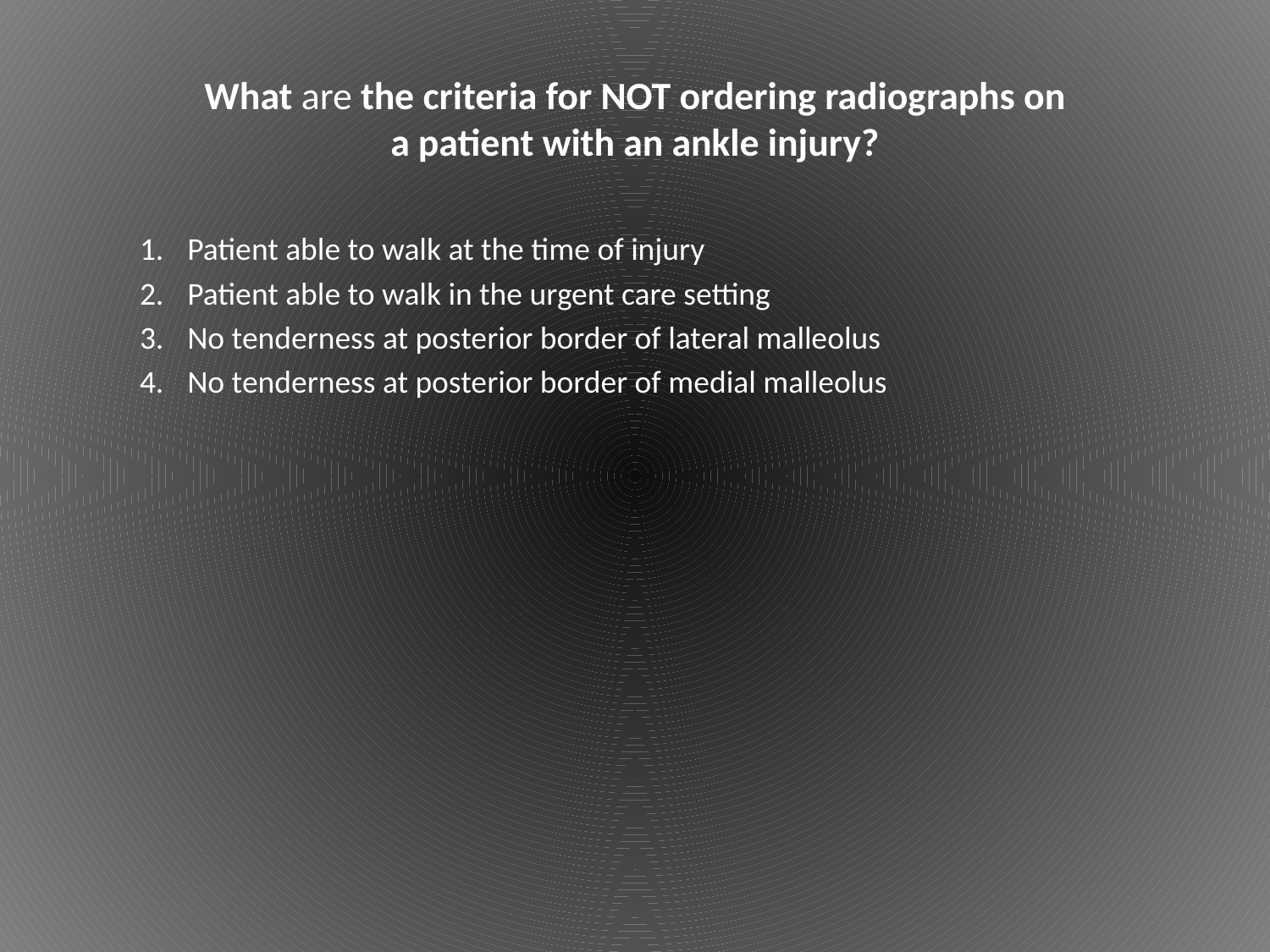

# What are the criteria for NOT ordering radiographs on a patient with an ankle injury?
Patient able to walk at the time of injury
Patient able to walk in the urgent care setting
No tenderness at posterior border of lateral malleolus
No tenderness at posterior border of medial malleolus

## Slide 53
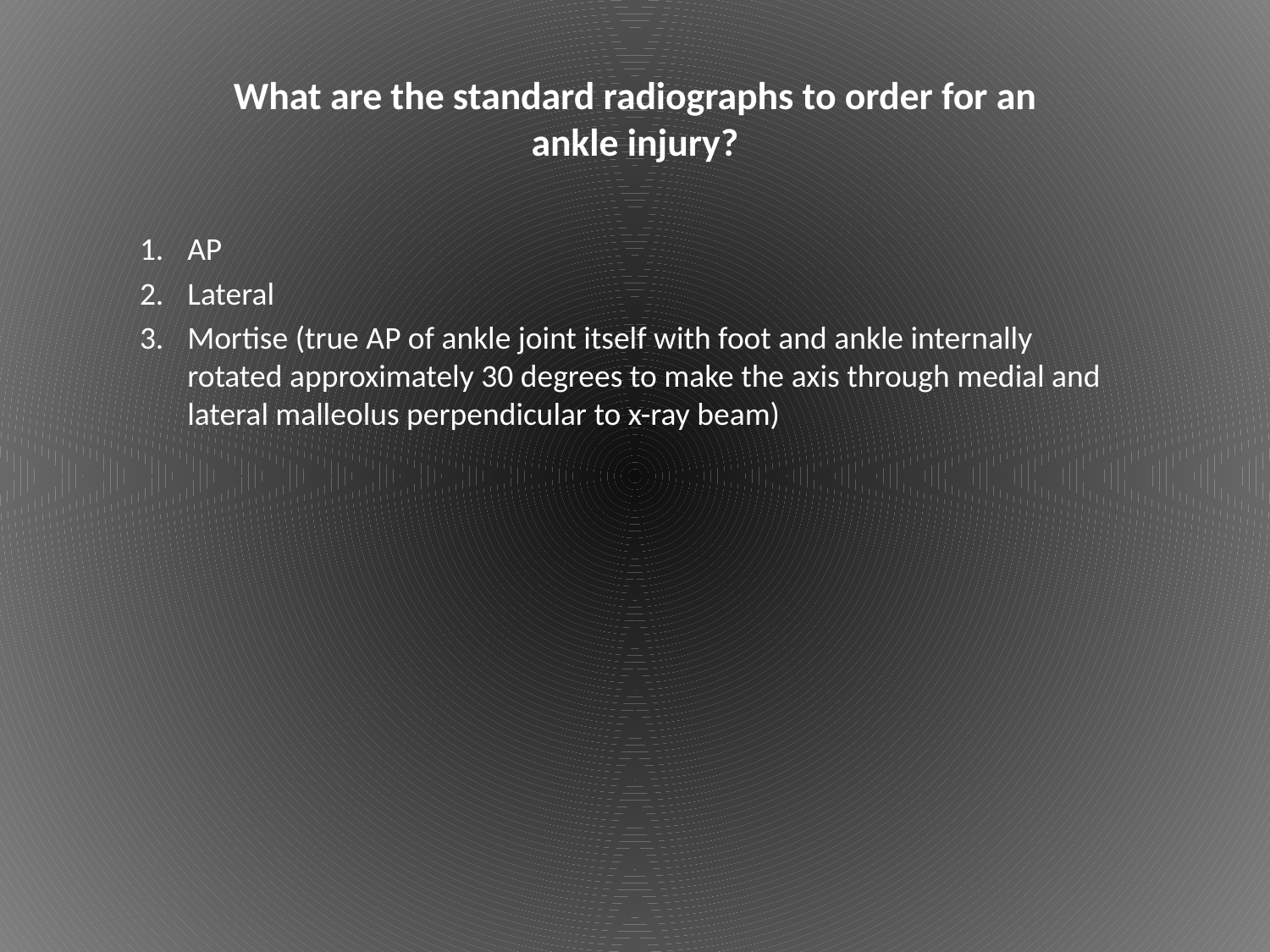

# What are the standard radiographs to order for an ankle injury?
AP
Lateral
Mortise (true AP of ankle joint itself with foot and ankle internally rotated approximately 30 degrees to make the axis through medial and lateral malleolus perpendicular to x-ray beam)

## Slide 54
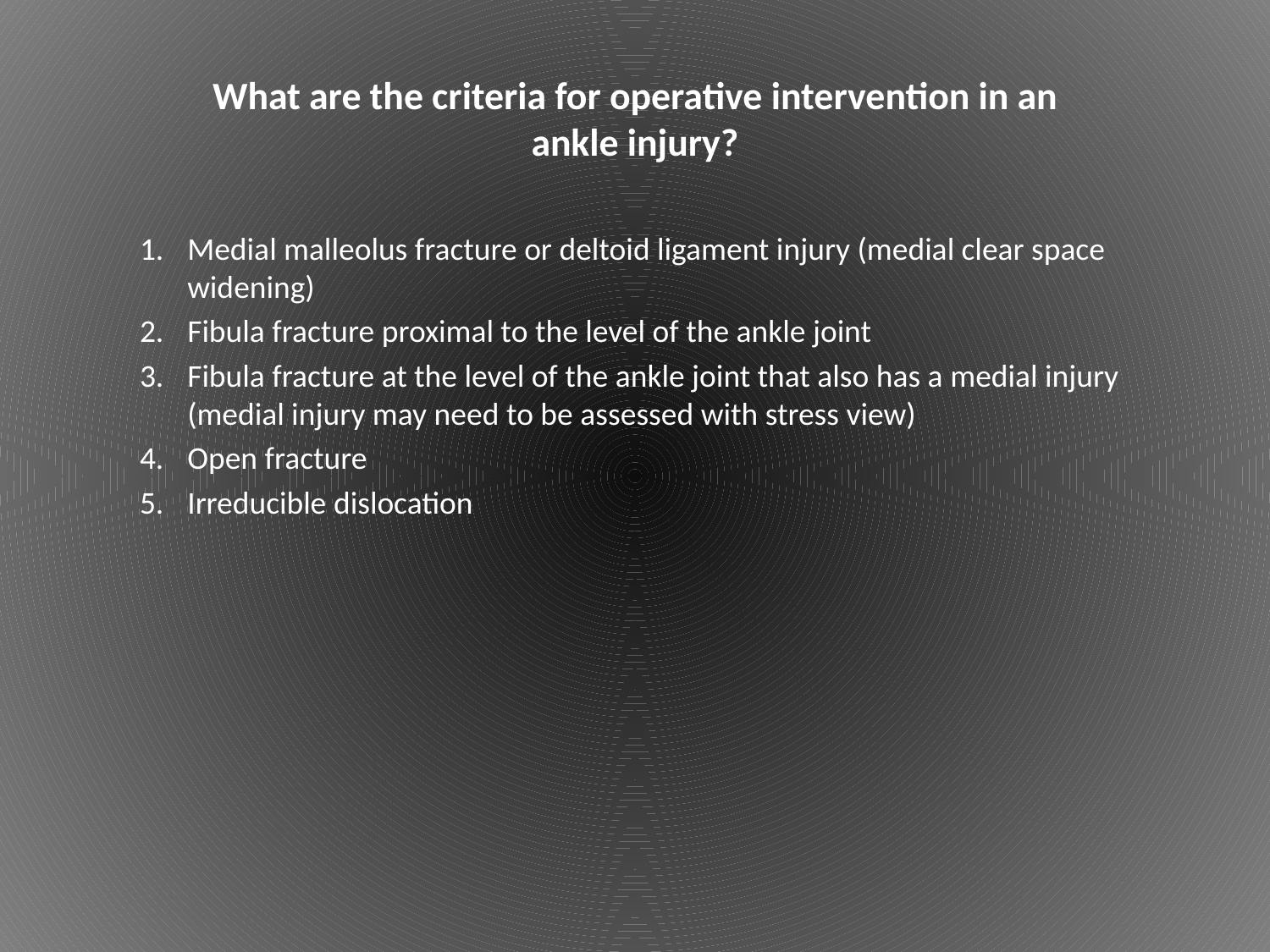

# What are the criteria for operative intervention in an ankle injury?
Medial malleolus fracture or deltoid ligament injury (medial clear space widening)
Fibula fracture proximal to the level of the ankle joint
Fibula fracture at the level of the ankle joint that also has a medial injury (medial injury may need to be assessed with stress view)
Open fracture
Irreducible dislocation

## Slide 55
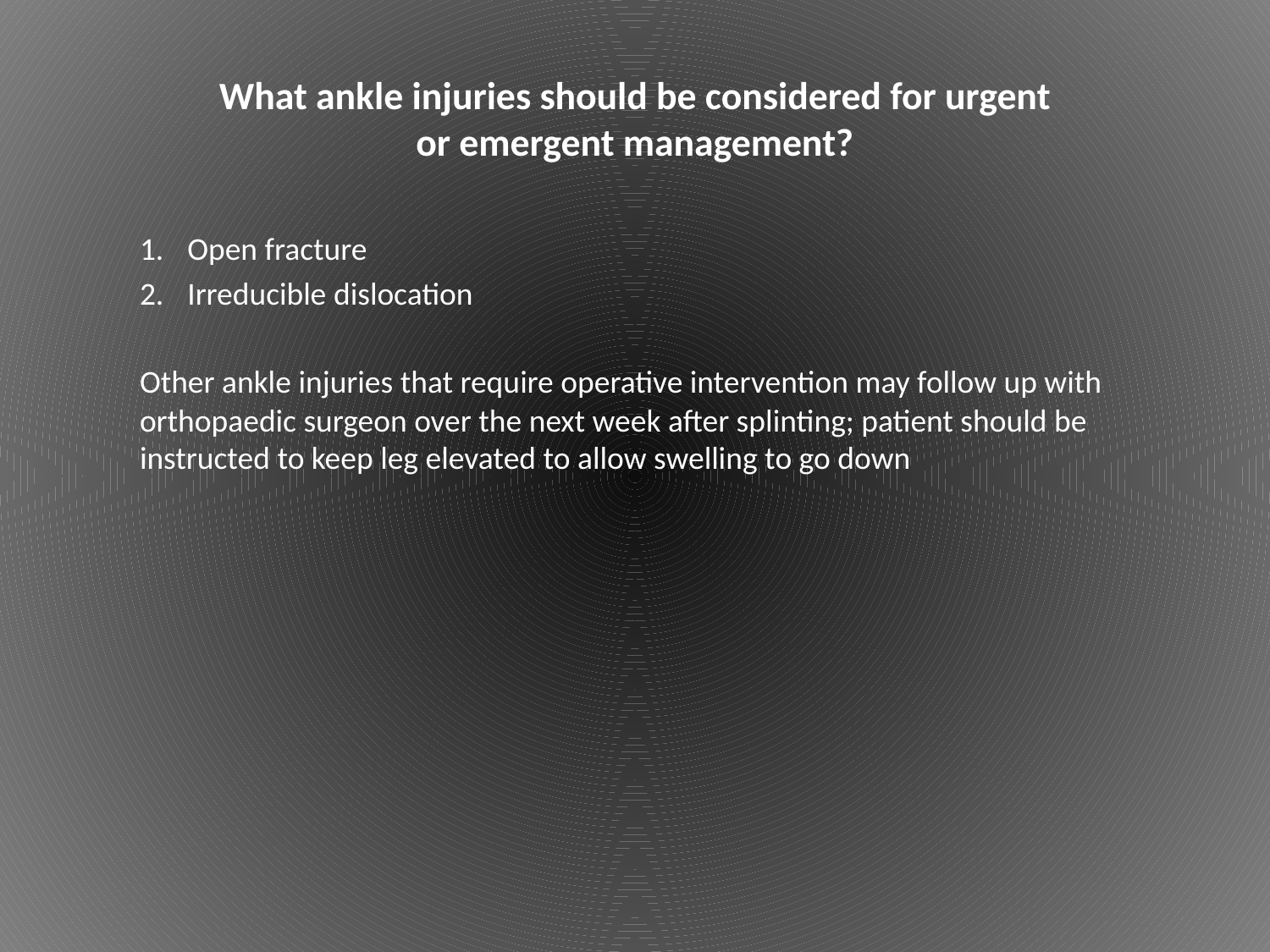

# What ankle injuries should be considered for urgent or emergent management?
Open fracture
Irreducible dislocation
Other ankle injuries that require operative intervention may follow up with orthopaedic surgeon over the next week after splinting; patient should be instructed to keep leg elevated to allow swelling to go down

## Slide 56
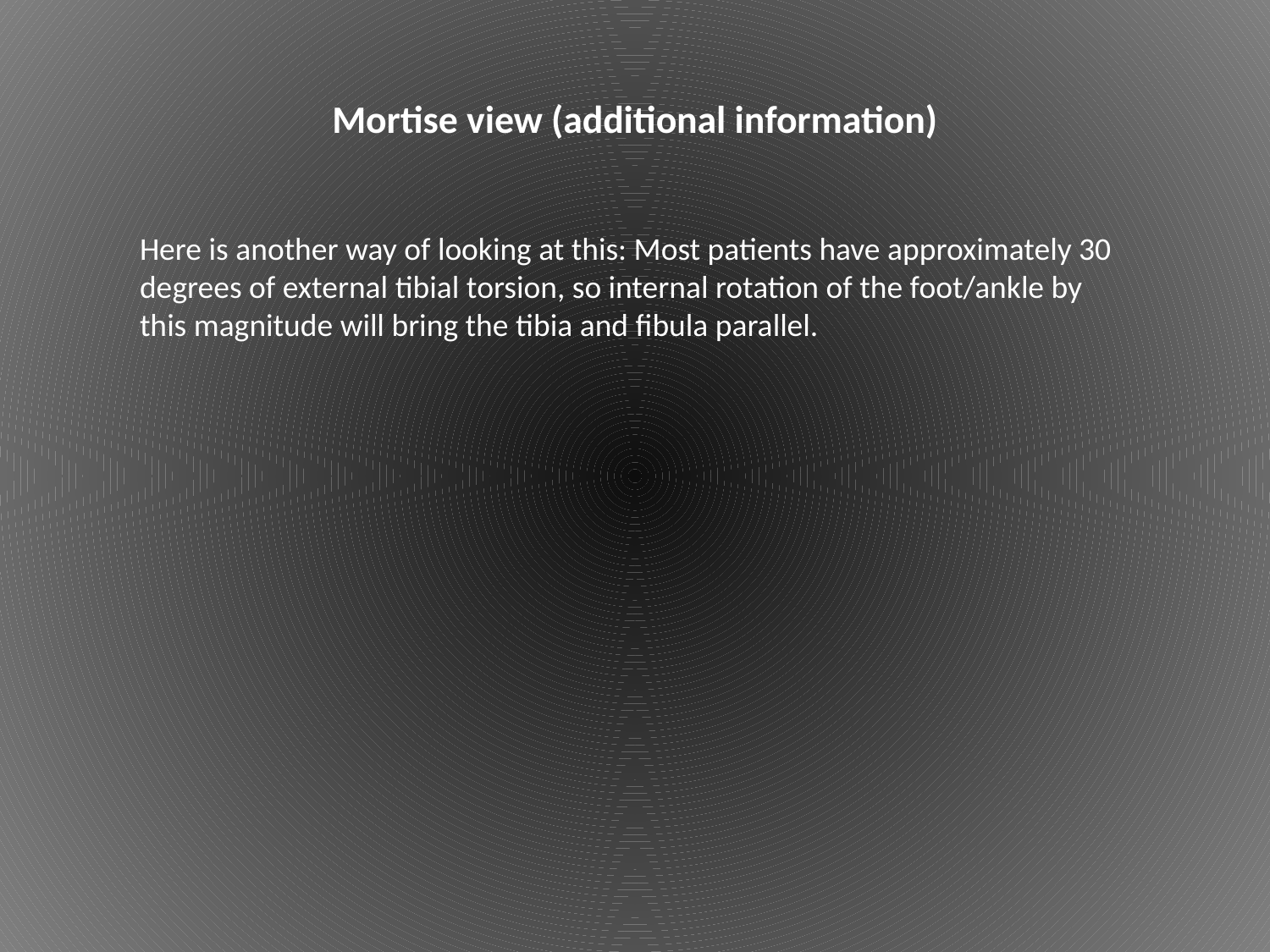

# Mortise view (additional information)
Here is another way of looking at this: Most patients have approximately 30 degrees of external tibial torsion, so internal rotation of the foot/ankle by this magnitude will bring the tibia and fibula parallel.

## Slide 57
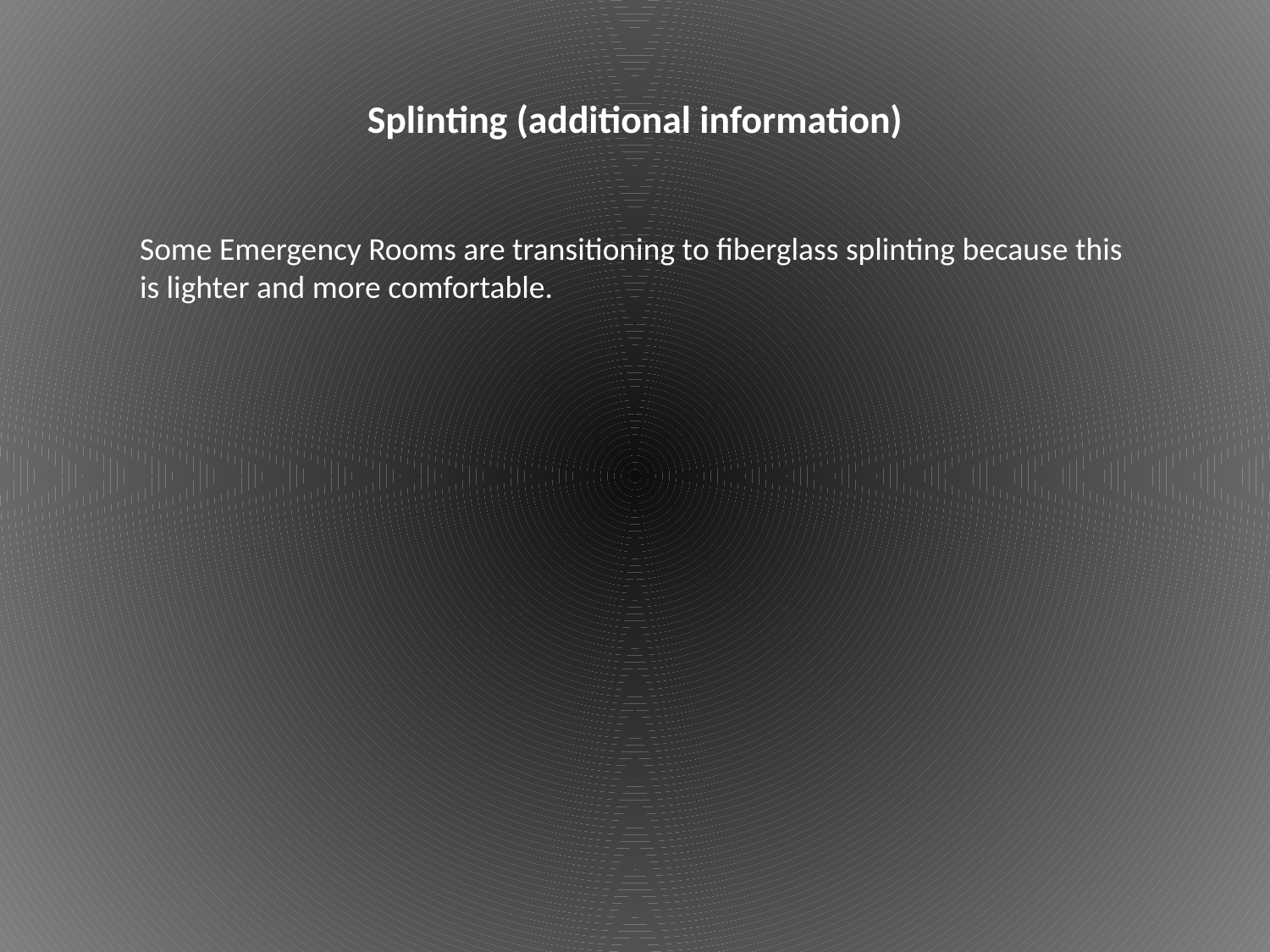

# Splinting (additional information)
Some Emergency Rooms are transitioning to fiberglass splinting because this is lighter and more comfortable.
