# Supplementary material for: Evaluation of Common Musculoskeletal Injuries in the Urgent Setting
Source: MedEdPORTAL. 2016 Dec 7;12:10514. doi: 10.15766/mep_2374-8265.10514 (PMC6440529; doi:10.15766/mep_2374-8265.10514)
Supplement: Supplementary file 1 — A. Evaluation of Common Musculoskeletal Injuries in the Urgent Setting.pptx B. Evaluation of Ankle Injuries in the Urgent Setting.pptx C. Evaluation of Hip Injuries in the Urgent Setting.pptx D. Evaluation of Shoulder Injuries in the Urgent Setting.pptx E. Evaluation of Wrist Injuries in the Urgent Setting.pptx [file mep-12-10514-s001.zip › C. Evaluation of Hip Injuries in the Urgent Setting.pptx]

## Slide 1
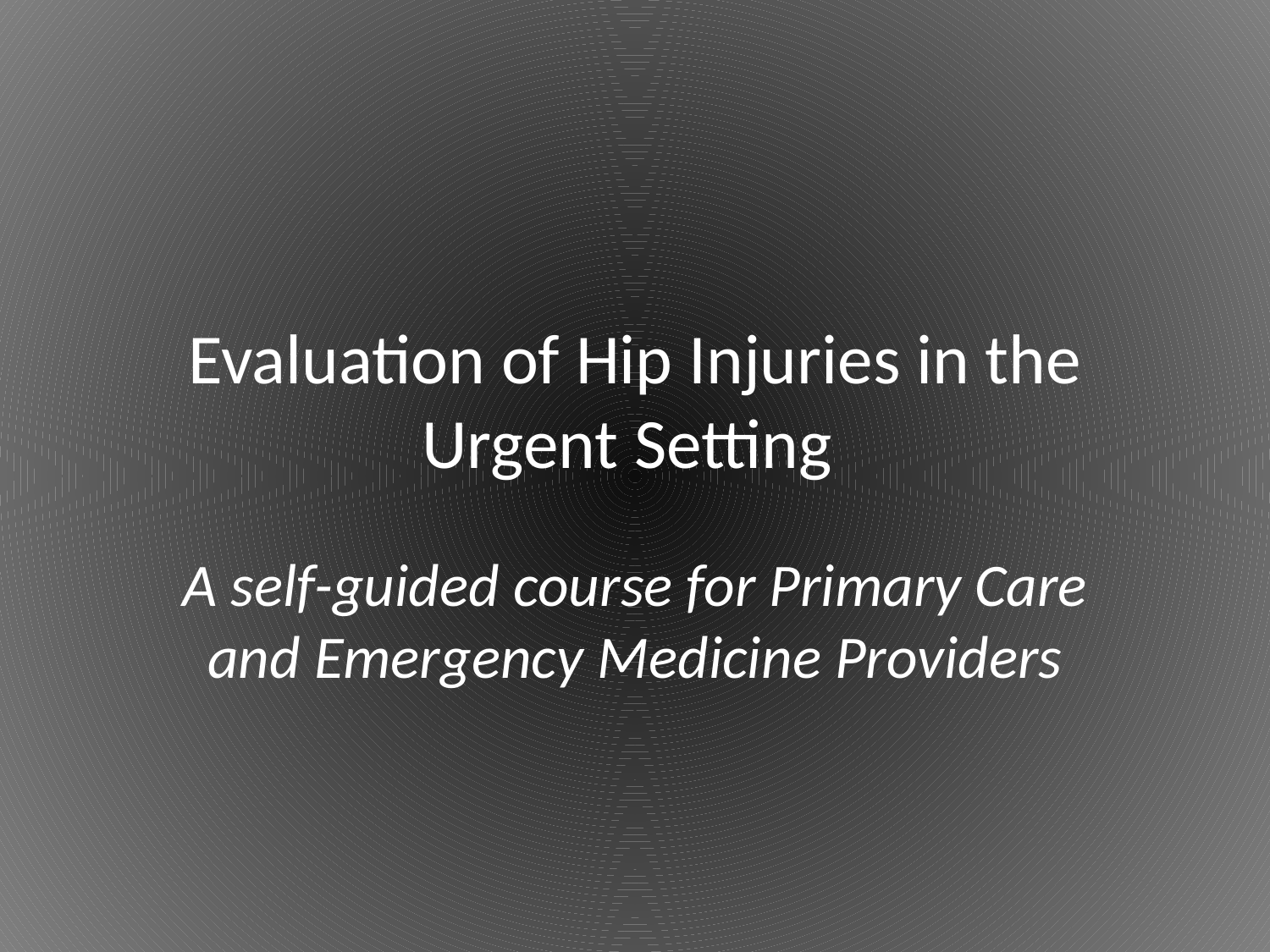

# Evaluation of Hip Injuries in the Urgent Setting
A self-guided course for Primary Care and Emergency Medicine Providers

## Slide 2
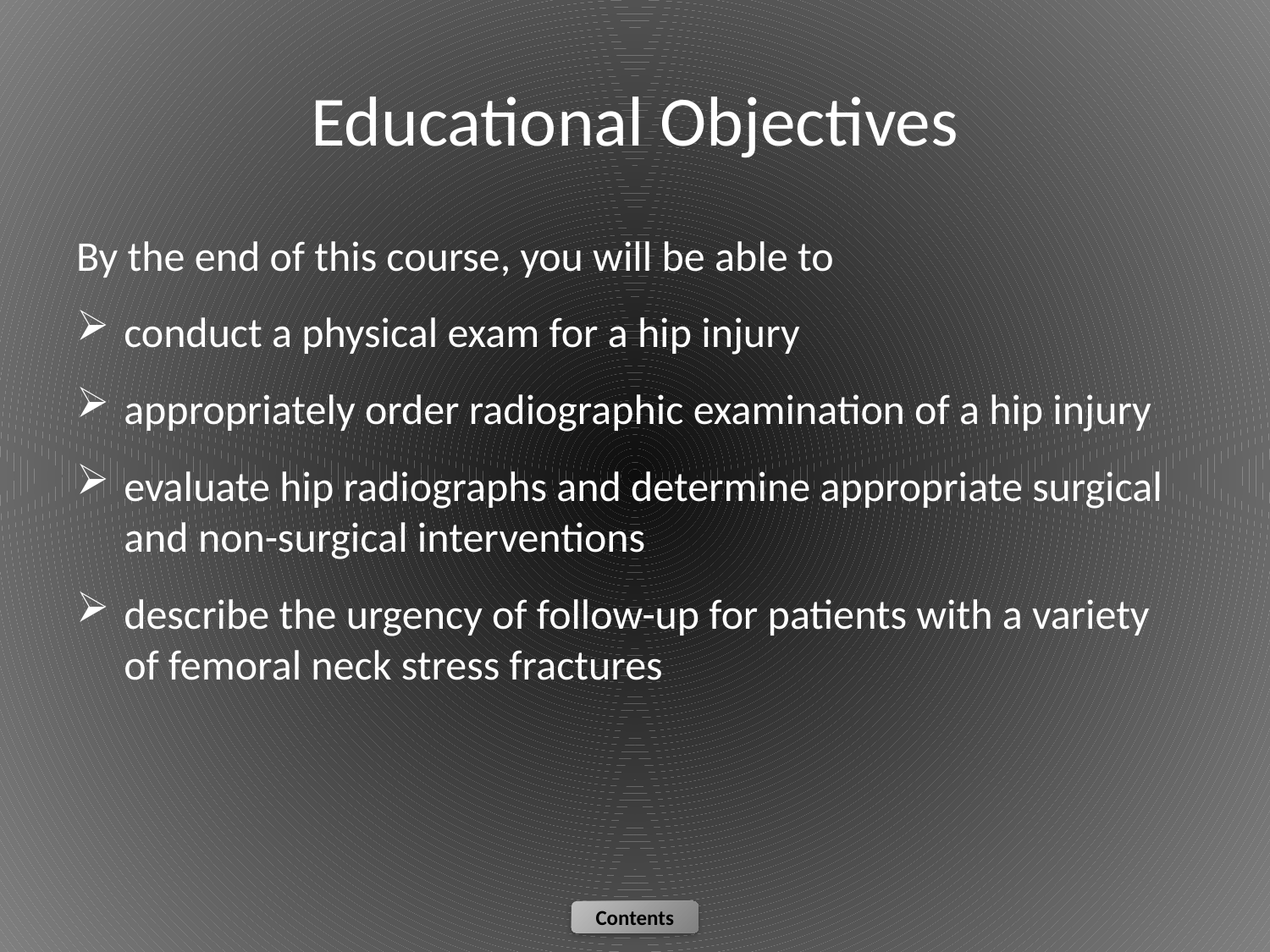

# Educational Objectives
By the end of this course, you will be able to
conduct a physical exam for a hip injury
appropriately order radiographic examination of a hip injury
evaluate hip radiographs and determine appropriate surgical and non-surgical interventions
describe the urgency of follow-up for patients with a variety of femoral neck stress fractures
Contents

## Slide 3
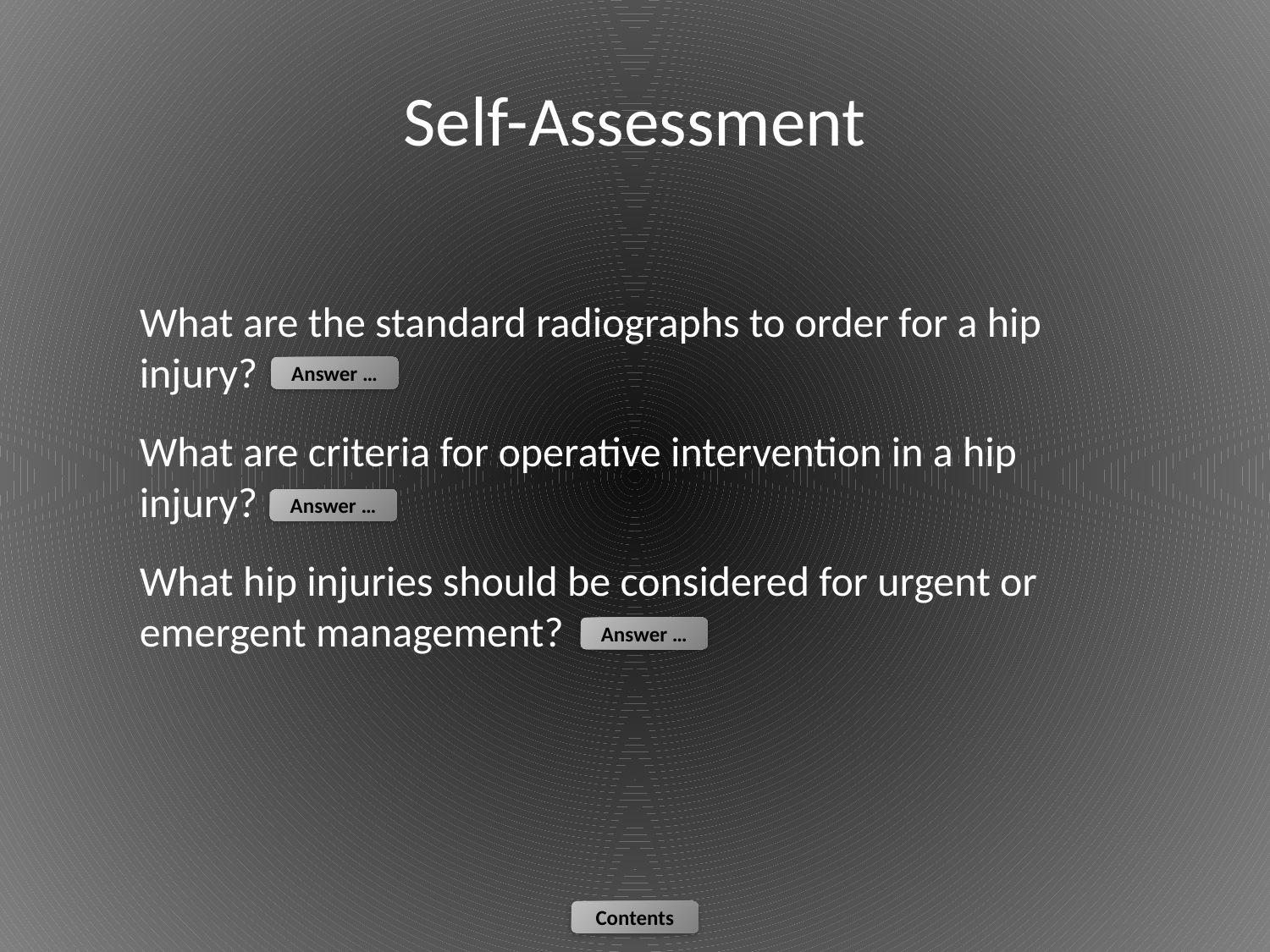

# Self-Assessment
What are the standard radiographs to order for a hip injury?
What are criteria for operative intervention in a hip injury?
What hip injuries should be considered for urgent or emergent management?
Answer …
Answer …
Answer …
Contents

## Slide 4
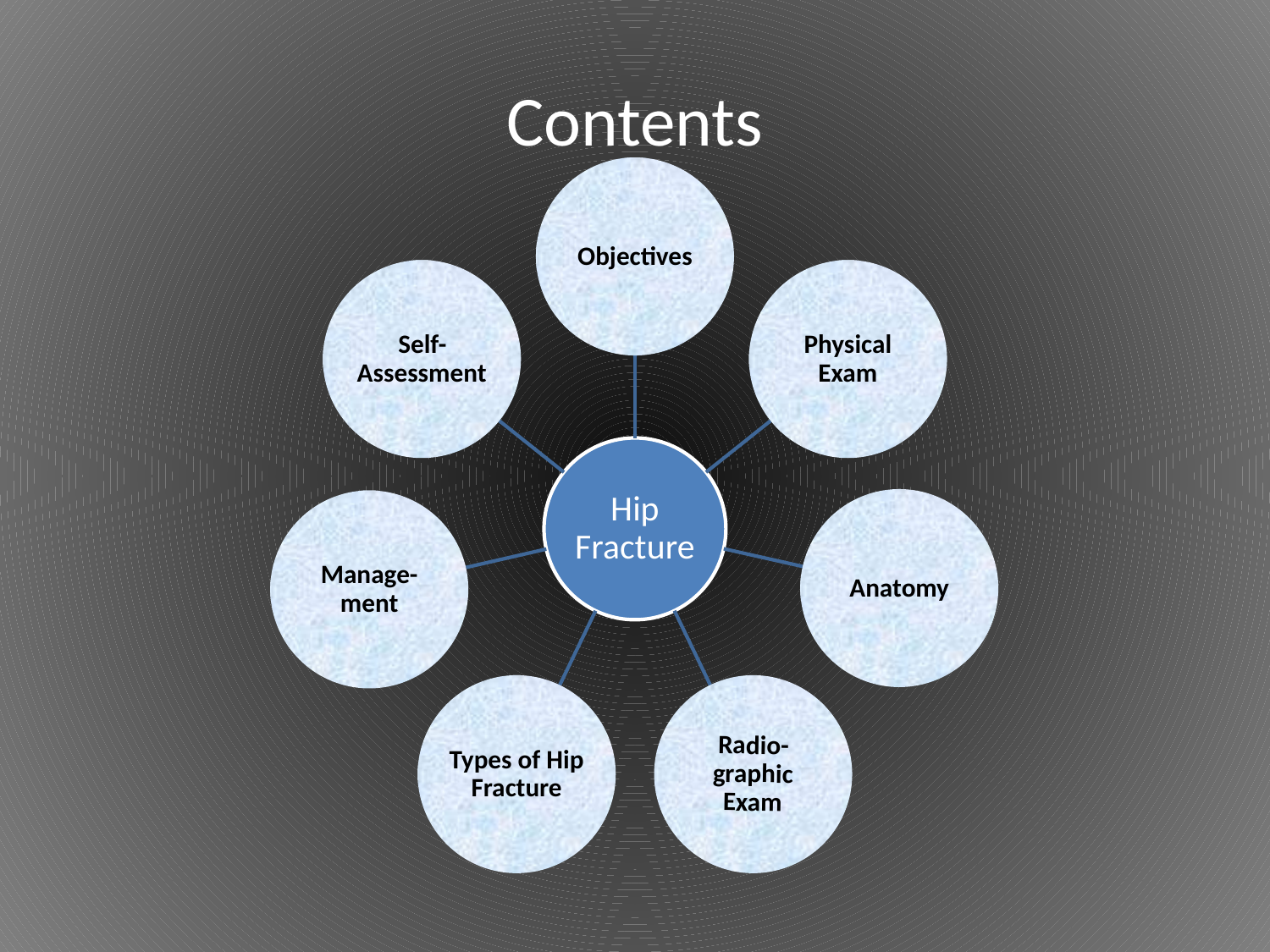

# Contents

## Slide 5
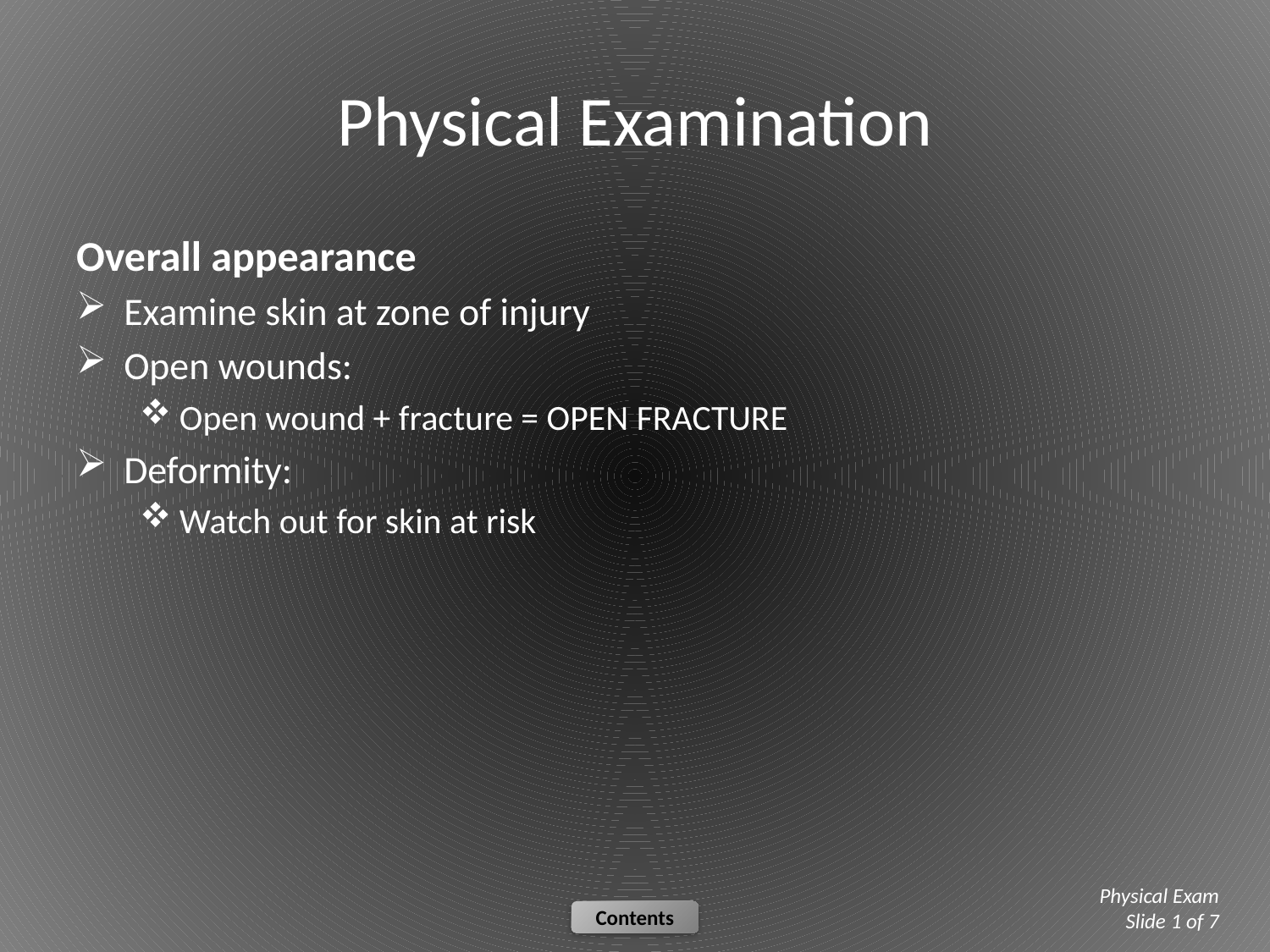

# Physical Examination
Overall appearance
Examine skin at zone of injury
Open wounds:
Open wound + fracture = OPEN FRACTURE
Deformity:
Watch out for skin at risk
Physical Exam
Slide 1 of 7
Contents

## Slide 6
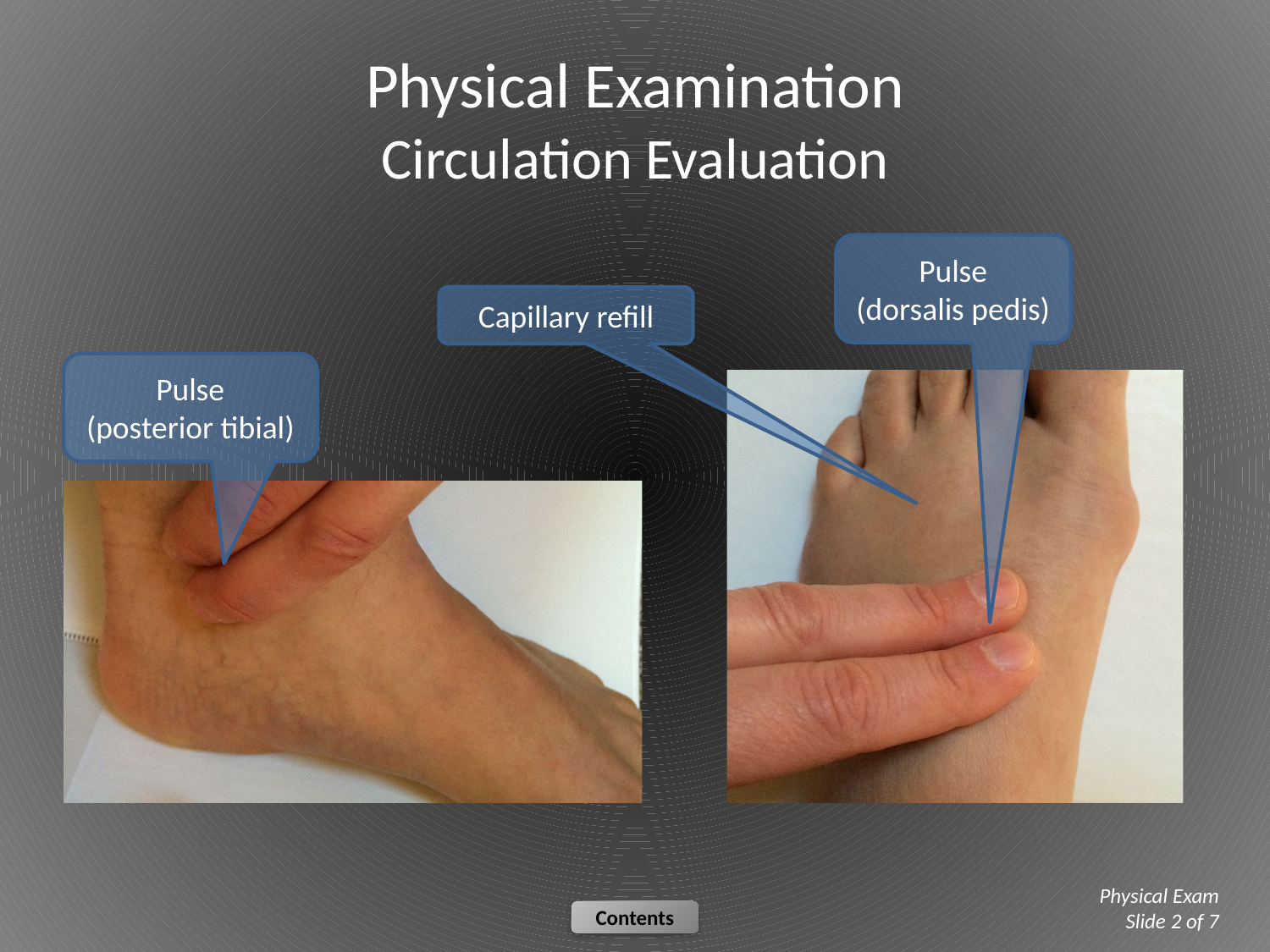

# Physical Examination Circulation Evaluation
Pulse
(dorsalis pedis)
Capillary refill
Pulse
(posterior tibial)
Physical Exam
Slide 2 of 7
Contents

## Slide 7
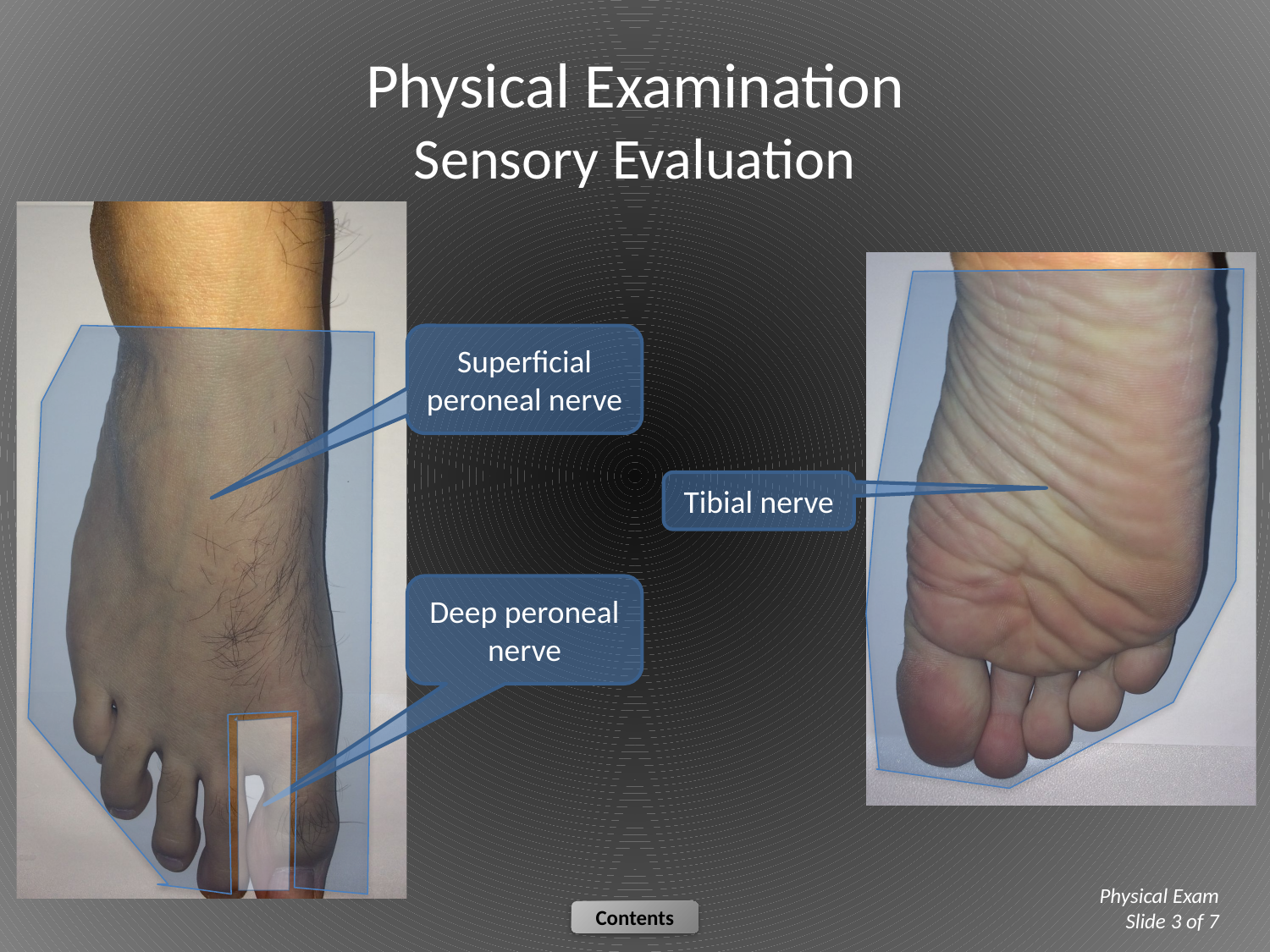

# Physical Examination Sensory Evaluation
Superficial peroneal nerve
Tibial nerve
Deep peroneal nerve
Physical Exam
Slide 3 of 7
Contents

## Slide 8
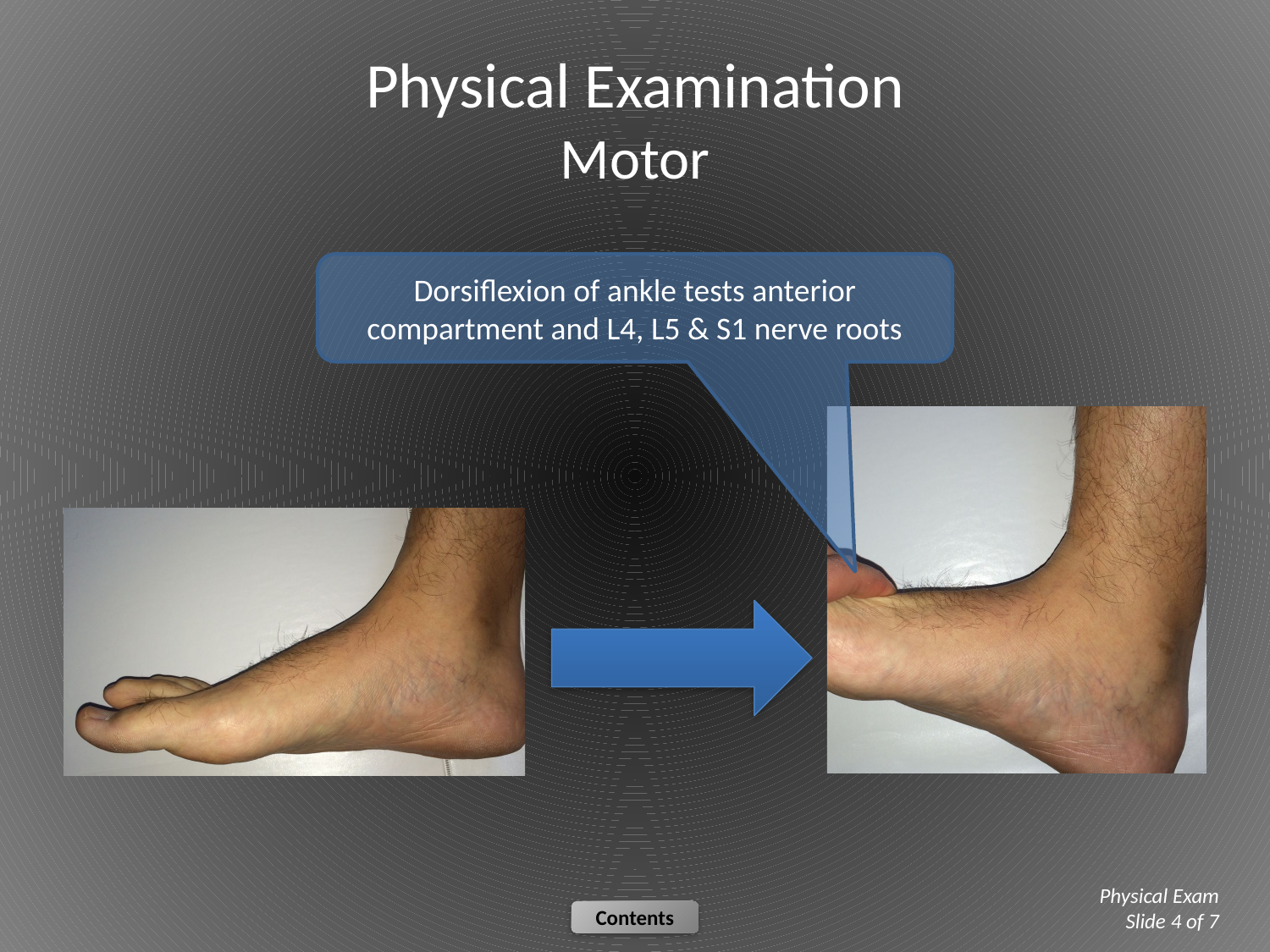

# Physical Examination Motor
Dorsiflexion of ankle tests anterior compartment and L4, L5 & S1 nerve roots
Physical Exam
Slide 4 of 7
Contents

## Slide 9
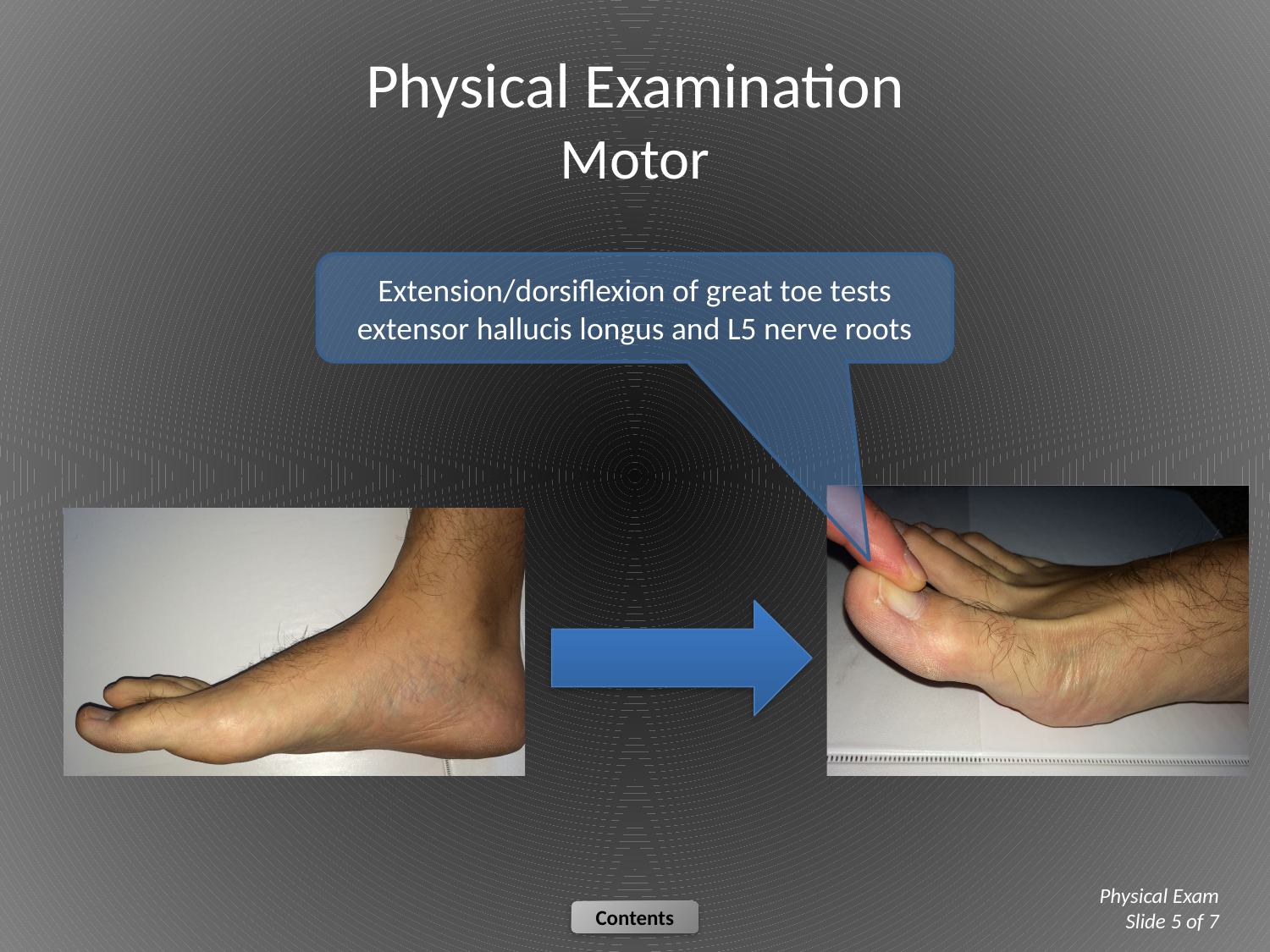

# Physical Examination Motor
Extension/dorsiflexion of great toe tests extensor hallucis longus and L5 nerve roots
Physical Exam
Slide 5 of 7
Contents

## Slide 10
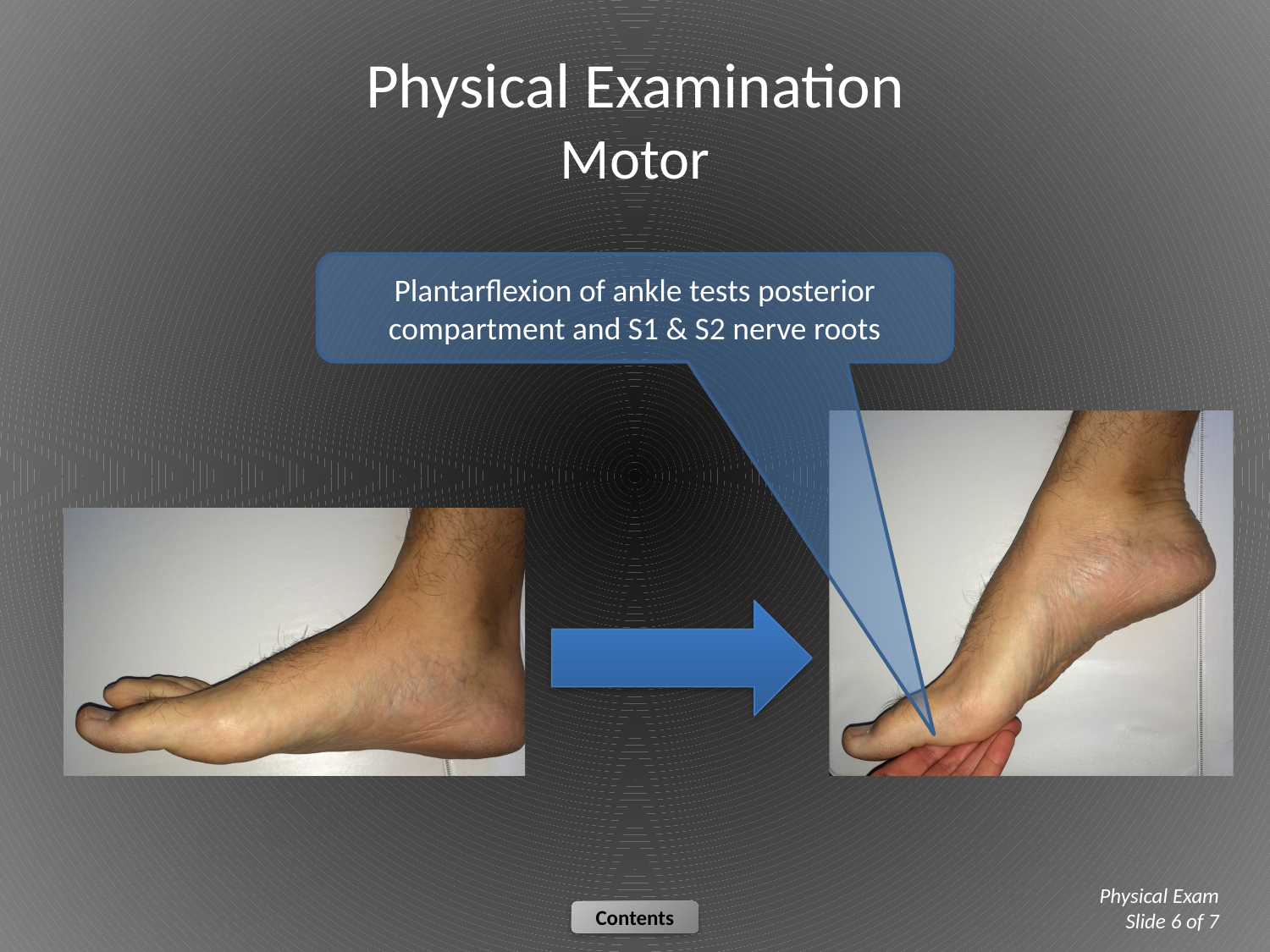

# Physical Examination Motor
Plantarflexion of ankle tests posterior compartment and S1 & S2 nerve roots
Physical Exam
Slide 6 of 7
Contents

## Slide 11
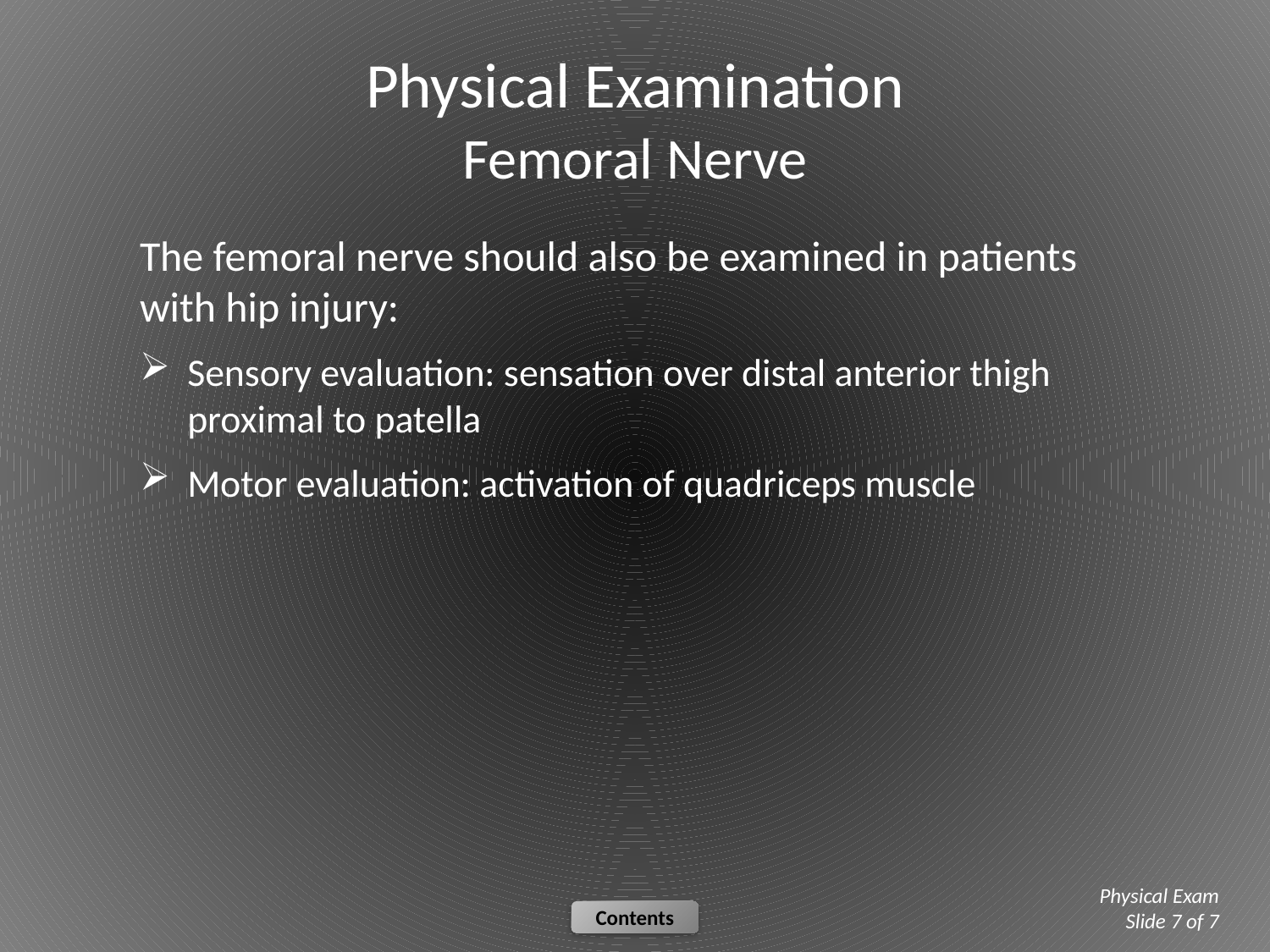

# Physical Examination Femoral Nerve
The femoral nerve should also be examined in patients with hip injury:
Sensory evaluation: sensation over distal anterior thigh proximal to patella
Motor evaluation: activation of quadriceps muscle
Physical Exam
Slide 7 of 7
Contents

## Slide 12
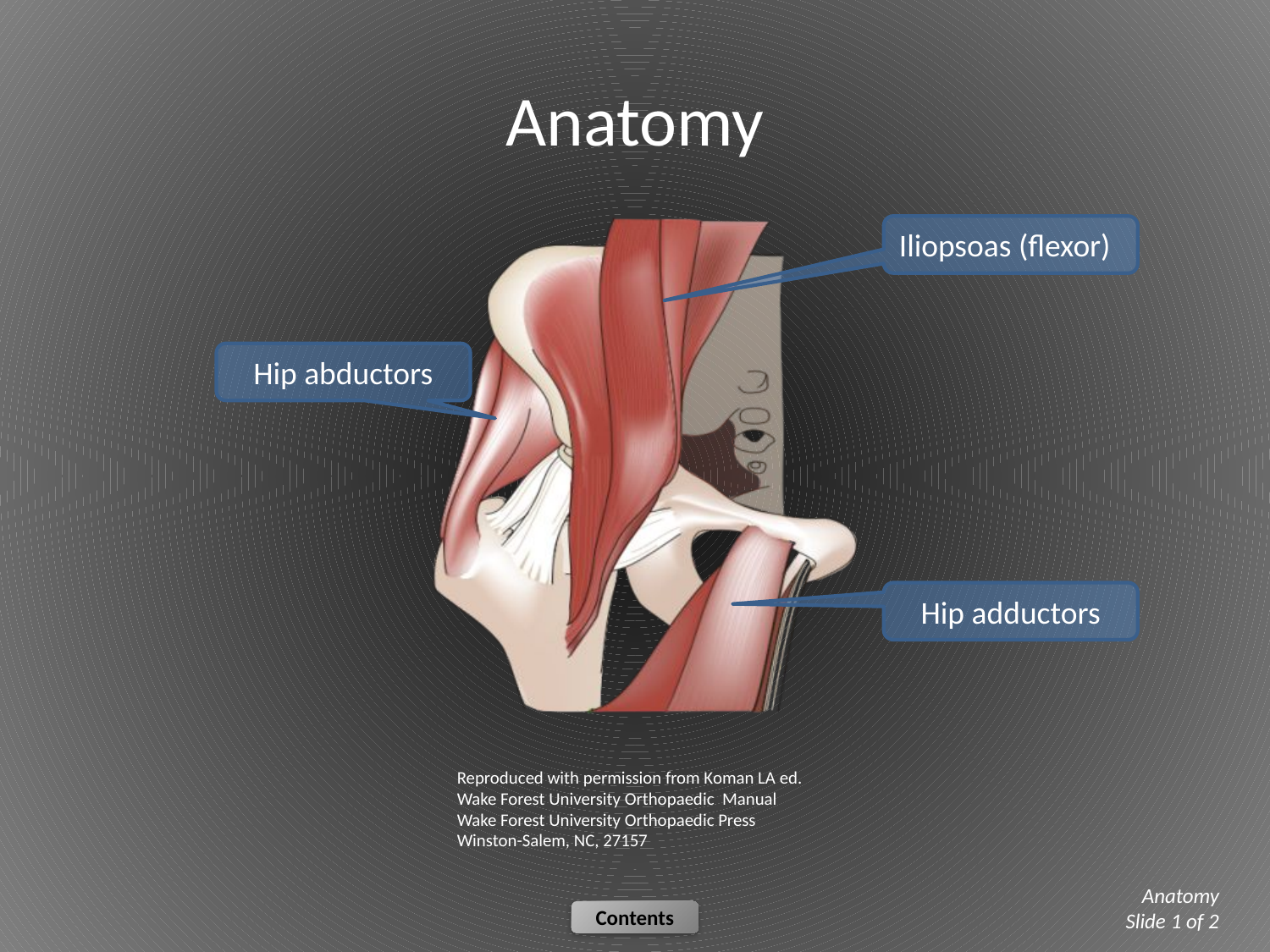

# Anatomy
Iliopsoas (flexor)
Hip abductors
Hip adductors
Reproduced with permission from Koman LA ed.
Wake Forest University Orthopaedic Manual
Wake Forest University Orthopaedic Press
Winston-Salem, NC, 27157
Anatomy
Slide 1 of 2
Contents

## Slide 13
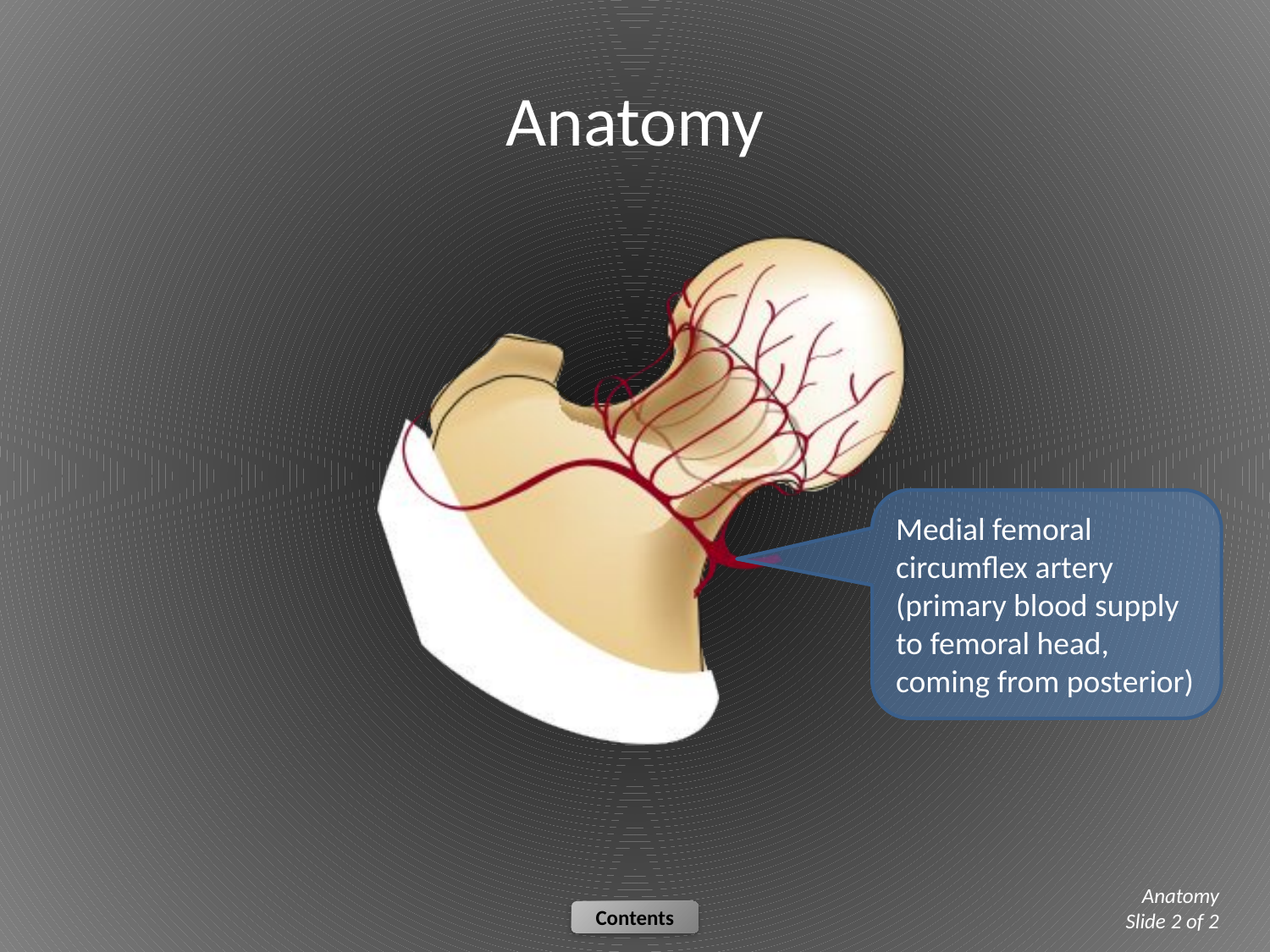

# Anatomy
Medial femoral circumflex artery (primary blood supply to femoral head, coming from posterior)
Anatomy
Slide 2 of 2
Contents

## Slide 14
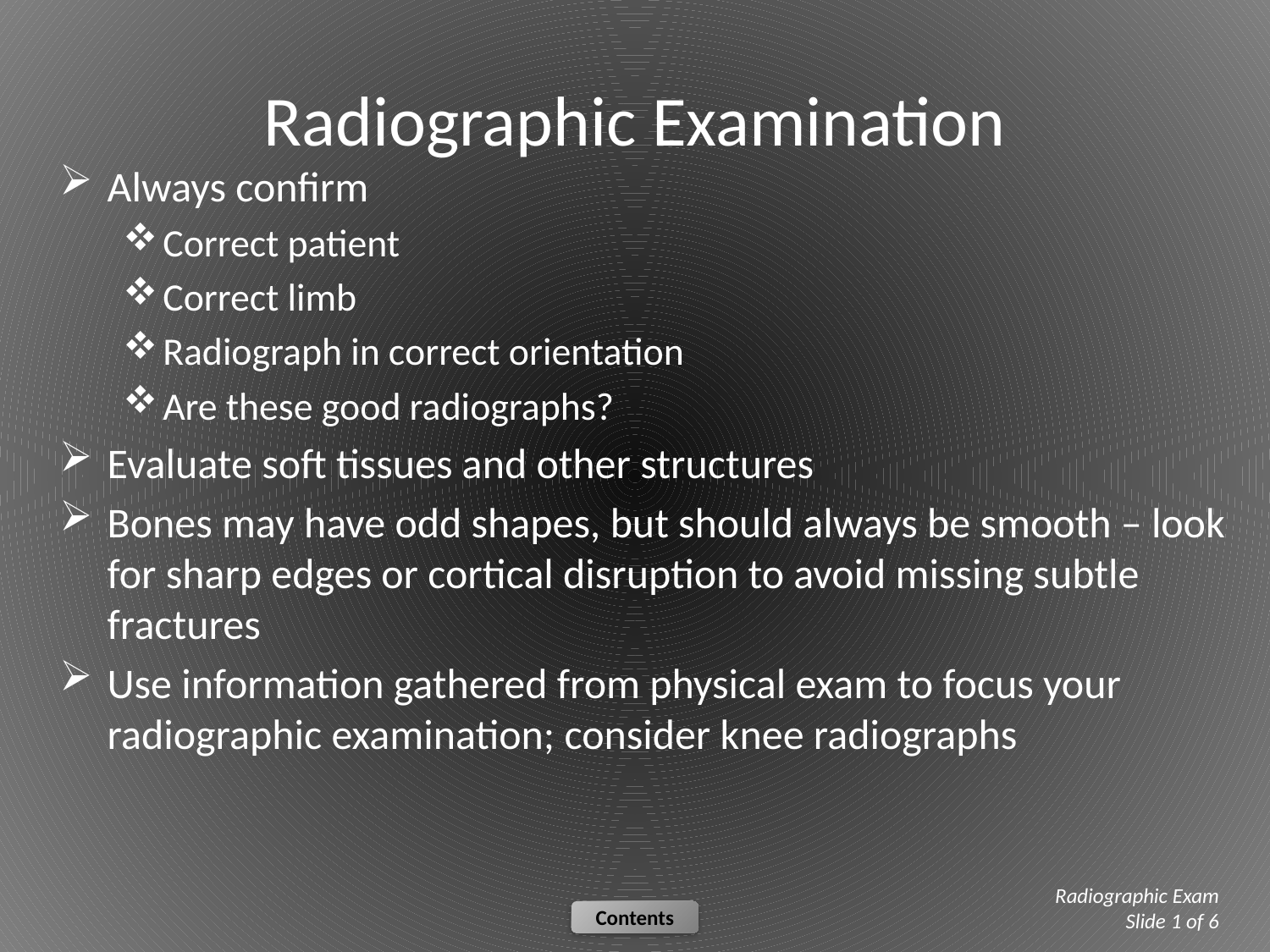

# Radiographic Examination
Always confirm
Correct patient
Correct limb
Radiograph in correct orientation
Are these good radiographs?
Evaluate soft tissues and other structures
Bones may have odd shapes, but should always be smooth – look for sharp edges or cortical disruption to avoid missing subtle fractures
Use information gathered from physical exam to focus your radiographic examination; consider knee radiographs
Radiographic Exam
Slide 1 of 6
Contents

## Slide 15
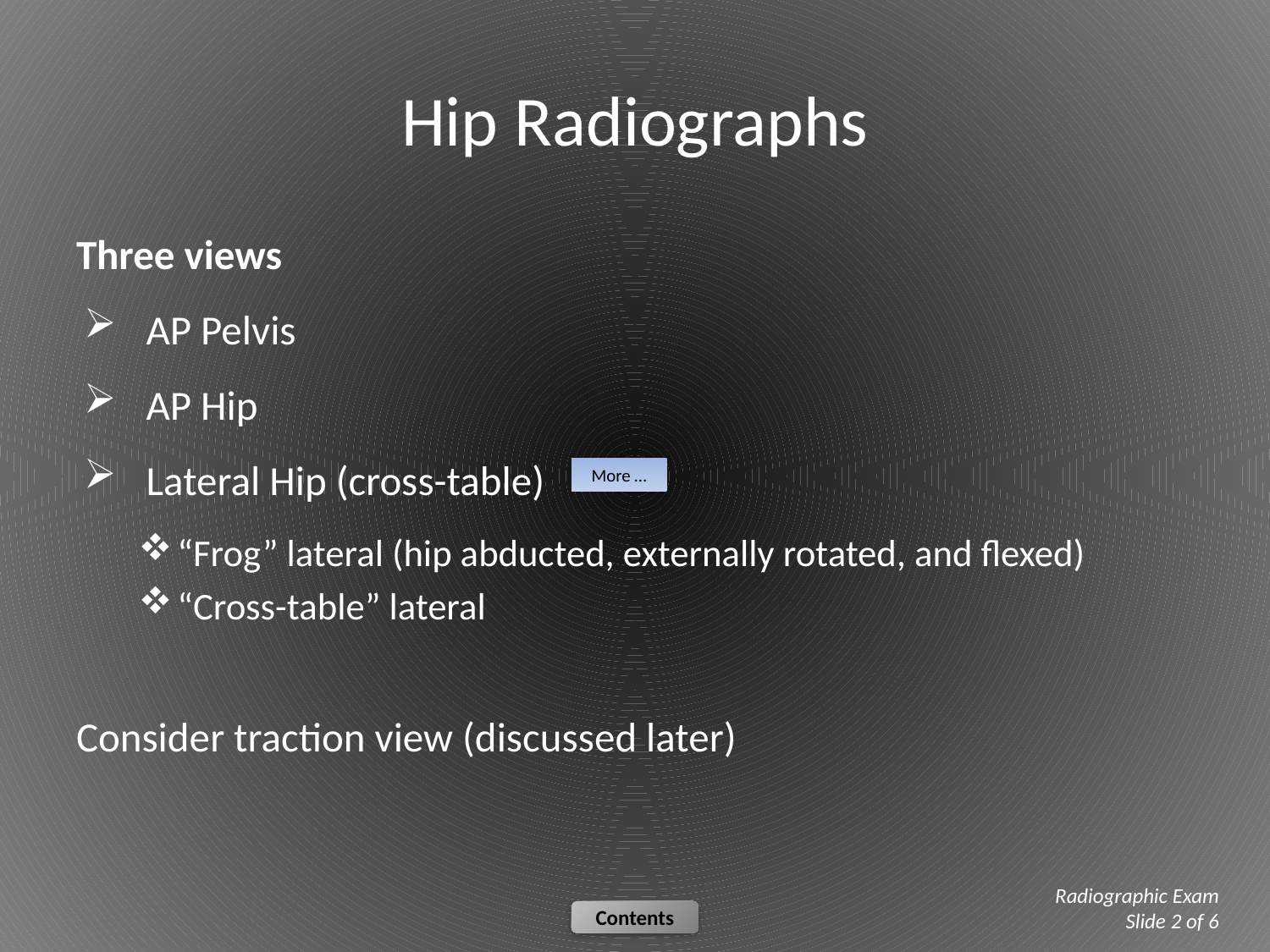

# Hip Radiographs
Three views
AP Pelvis
AP Hip
Lateral Hip (cross-table)
“Frog” lateral (hip abducted, externally rotated, and flexed)
“Cross-table” lateral
Consider traction view (discussed later)
More …
Radiographic Exam
Slide 2 of 6
Contents

## Slide 16
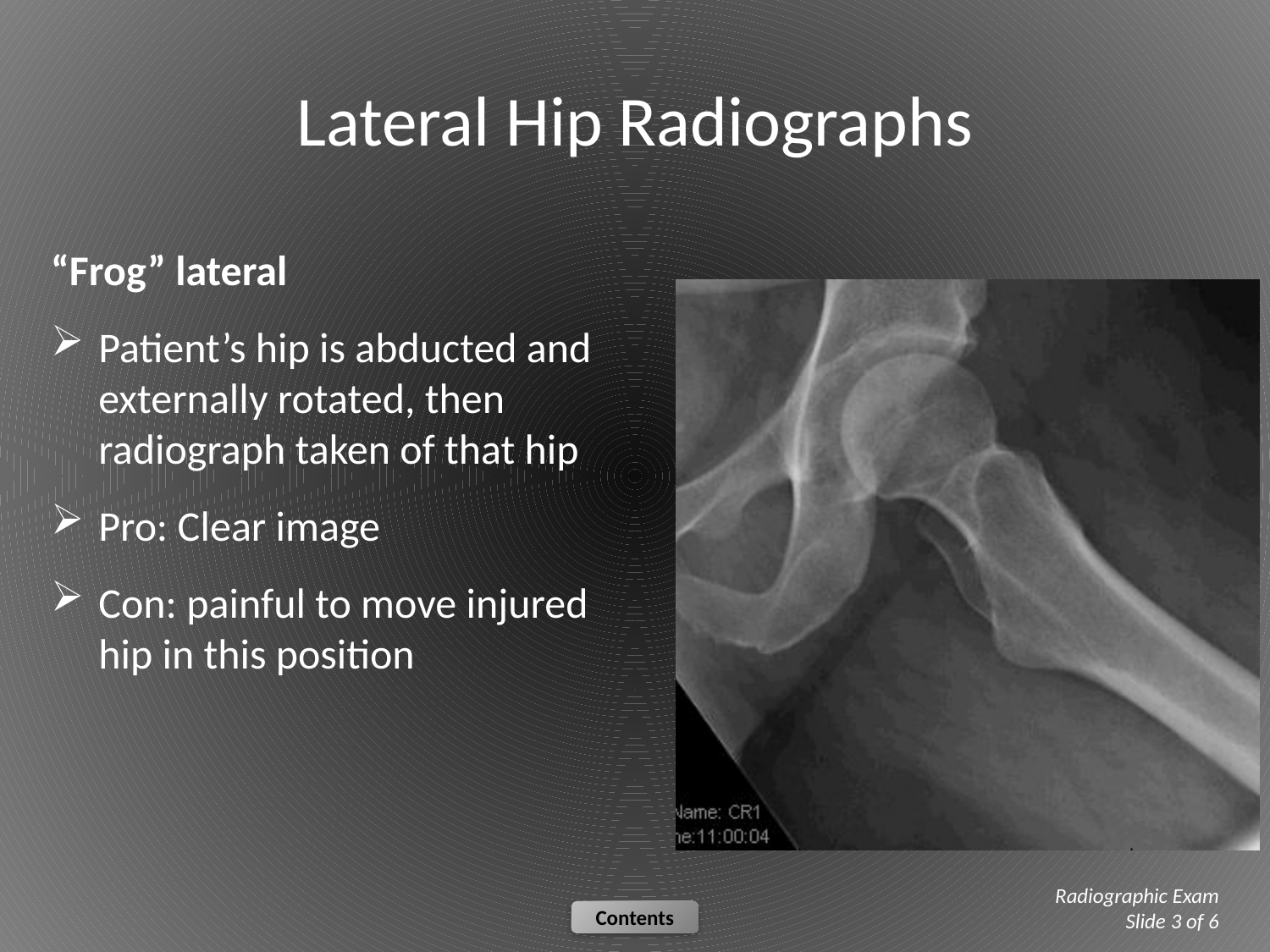

# Lateral Hip Radiographs
“Frog” lateral
Patient’s hip is abducted and externally rotated, then radiograph taken of that hip
Pro: Clear image
Con: painful to move injured hip in this position
Radiographic Exam
Slide 3 of 6
Contents

## Slide 17
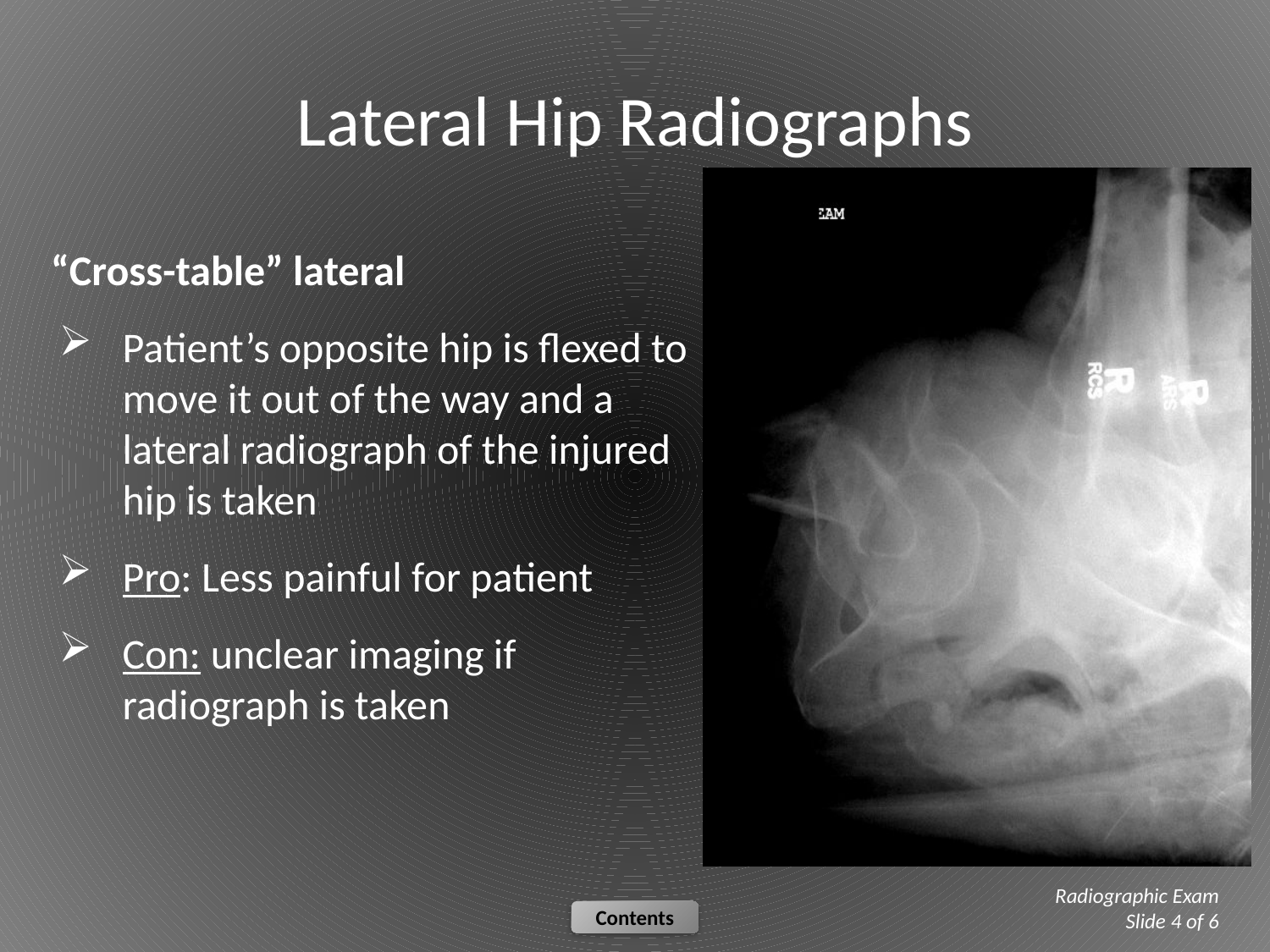

# Lateral Hip Radiographs
“Cross-table” lateral
Patient’s opposite hip is flexed to move it out of the way and a lateral radiograph of the injured hip is taken
Pro: Less painful for patient
Con: unclear imaging if radiograph is taken
Radiographic Exam
Slide 4 of 6
Contents

## Slide 18
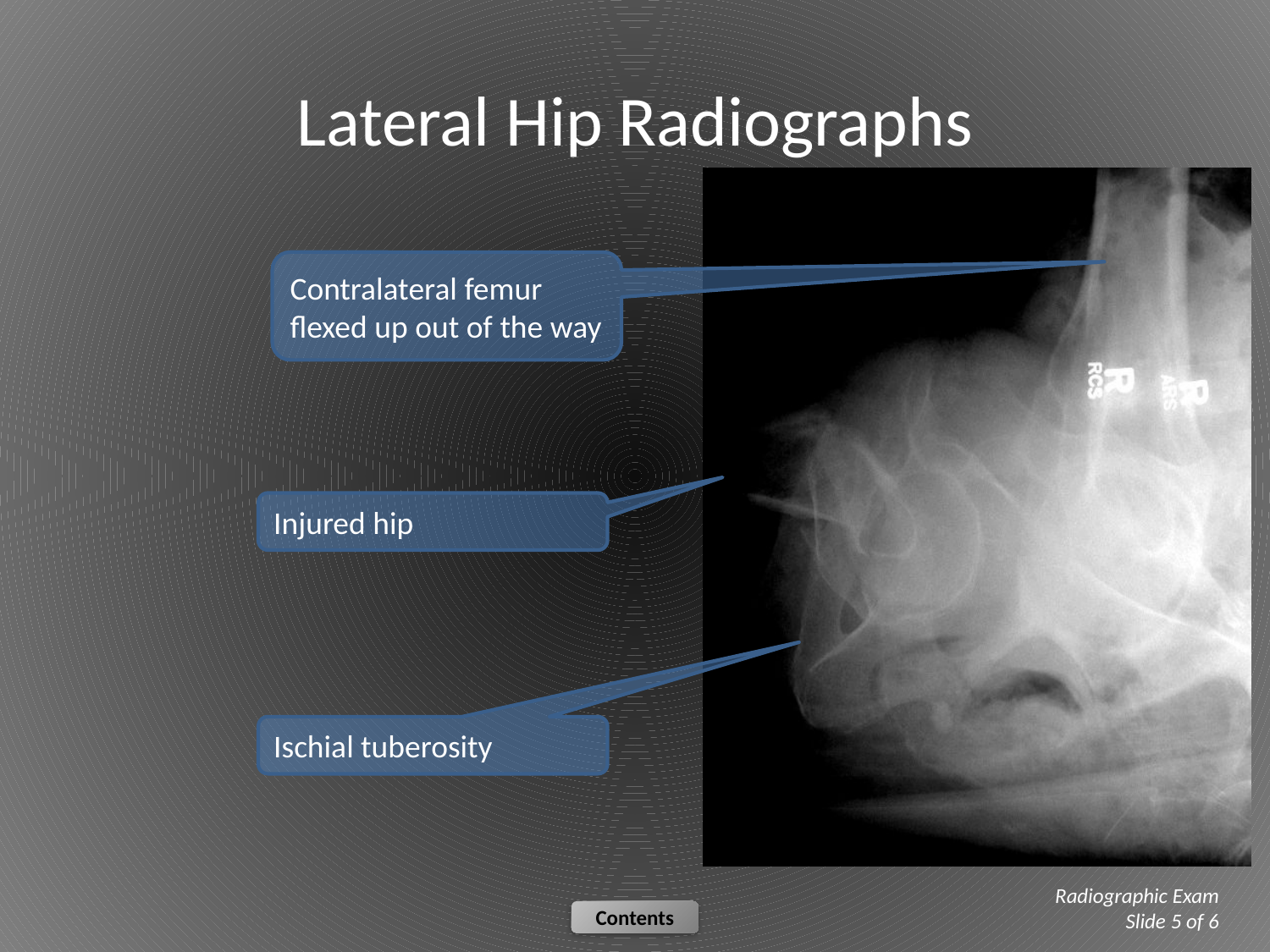

# Lateral Hip Radiographs
Contralateral femur flexed up out of the way
Injured hip
Ischial tuberosity
Radiographic Exam
Slide 5 of 6
Contents

## Slide 19
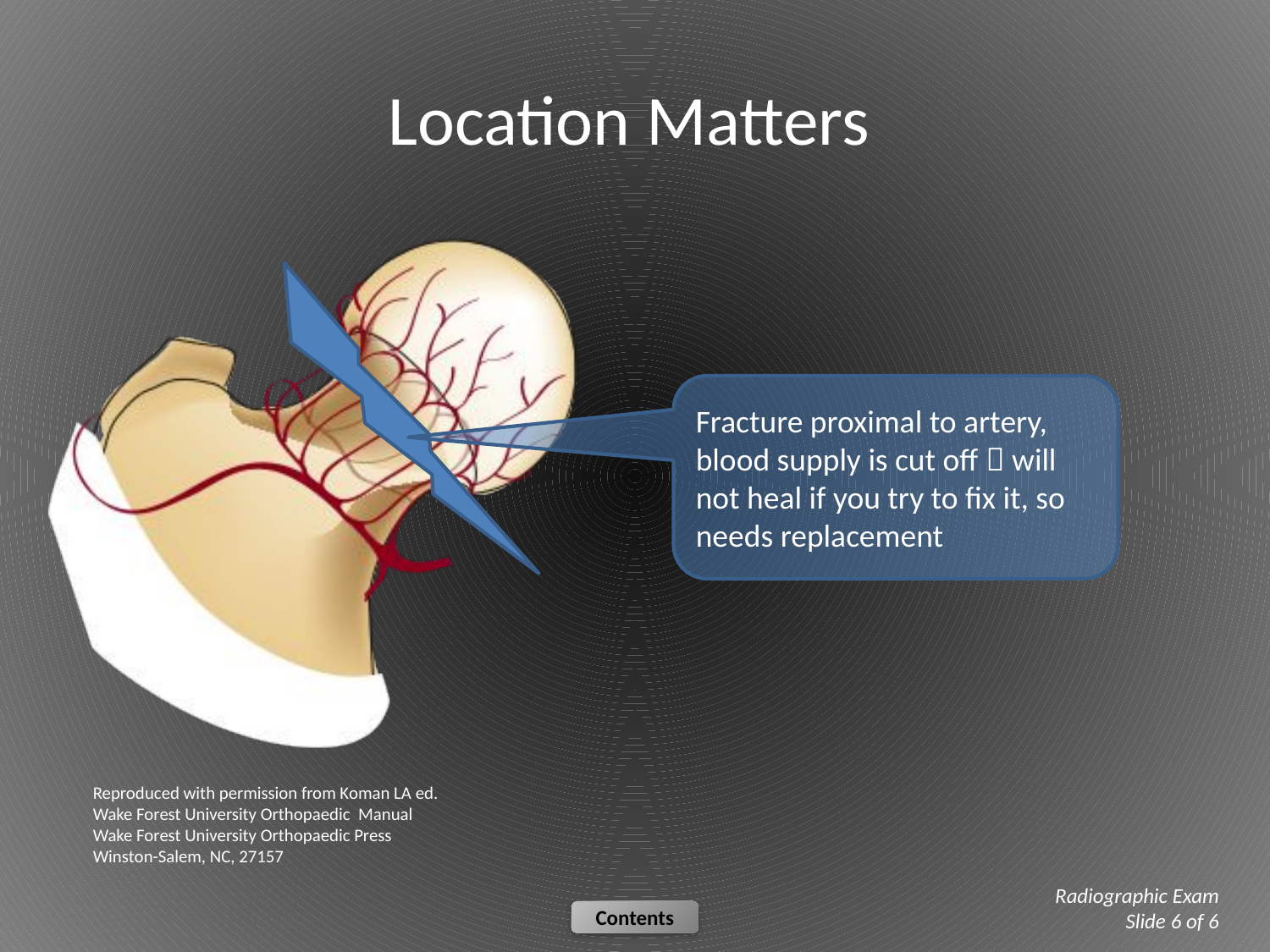

# Location Matters
Fracture proximal to artery, blood supply is cut off  will not heal if you try to fix it, so needs replacement
Reproduced with permission from Koman LA ed.
Wake Forest University Orthopaedic Manual
Wake Forest University Orthopaedic Press
Winston-Salem, NC, 27157
Radiographic Exam
Slide 6 of 6
Contents

## Slide 20
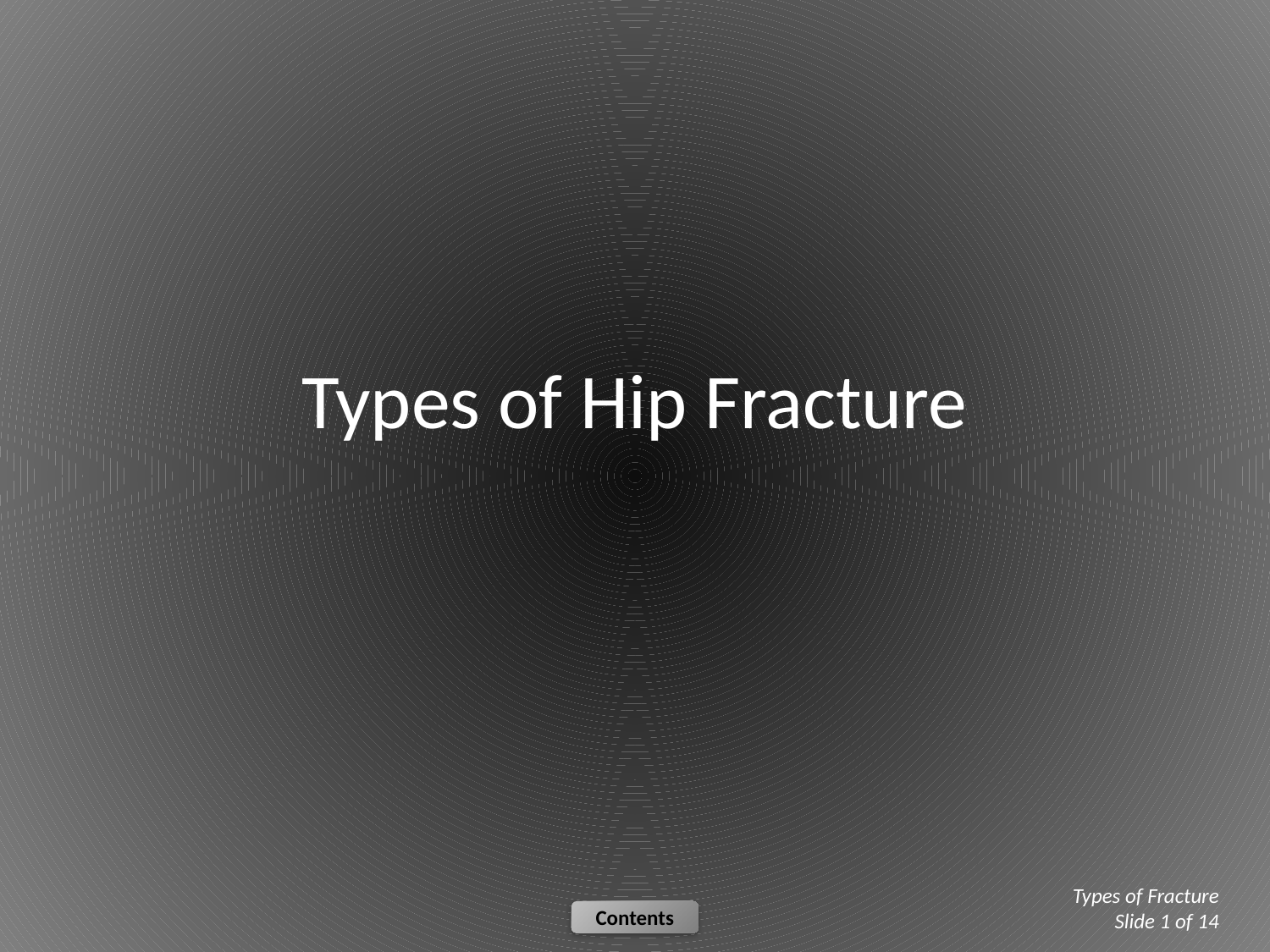

# Types of Hip Fracture
Types of Fracture
Slide 1 of 14
Contents

## Slide 21
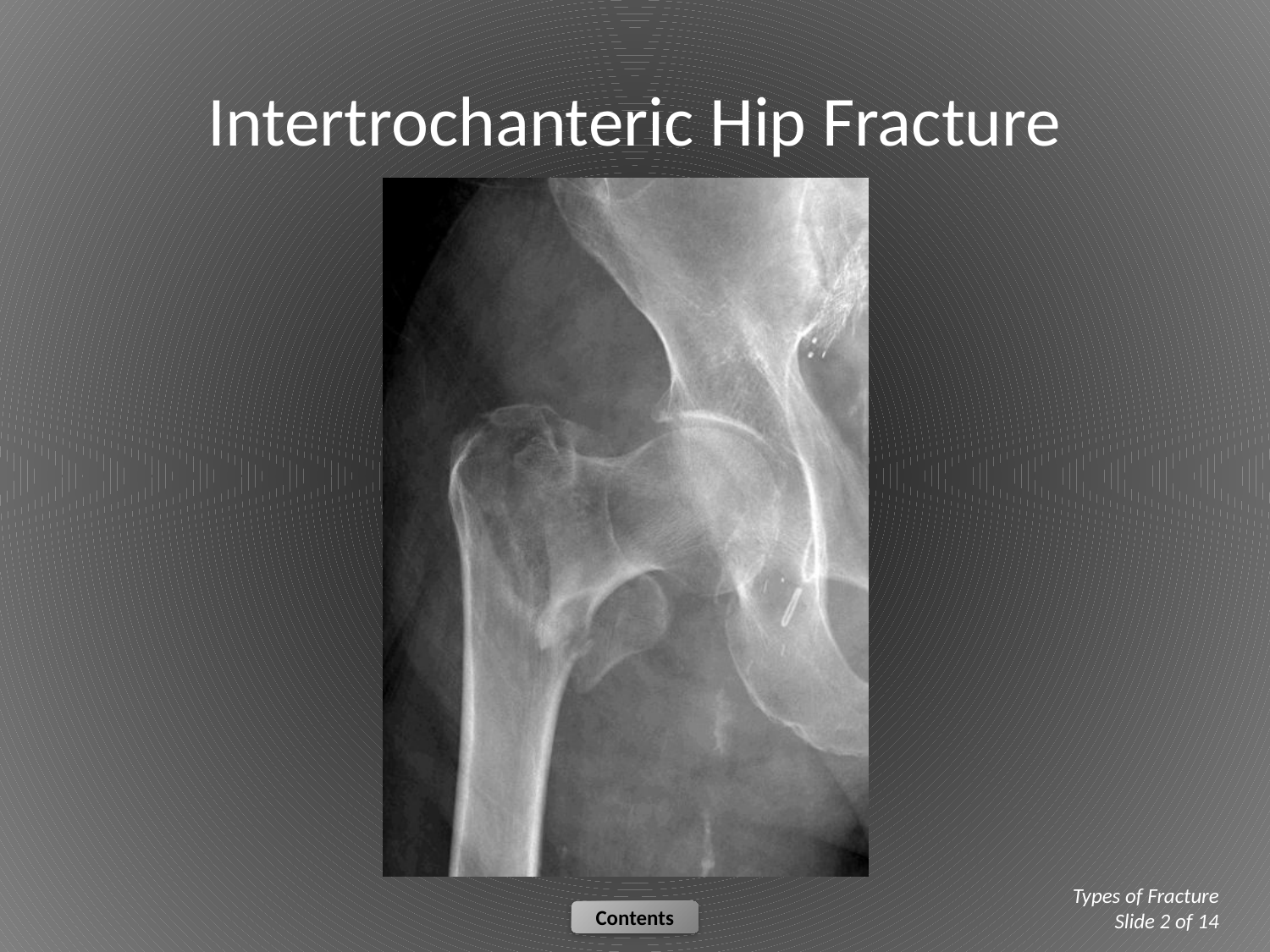

# Intertrochanteric Hip Fracture
Types of Fracture
Slide 2 of 14
Contents

## Slide 22
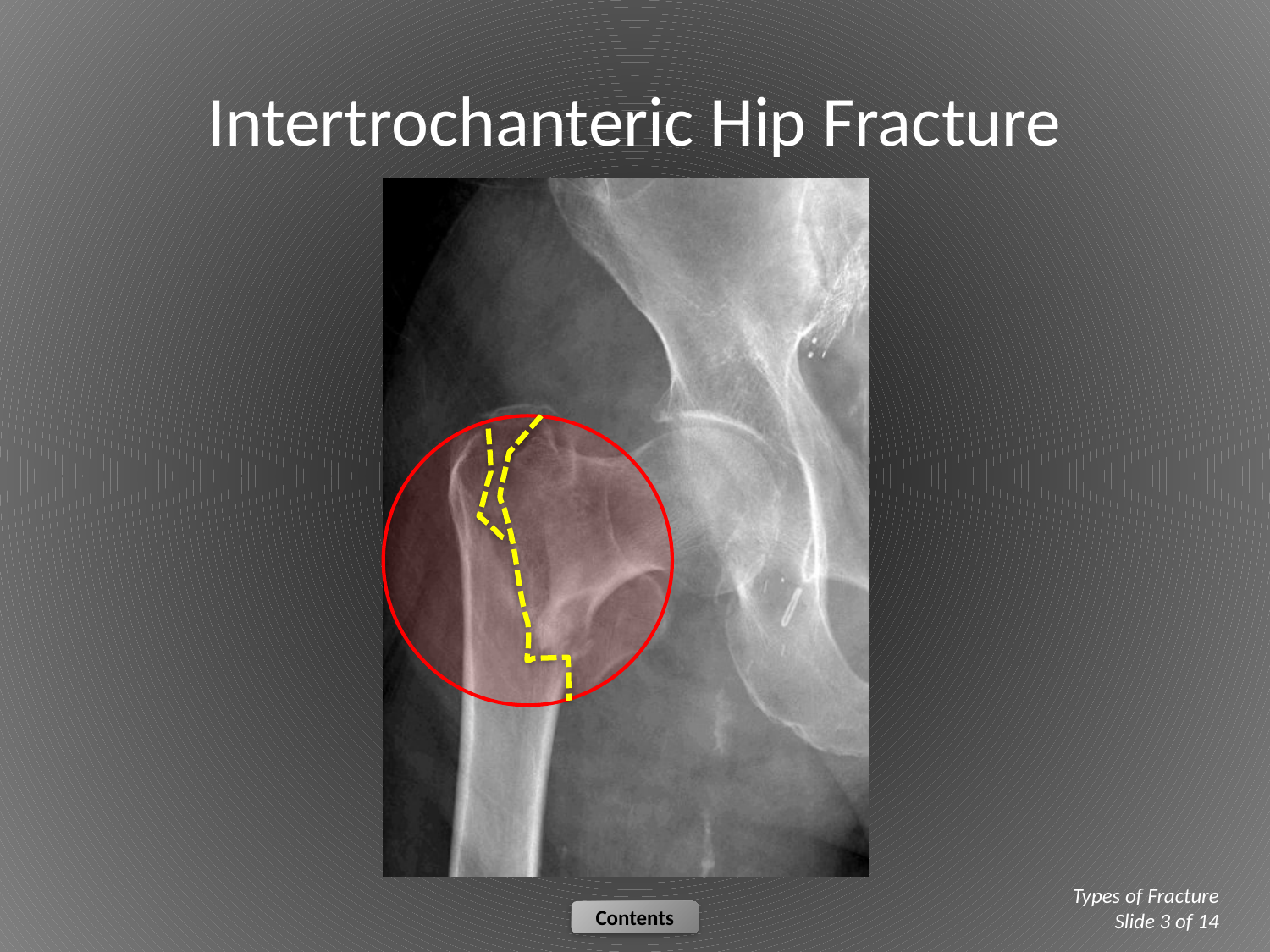

# Intertrochanteric Hip Fracture
Types of Fracture
Slide 3 of 14
Contents

## Slide 23
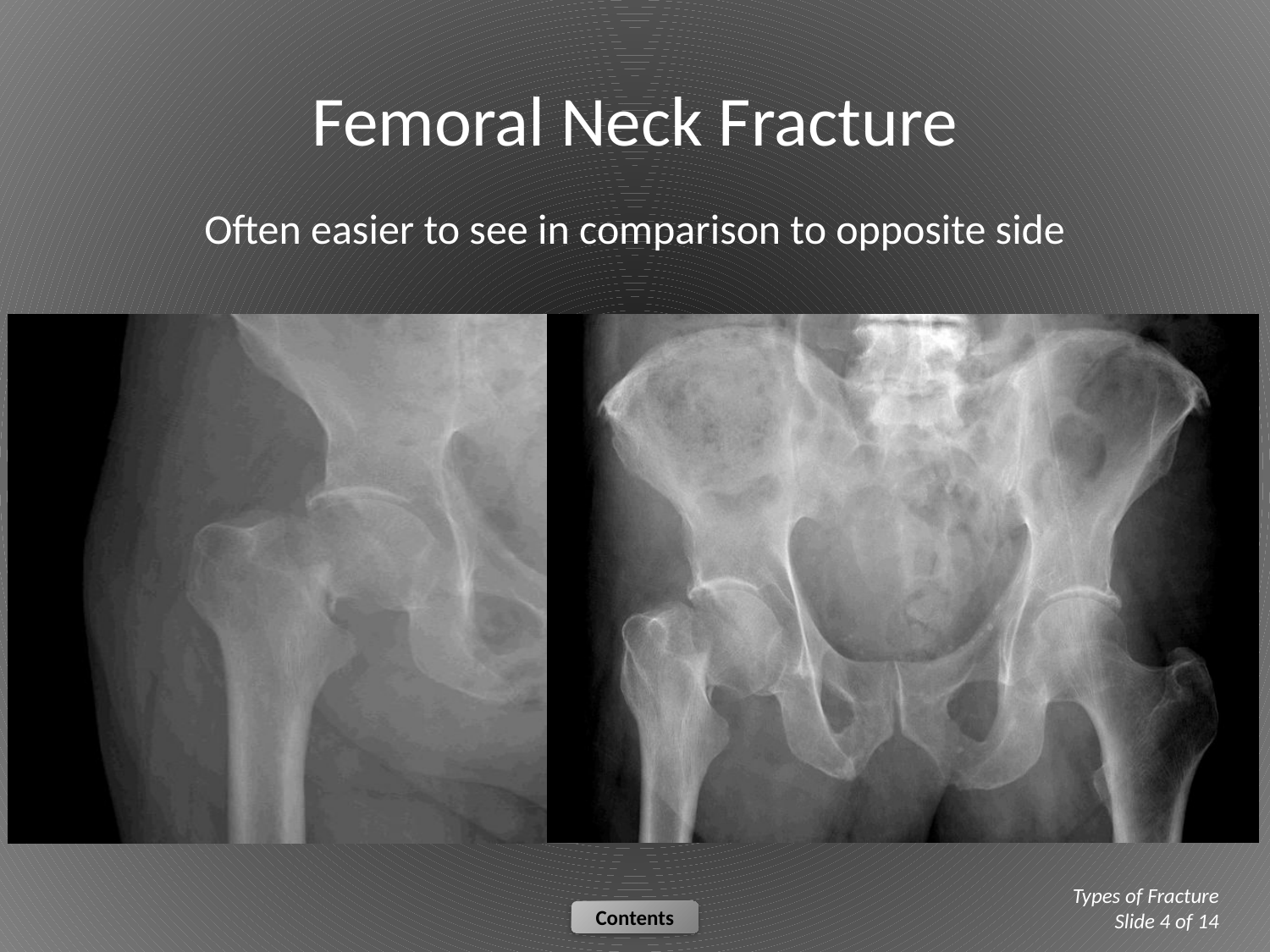

# Femoral Neck Fracture
Often easier to see in comparison to opposite side
Types of Fracture
Slide 4 of 14
Contents

## Slide 24
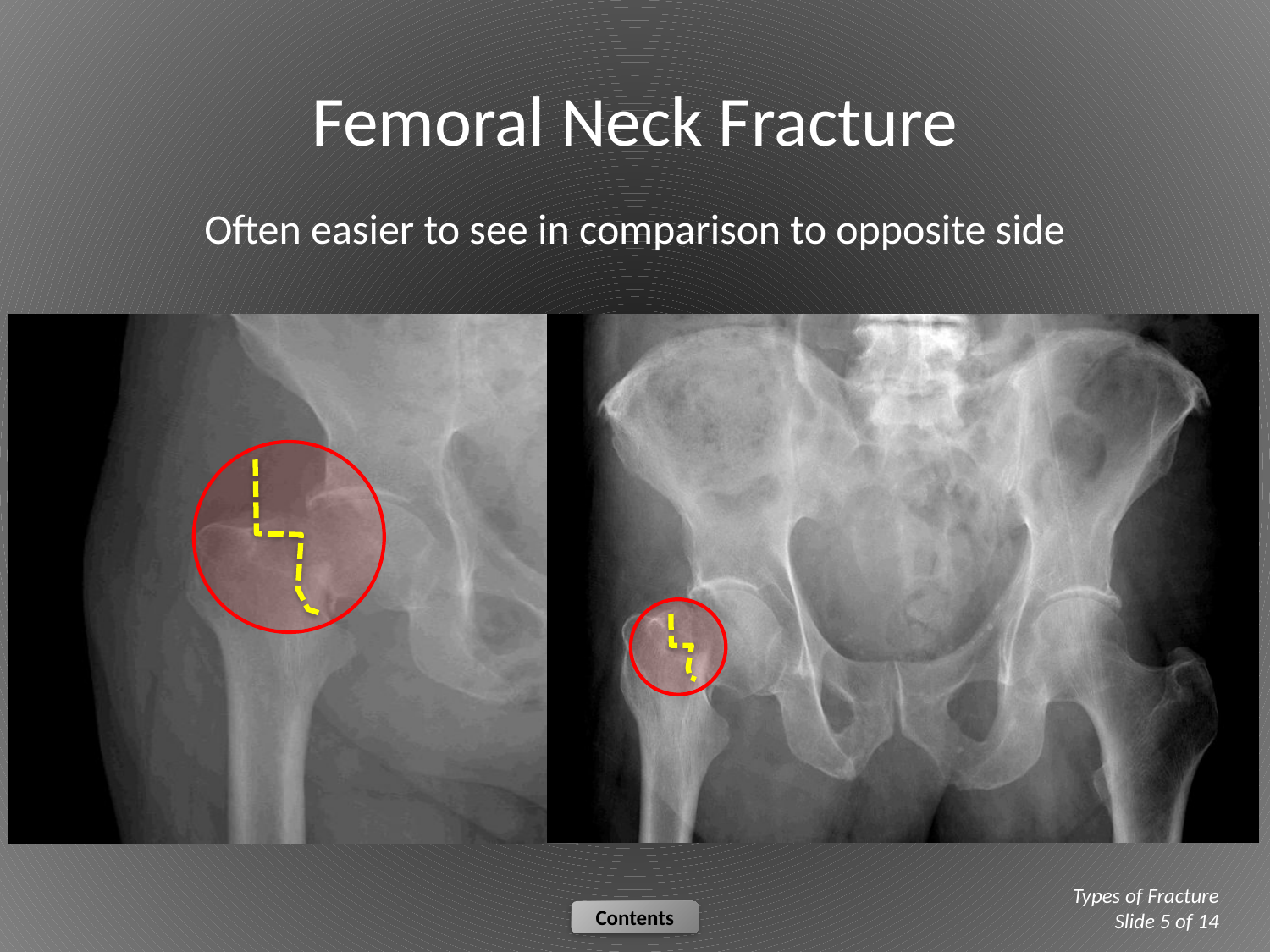

# Femoral Neck Fracture
Often easier to see in comparison to opposite side
Types of Fracture
Slide 5 of 14
Contents

## Slide 25
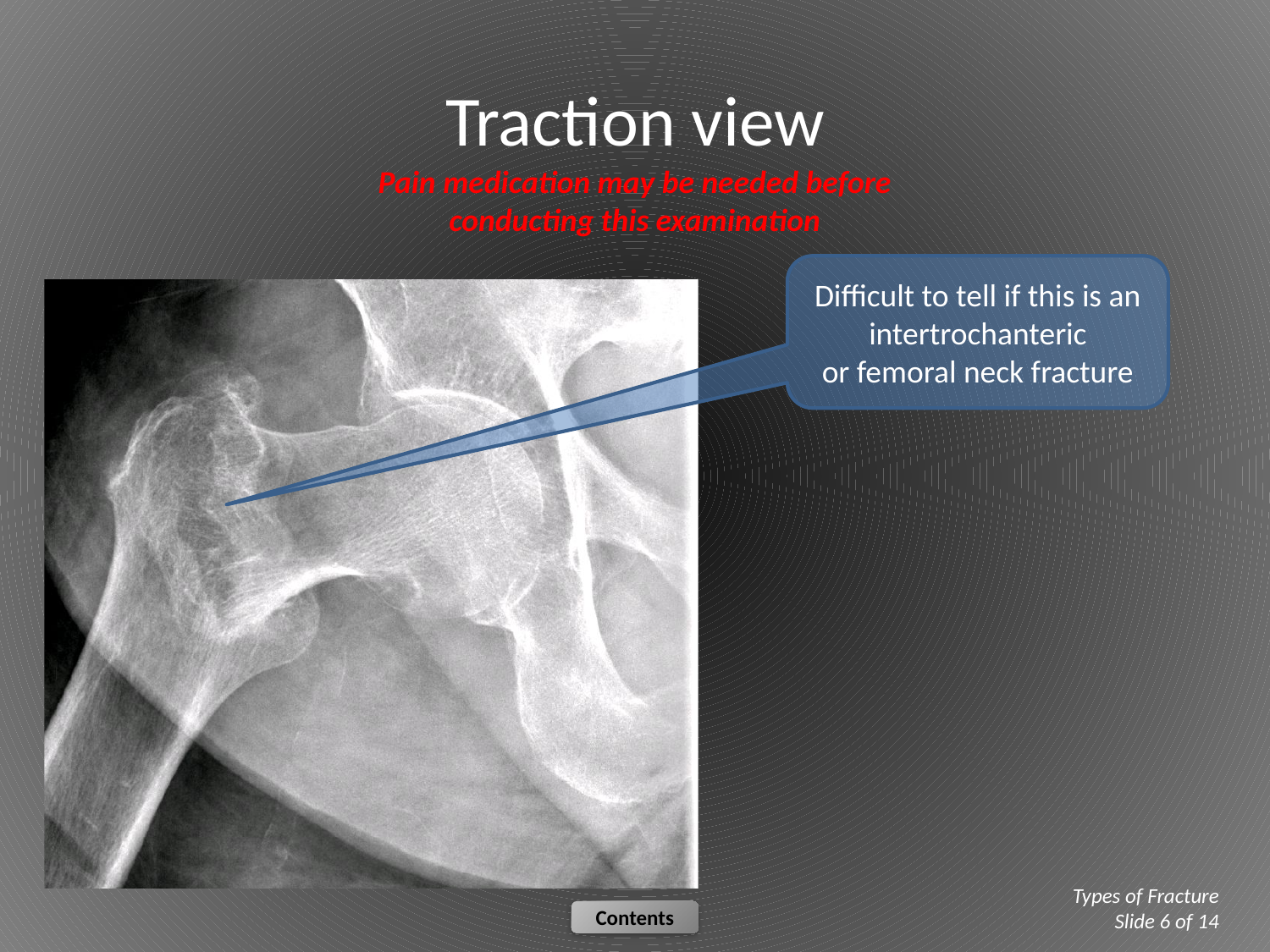

# Traction view
Pain medication may be needed before conducting this examination
Difficult to tell if this is an intertrochanteric
or femoral neck fracture
Types of Fracture
Slide 6 of 14
Contents

## Slide 26
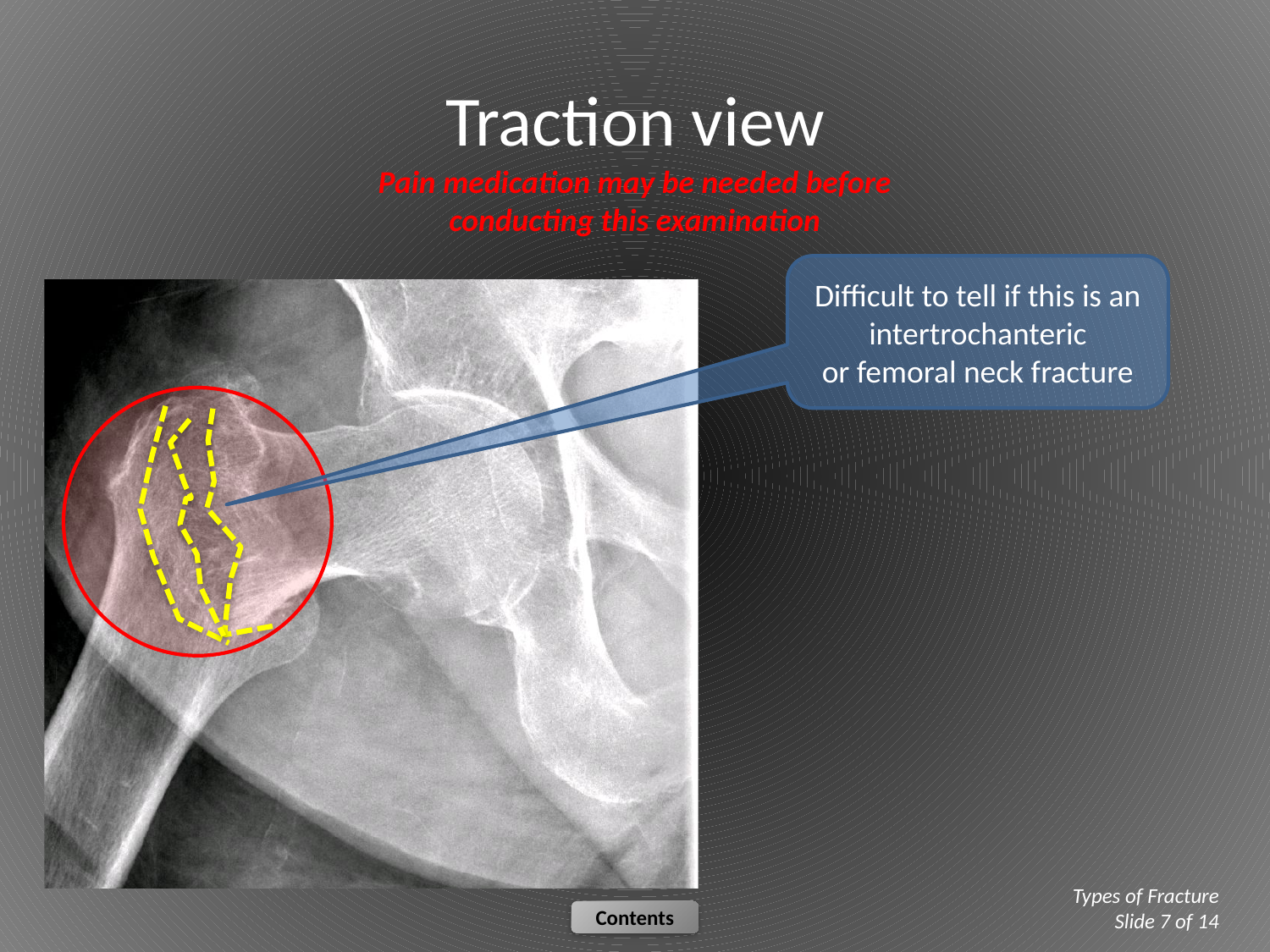

# Traction view
Pain medication may be needed before conducting this examination
Difficult to tell if this is an intertrochanteric
or femoral neck fracture
Types of Fracture
Slide 7 of 14
Contents

## Slide 27
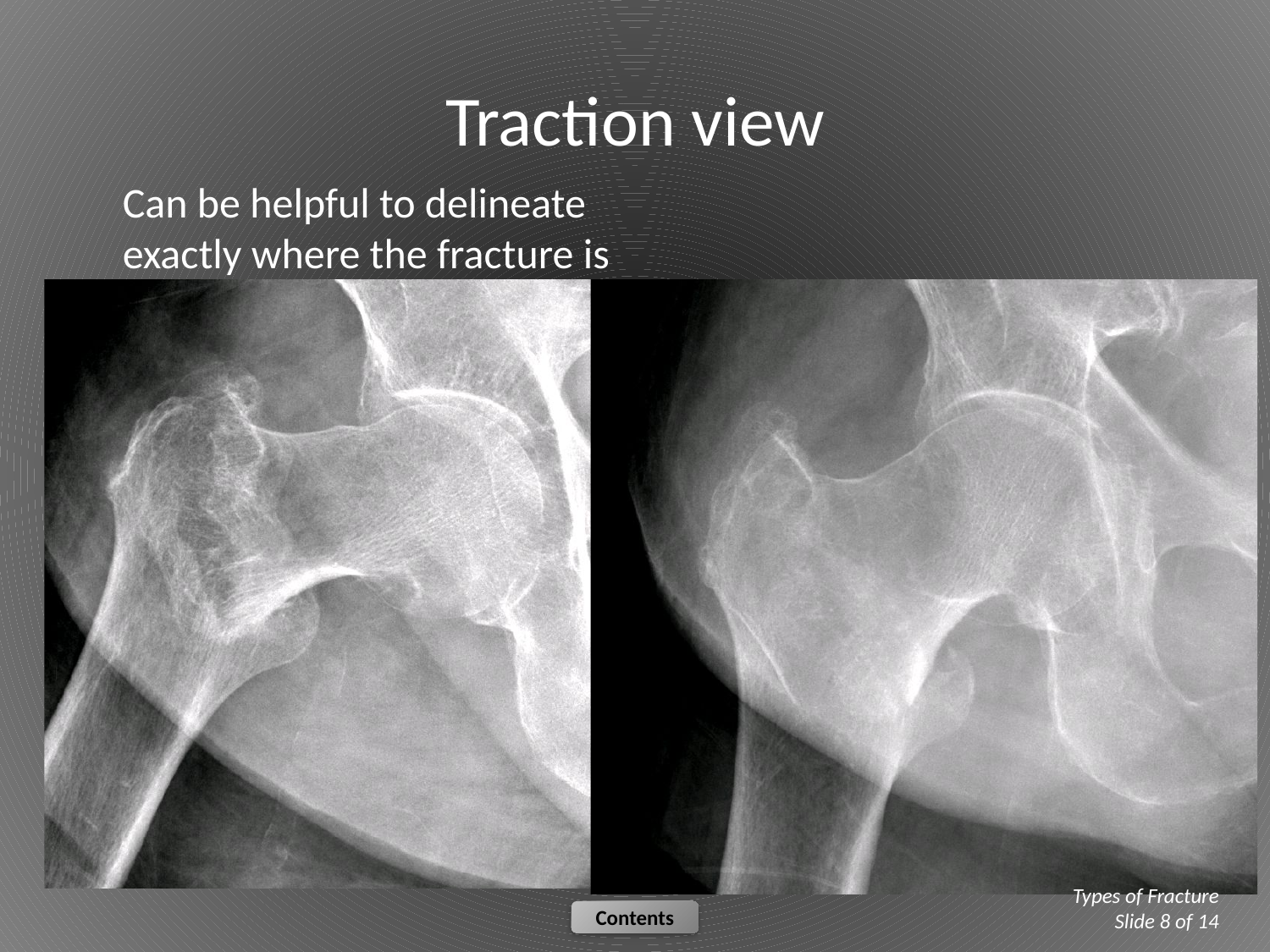

# Traction view
Can be helpful to delineate exactly where the fracture is
Types of Fracture
Slide 8 of 14
Contents

## Slide 28
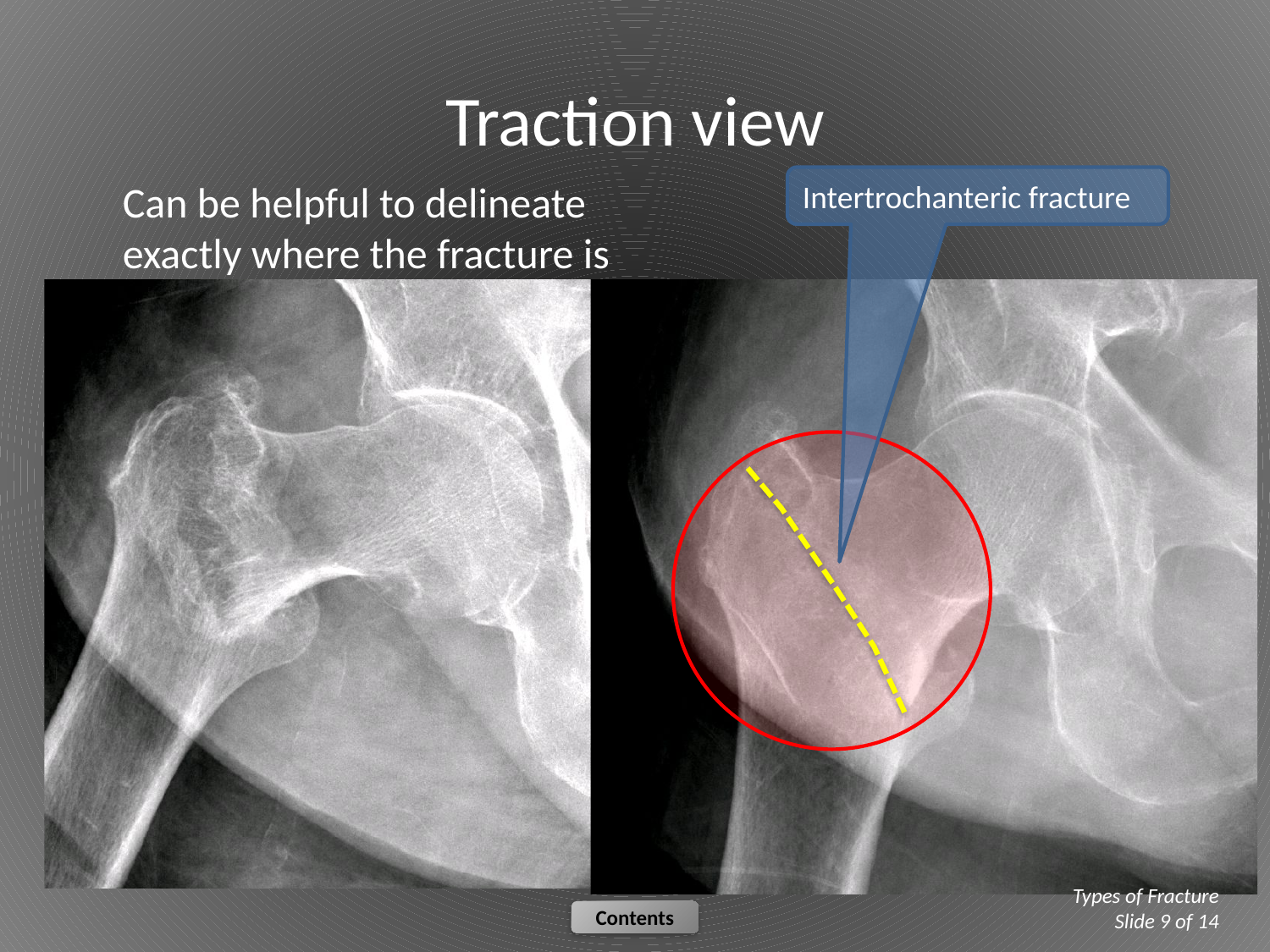

# Traction view
Intertrochanteric fracture
Can be helpful to delineate exactly where the fracture is
Types of Fracture
Slide 9 of 14
Contents

## Slide 29
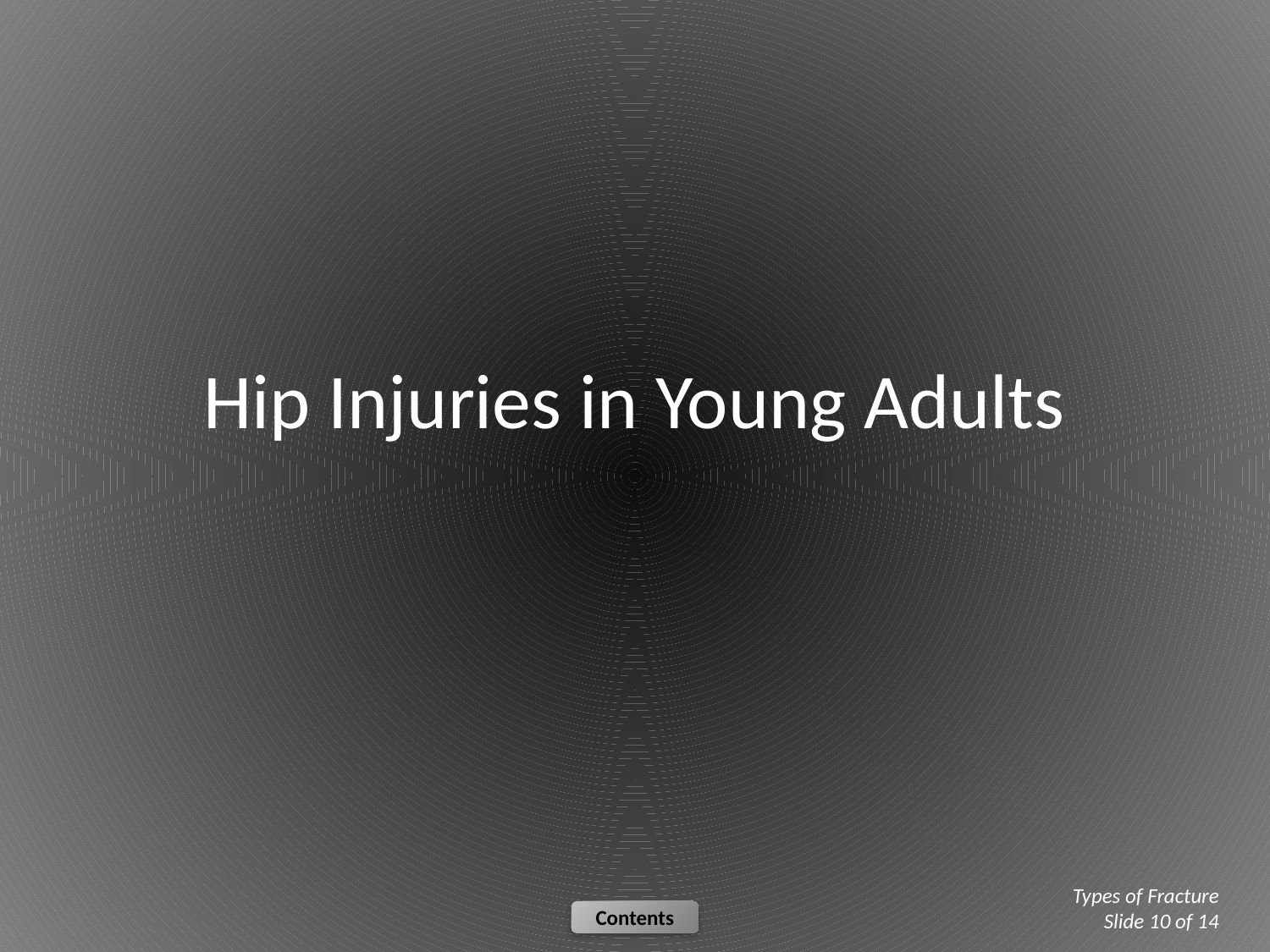

# Hip Injuries in Young Adults
Types of Fracture
Slide 10 of 14
Contents

## Slide 30
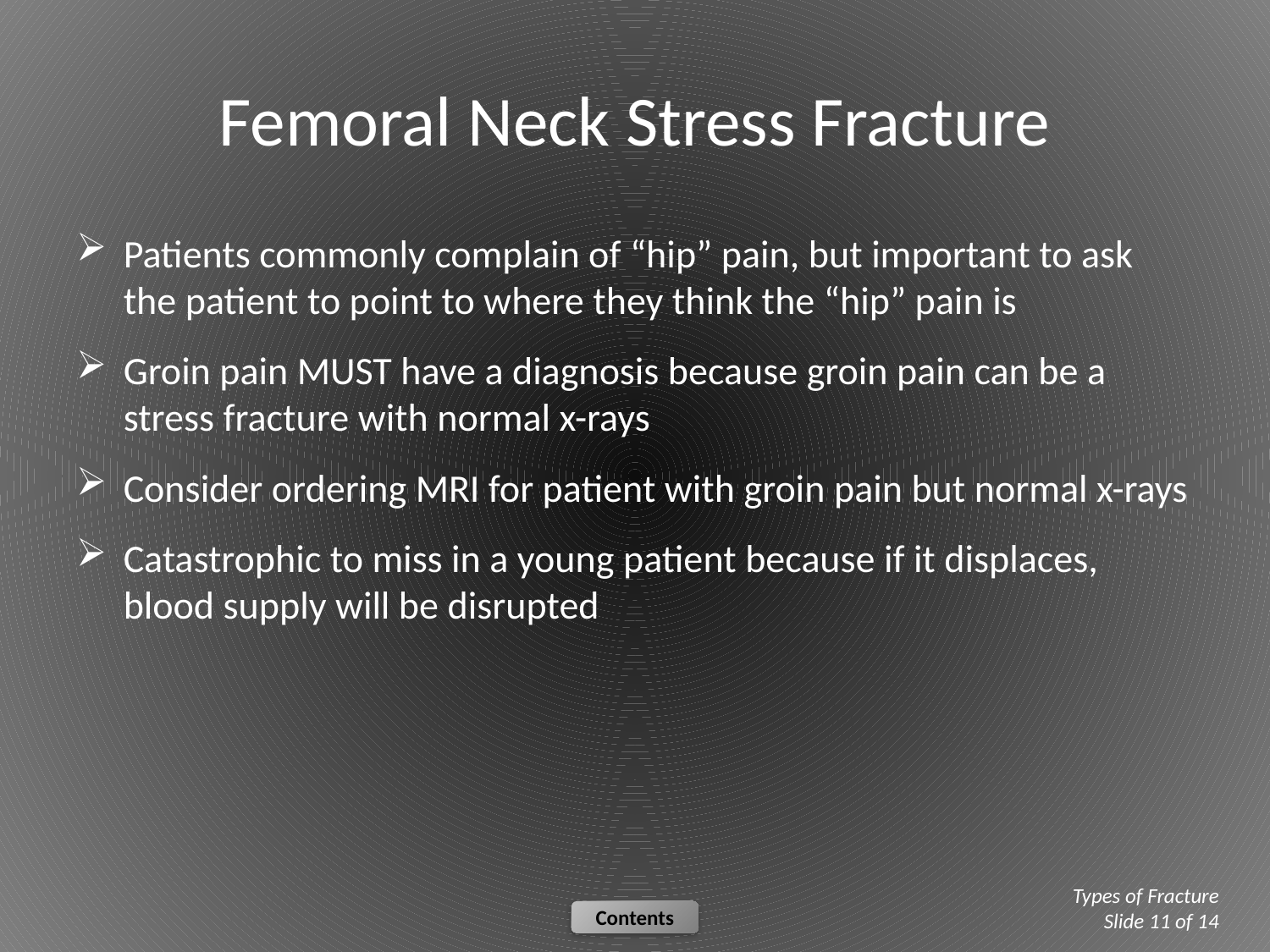

# Femoral Neck Stress Fracture
Patients commonly complain of “hip” pain, but important to ask the patient to point to where they think the “hip” pain is
Groin pain MUST have a diagnosis because groin pain can be a stress fracture with normal x-rays
Consider ordering MRI for patient with groin pain but normal x-rays
Catastrophic to miss in a young patient because if it displaces, blood supply will be disrupted
Types of Fracture
Slide 11 of 14
Contents

## Slide 31
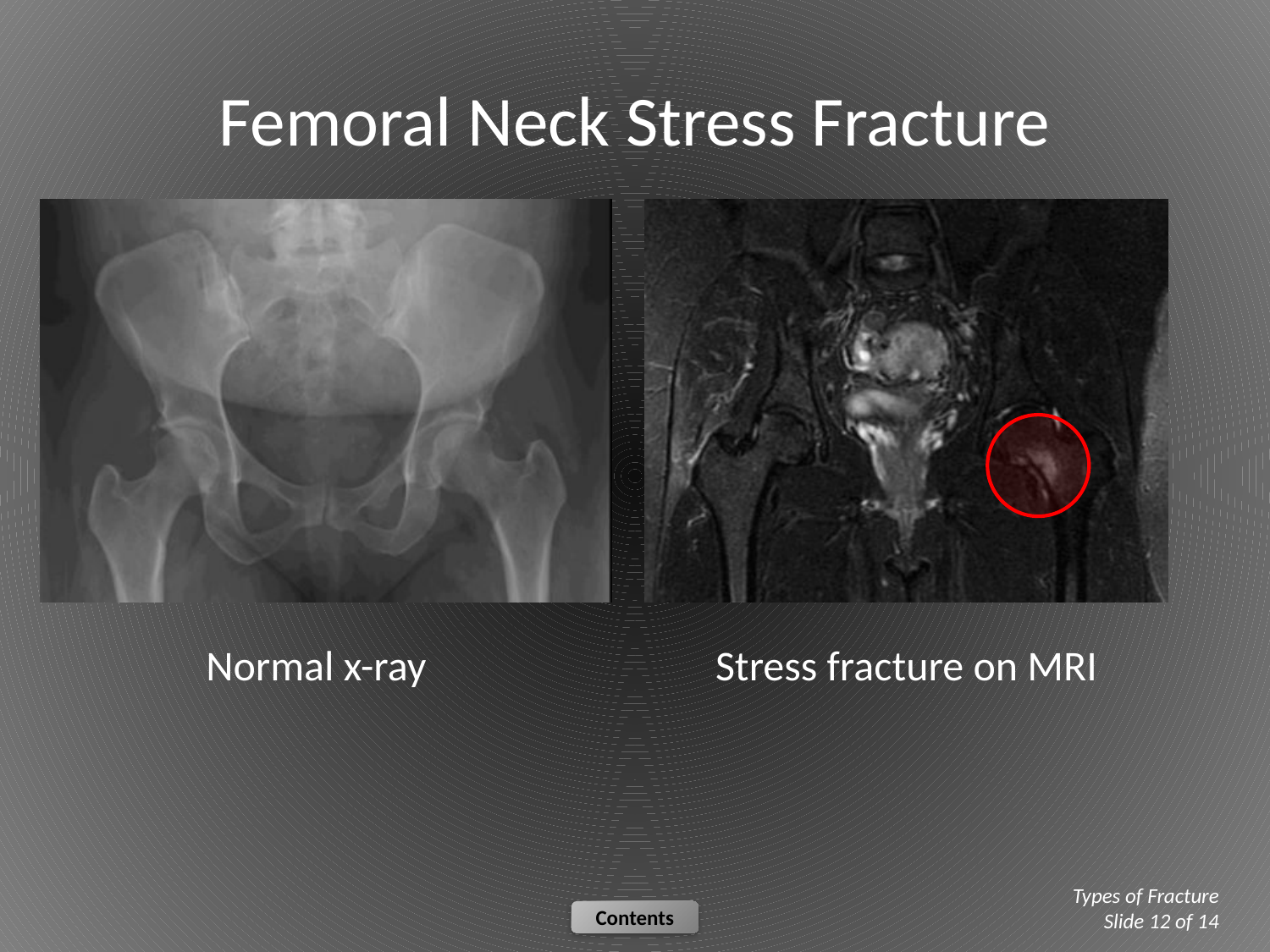

# Femoral Neck Stress Fracture
Stress fracture on MRI
Normal x-ray
Types of Fracture
Slide 12 of 14
Contents

## Slide 32
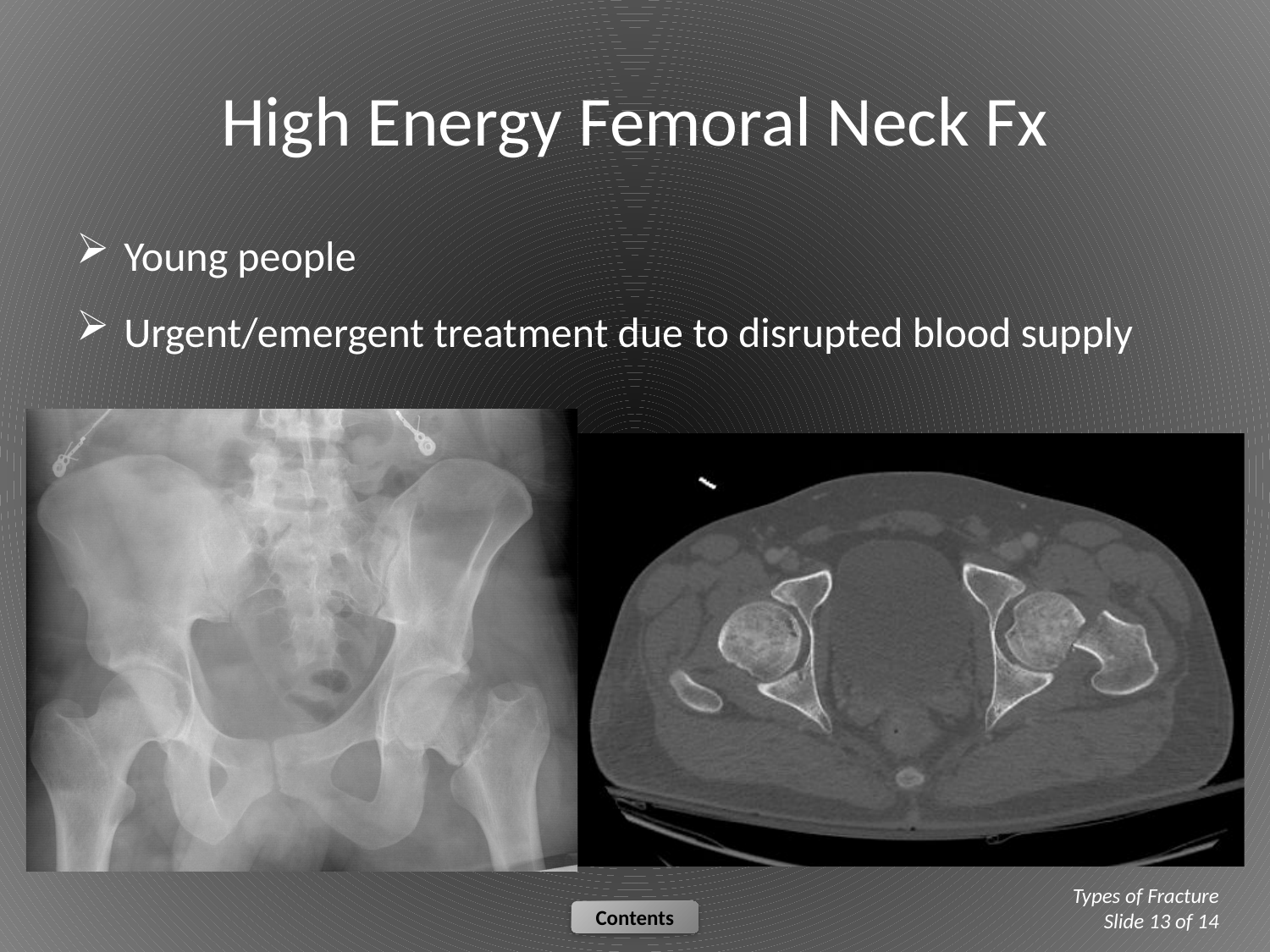

# High Energy Femoral Neck Fx
Young people
Urgent/emergent treatment due to disrupted blood supply
Types of Fracture
Slide 13 of 14
Contents

## Slide 33
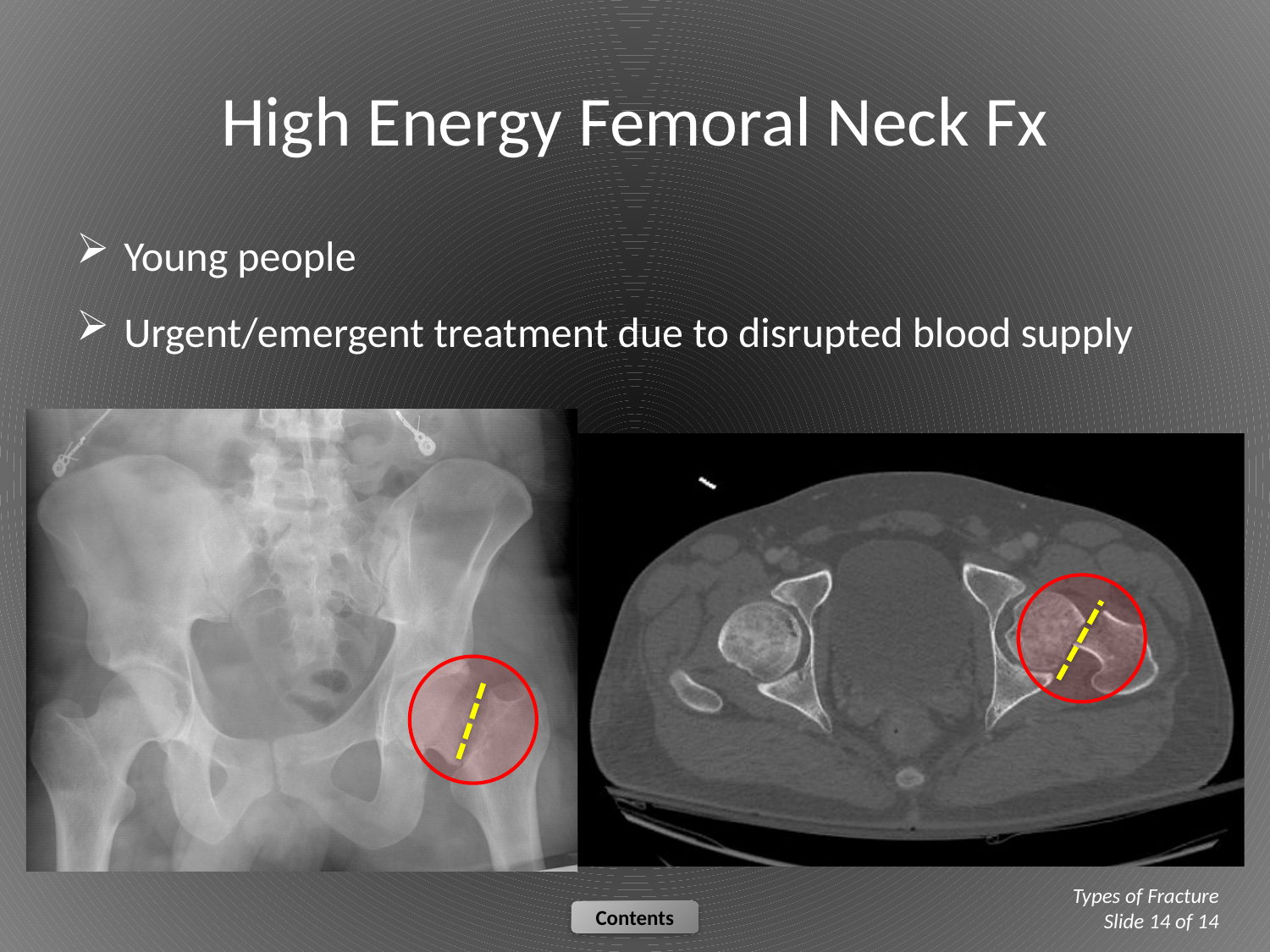

# High Energy Femoral Neck Fx
Young people
Urgent/emergent treatment due to disrupted blood supply
Types of Fracture
Slide 14 of 14
Contents

## Slide 34
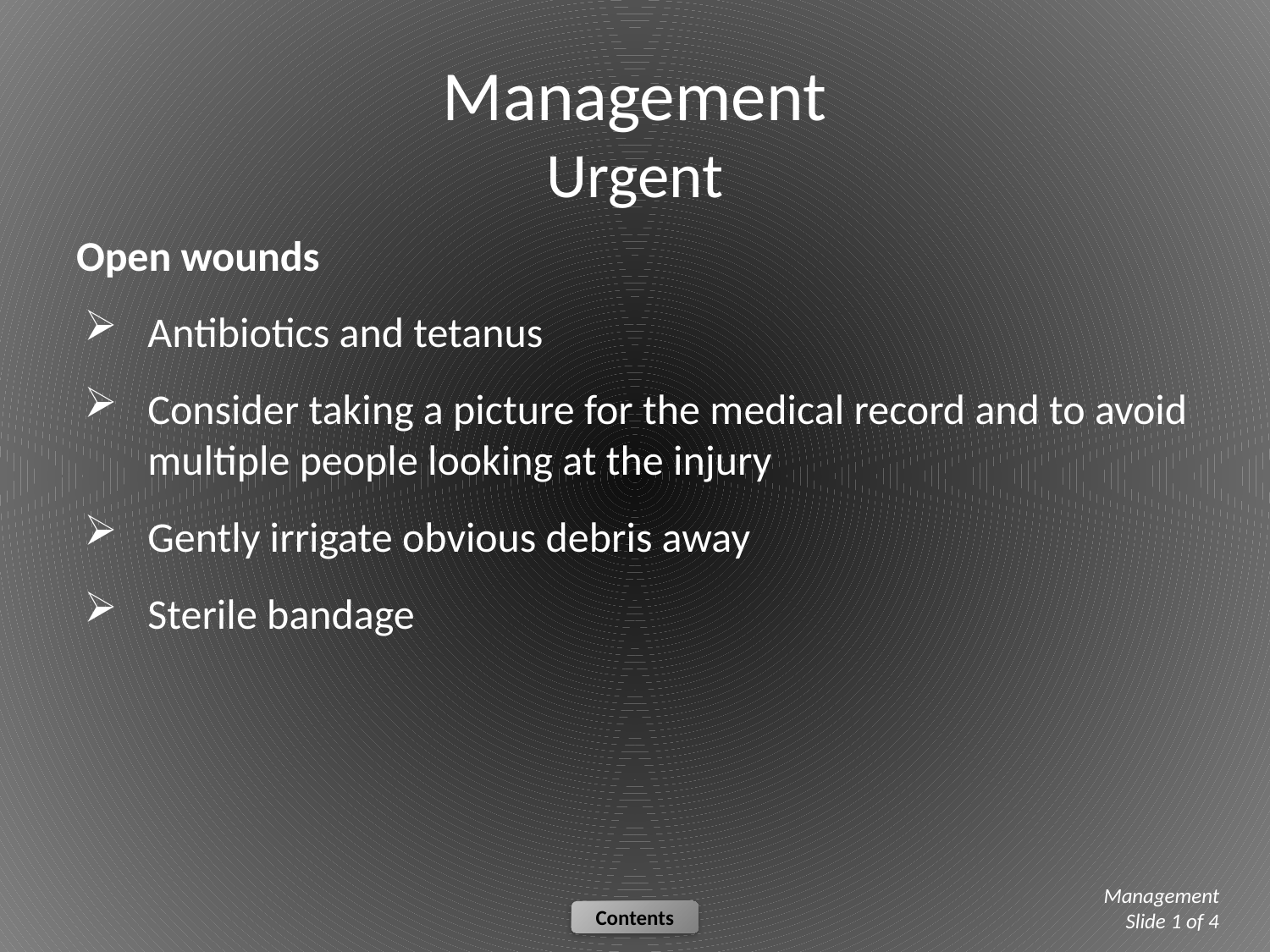

# Management Urgent
Open wounds
Antibiotics and tetanus
Consider taking a picture for the medical record and to avoid multiple people looking at the injury
Gently irrigate obvious debris away
Sterile bandage
Management
Slide 1 of 4
Contents

## Slide 35
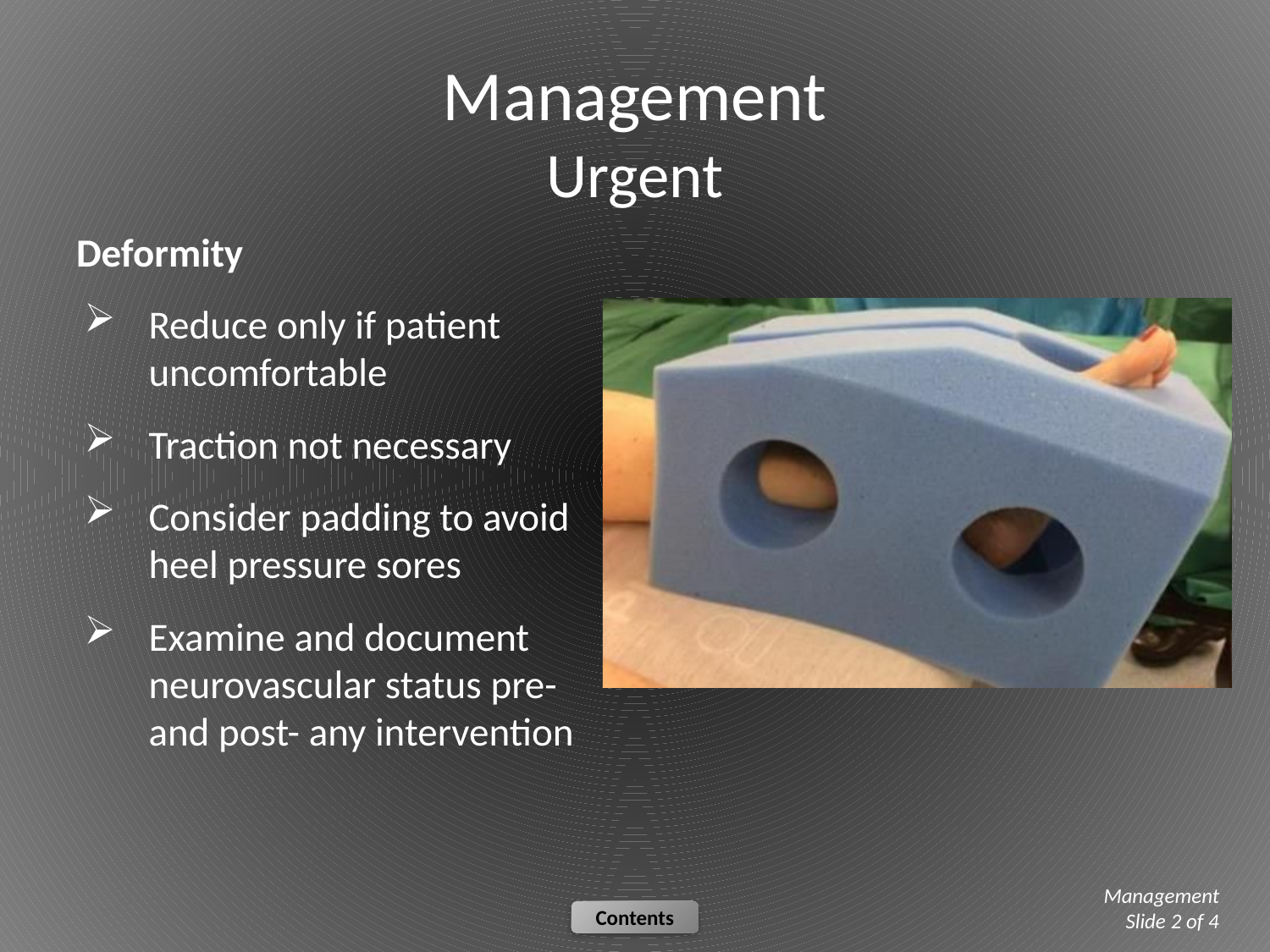

# Management Urgent
Deformity
Reduce only if patient uncomfortable
Traction not necessary
Consider padding to avoid heel pressure sores
Examine and document neurovascular status pre- and post- any intervention
Management
Slide 2 of 4
Contents

## Slide 36
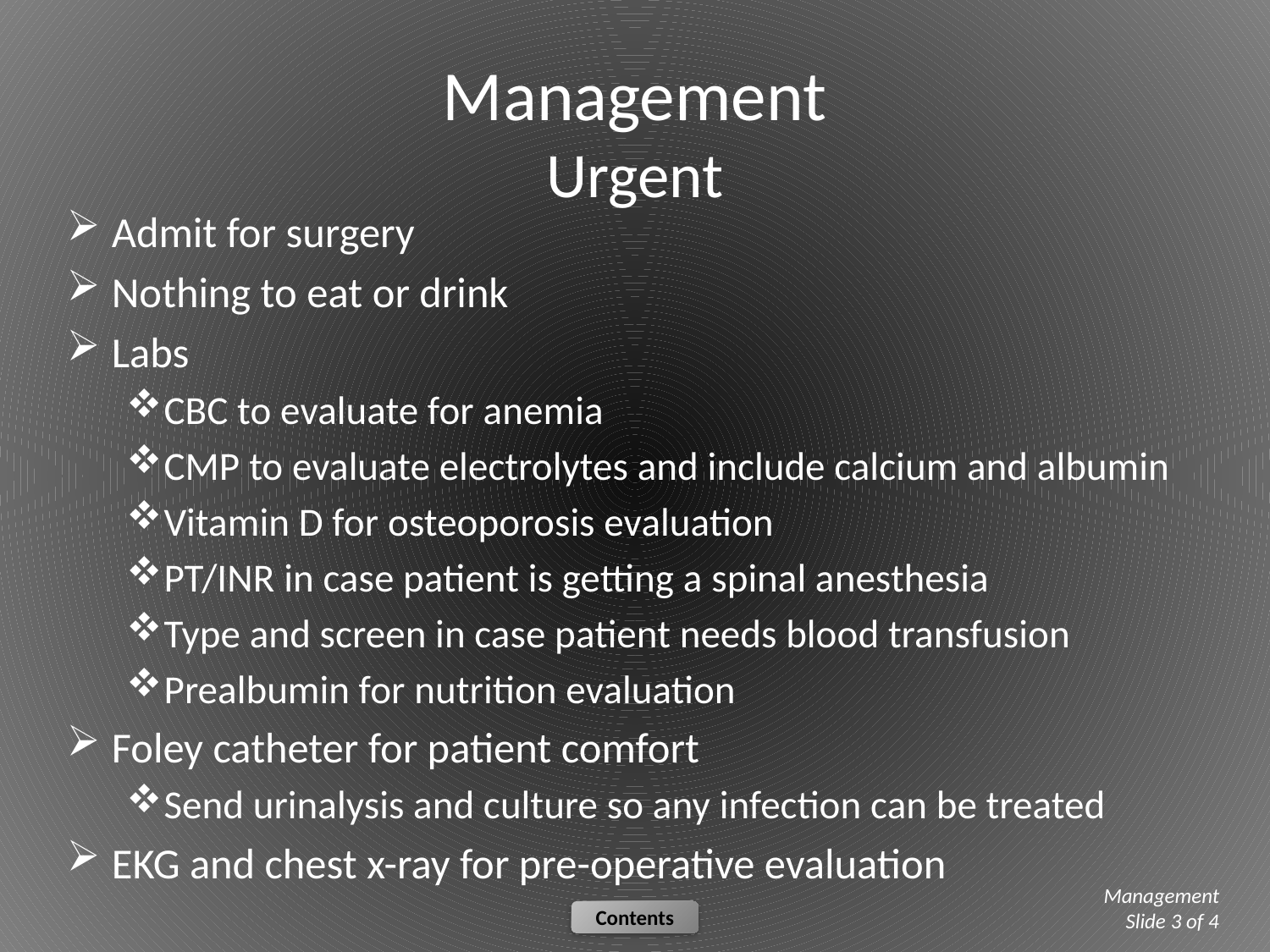

# Management Urgent
Admit for surgery
Nothing to eat or drink
Labs
CBC to evaluate for anemia
CMP to evaluate electrolytes and include calcium and albumin
Vitamin D for osteoporosis evaluation
PT/INR in case patient is getting a spinal anesthesia
Type and screen in case patient needs blood transfusion
Prealbumin for nutrition evaluation
Foley catheter for patient comfort
Send urinalysis and culture so any infection can be treated
EKG and chest x-ray for pre-operative evaluation
Management
Slide 3 of 4
Contents

## Slide 37
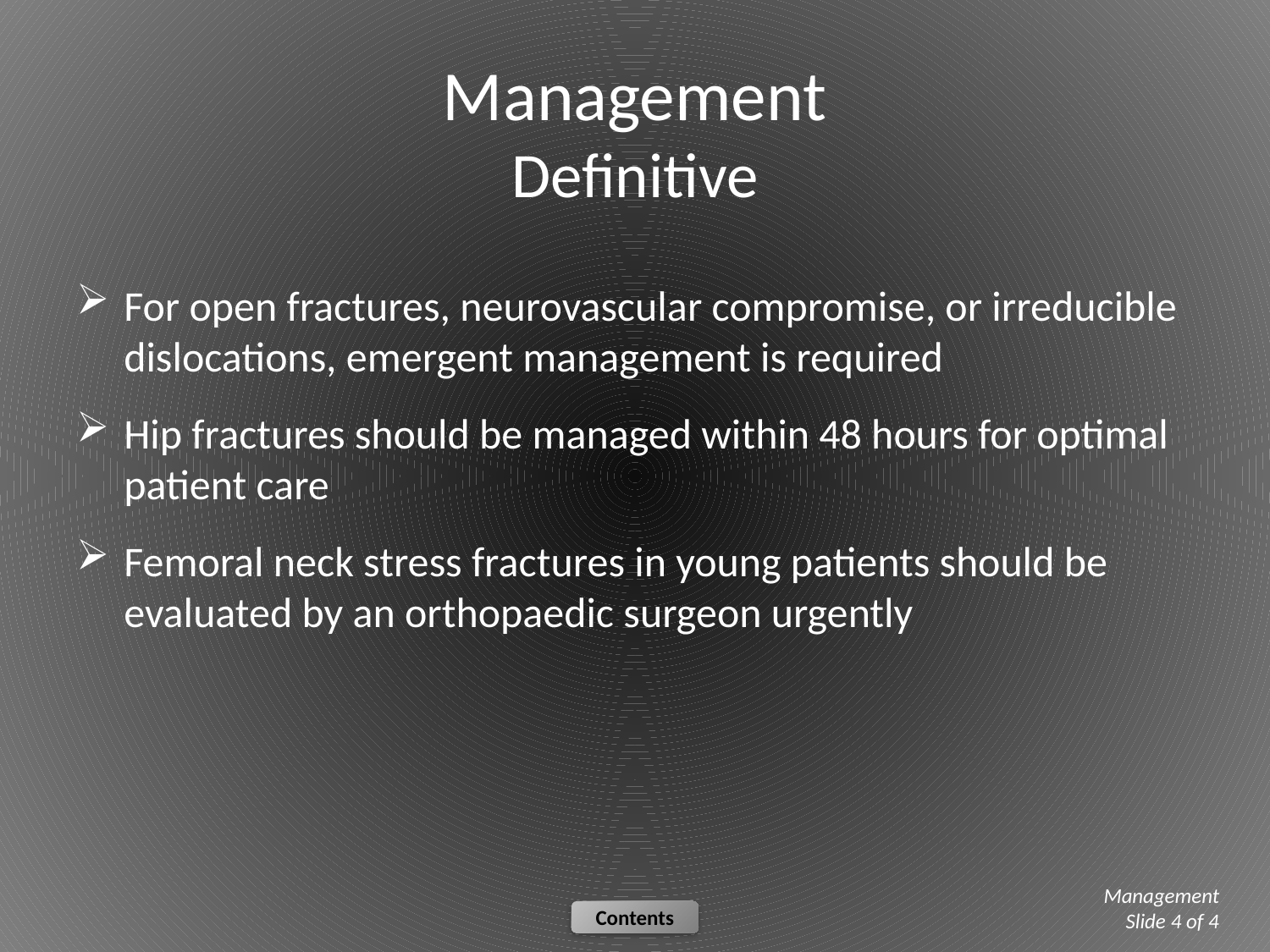

# Management Definitive
For open fractures, neurovascular compromise, or irreducible dislocations, emergent management is required
Hip fractures should be managed within 48 hours for optimal patient care
Femoral neck stress fractures in young patients should be evaluated by an orthopaedic surgeon urgently
Management
Slide 4 of 4
Contents

## Slide 38
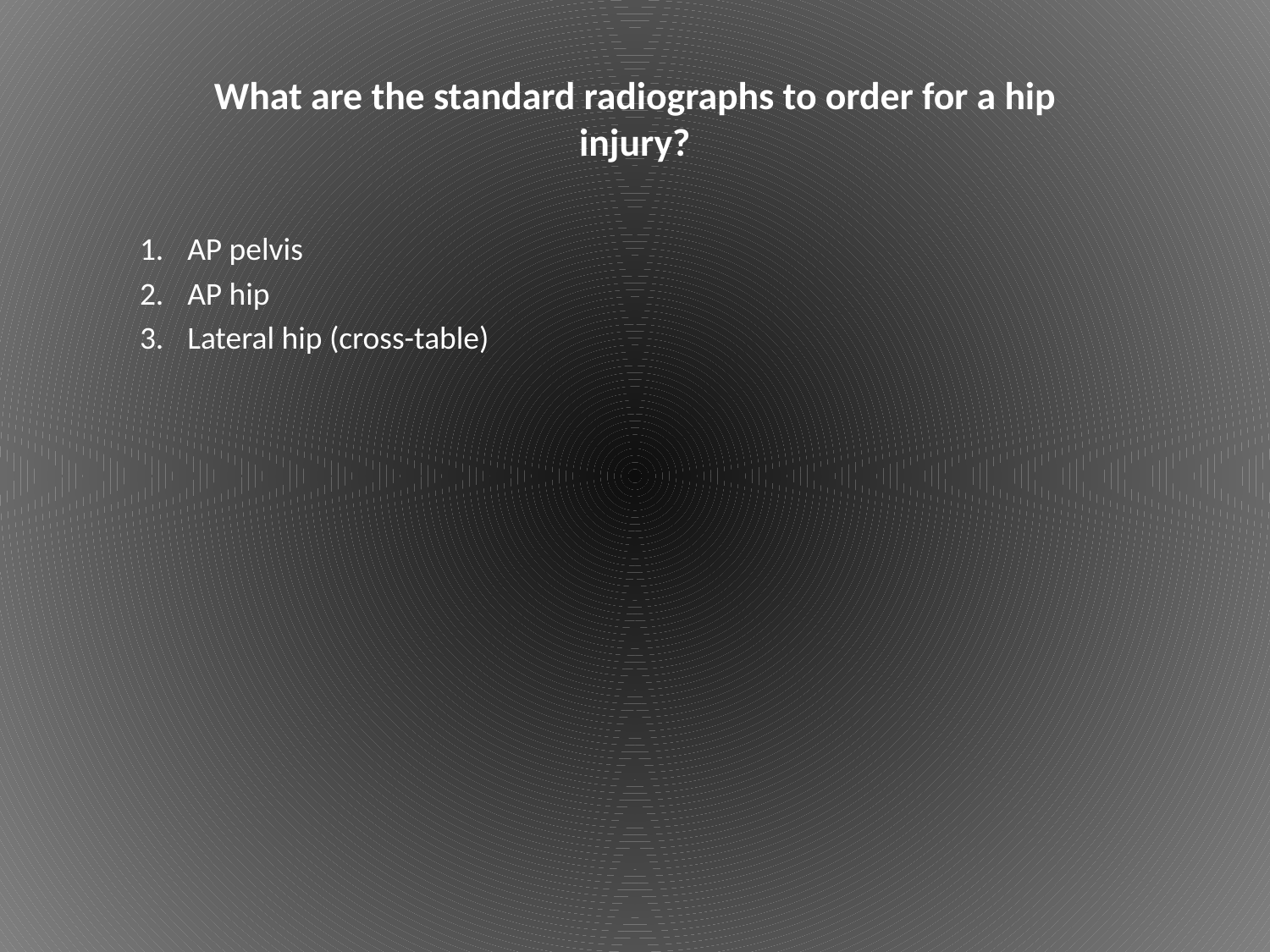

# What are the standard radiographs to order for a hip injury?
AP pelvis
AP hip
Lateral hip (cross-table)

## Slide 39
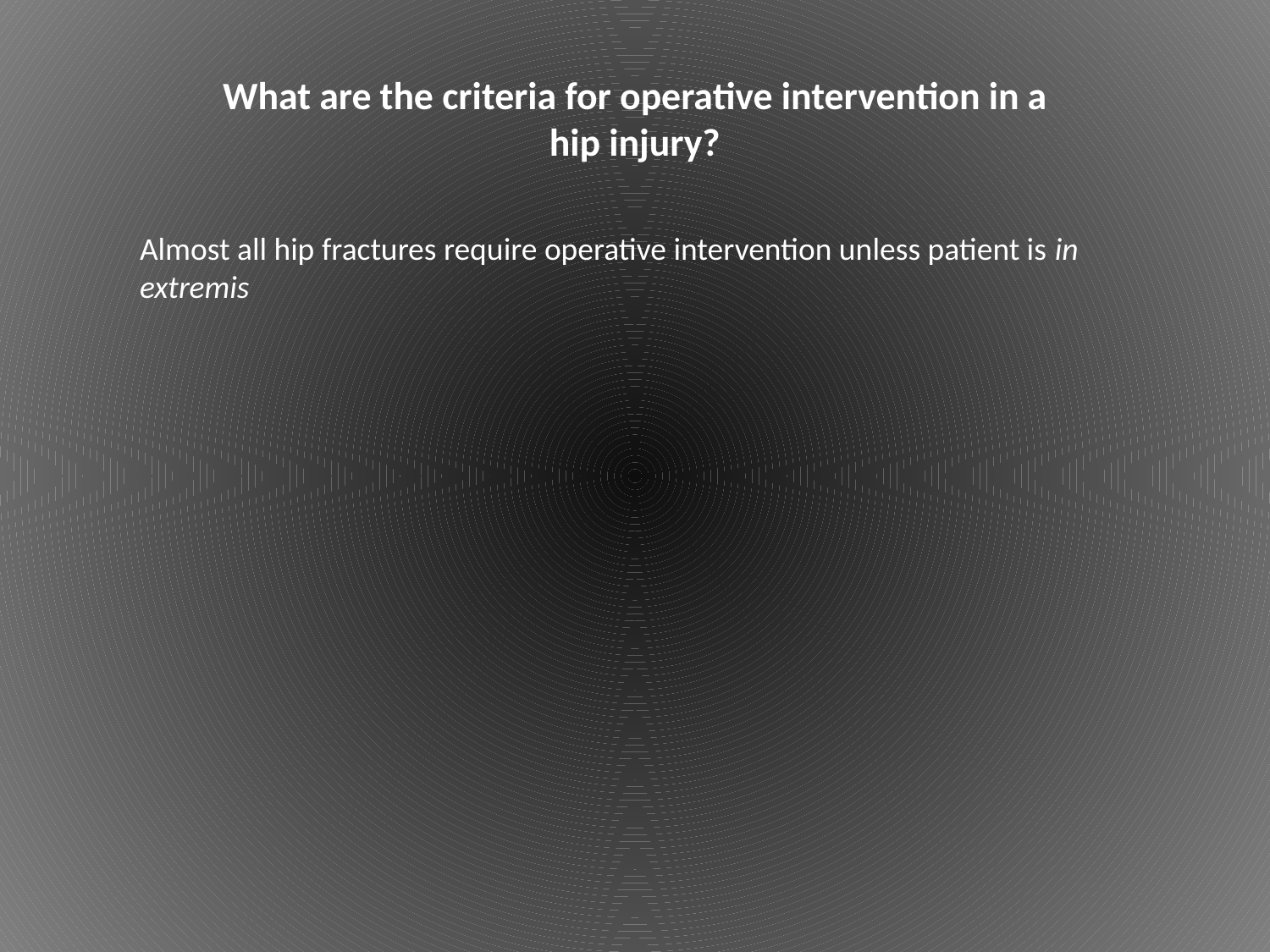

# What are the criteria for operative intervention in a hip injury?
Almost all hip fractures require operative intervention unless patient is in extremis

## Slide 40
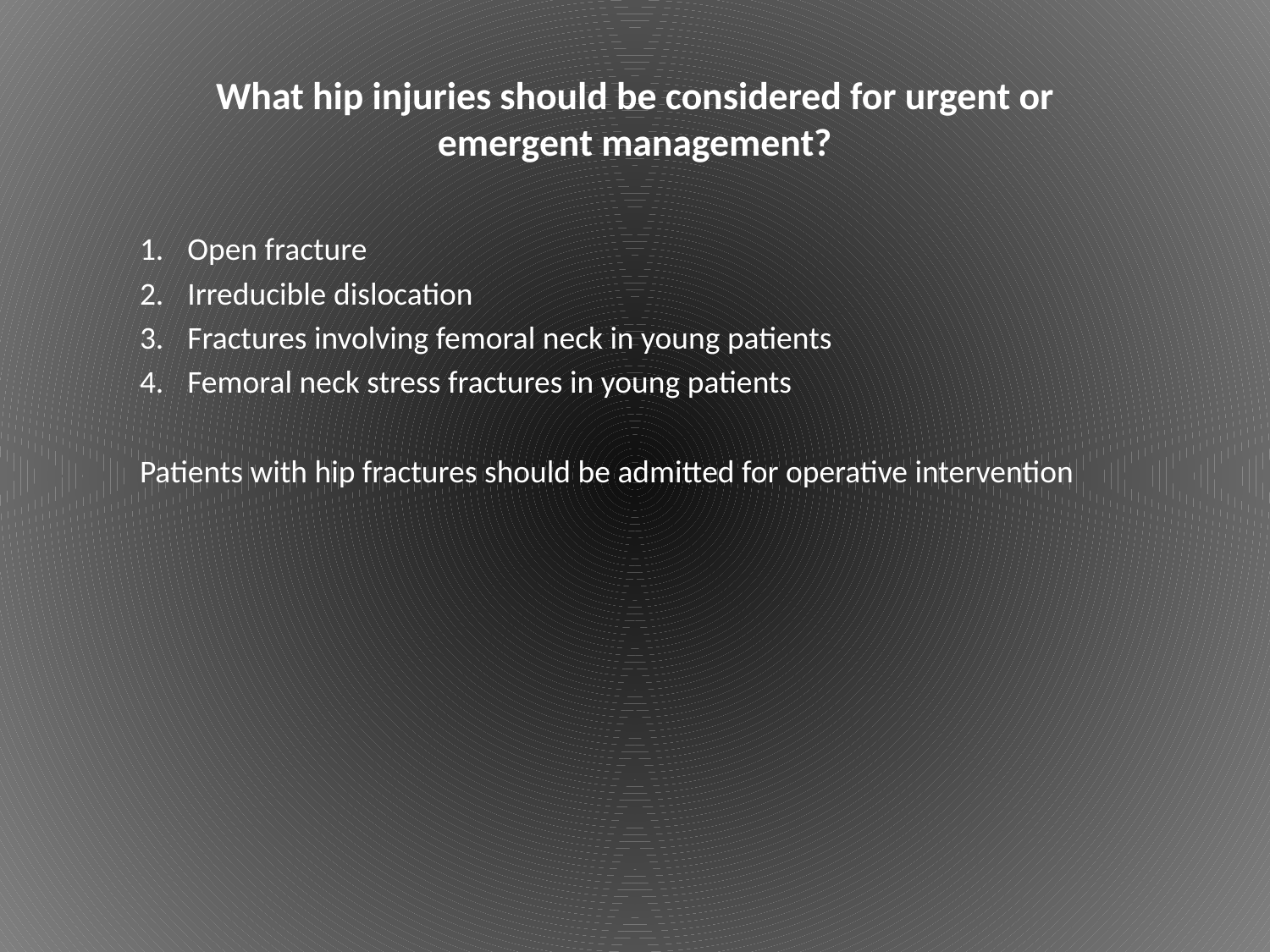

# What hip injuries should be considered for urgent or emergent management?
Open fracture
Irreducible dislocation
Fractures involving femoral neck in young patients
Femoral neck stress fractures in young patients
Patients with hip fractures should be admitted for operative intervention

## Slide 41
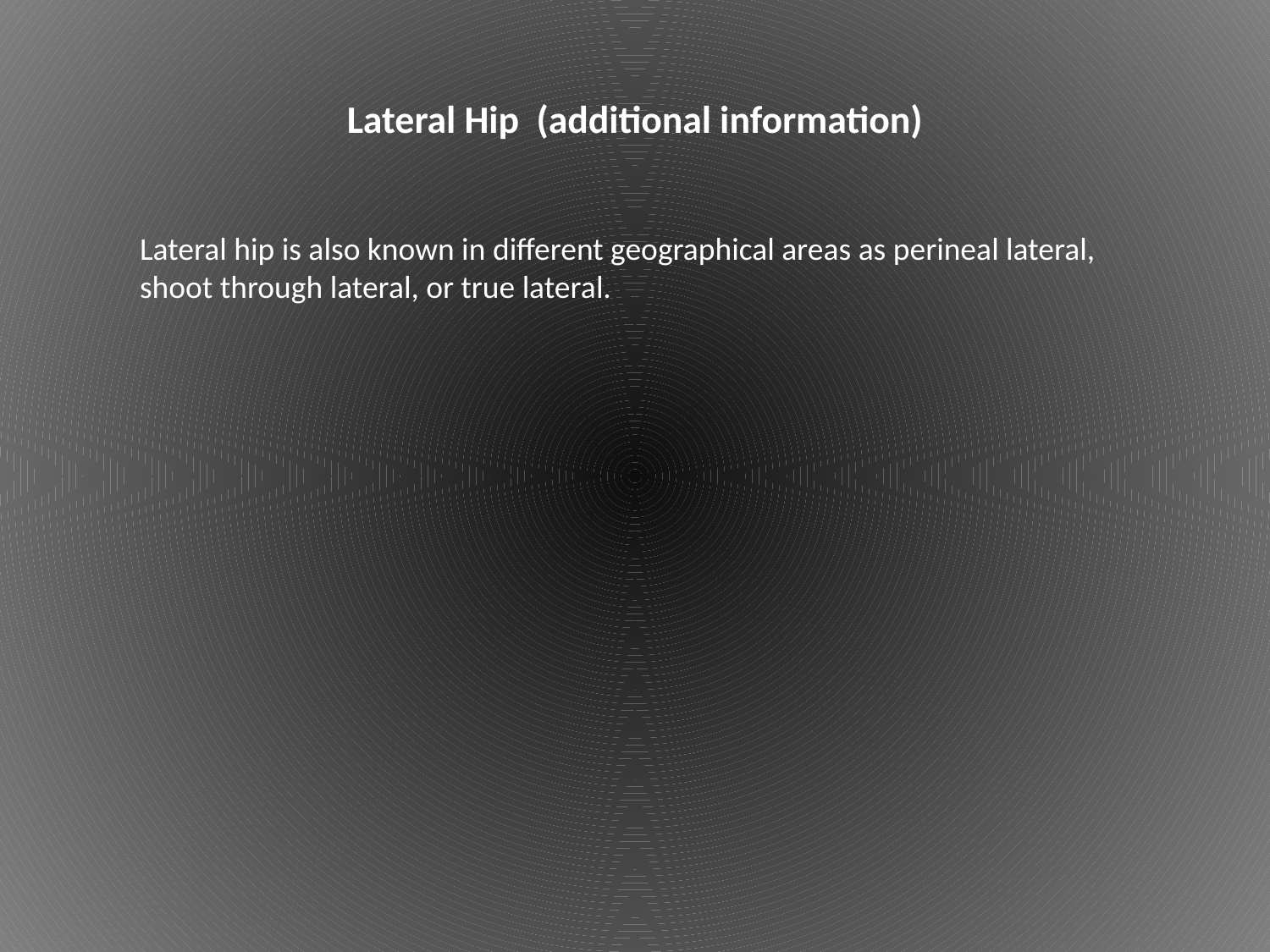

# Lateral Hip (additional information)
Lateral hip is also known in different geographical areas as perineal lateral, shoot through lateral, or true lateral.
