# Supplementary material for: Evaluation of Common Musculoskeletal Injuries in the Urgent Setting
Source: MedEdPORTAL. 2016 Dec 7;12:10514. doi: 10.15766/mep_2374-8265.10514 (PMC6440529; doi:10.15766/mep_2374-8265.10514)
Supplement: Supplementary file 1 — A. Evaluation of Common Musculoskeletal Injuries in the Urgent Setting.pptx B. Evaluation of Ankle Injuries in the Urgent Setting.pptx C. Evaluation of Hip Injuries in the Urgent Setting.pptx D. Evaluation of Shoulder Injuries in the Urgent Setting.pptx E. Evaluation of Wrist Injuries in the Urgent Setting.pptx [file mep-12-10514-s001.zip › D. Evaluation of Shoulder Injuries in the Urgent Setting.pptx]

## Slide 1
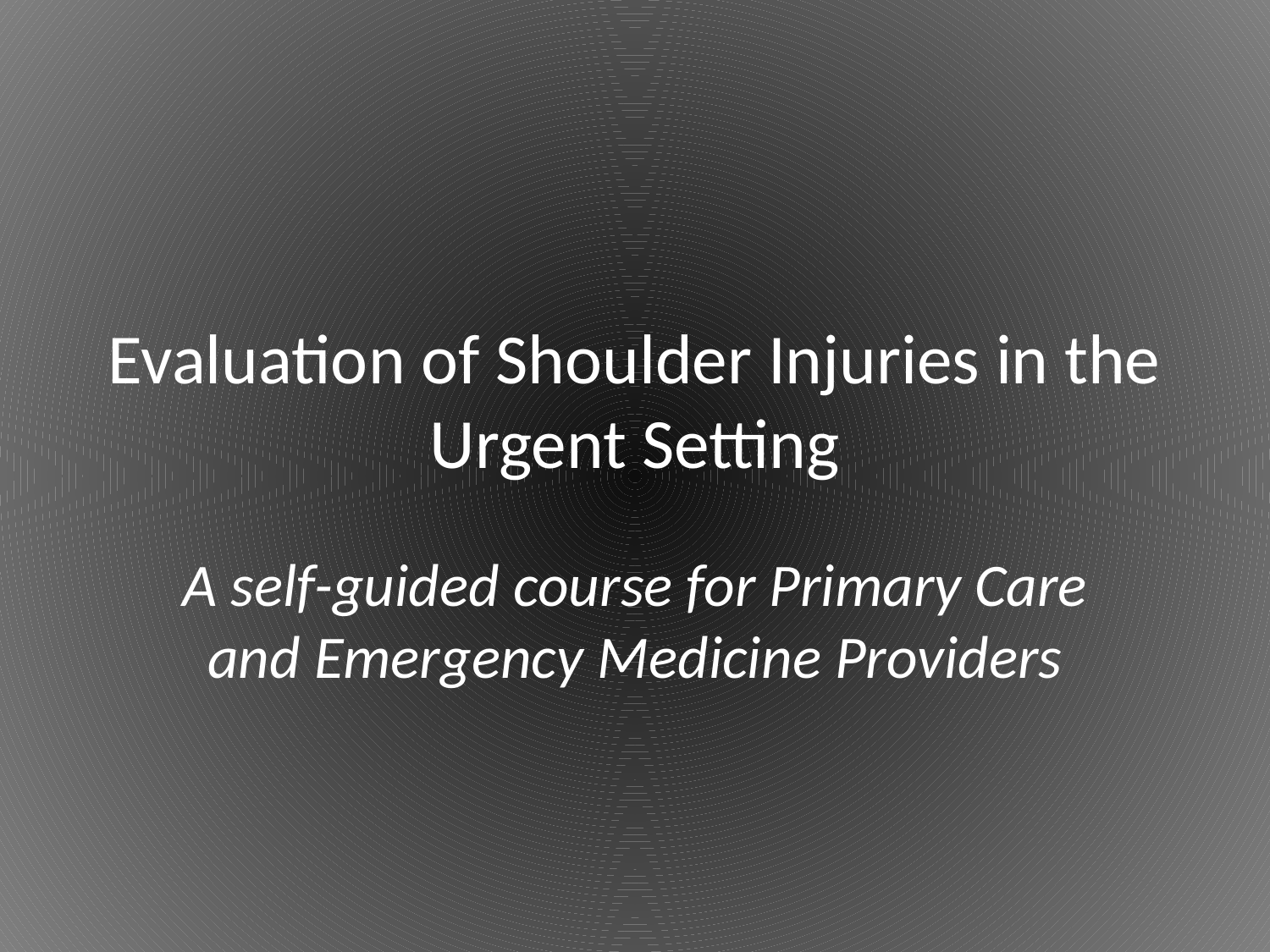

# Evaluation of Shoulder Injuries in the Urgent Setting
A self-guided course for Primary Care and Emergency Medicine Providers

## Slide 2
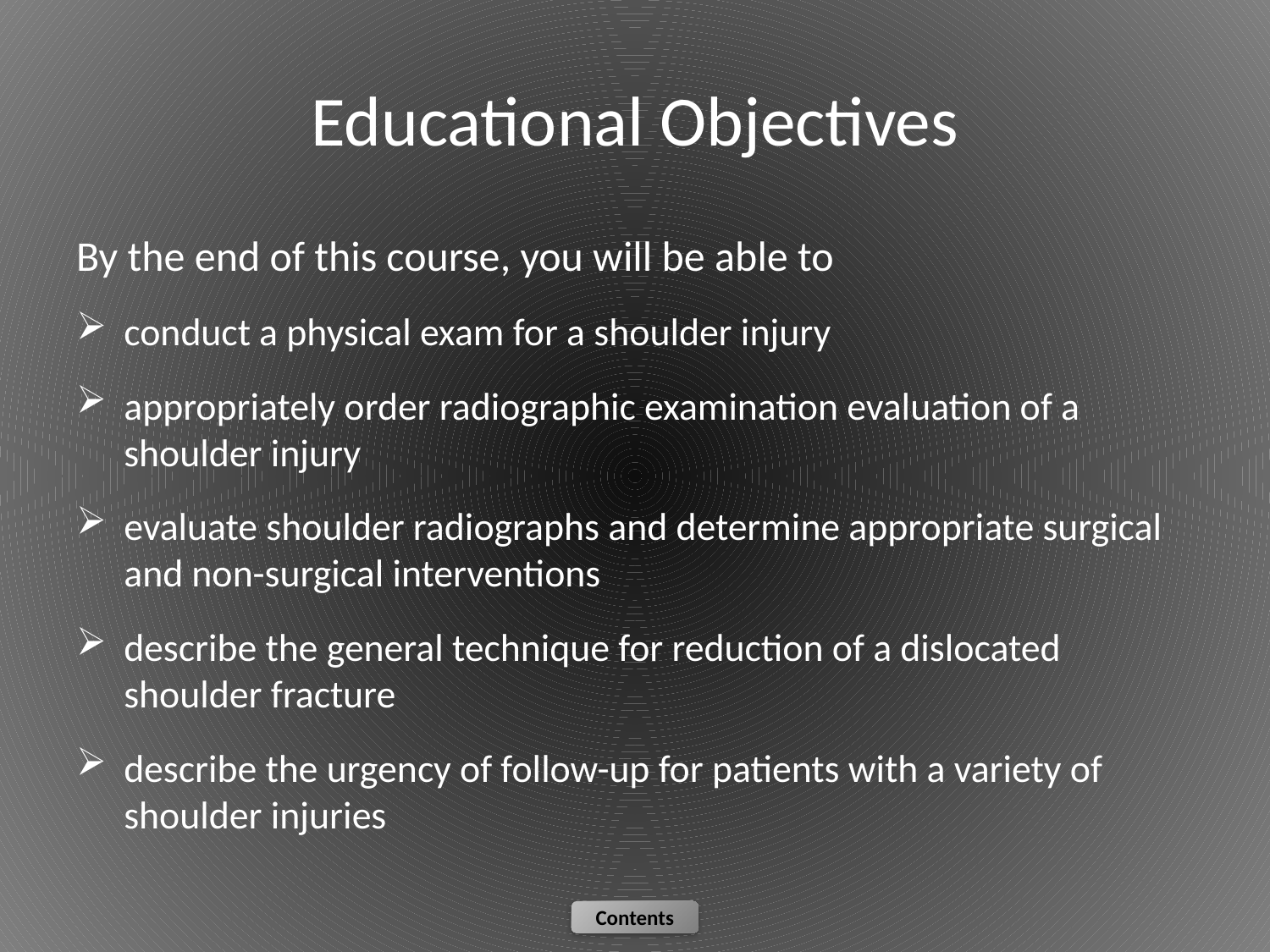

# Educational Objectives
By the end of this course, you will be able to
conduct a physical exam for a shoulder injury
appropriately order radiographic examination evaluation of a shoulder injury
evaluate shoulder radiographs and determine appropriate surgical and non-surgical interventions
describe the general technique for reduction of a dislocated shoulder fracture
describe the urgency of follow-up for patients with a variety of shoulder injuries
Contents

## Slide 3
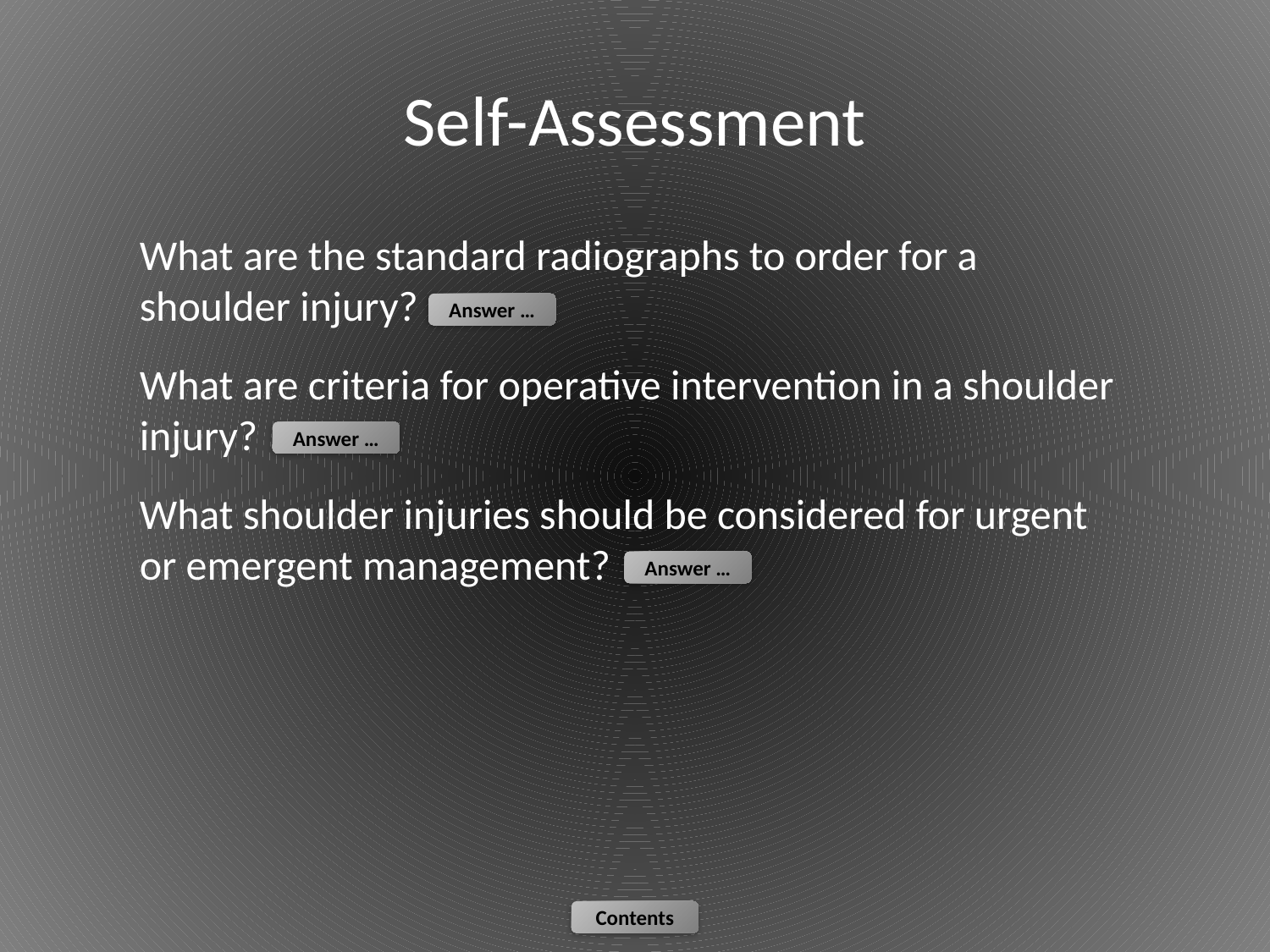

# Self-Assessment
What are the standard radiographs to order for a shoulder injury?
What are criteria for operative intervention in a shoulder injury?
What shoulder injuries should be considered for urgent or emergent management?
Answer …
Answer …
Answer …
Contents

## Slide 4
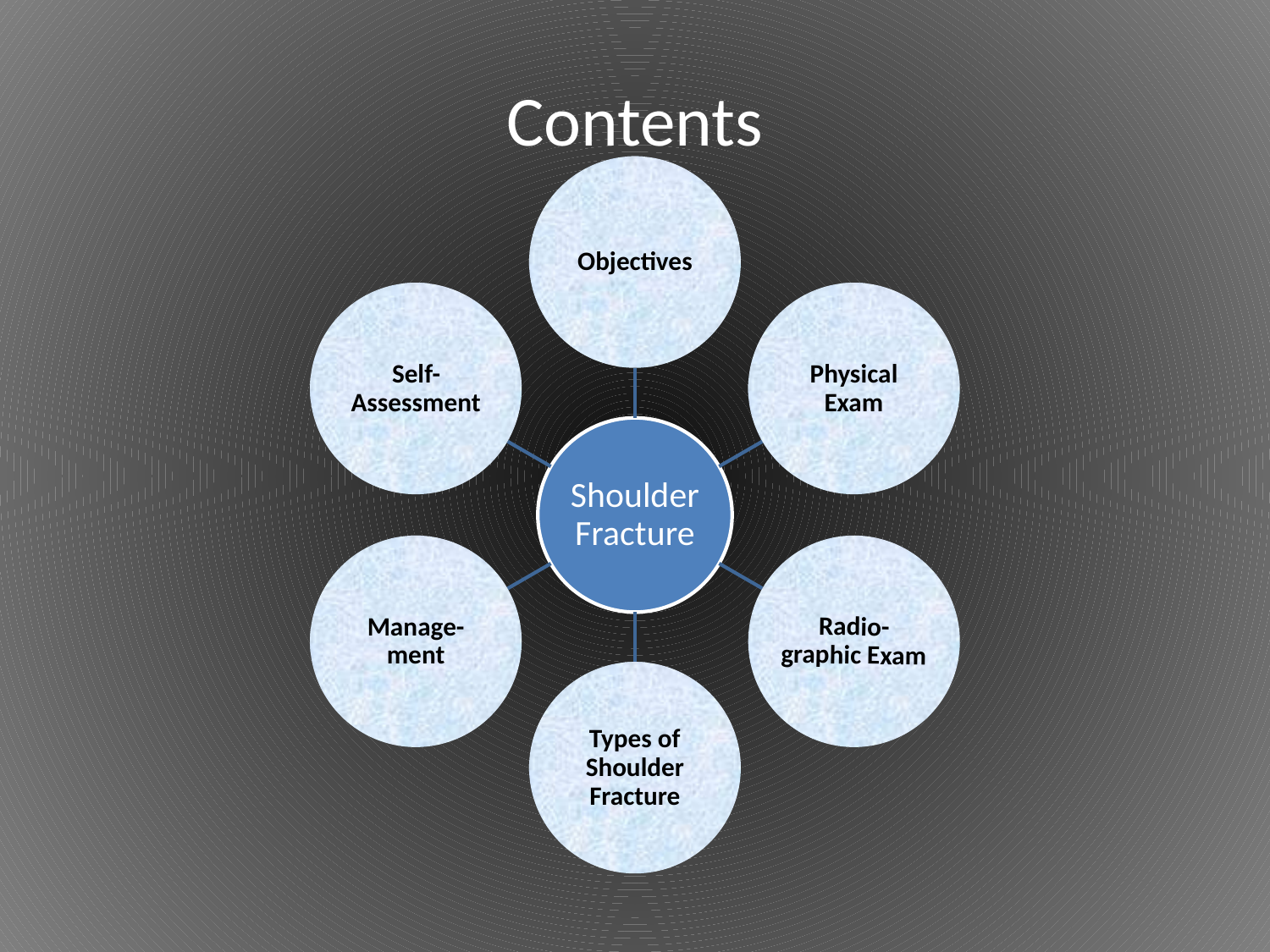

# Contents

## Slide 5
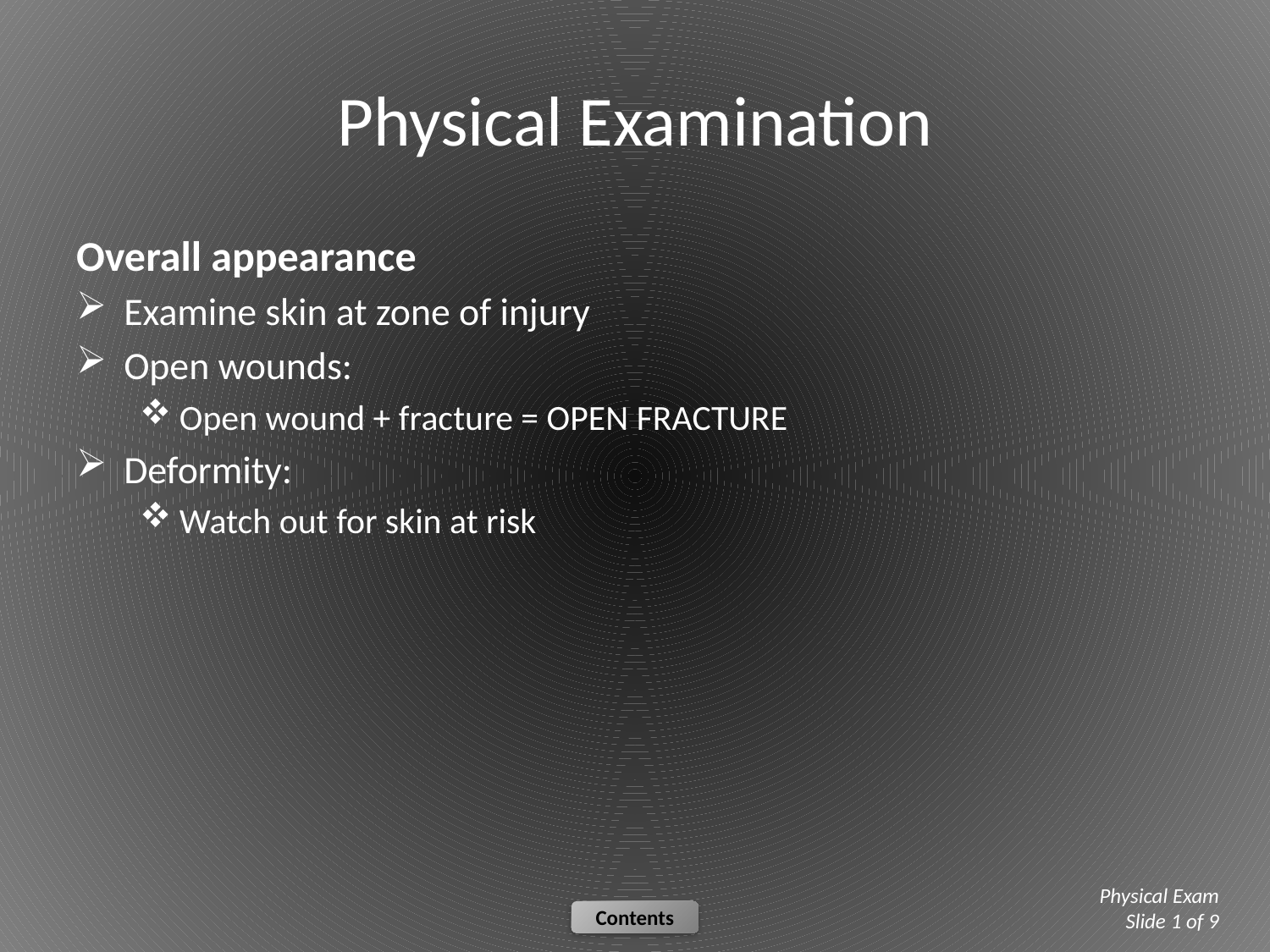

# Physical Examination
Overall appearance
Examine skin at zone of injury
Open wounds:
Open wound + fracture = OPEN FRACTURE
Deformity:
Watch out for skin at risk
Physical Exam
Slide 1 of 9
Contents

## Slide 6
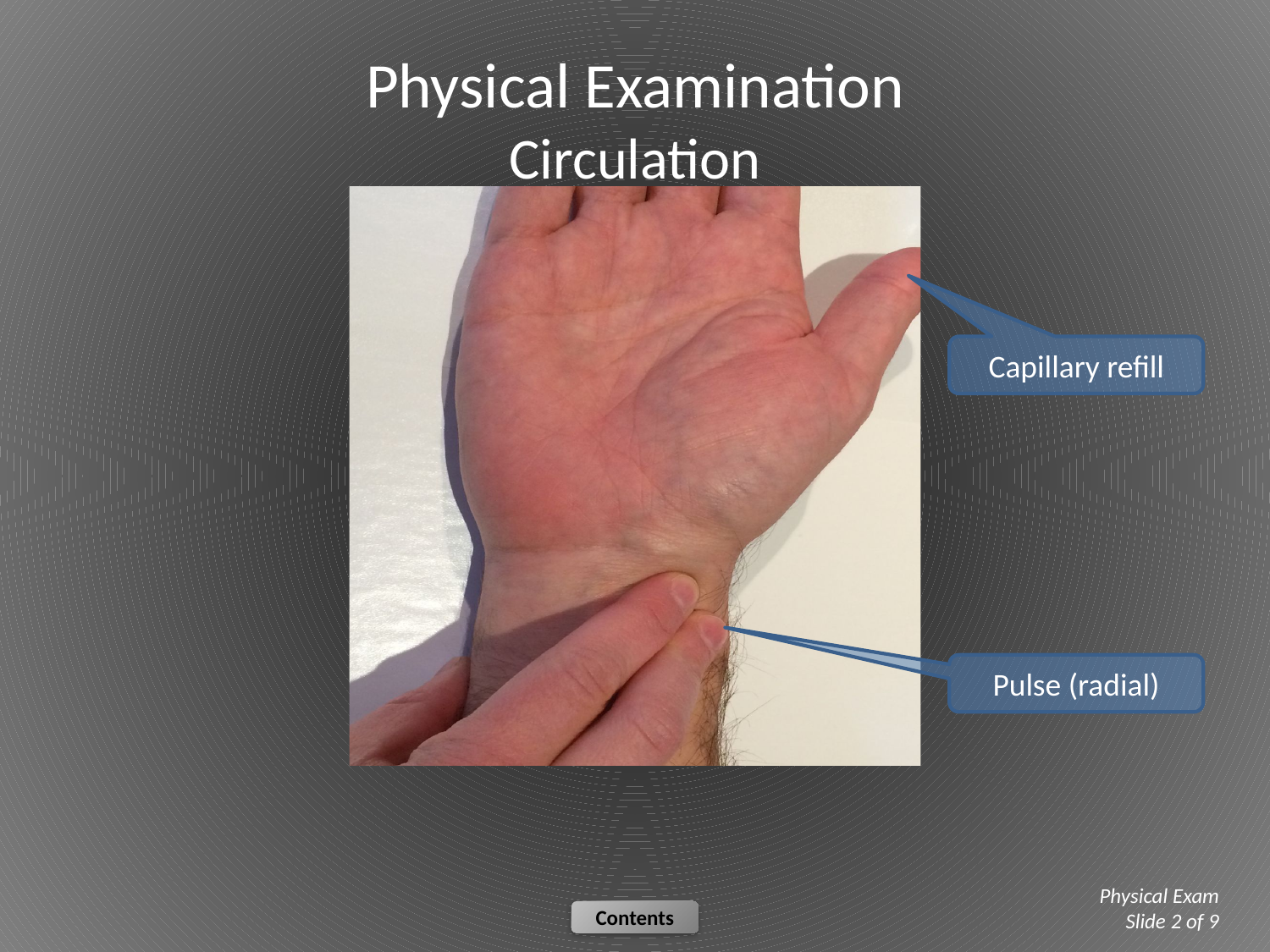

# Physical Examination Circulation
Capillary refill
Pulse (radial)
Physical Exam
Slide 2 of 9
Contents

## Slide 7
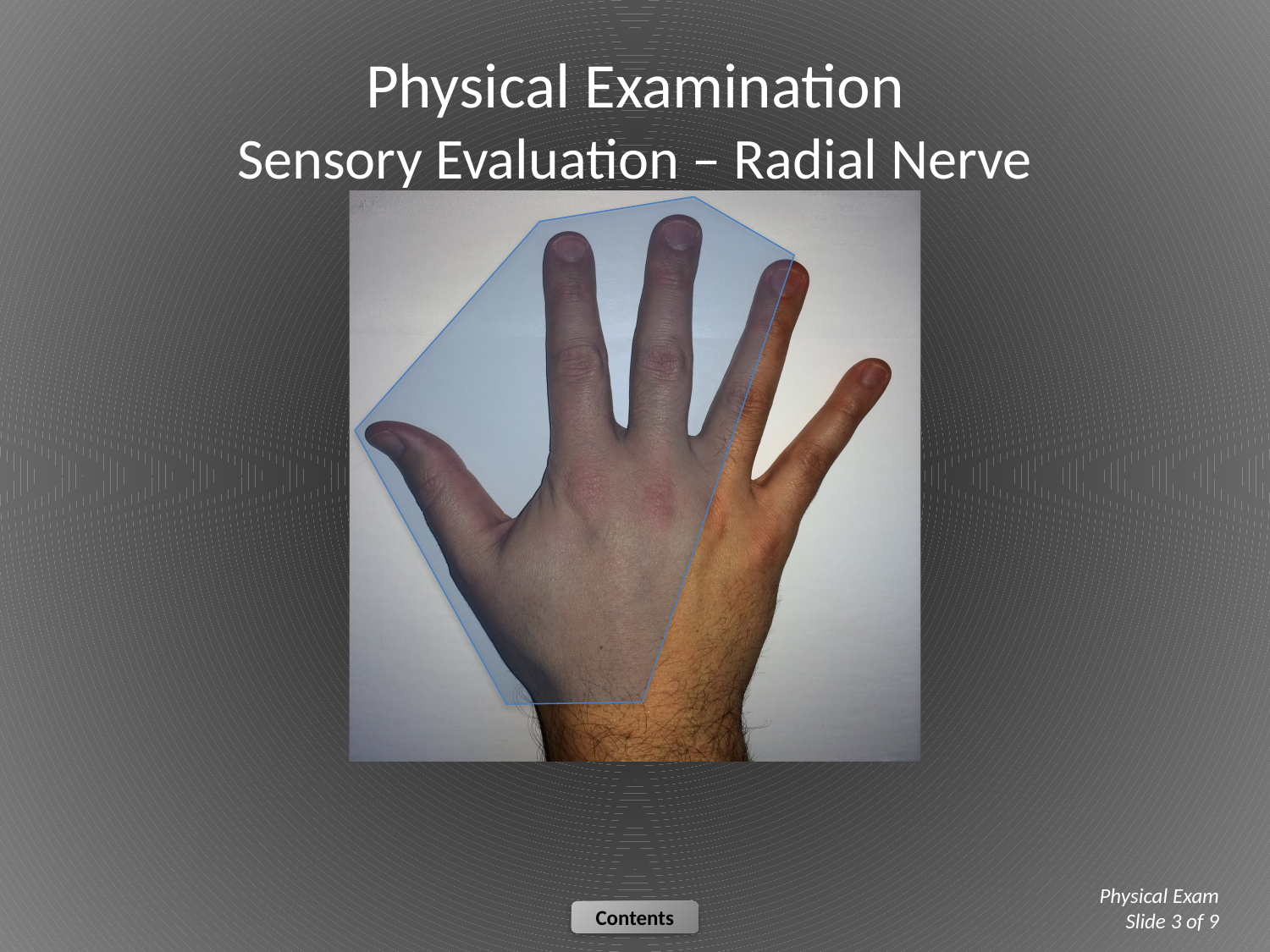

# Physical Examination Sensory Evaluation – Radial Nerve
Physical Exam
Slide 3 of 9
Contents

## Slide 8
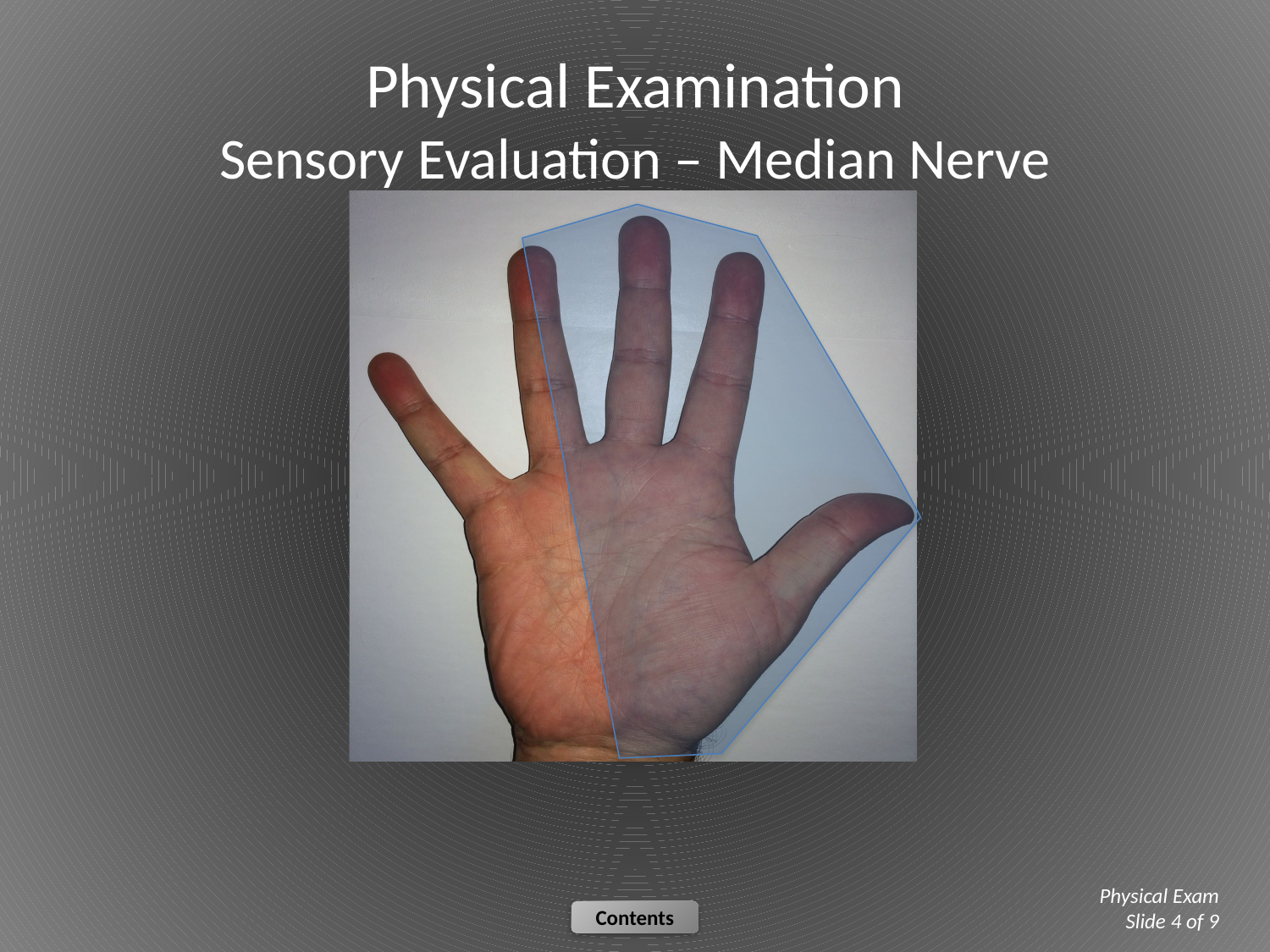

# Physical Examination Sensory Evaluation – Median Nerve
Physical Exam
Slide 4 of 9
Contents

## Slide 9
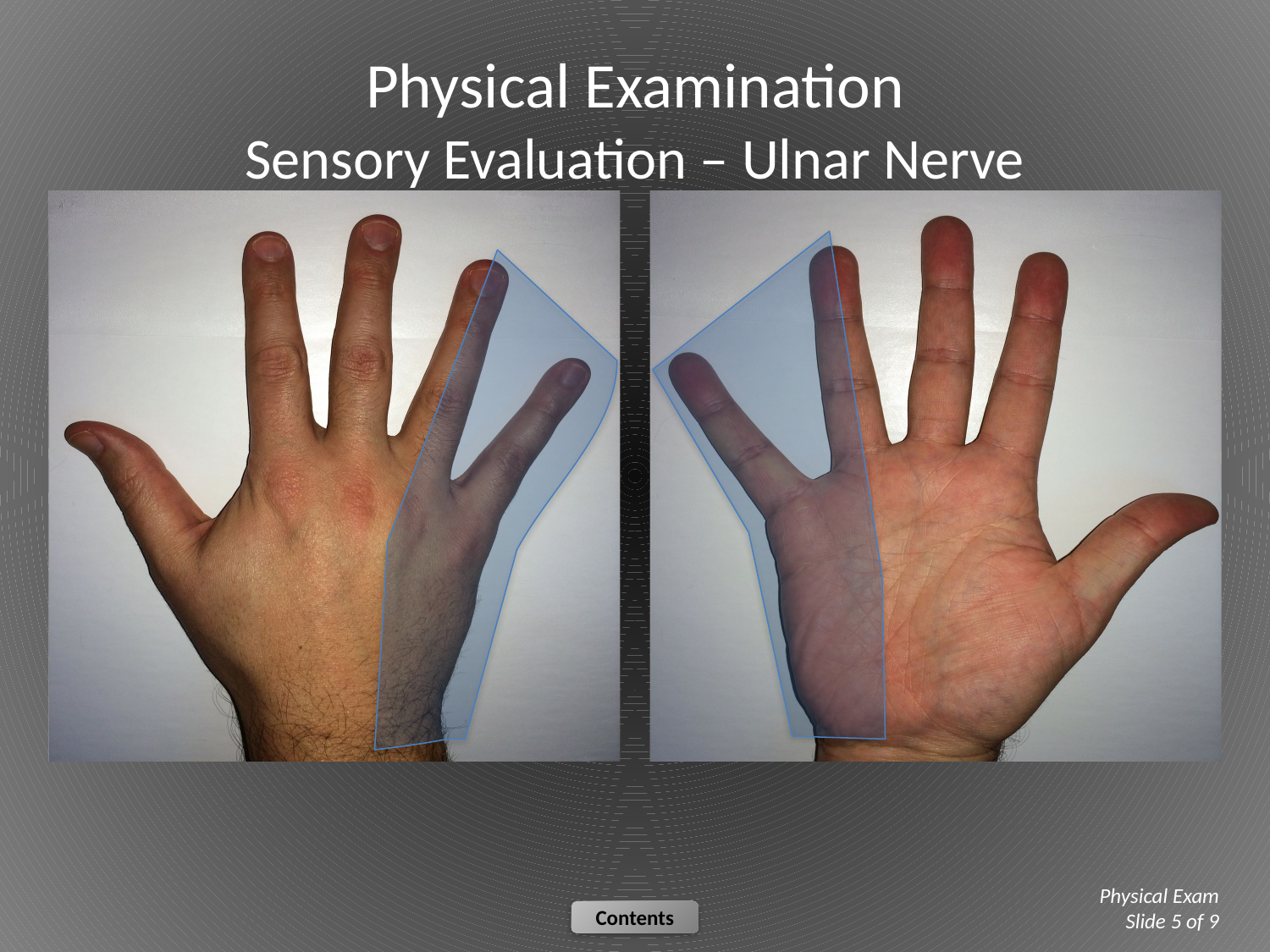

# Physical Examination Sensory Evaluation – Ulnar Nerve
Physical Exam
Slide 5 of 9
Contents

## Slide 10
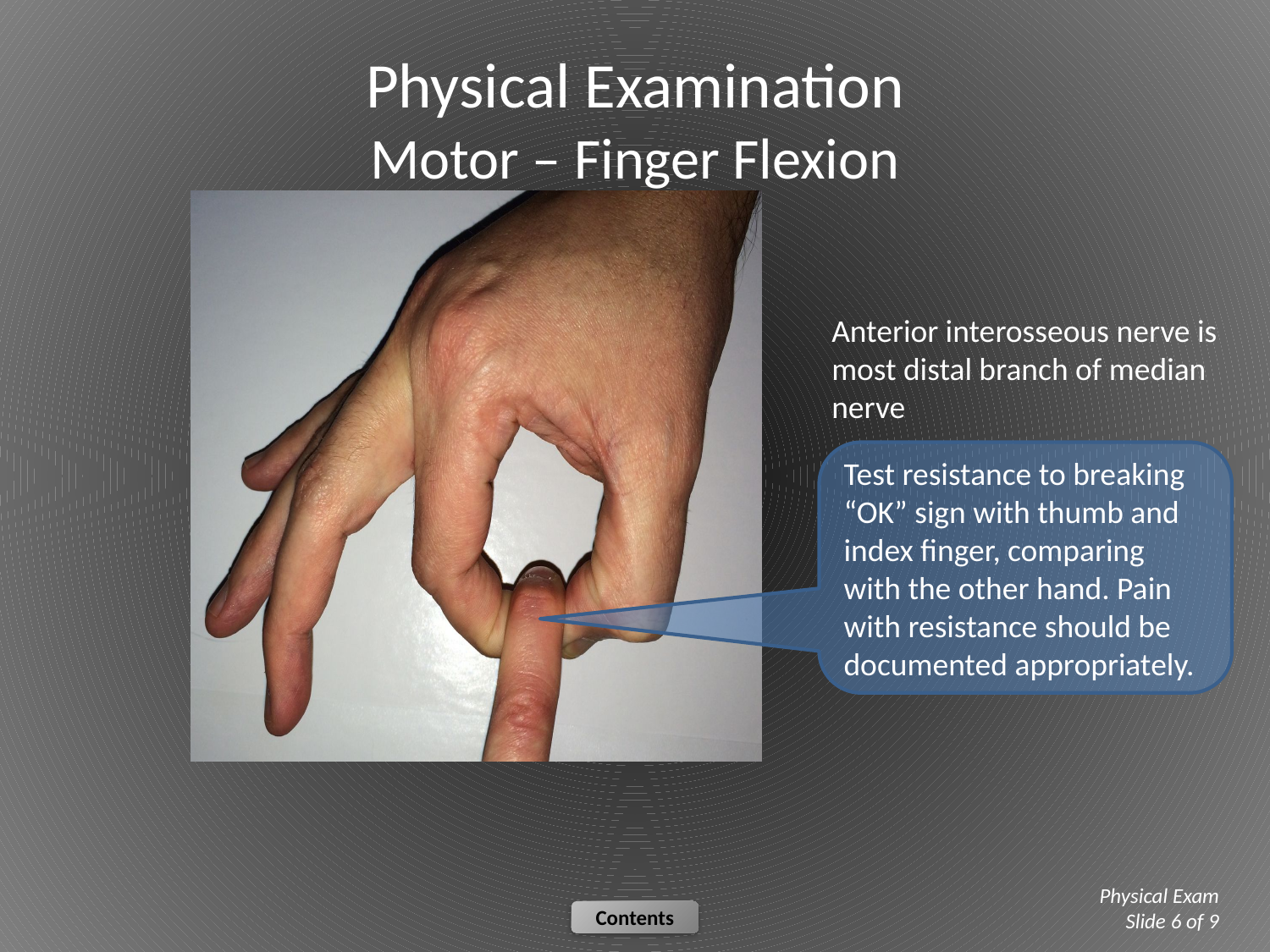

# Physical Examination Motor – Finger Flexion
Anterior interosseous nerve is most distal branch of median nerve
Test resistance to breaking “OK” sign with thumb and index finger, comparing with the other hand. Pain with resistance should be documented appropriately.
Physical Exam
Slide 6 of 9
Contents

## Slide 11
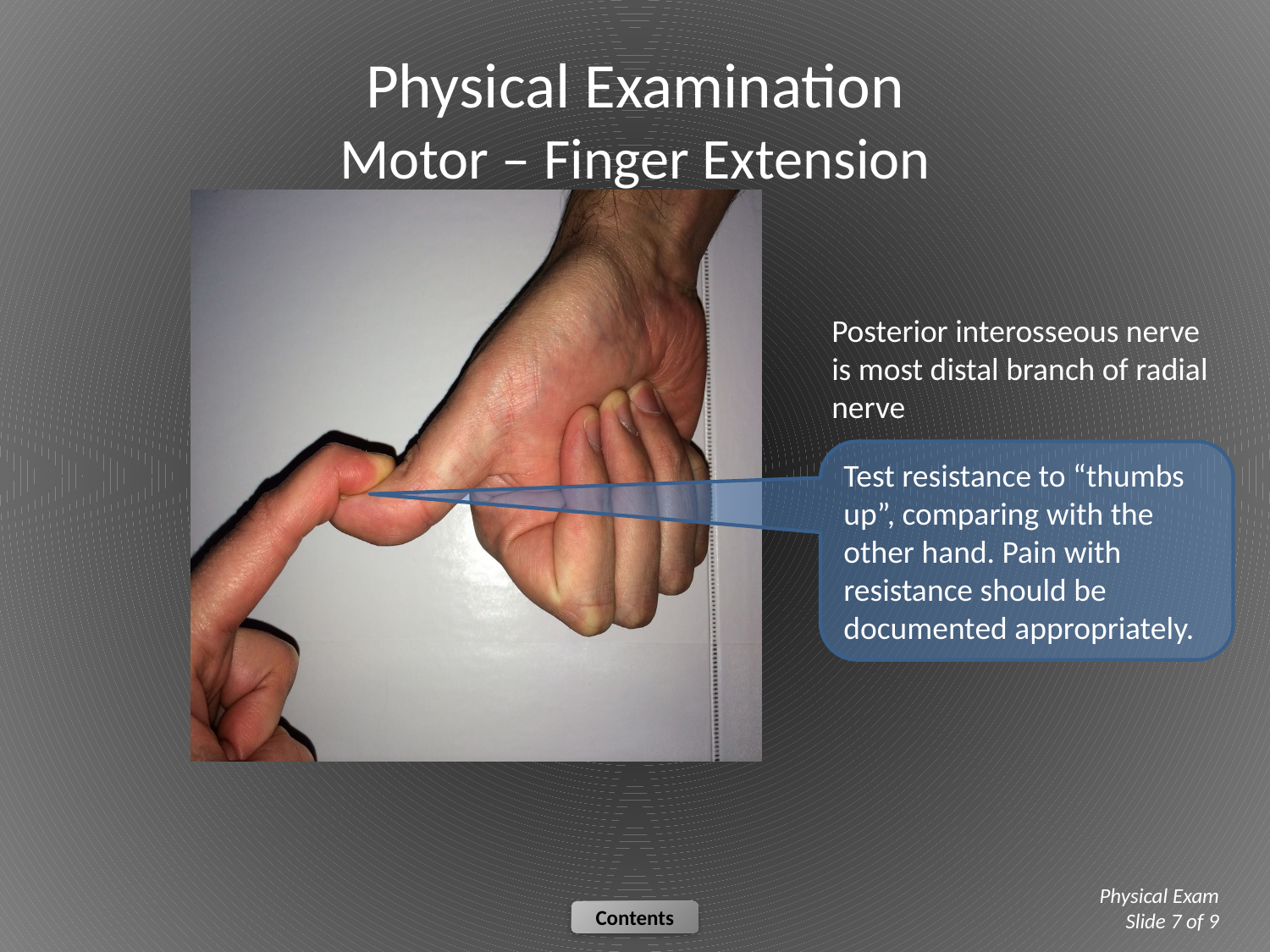

# Physical Examination Motor – Finger Extension
Posterior interosseous nerve is most distal branch of radial nerve
Test resistance to “thumbs up”, comparing with the other hand. Pain with resistance should be documented appropriately.
Physical Exam
Slide 7 of 9
Contents

## Slide 12
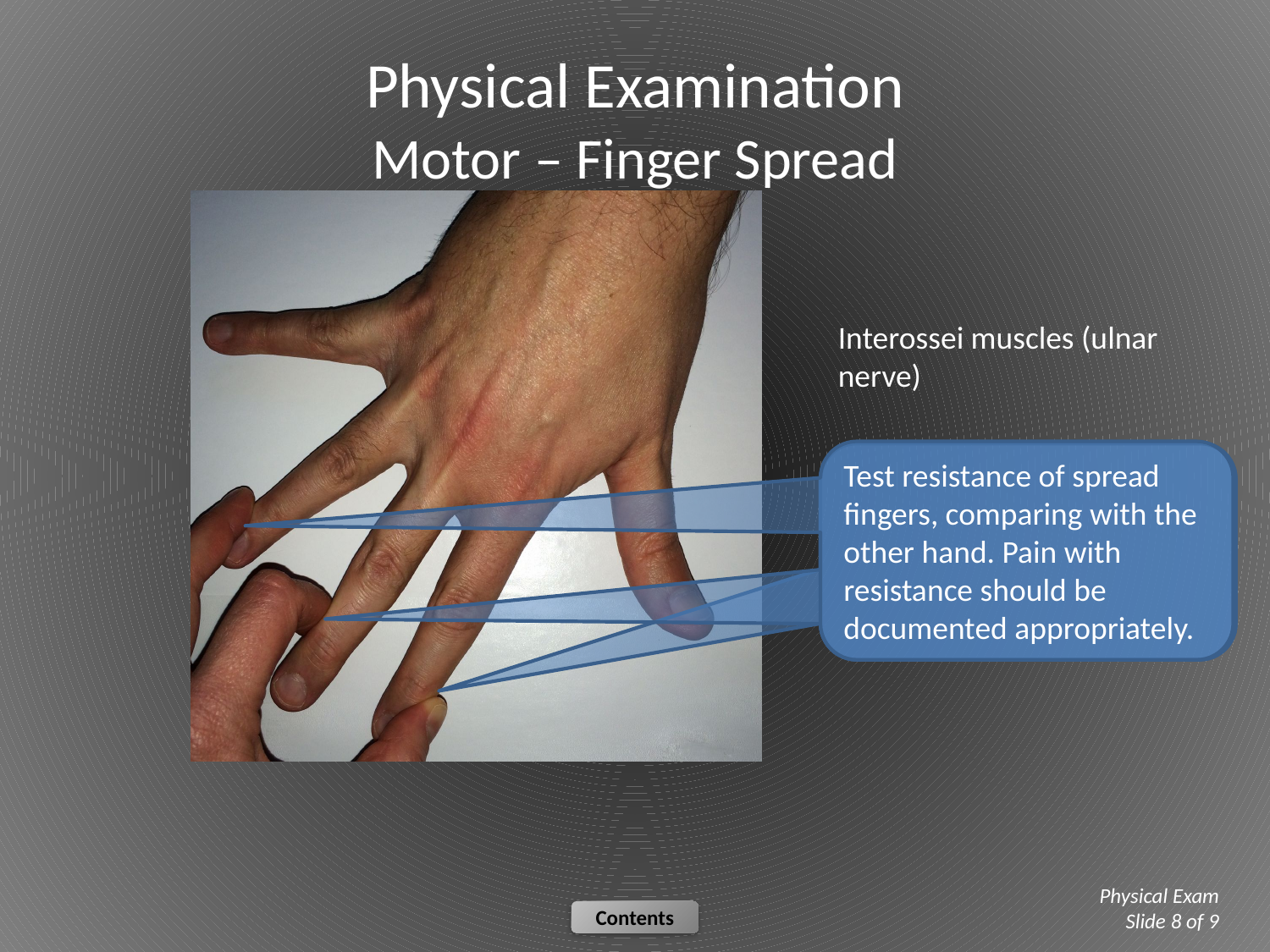

# Physical Examination Motor – Finger Spread
Interossei muscles (ulnar nerve)
Test resistance of spread fingers, comparing with the other hand. Pain with resistance should be documented appropriately.
Physical Exam
Slide 8 of 9
Contents

## Slide 13
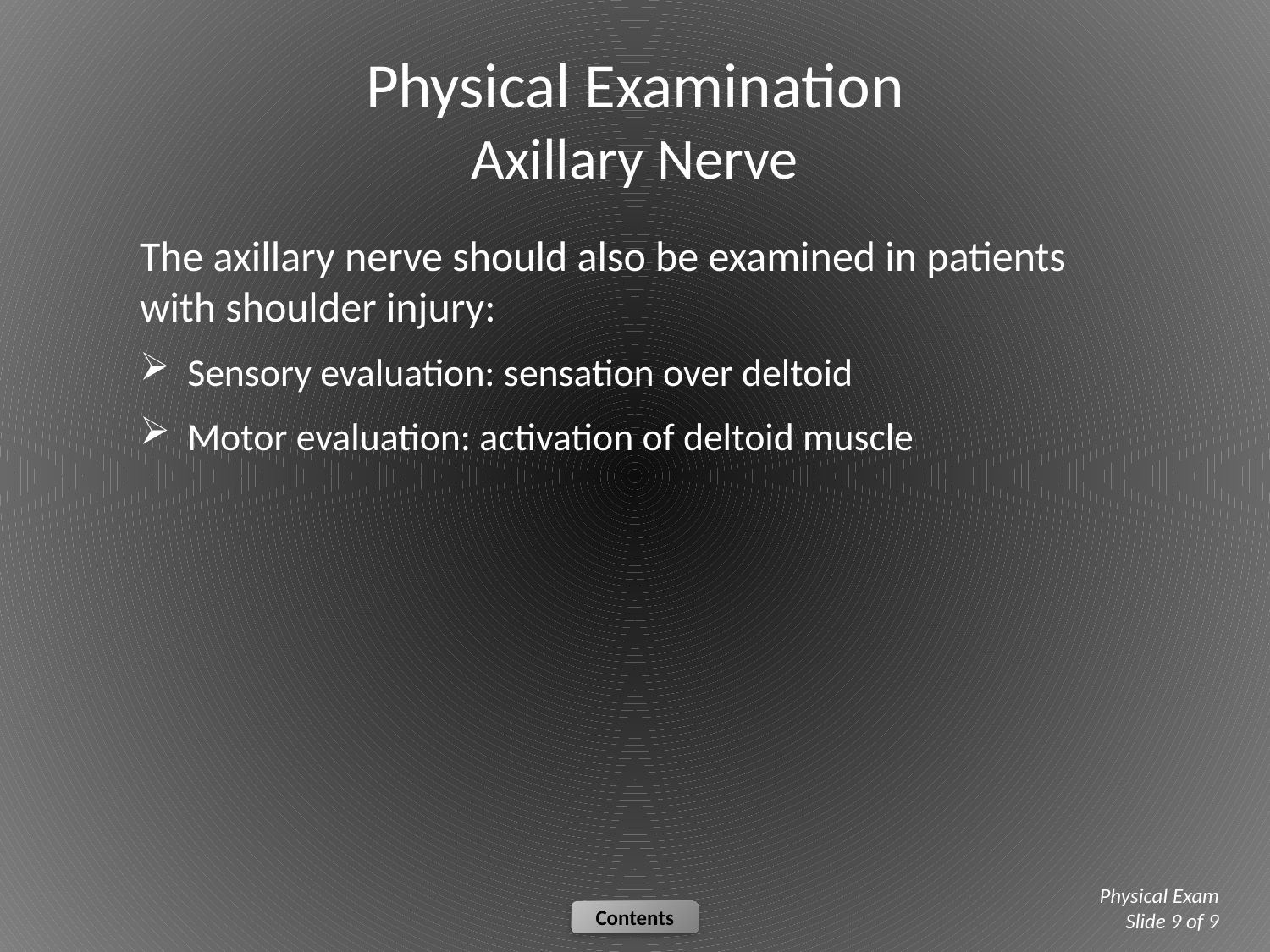

# Physical Examination Axillary Nerve
The axillary nerve should also be examined in patients with shoulder injury:
Sensory evaluation: sensation over deltoid
Motor evaluation: activation of deltoid muscle
Physical Exam
Slide 9 of 9
Contents

## Slide 14
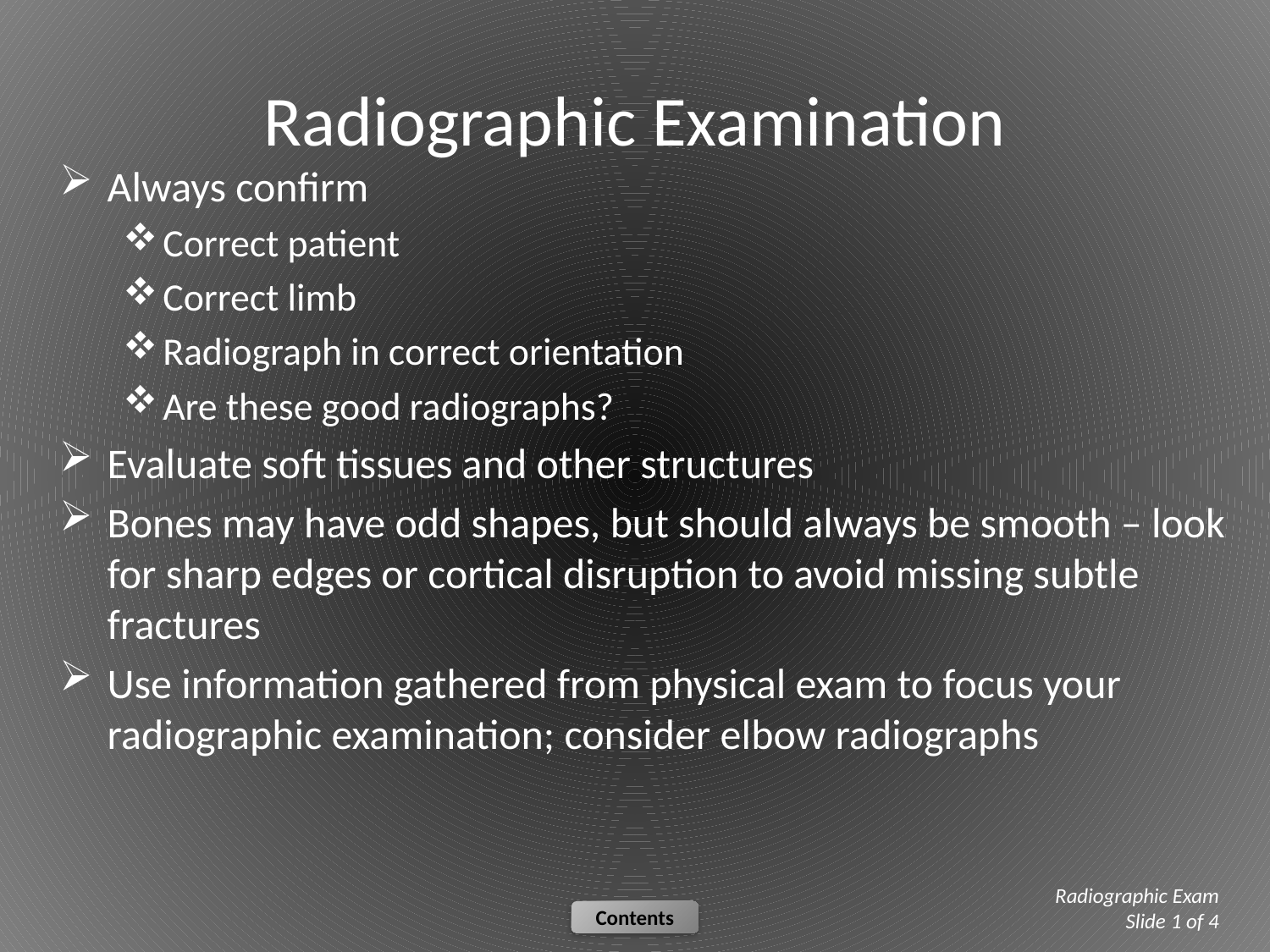

# Radiographic Examination
Always confirm
Correct patient
Correct limb
Radiograph in correct orientation
Are these good radiographs?
Evaluate soft tissues and other structures
Bones may have odd shapes, but should always be smooth – look for sharp edges or cortical disruption to avoid missing subtle fractures
Use information gathered from physical exam to focus your radiographic examination; consider elbow radiographs
Radiographic Exam
Slide 1 of 4
Contents

## Slide 15
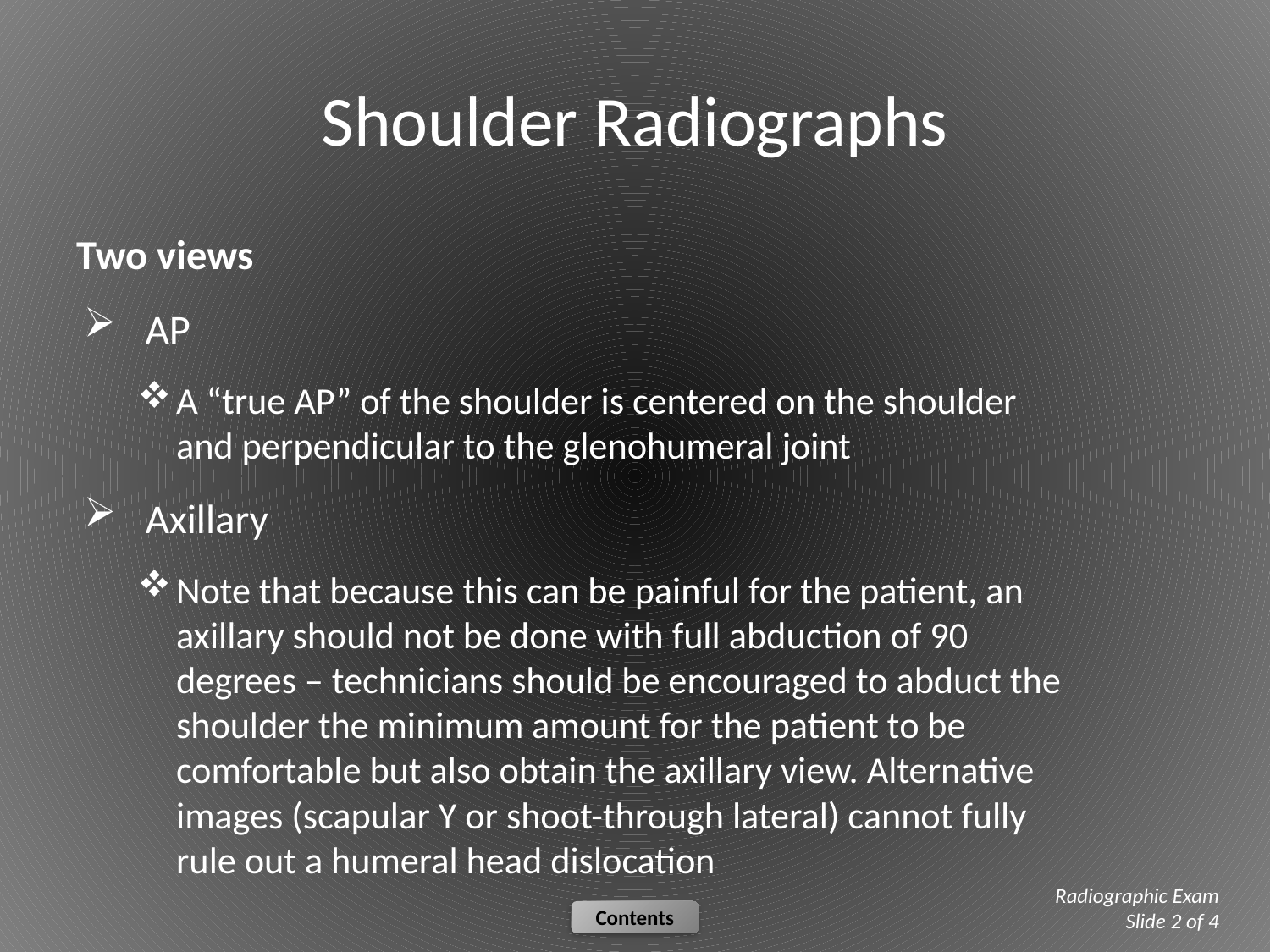

# Shoulder Radiographs
Two views
AP
A “true AP” of the shoulder is centered on the shoulder and perpendicular to the glenohumeral joint
Axillary
Note that because this can be painful for the patient, an axillary should not be done with full abduction of 90 degrees – technicians should be encouraged to abduct the shoulder the minimum amount for the patient to be comfortable but also obtain the axillary view. Alternative images (scapular Y or shoot-through lateral) cannot fully rule out a humeral head dislocation
Radiographic Exam
Slide 2 of 4
Contents

## Slide 16
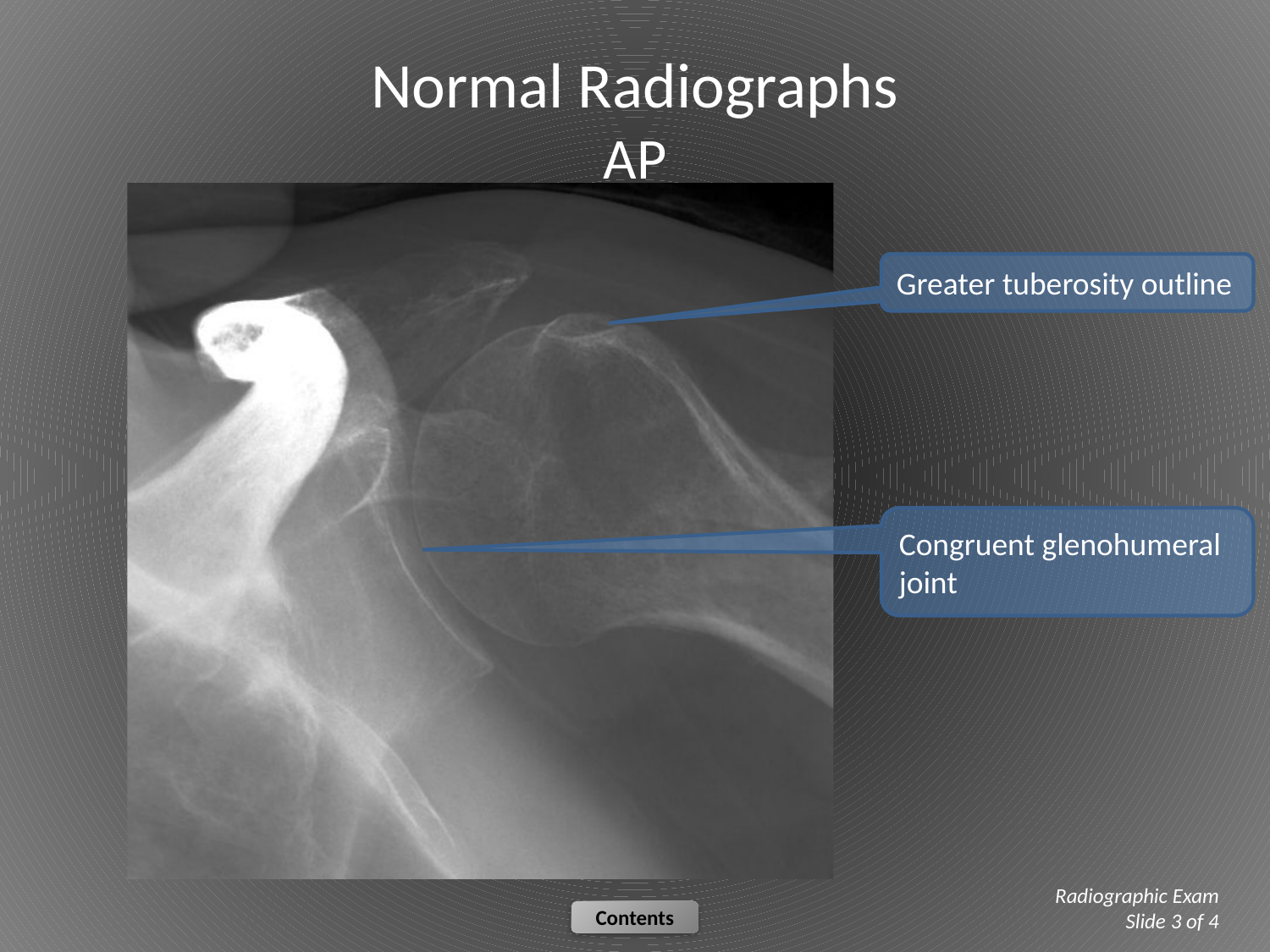

# Normal Radiographs AP
Greater tuberosity outline
Congruent glenohumeral joint
Radiographic Exam
Slide 3 of 4
Contents

## Slide 17
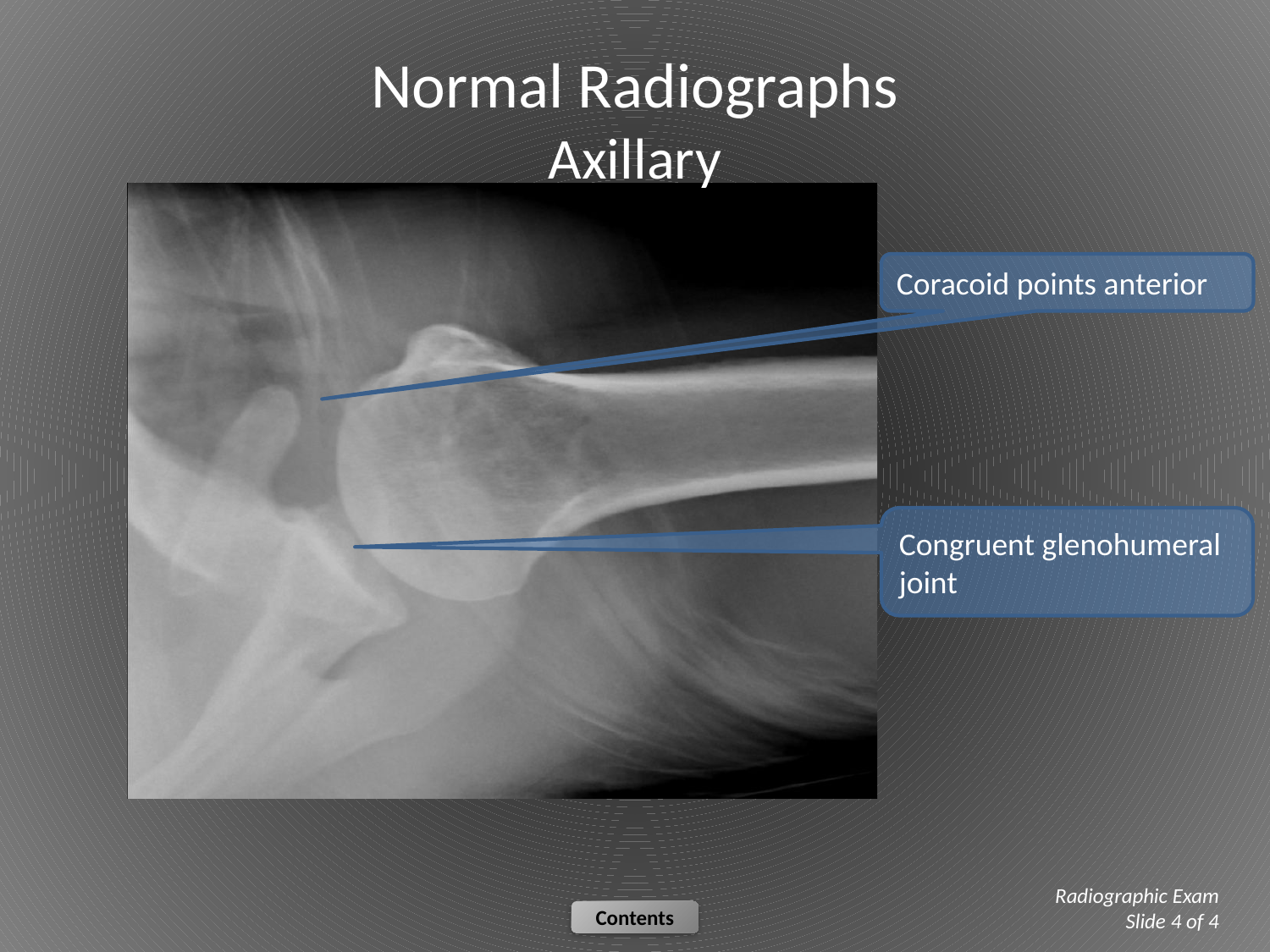

# Normal Radiographs Axillary
Coracoid points anterior
Congruent glenohumeral joint
Radiographic Exam
Slide 4 of 4
Contents

## Slide 18
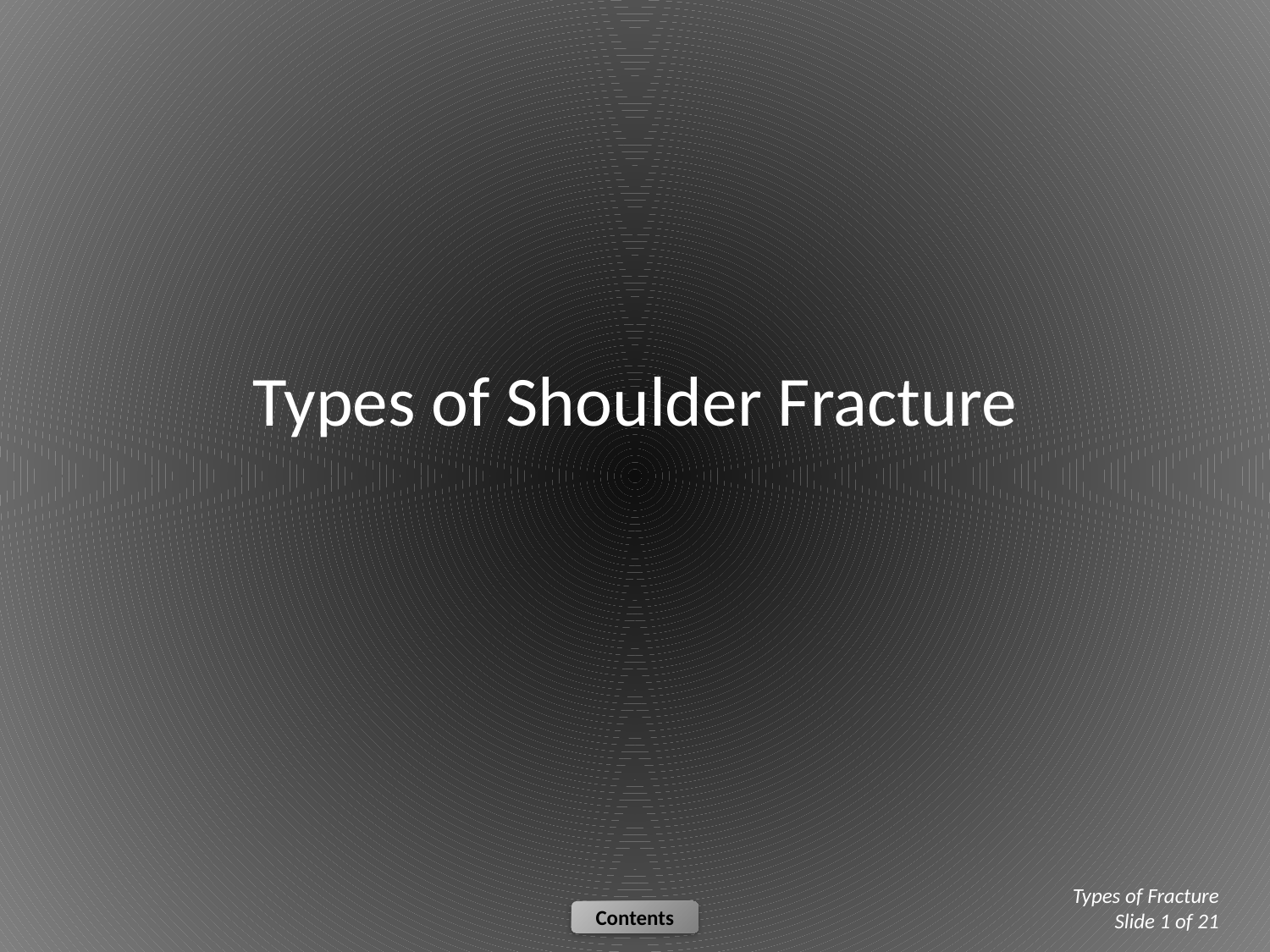

# Types of Shoulder Fracture
Types of Fracture
Slide 1 of 21
Contents

## Slide 19
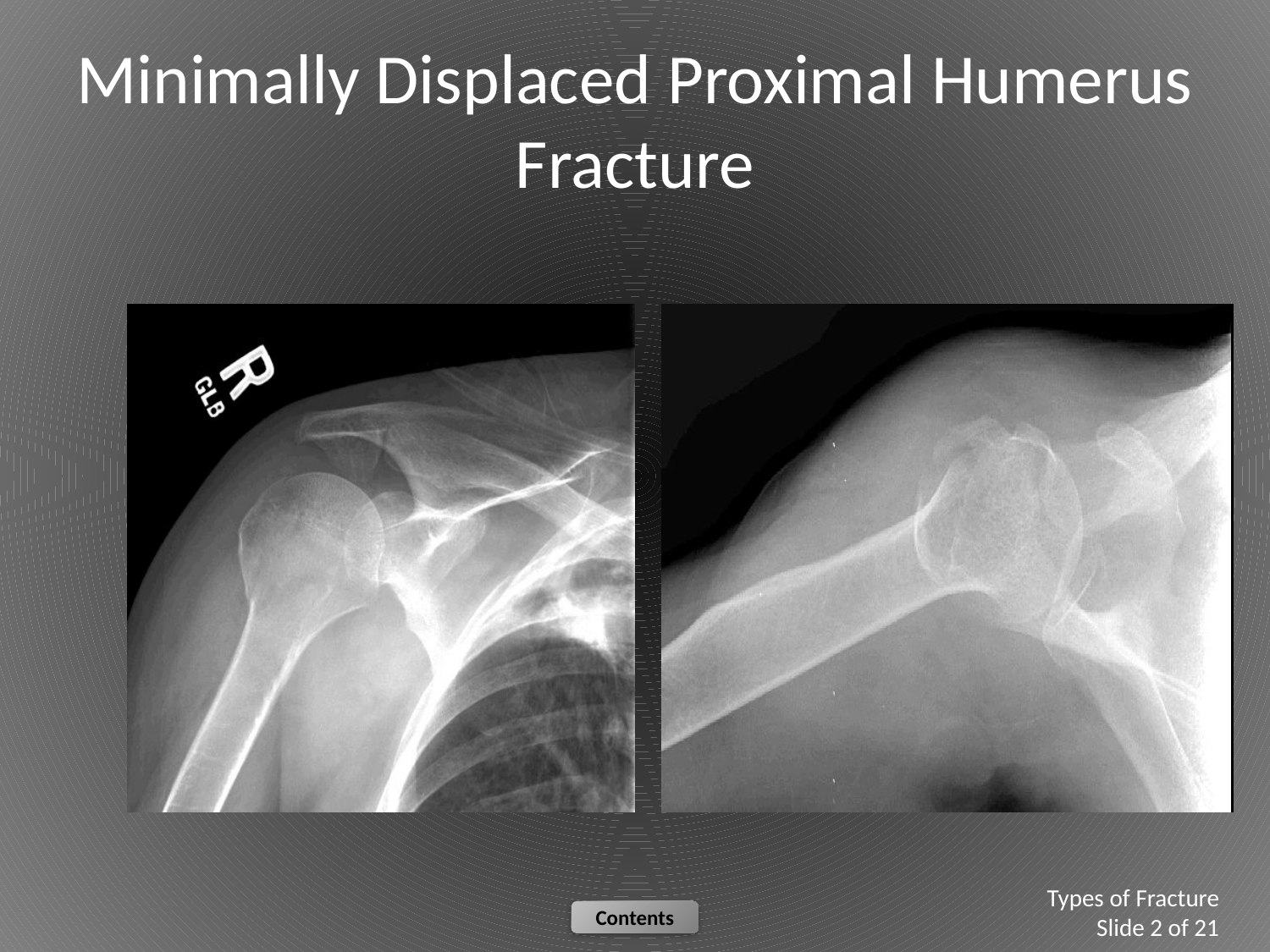

# Minimally Displaced Proximal Humerus Fracture
Types of Fracture
Slide 2 of 21
Contents

## Slide 20
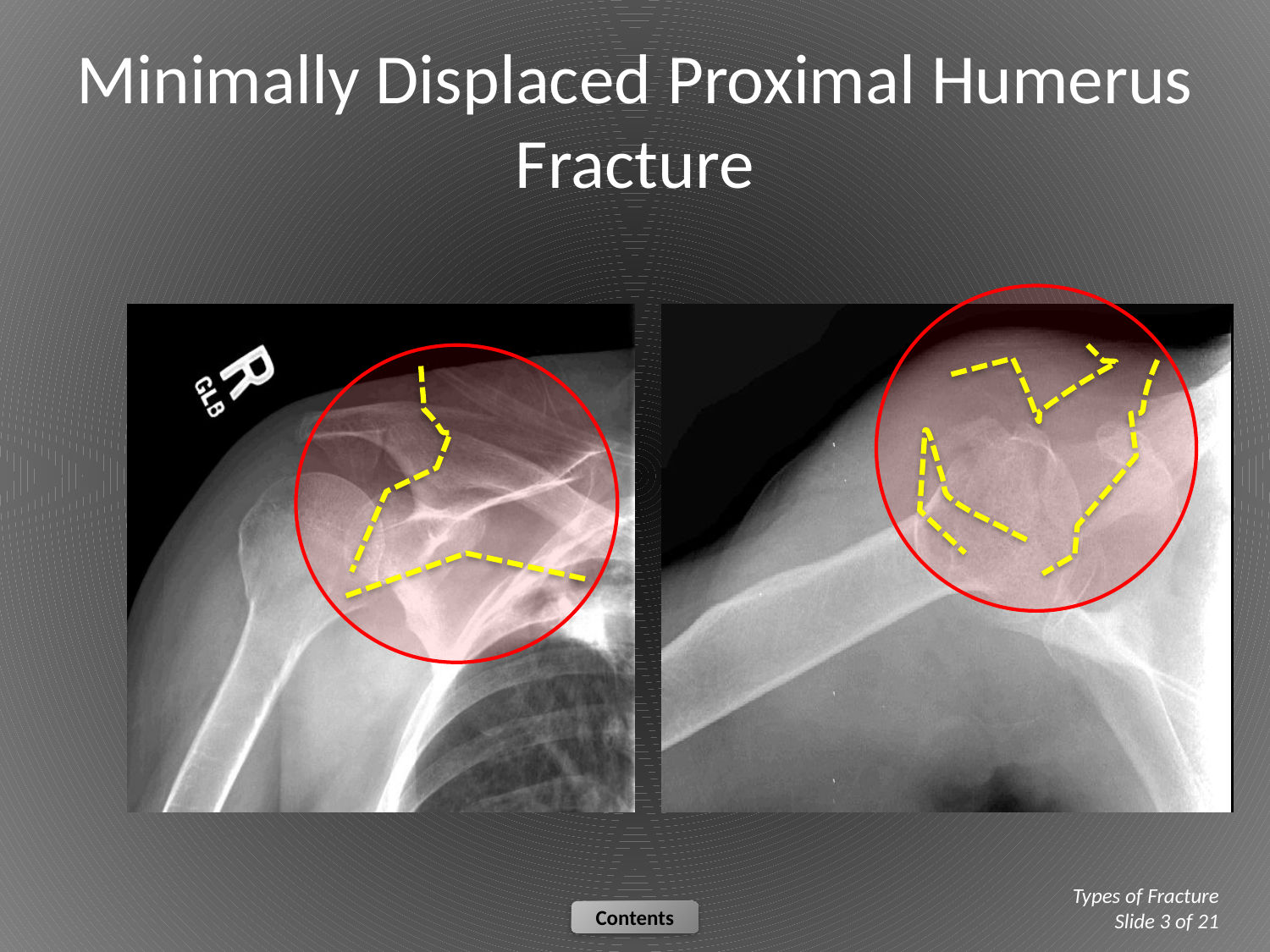

# Minimally Displaced Proximal Humerus Fracture
Types of Fracture
Slide 3 of 21
Contents

## Slide 21
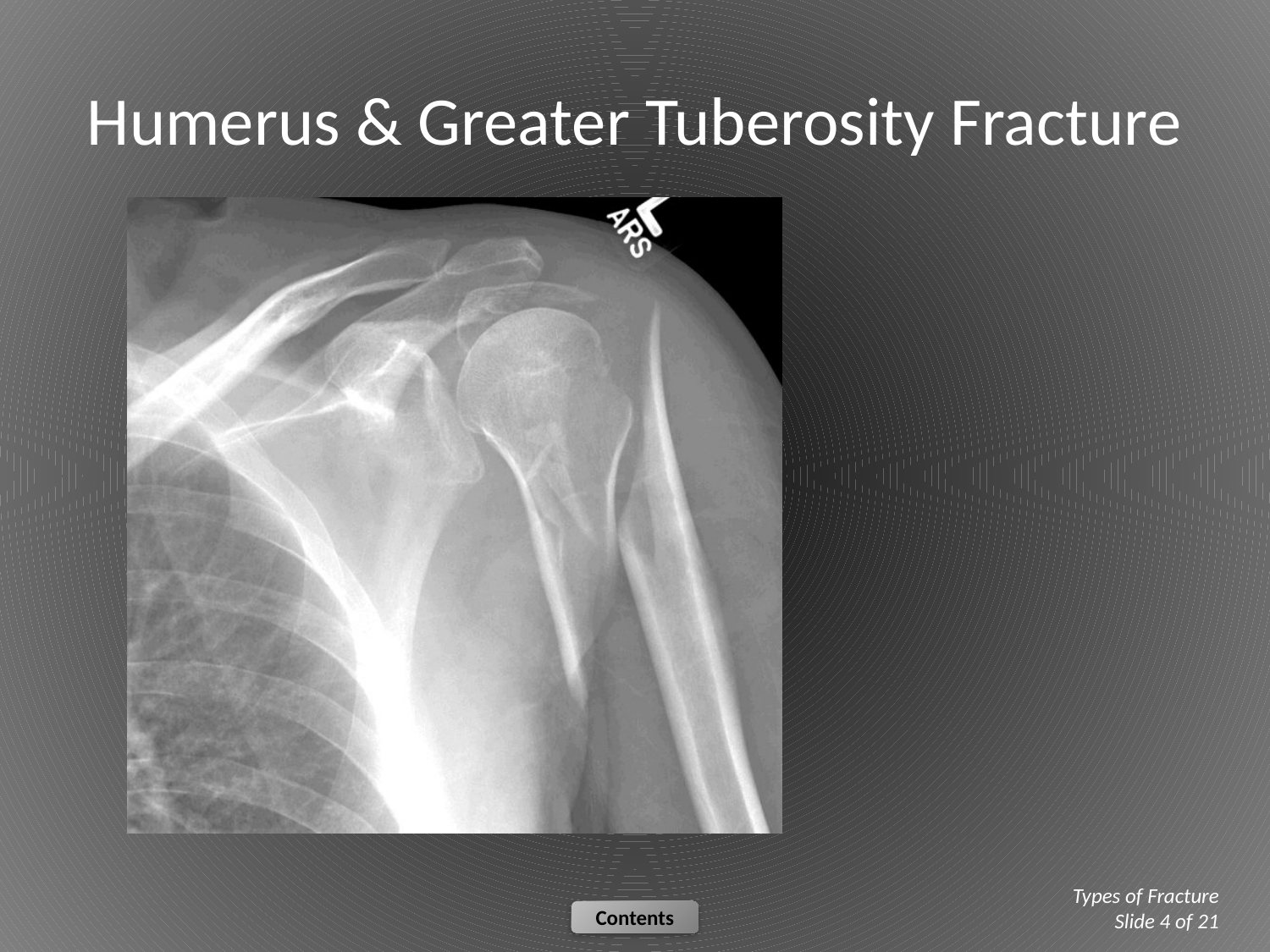

# Humerus & Greater Tuberosity Fracture
Types of Fracture
Slide 4 of 21
Contents

## Slide 22
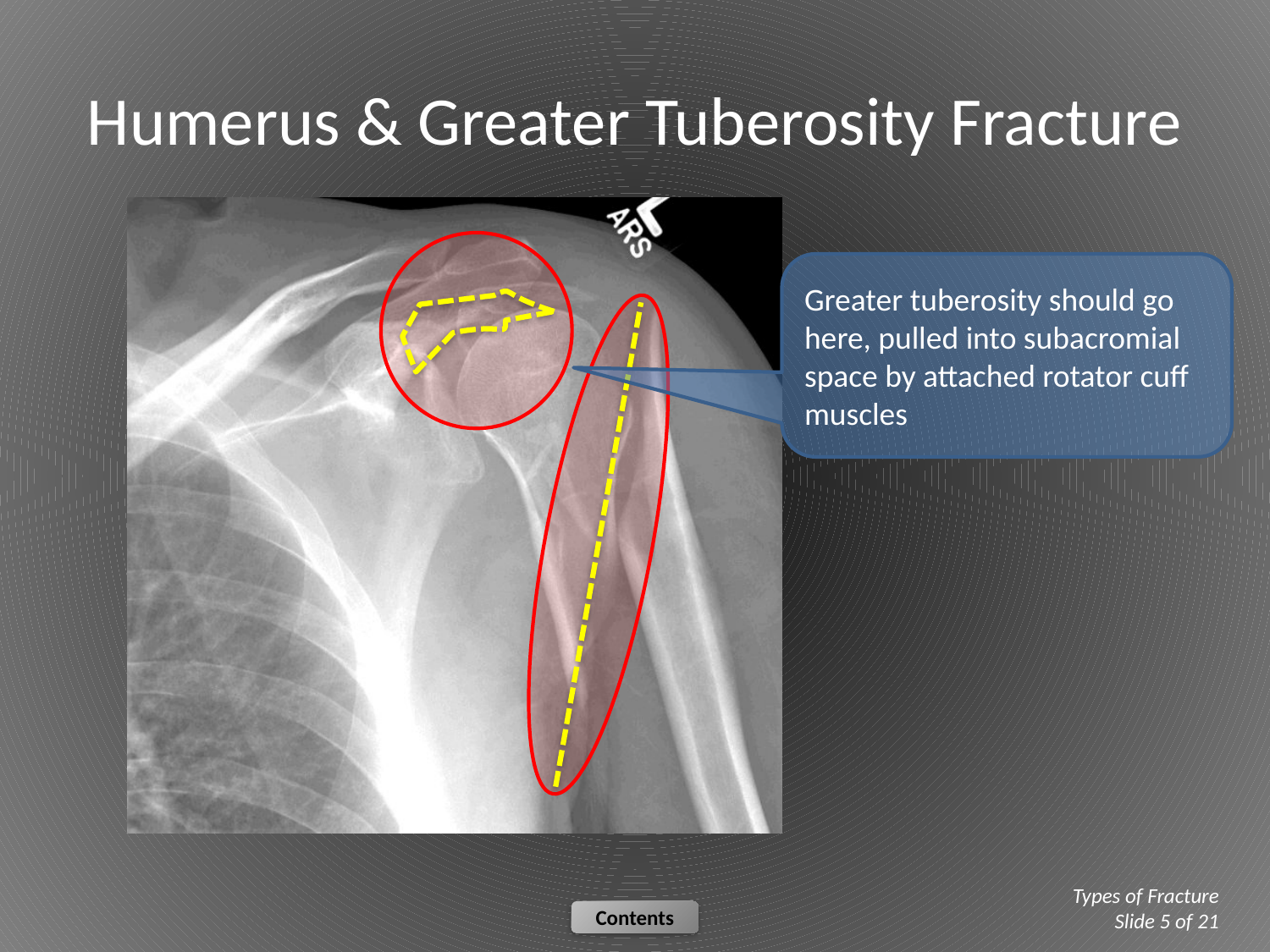

# Humerus & Greater Tuberosity Fracture
Greater tuberosity should go here, pulled into subacromial
space by attached rotator cuff muscles
Types of Fracture
Slide 5 of 21
Contents

## Slide 23
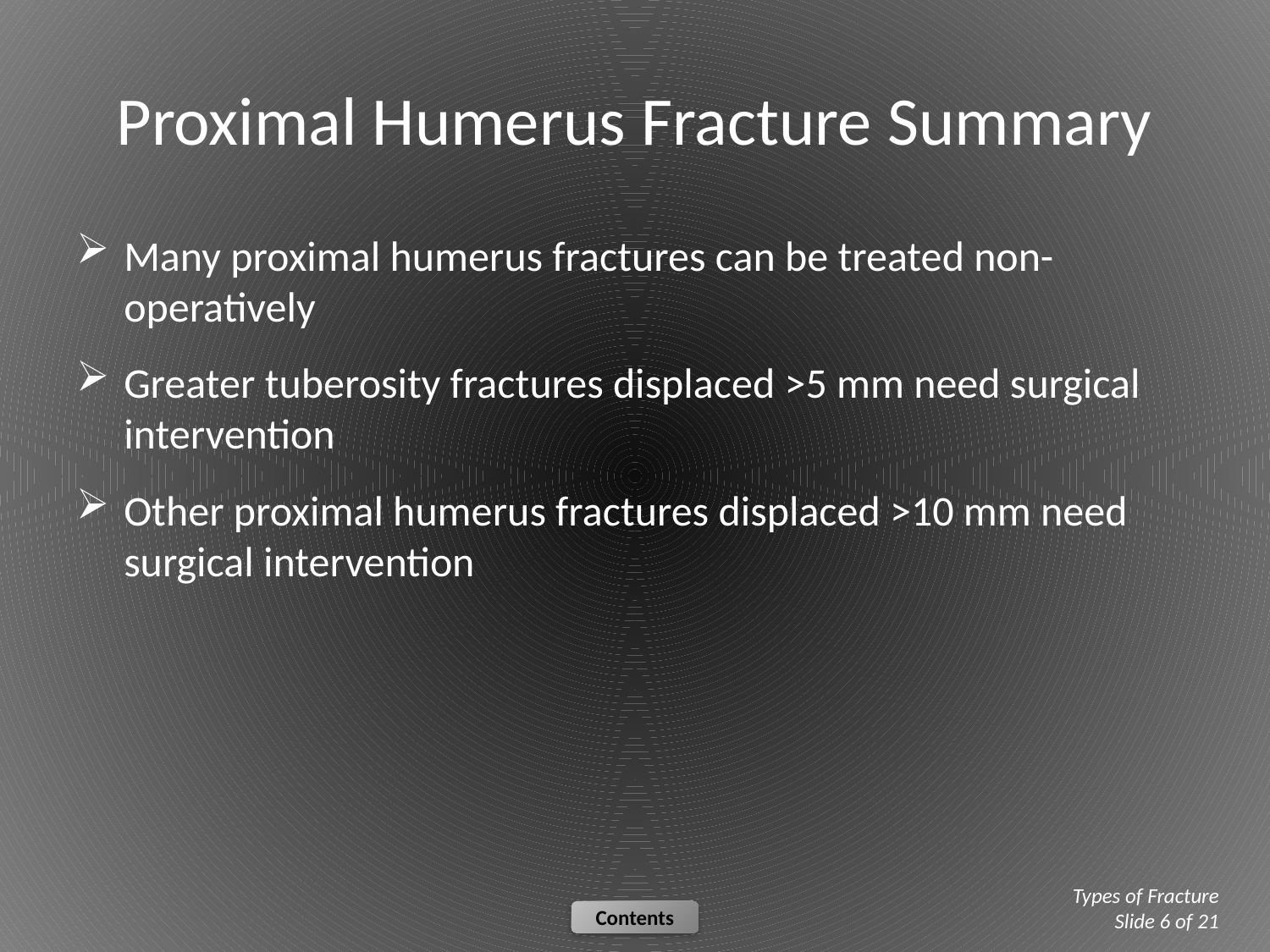

# Proximal Humerus Fracture Summary
Many proximal humerus fractures can be treated non-operatively
Greater tuberosity fractures displaced >5 mm need surgical intervention
Other proximal humerus fractures displaced >10 mm need surgical intervention
Types of Fracture
Slide 6 of 21
Contents

## Slide 24
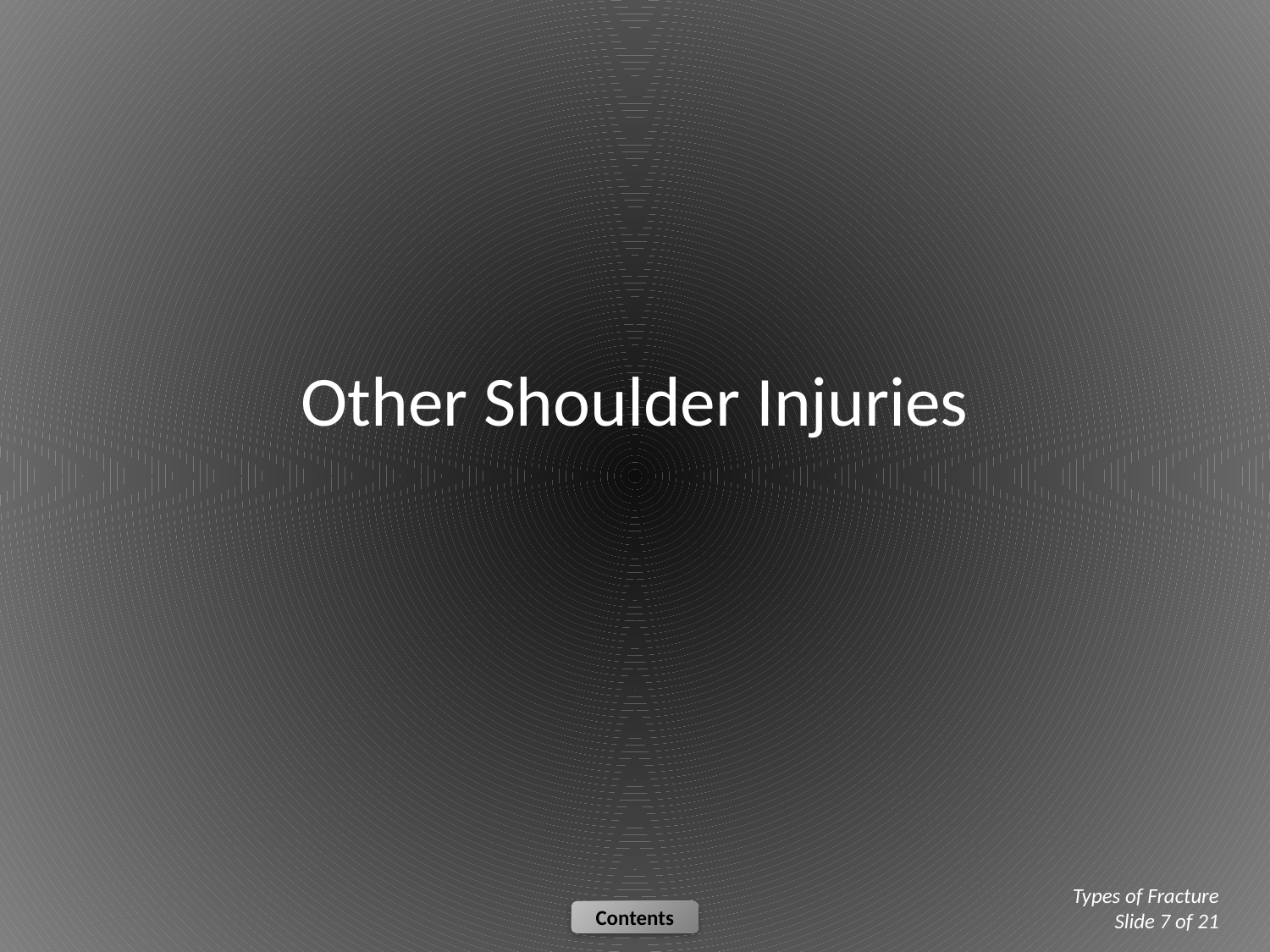

# Other Shoulder Injuries
Types of Fracture
Slide 7 of 21
Contents

## Slide 25
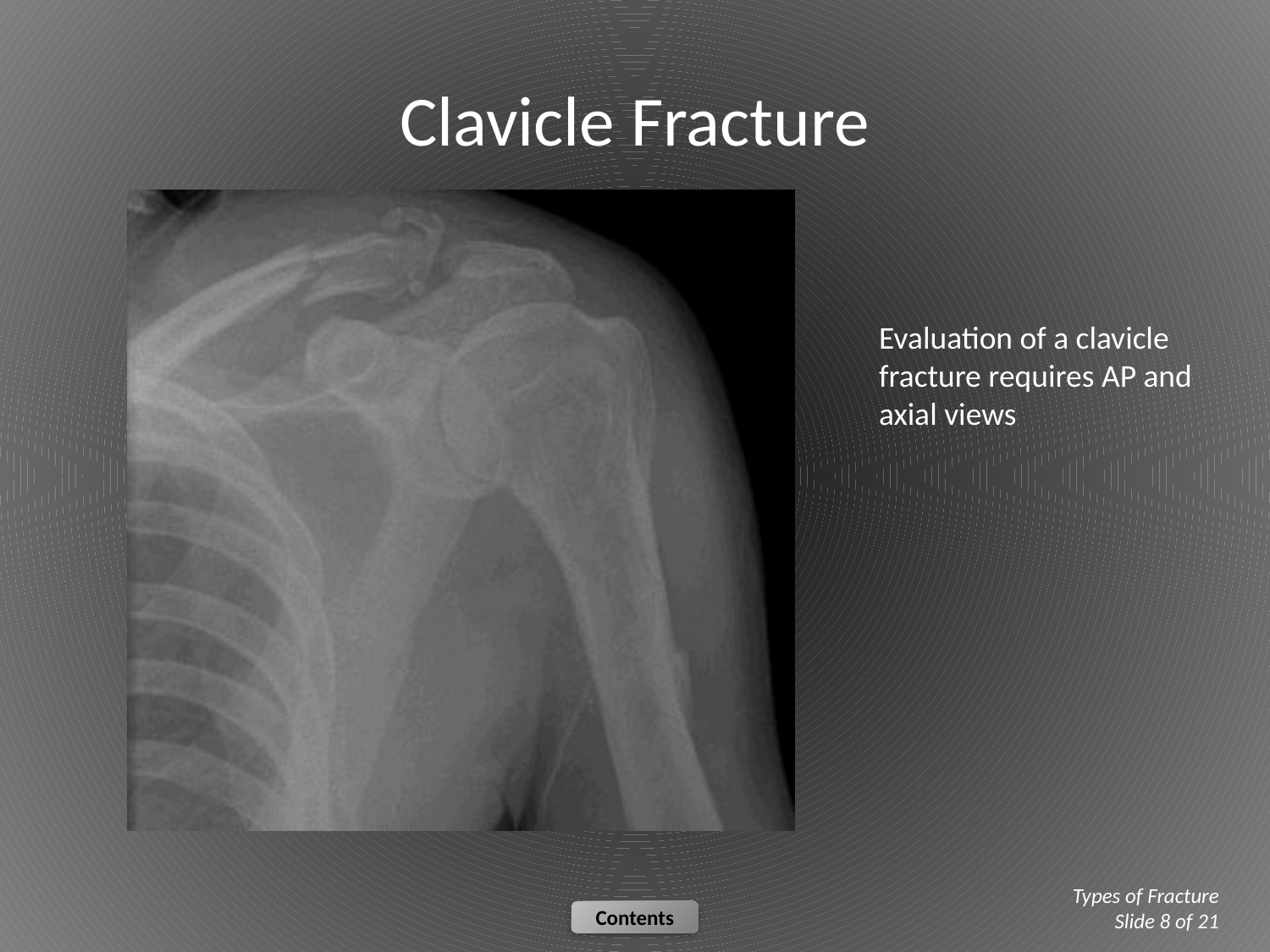

# Clavicle Fracture
Evaluation of a clavicle fracture requires AP and axial views
Types of Fracture
Slide 8 of 21
Contents

## Slide 26
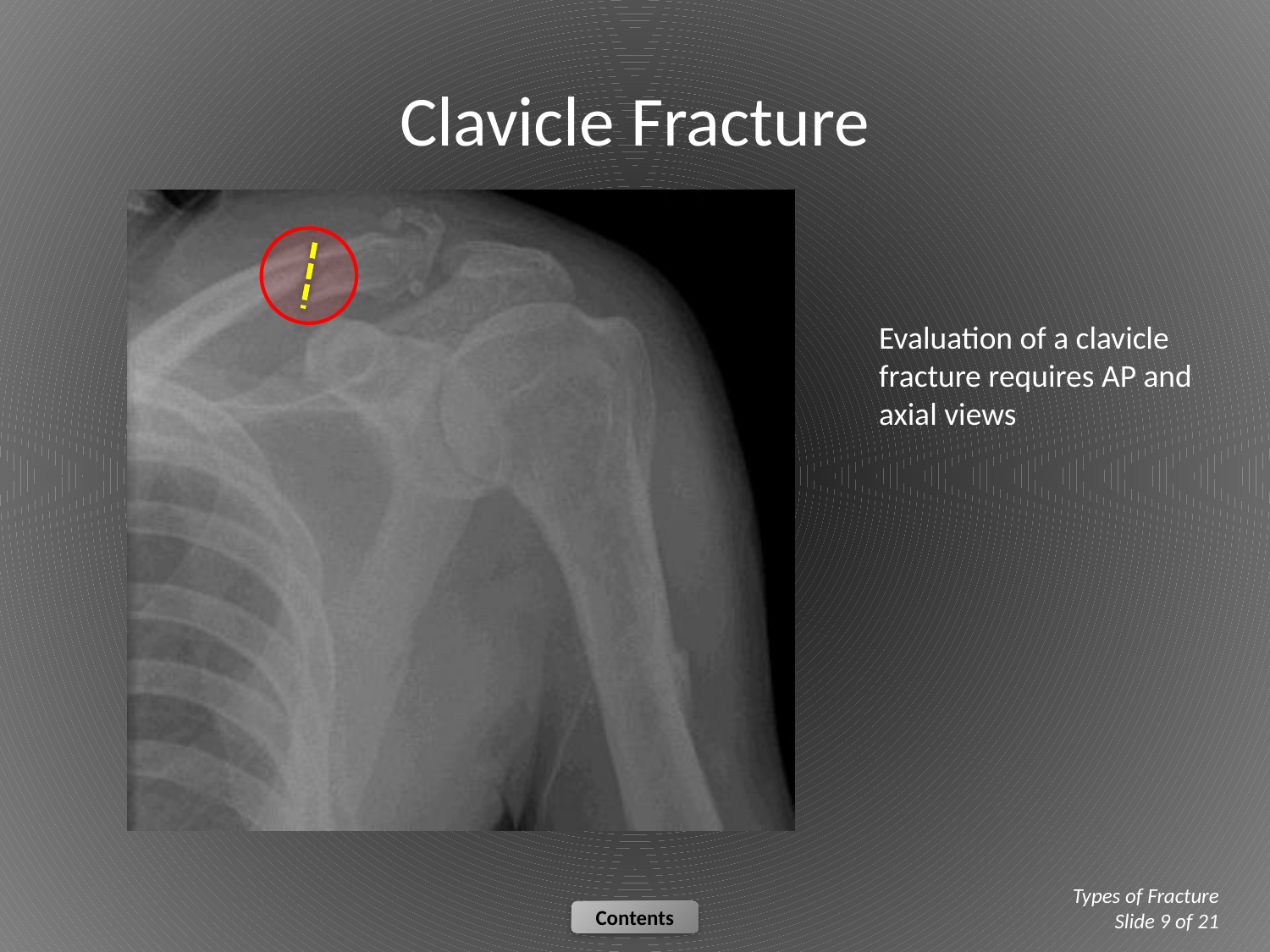

# Clavicle Fracture
Evaluation of a clavicle fracture requires AP and axial views
Types of Fracture
Slide 9 of 21
Contents

## Slide 27
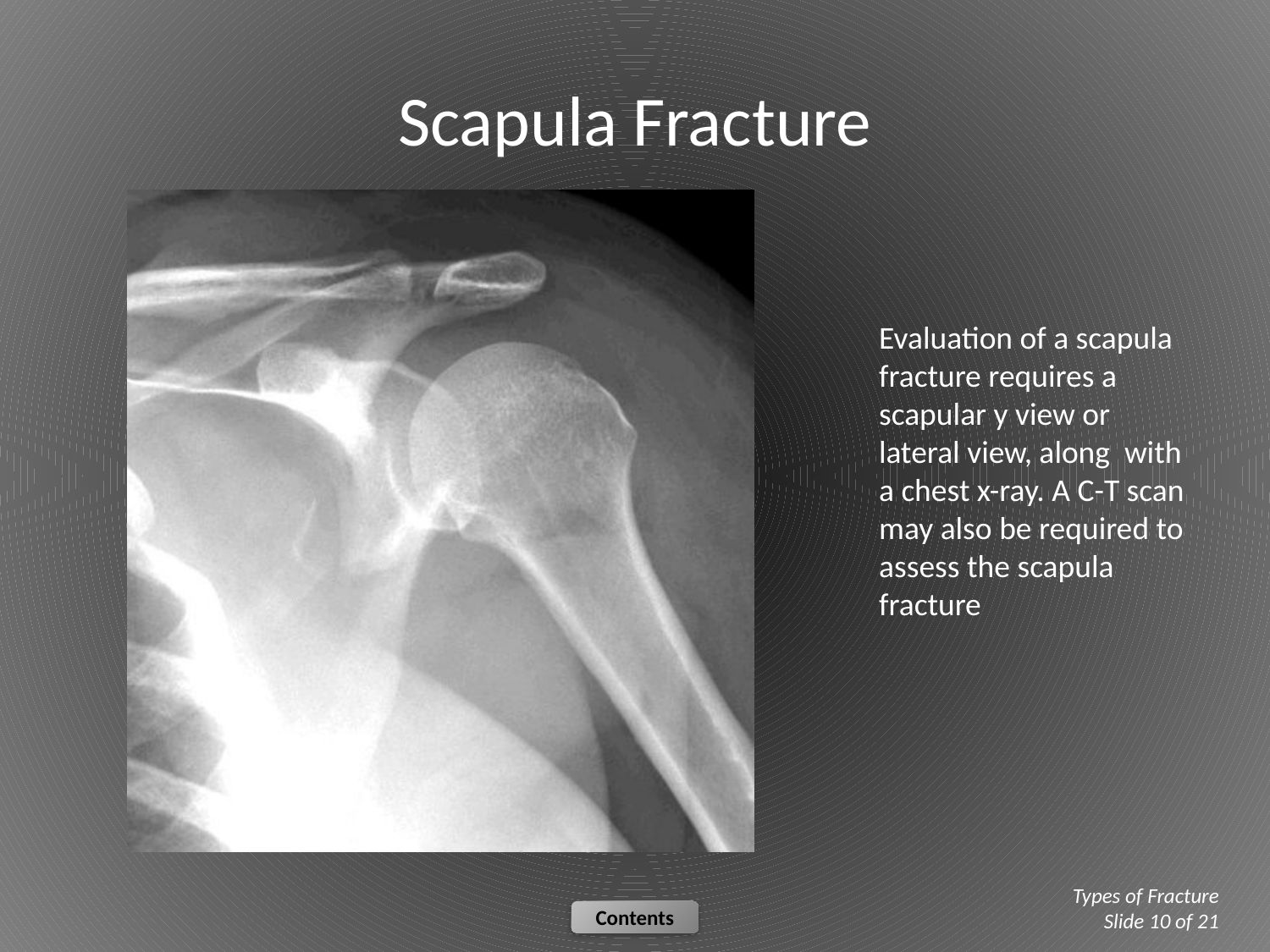

# Scapula Fracture
Evaluation of a scapula fracture requires a scapular y view or lateral view, along with a chest x-ray. A C-T scan may also be required to assess the scapula fracture
Types of Fracture
Slide 10 of 21
Contents

## Slide 28
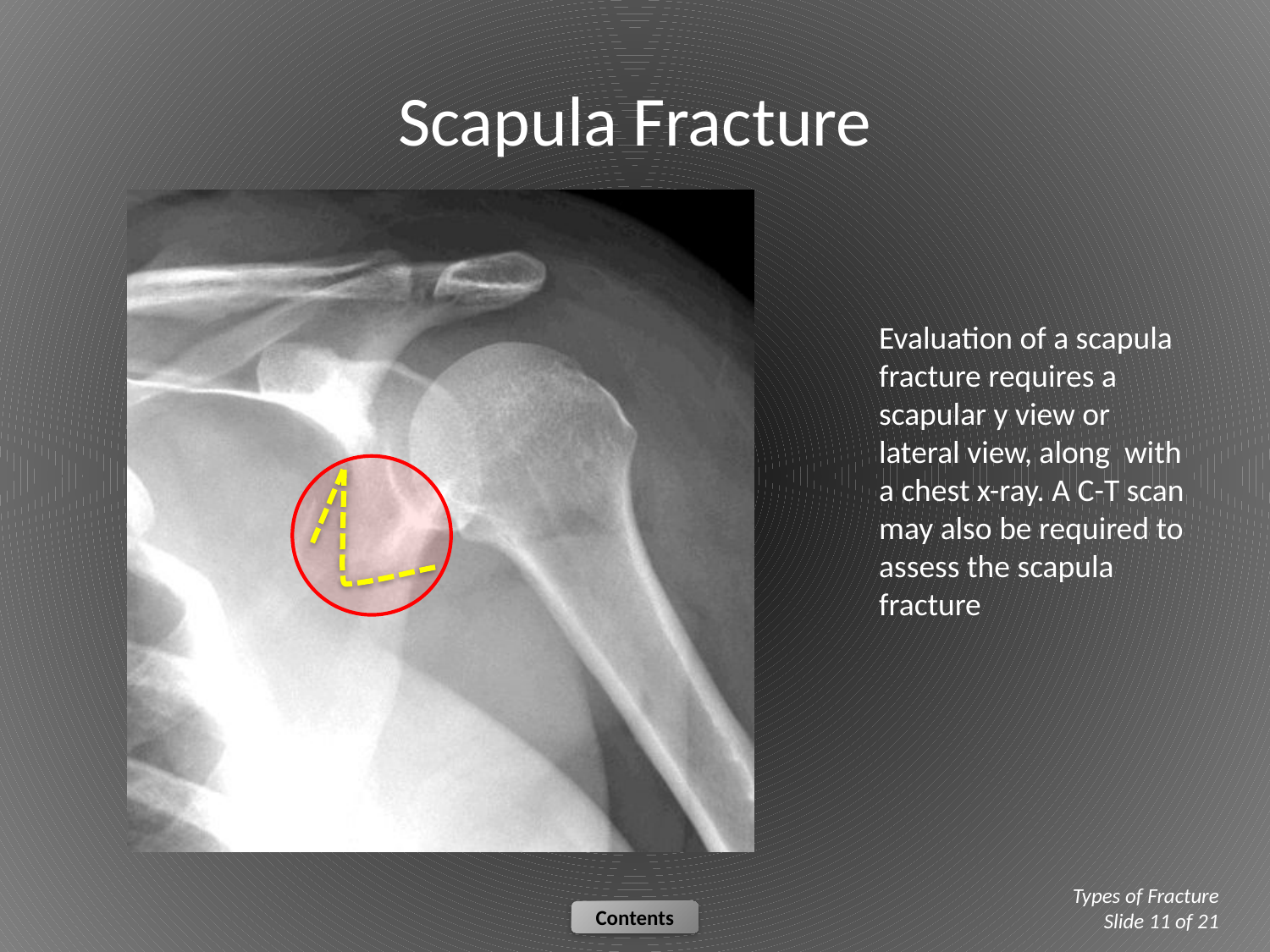

# Scapula Fracture
Evaluation of a scapula fracture requires a scapular y view or lateral view, along with a chest x-ray. A C-T scan may also be required to assess the scapula fracture
Types of Fracture
Slide 11 of 21
Contents

## Slide 29
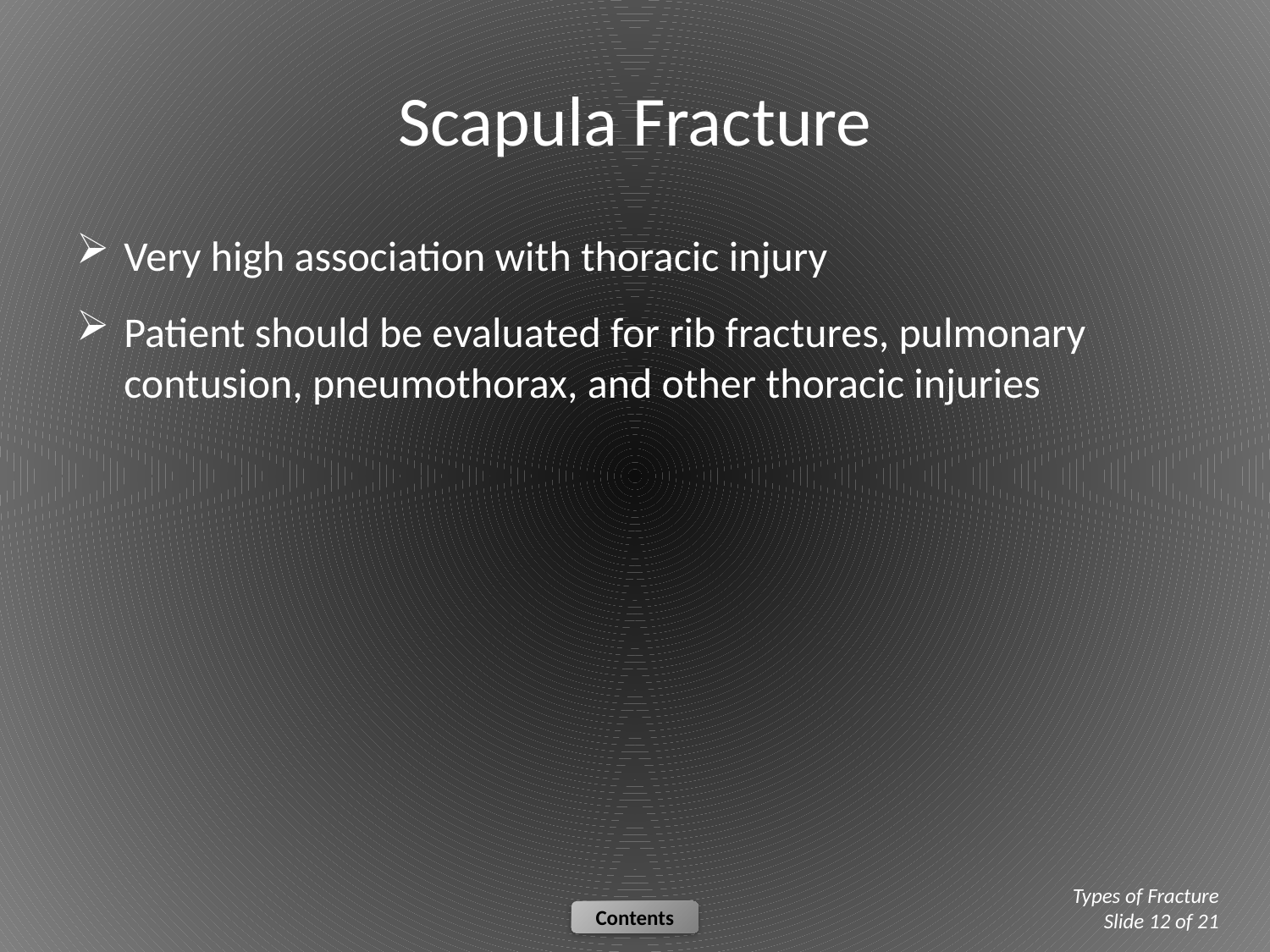

# Scapula Fracture
Very high association with thoracic injury
Patient should be evaluated for rib fractures, pulmonary contusion, pneumothorax, and other thoracic injuries
Types of Fracture
Slide 12 of 21
Contents

## Slide 30
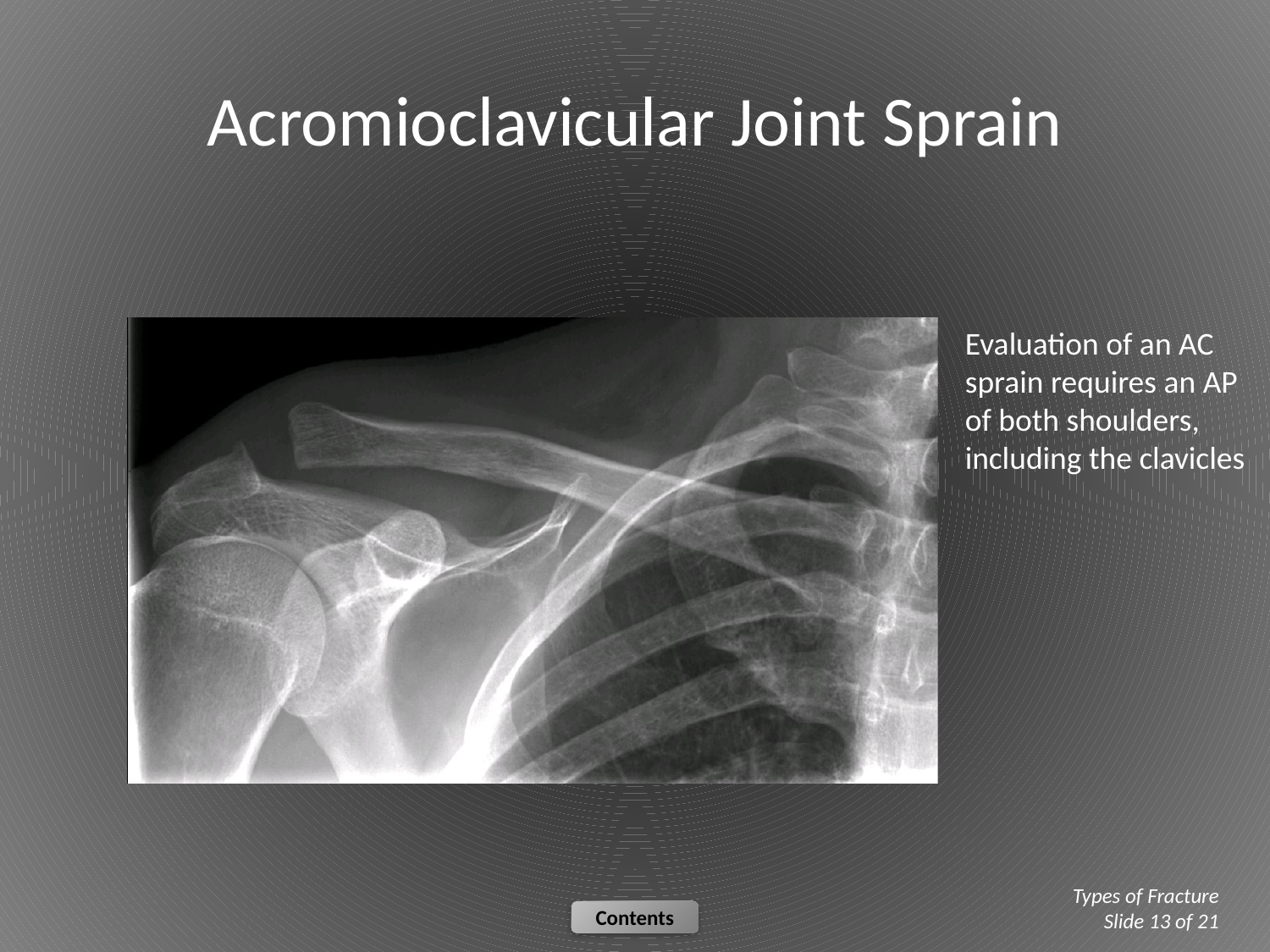

# Acromioclavicular Joint Sprain
Evaluation of an AC sprain requires an AP of both shoulders, including the clavicles
Types of Fracture
Slide 13 of 21
Contents

## Slide 31
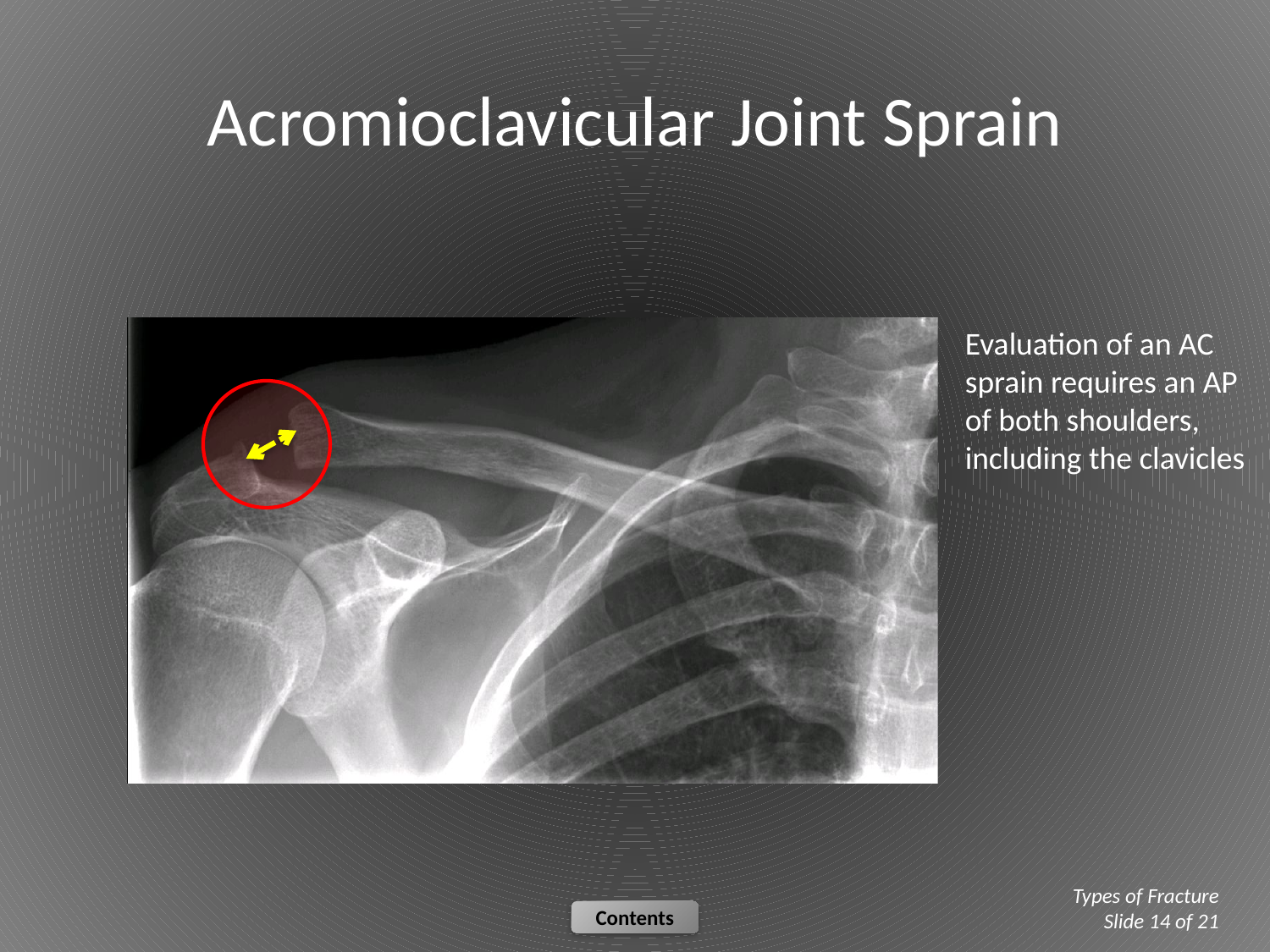

# Acromioclavicular Joint Sprain
Evaluation of an AC sprain requires an AP of both shoulders, including the clavicles
Types of Fracture
Slide 14 of 21
Contents

## Slide 32
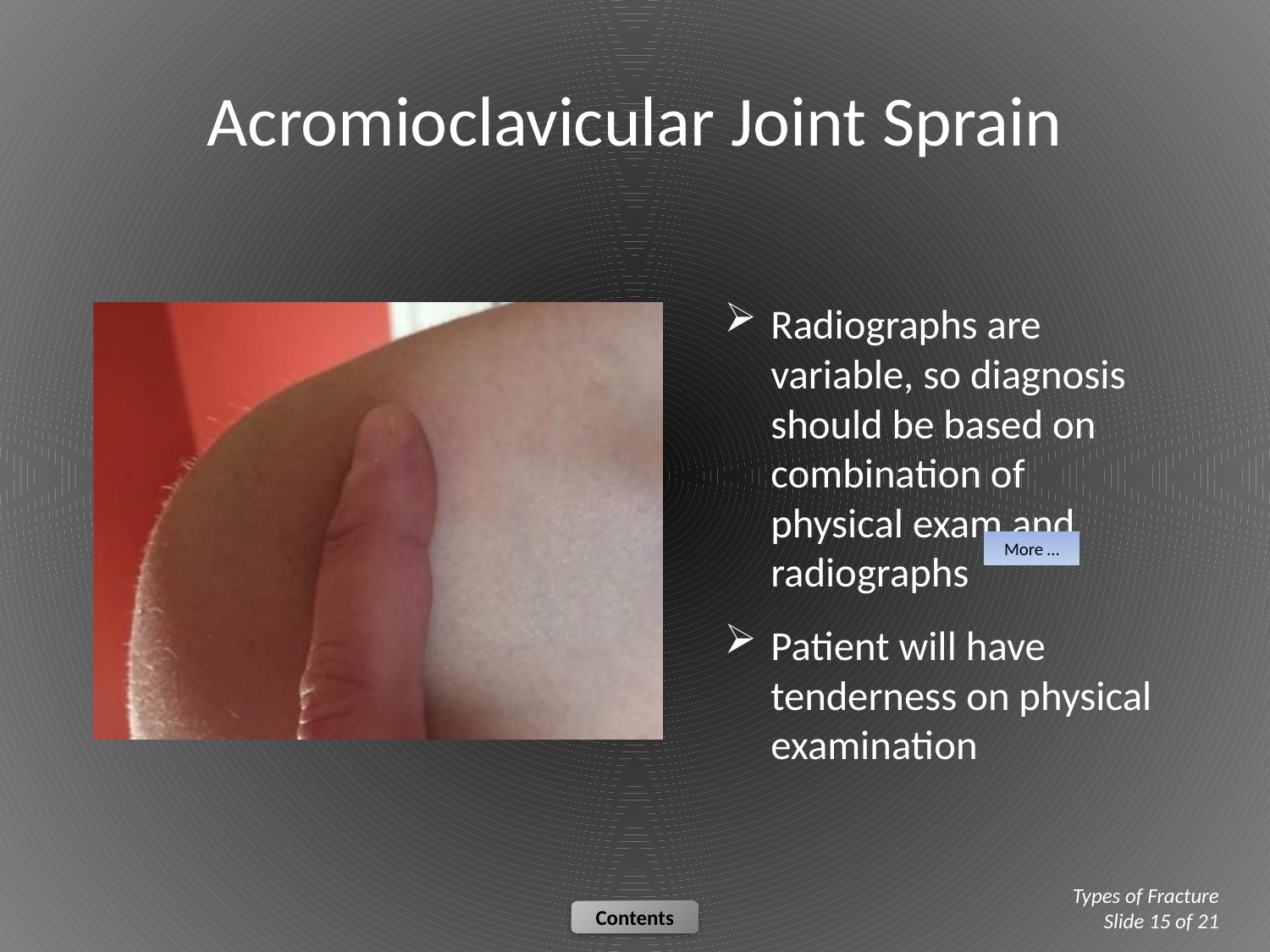

# Acromioclavicular Joint Sprain
Radiographs are variable, so diagnosis should be based on combination of physical exam and radiographs
Patient will have tenderness on physical examination
More …
Types of Fracture
Slide 15 of 21
Contents

## Slide 33
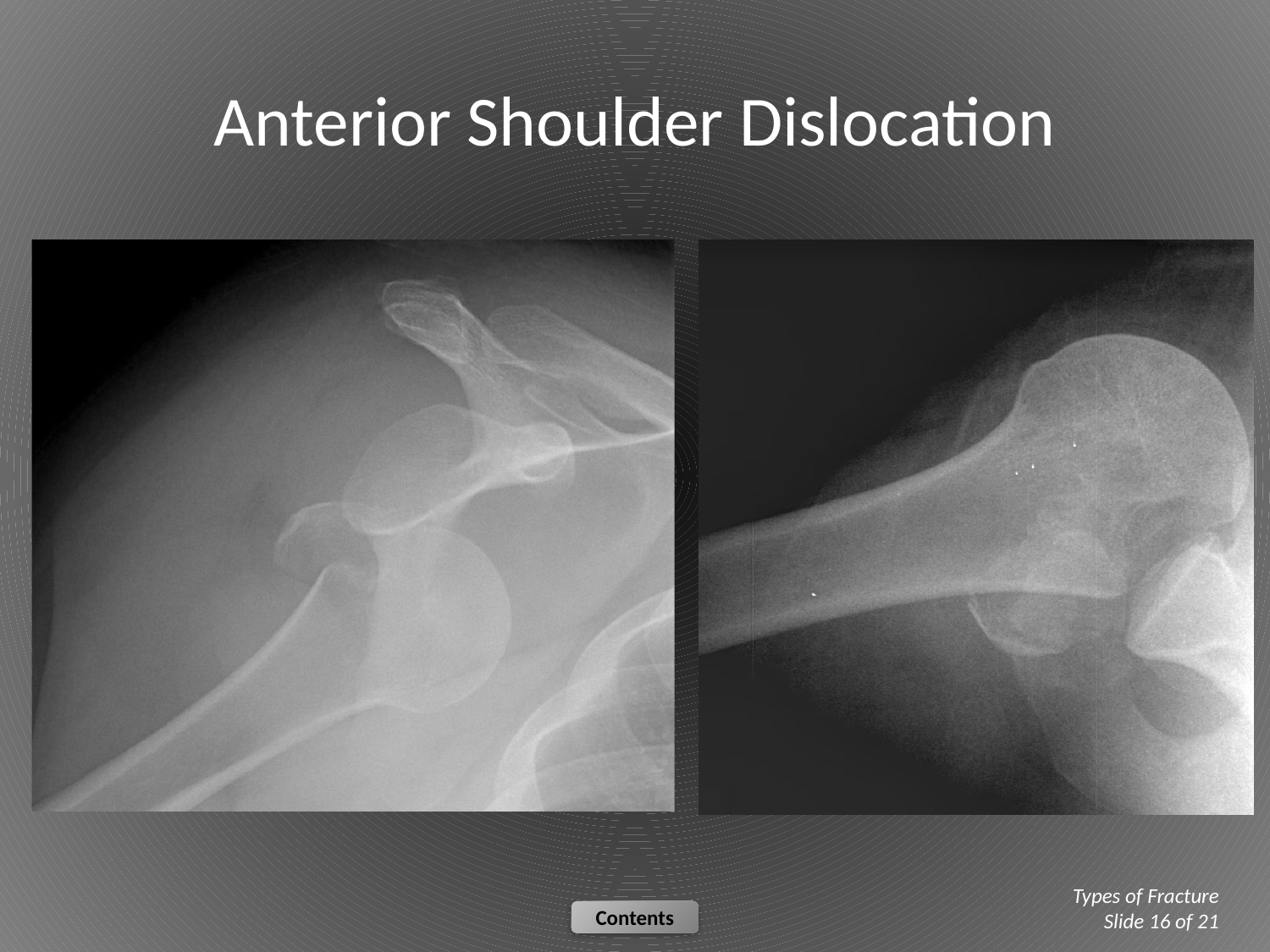

# Anterior Shoulder Dislocation
Types of Fracture
Slide 16 of 21
Contents

## Slide 34
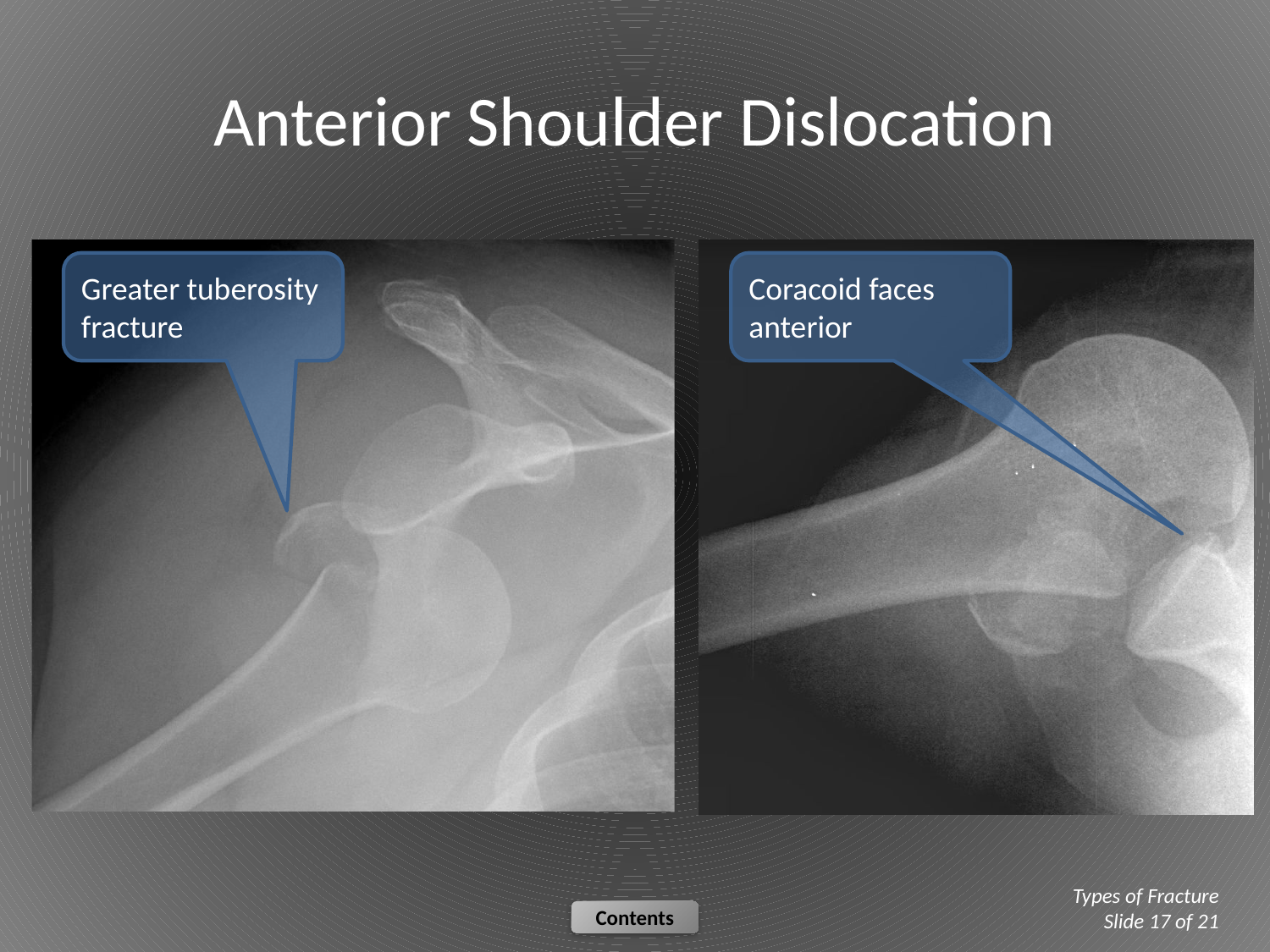

# Anterior Shoulder Dislocation
Greater tuberosity fracture
Coracoid faces anterior
Types of Fracture
Slide 17 of 21
Contents

## Slide 35
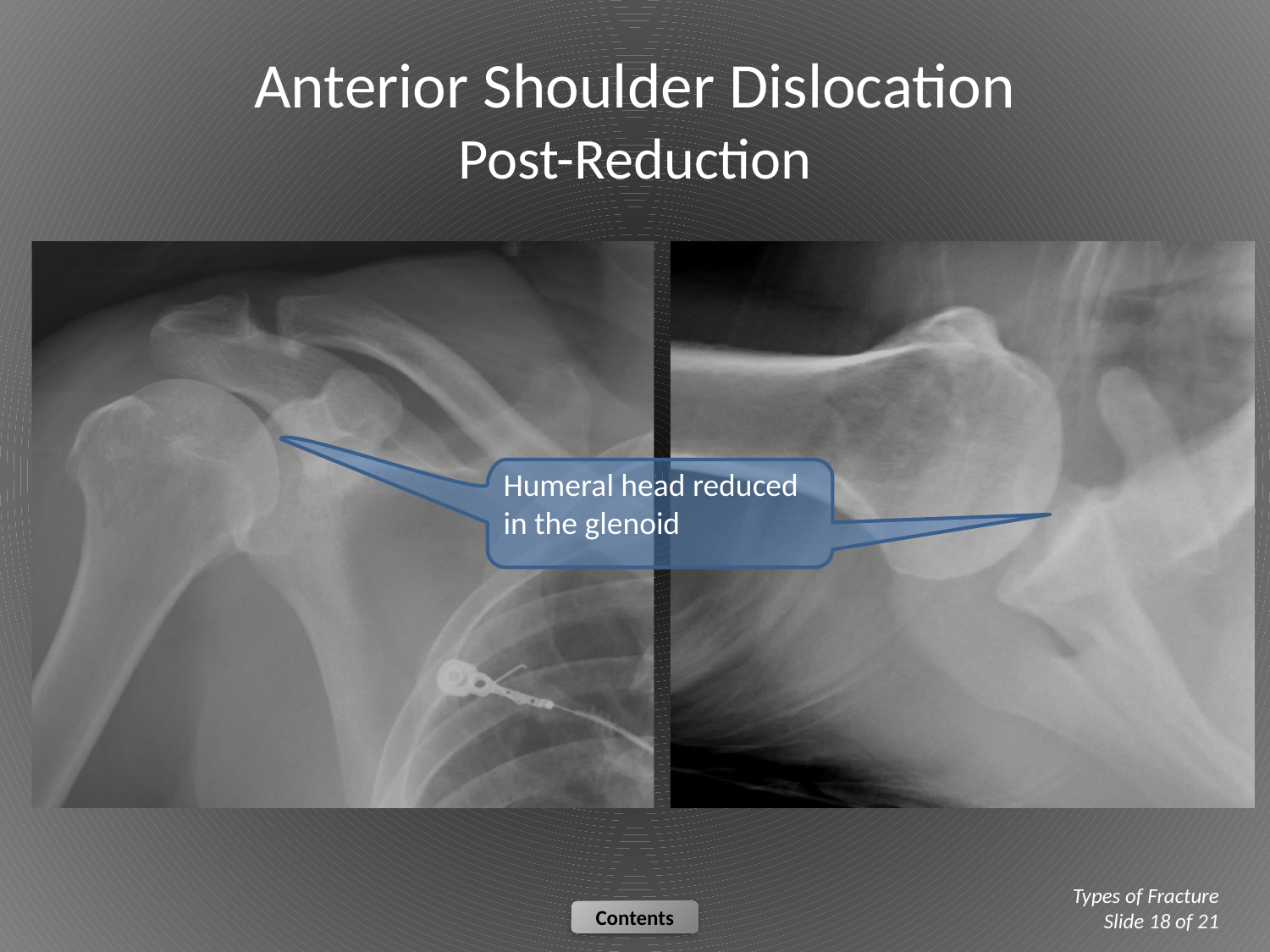

# Anterior Shoulder Dislocation Post-Reduction
Humeral head reduced
in the glenoid
Types of Fracture
Slide 18 of 21
Contents

## Slide 36
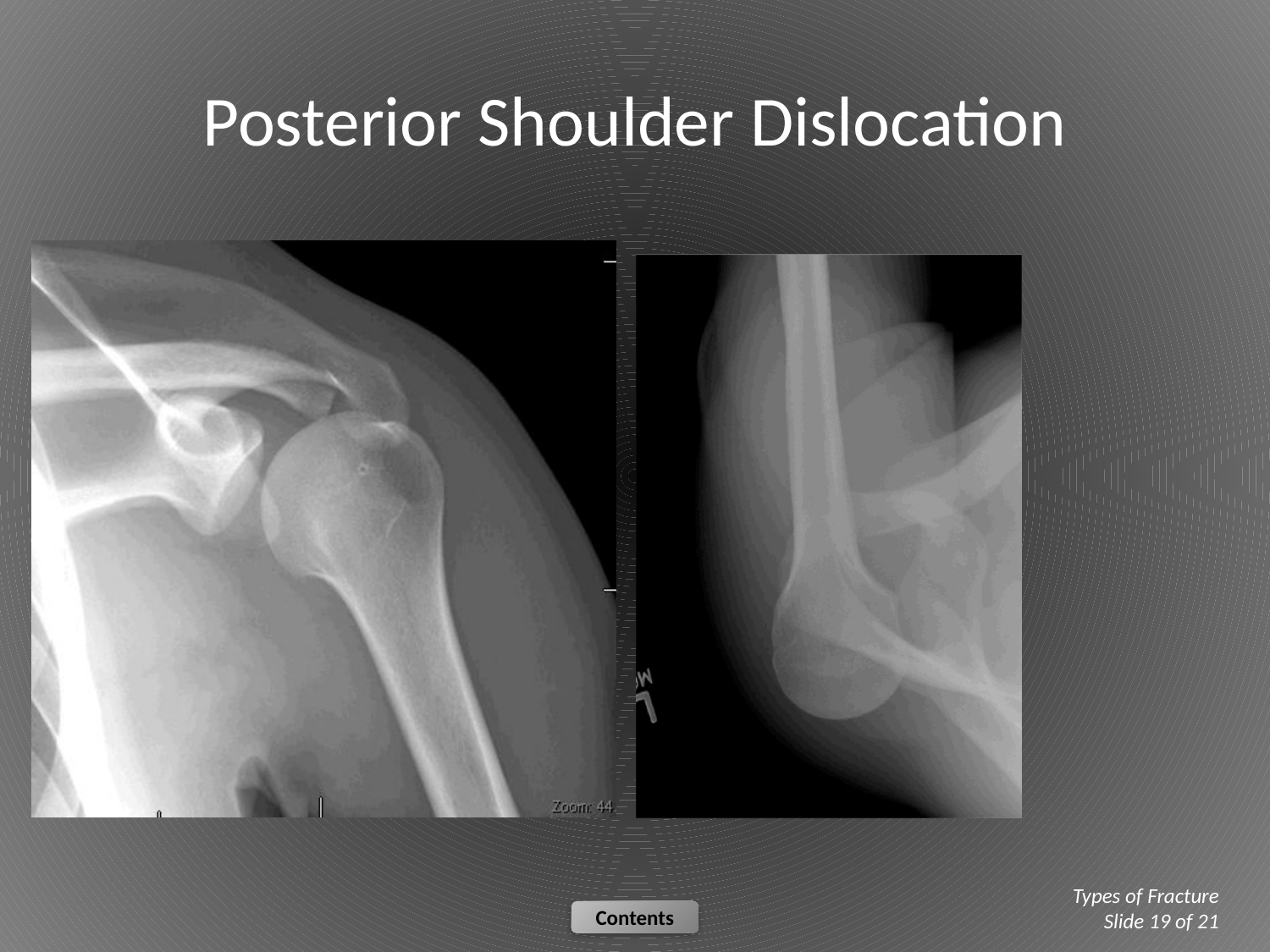

# Posterior Shoulder Dislocation
Types of Fracture
Slide 19 of 21
Contents

## Slide 37
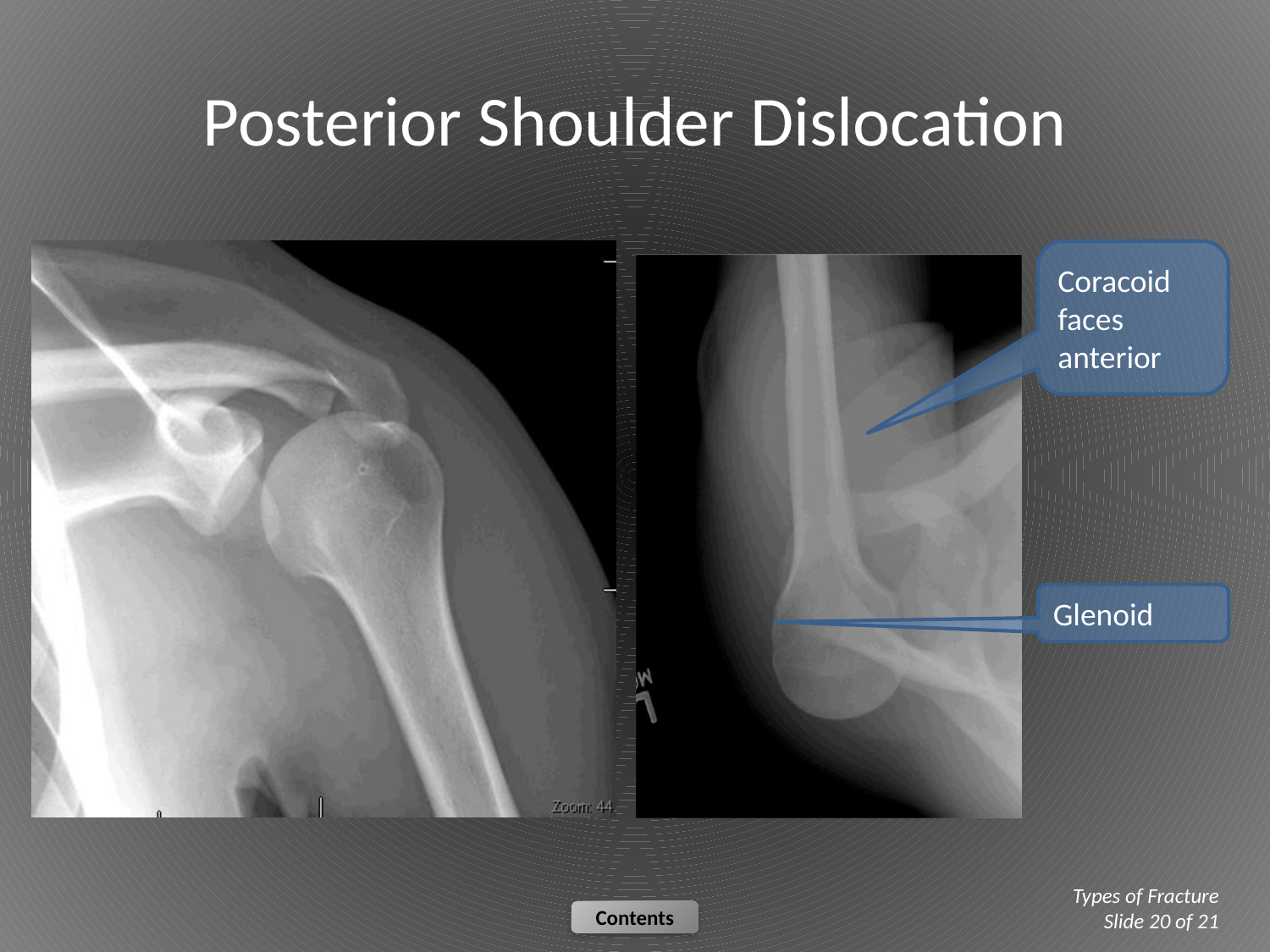

# Posterior Shoulder Dislocation
Coracoid faces anterior
Glenoid
Types of Fracture
Slide 20 of 21
Contents

## Slide 38
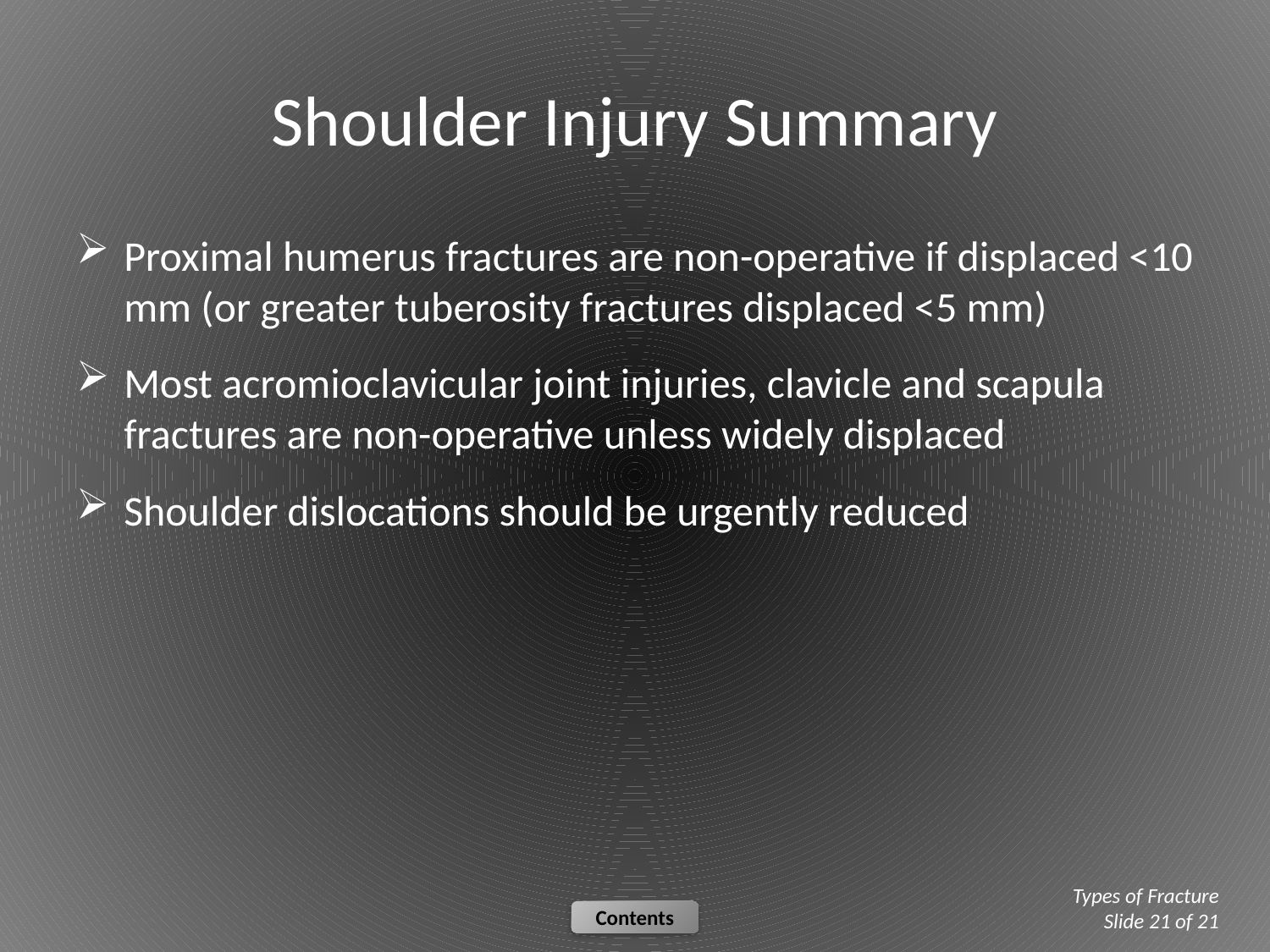

# Shoulder Injury Summary
Proximal humerus fractures are non-operative if displaced <10 mm (or greater tuberosity fractures displaced <5 mm)
Most acromioclavicular joint injuries, clavicle and scapula fractures are non-operative unless widely displaced
Shoulder dislocations should be urgently reduced
Types of Fracture
Slide 21 of 21
Contents

## Slide 39
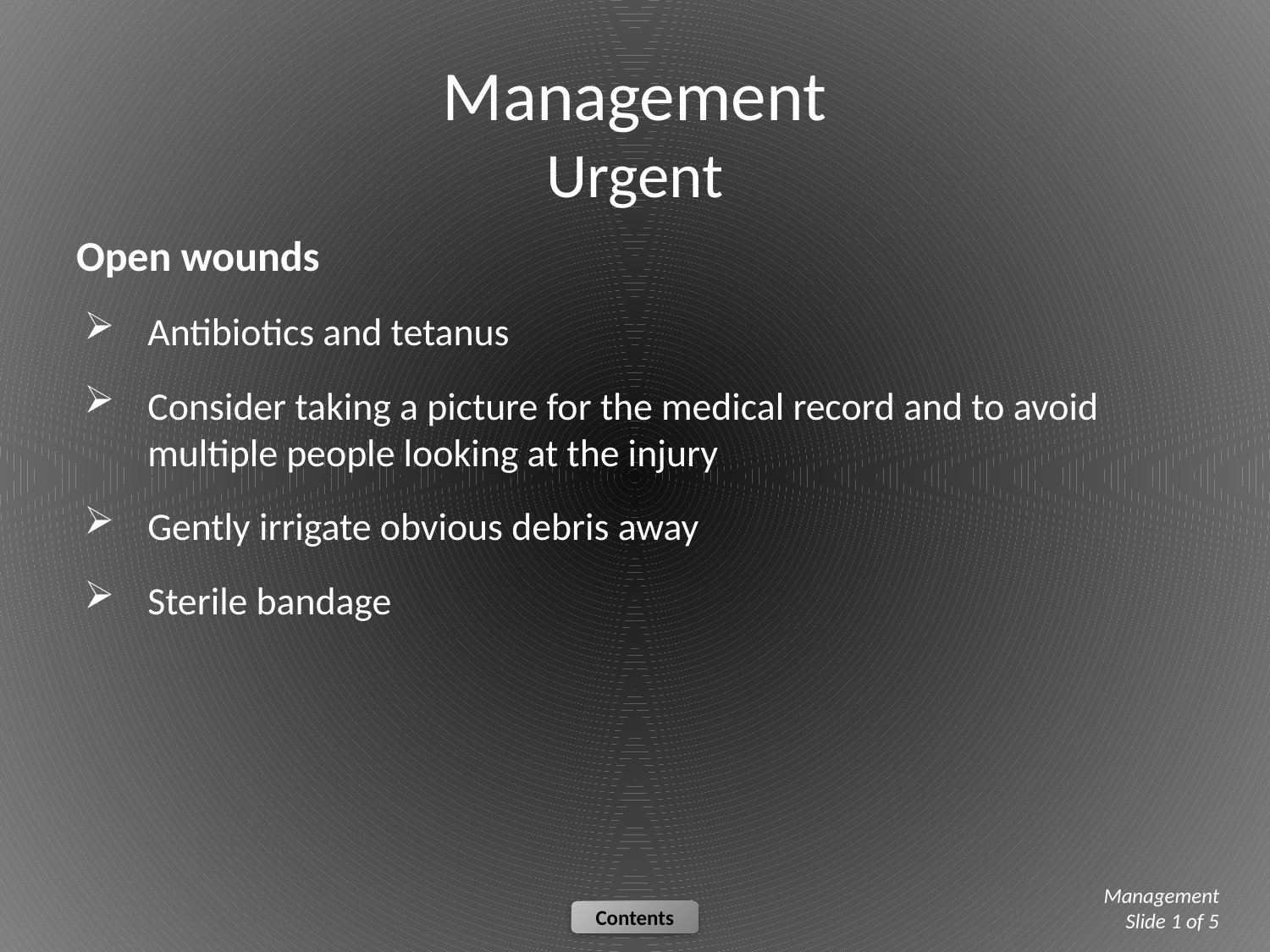

# Management Urgent
Open wounds
Antibiotics and tetanus
Consider taking a picture for the medical record and to avoid multiple people looking at the injury
Gently irrigate obvious debris away
Sterile bandage
Management
Slide 1 of 5
Contents

## Slide 40
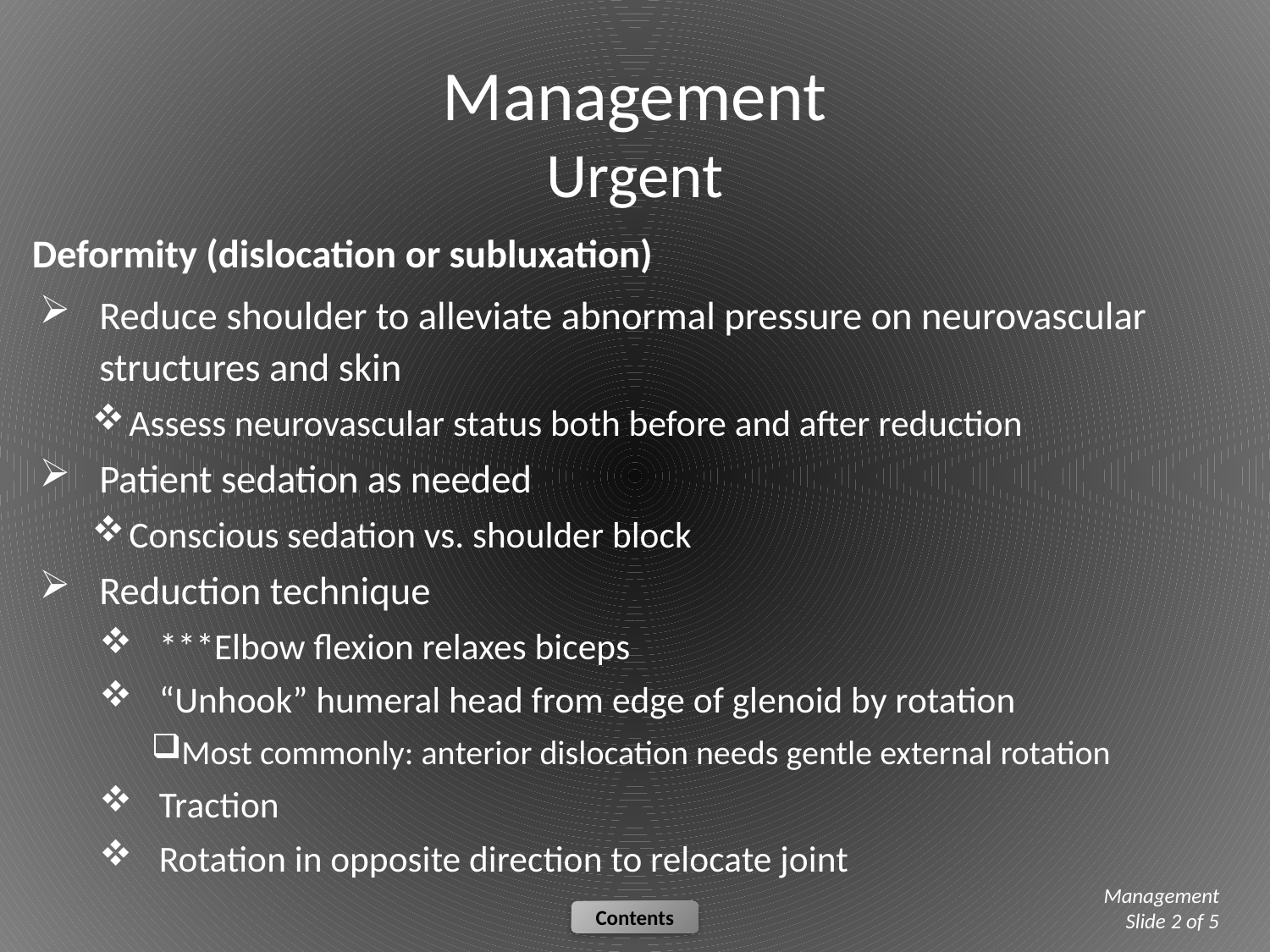

# Management Urgent
Deformity (dislocation or subluxation)
Reduce shoulder to alleviate abnormal pressure on neurovascular structures and skin
Assess neurovascular status both before and after reduction
Patient sedation as needed
Conscious sedation vs. shoulder block
Reduction technique
***Elbow flexion relaxes biceps
“Unhook” humeral head from edge of glenoid by rotation
Most commonly: anterior dislocation needs gentle external rotation
Traction
Rotation in opposite direction to relocate joint
Management
Slide 2 of 5
Contents

## Slide 41
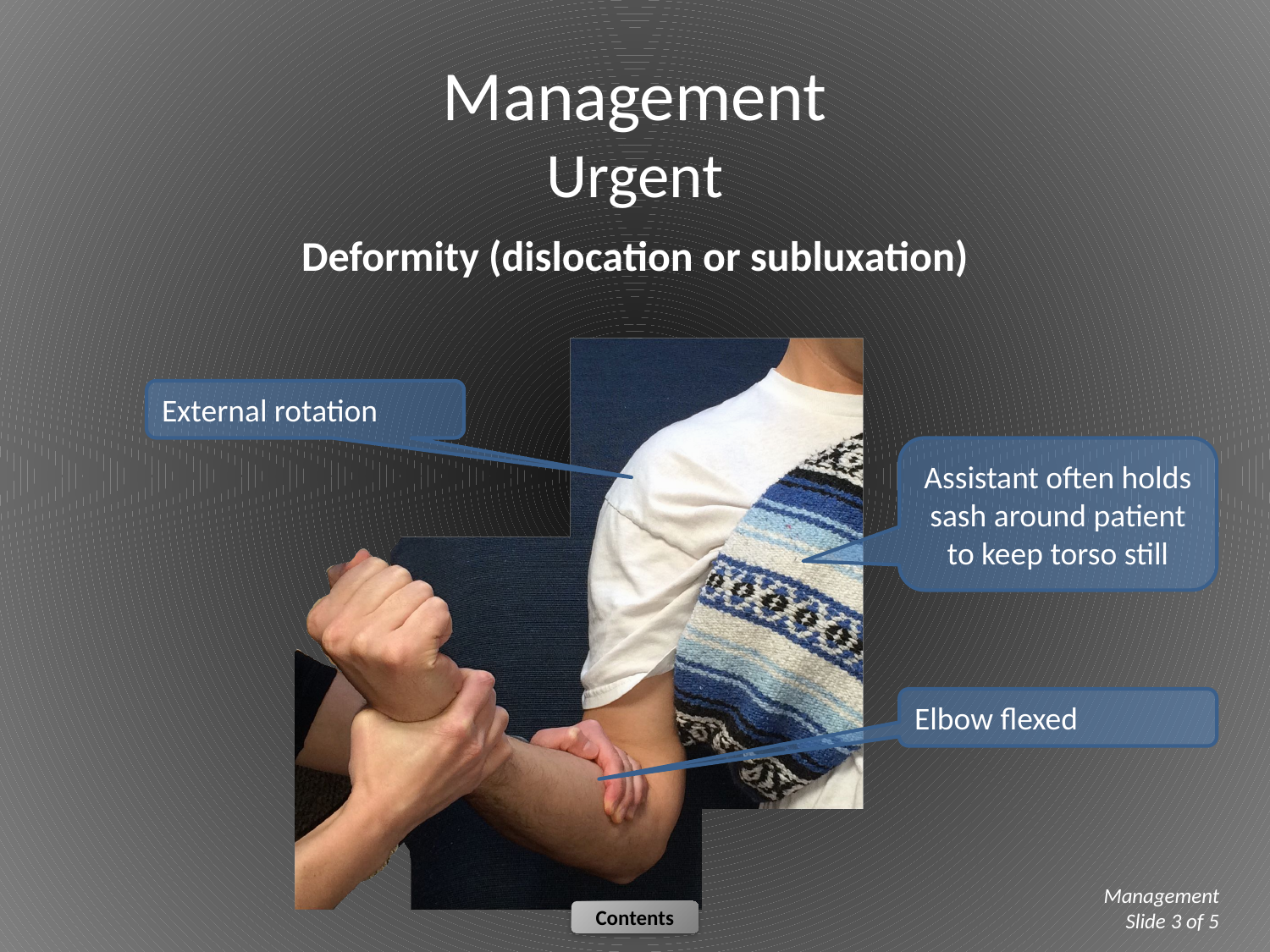

# Management Urgent
Deformity (dislocation or subluxation)
External rotation
Assistant often holds sash around patient to keep torso still
Elbow flexed
Management
Slide 3 of 5
Contents

## Slide 42
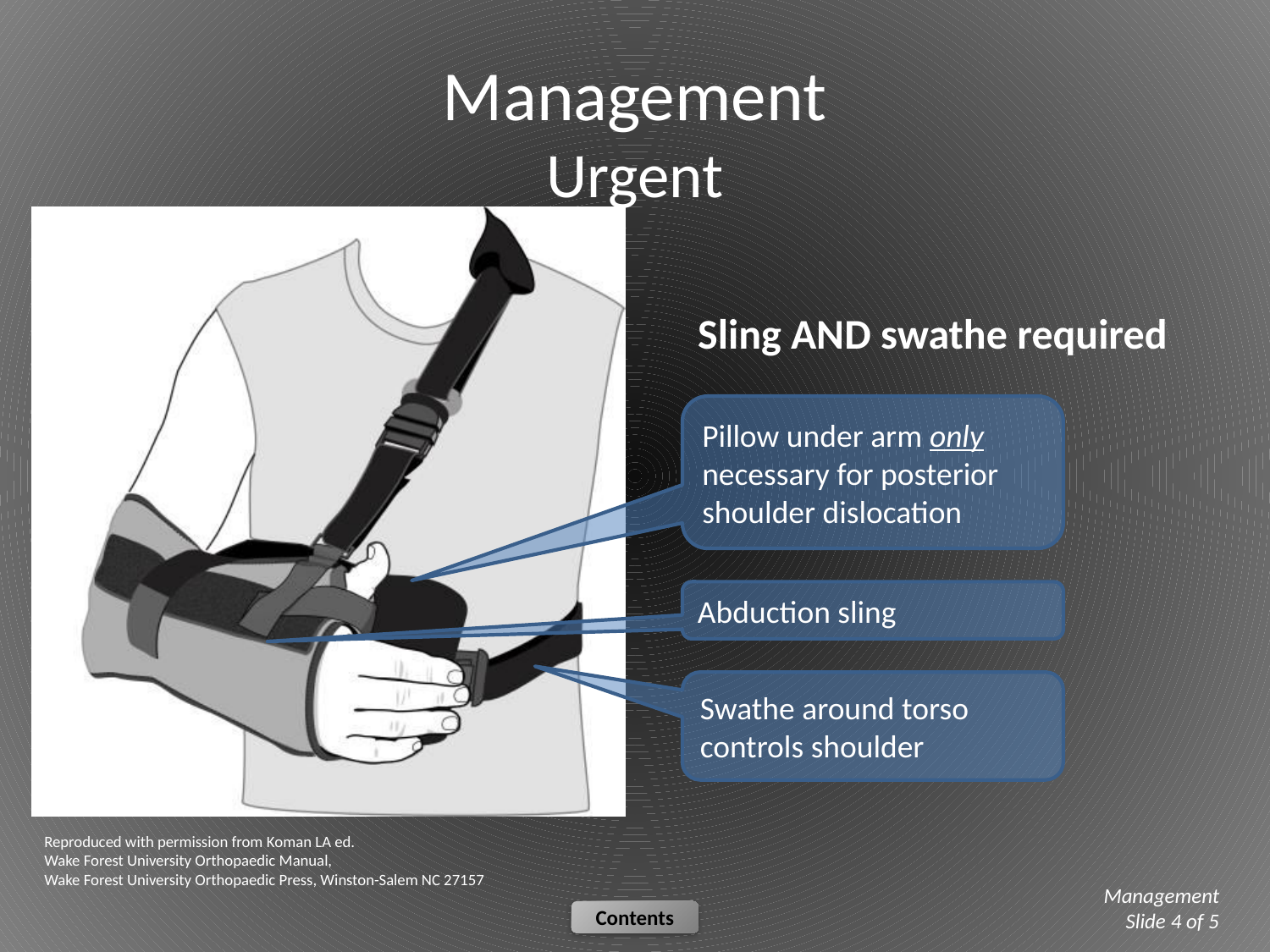

# Management Urgent
Sling AND swathe required
Pillow under arm only necessary for posterior shoulder dislocation
Abduction sling
Swathe around torso controls shoulder
Reproduced with permission from Koman LA ed.
Wake Forest University Orthopaedic Manual,
Wake Forest University Orthopaedic Press, Winston-Salem NC 27157
Management
Slide 4 of 5
Contents

## Slide 43
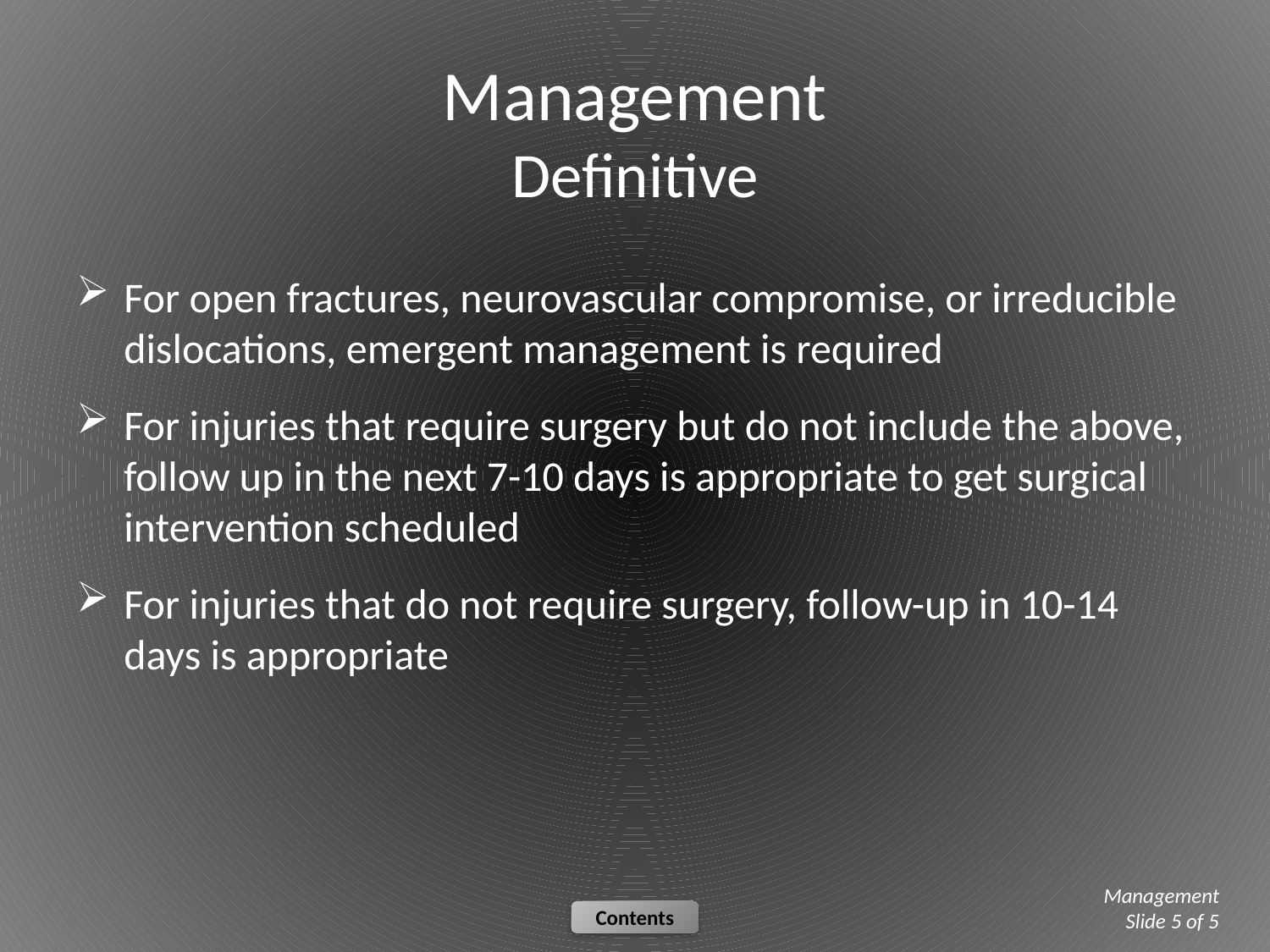

# Management Definitive
For open fractures, neurovascular compromise, or irreducible dislocations, emergent management is required
For injuries that require surgery but do not include the above, follow up in the next 7-10 days is appropriate to get surgical intervention scheduled
For injuries that do not require surgery, follow-up in 10-14 days is appropriate
Management
Slide 5 of 5
Contents

## Slide 44
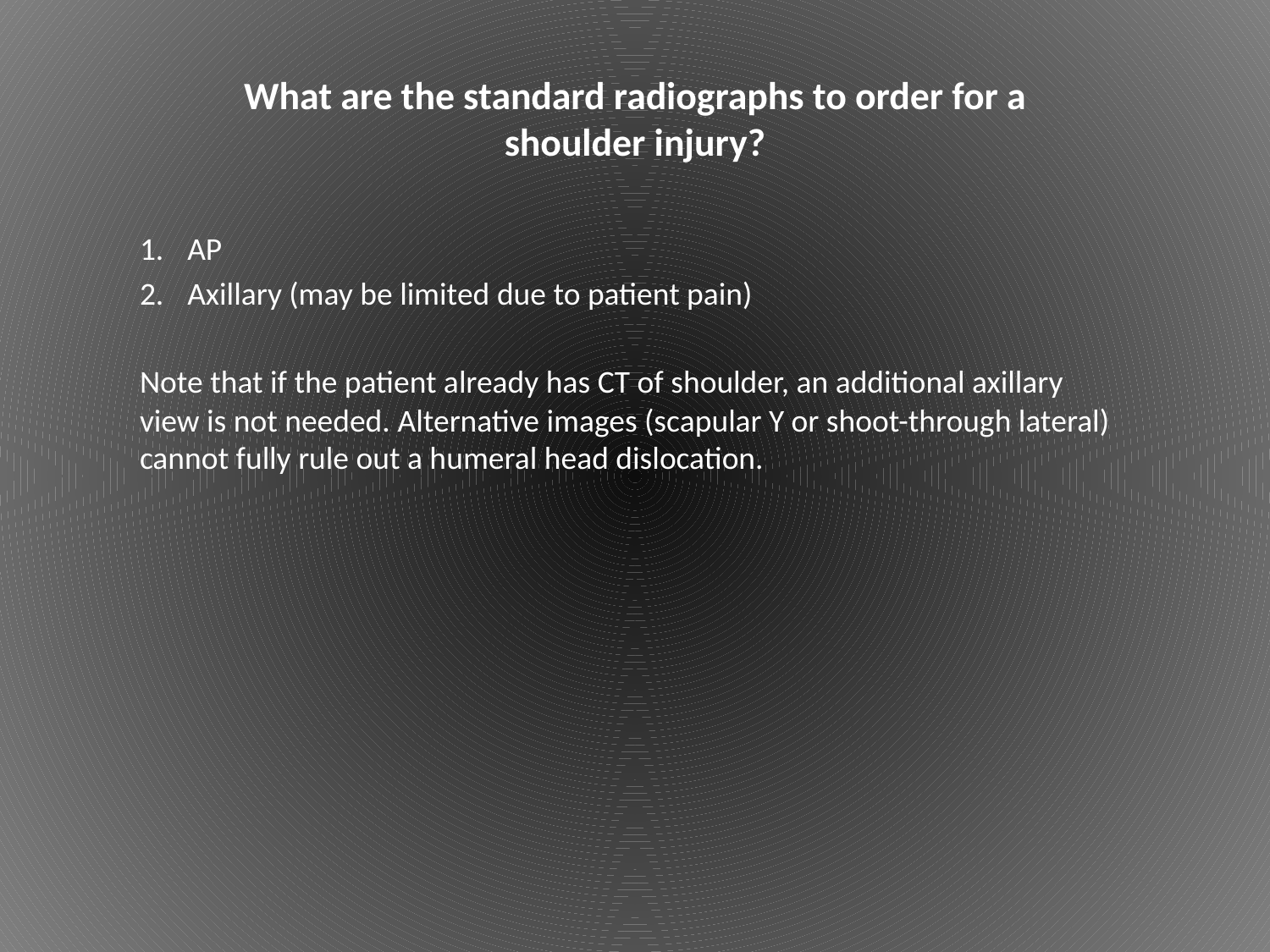

# What are the standard radiographs to order for a shoulder injury?
AP
Axillary (may be limited due to patient pain)
Note that if the patient already has CT of shoulder, an additional axillary view is not needed. Alternative images (scapular Y or shoot-through lateral) cannot fully rule out a humeral head dislocation.

## Slide 45
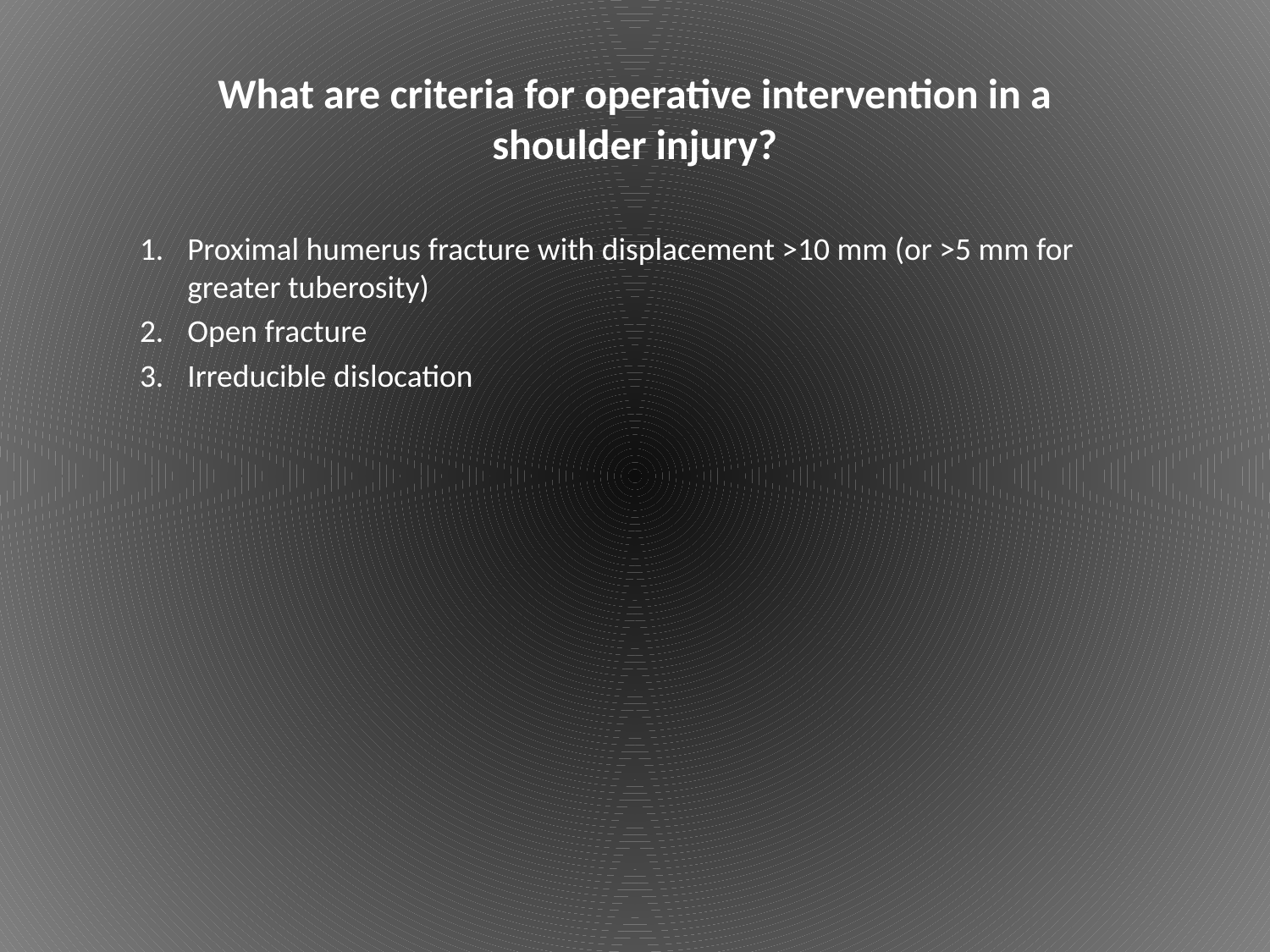

# What are criteria for operative intervention in a shoulder injury?
Proximal humerus fracture with displacement >10 mm (or >5 mm for greater tuberosity)
Open fracture
Irreducible dislocation

## Slide 46
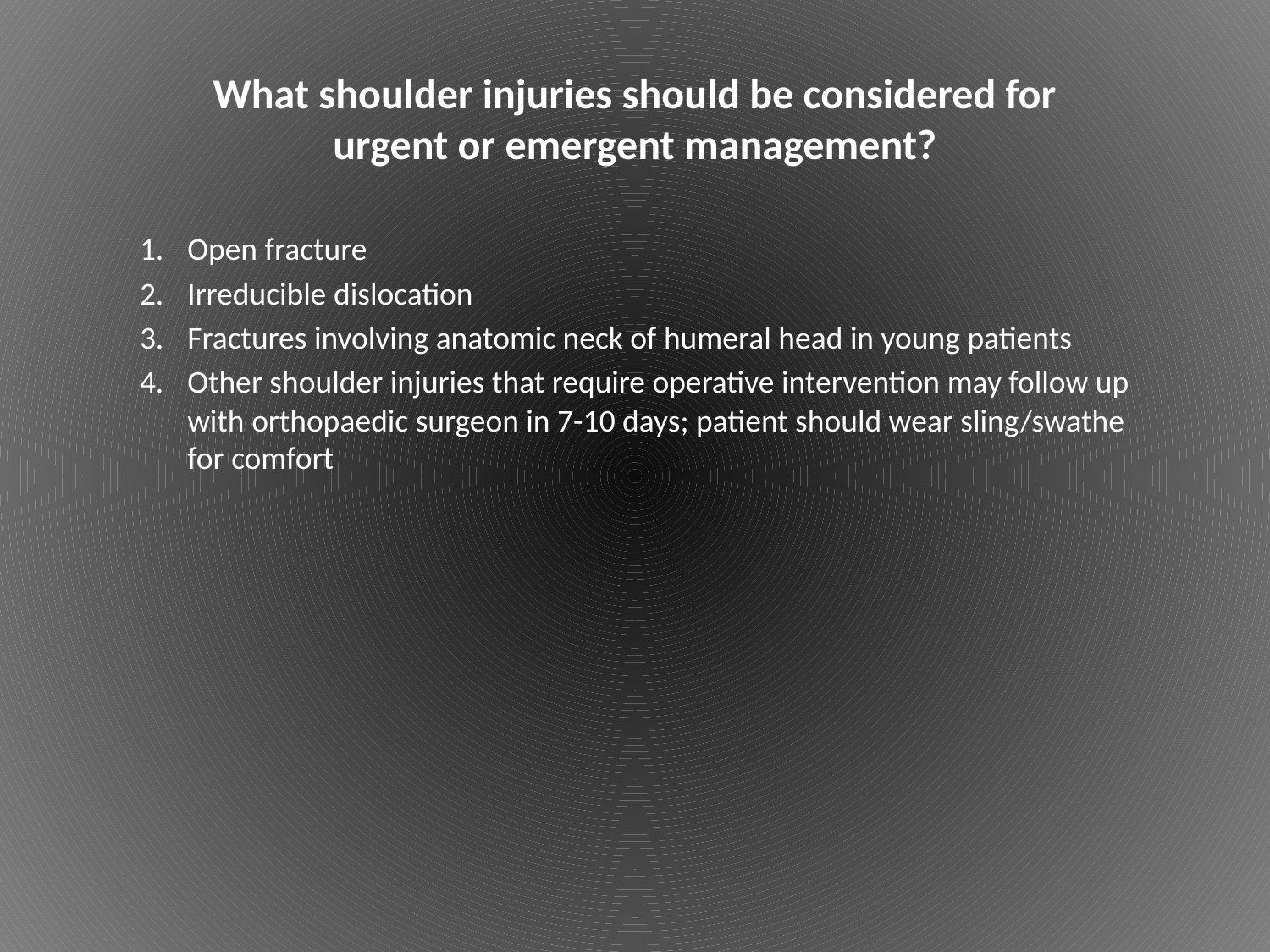

# What shoulder injuries should be considered for urgent or emergent management?
Open fracture
Irreducible dislocation
Fractures involving anatomic neck of humeral head in young patients
Other shoulder injuries that require operative intervention may follow up with orthopaedic surgeon in 7-10 days; patient should wear sling/swathe for comfort

## Slide 47
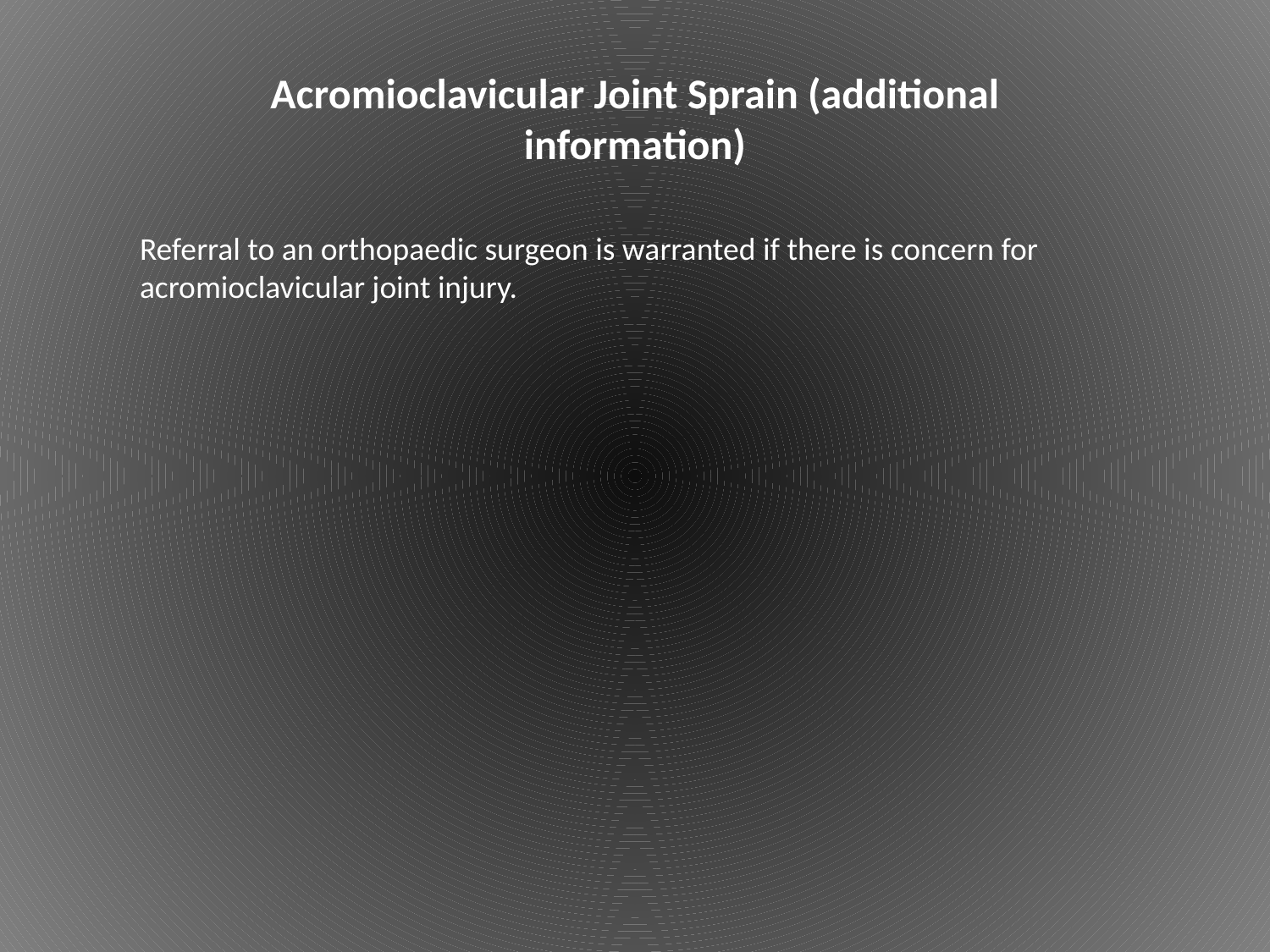

# Acromioclavicular Joint Sprain (additional information)
Referral to an orthopaedic surgeon is warranted if there is concern for acromioclavicular joint injury.
